# Examining variations in adherence to guideline-recommended post-acute kidney injury care in primary care in England: a population-based cohort study

**DOI:** 10.1186/s12916-026-05042-0

**Published:** 2026-08-12

**Authors:** Pearl L H Mok, Simon Sawhney, Tom Blakeman, Matthew J Carr, Rachel A Elliott, Robbie Foy, Simon DS Fraser, Evangelos Kontopantelis, Andrew J P Lewington, Alireza Mahboub-Ahari, Paul Roderick, Nicholas M Selby, Darren M Ashcroft

**Affiliations:** 1 Centre for Pharmacoepidemiology & Drug Safety, Division of Pharmacy & Optometry, Faculty of Biology, Medicine and Health, The University of Manchester, Manchester, UK; 2 NIHR Greater Manchester Patient Safety Research Collaboration, The University of Manchester, Manchester, UK; 3 Aberdeen Centre for Health Data Science, University of Aberdeen, Aberdeen, UK; 4 Division of Population Health, Health Services Research & Primary Care, The University of Manchester, Manchester, UK; 5 Manchester Centre for Health Economics, Division of Population Health, Health Services Research & Primary Care, The University of Manchester, Manchester, UK; 6 Leeds Institute of Health Sciences, University of Leeds, Leeds, UK; 7 School of Primary Care, Population Sciences and Medical Education, Faculty of Medicine, University of Southampton, Southampton General Hospital, Southampton, UK; 8 Division of Informatics, Imaging and Data Sciences, The University of Manchester, Manchester, UK; 9 Division of Family Medicine, Yong Loo Lin School of Medicine, National University of Singapore, Singapore, 119228 Singapore; 10 Centre for Research in Health Systems Performance, National University of Singapore, Singapore, Singapore; 11 Leeds Teaching Hospitals NHS Trust, Leeds, UK; 12 Centre for Kidney Research and Innovation, Academic Unit of Translational Medical Sciences, School of Medicine, University of Nottingham, Nottingham, UK

**Keywords:** Acute kidney injury, Kidney diseases, Primary care, Post-discharge care, Transition of care

## Abstract

**Background:**

Acute kidney injury (AKI) is a clinical syndrome characterised by a sudden deterioration of kidney function. It is common and usually occurs as a complication of severe illness or major surgery. Despite the high risk of complications and a decade of improvement initiatives in the UK, little is known about the quality of post-discharge AKI care. Our population-based cohort study investigated adherence to guideline-recommended post-AKI care in general practices in England.

**Methods:**

Using English hospital admission data (2017–2021), we created a cohort of discharged patients (≥ 18 years) with a hospital diagnostic code of AKI. Using linked Clinical Practice Research Datalink Aurum primary care data, we examined percentages of AKI episodes meeting the criteria of 14 guideline-recommended post-AKI care indicators, covering: AKI coding in primary care, post-discharge primary care contacts, kidney health and blood pressure monitoring, and guideline-indicated prescribing. Variations of indicator adherence according to patient characteristics were quantified using binomial mixed regression.

**Results:**

209,222 patients (48.0% females; mean age 74.1 years) were included, representing 279,187 AKI inpatient episodes. Only 19.5% (95% CI 18.5–20.5) of episodes had AKI coded in primary care within 30 days of discharge, while 72.6% (95% CI 71.8–73.4) had a documented contact with general practice. At 90 ± 30 days after discharge, serum creatinine was measured in 34.2% (95% CI 33.7–34.8) of episodes, blood pressure in 34.6% (95% CI 34.0-35.1), and albumin-creatinine ratio in 4.2% (95% CI 4.0-4.4). Testing was less common amongst younger patients and those without comorbid conditions. Renin-angiotensin system inhibitor prescribing rates were low in patients likely to benefit.

**Conclusions:**

There are multiple missed opportunities for improving post-AKI care. Rates of measuring albuminuria were particularly low, despite its strong association with subsequent kidney and cardiovascular events. The limited post-AKI clinical activity amongst younger patients and those without comorbidities undermines the prevention and early intervention of chronic kidney diseases. Clearer discharge arrangements, including case-specific guidance on discharge summaries, and the development and evaluation of concerted implementation strategies spanning secondary and primary care are needed.

**Supplementary Information:**

The online version contains supplementary material available at 10.1186/s12916-026-05042-0.

## Background

Acute kidney injury (AKI) is a clinical syndrome characterised by a sudden deterioration of kidney function [1]. It affects around one in five hospitalised adults worldwide and usually occurs as a complication of severe illness or major surgery; older people living with multiple long-term conditions and those in intensive care are particularly vulnerable [1–3]. Kidney function recovery is often incomplete at the time of discharge [4]. There are associated increased risks of all-cause mortality, emergency readmission including with acute heart failure, development of new or worsening of existing chronic kidney disease (CKD), and major adverse cardiovascular events [1, 5, 6]. Timely post-discharge care following AKI is thus crucial, and primary care is central to such care provision and management [7, 8].

Over the last decade, a range of initiatives have been introduced, and clinical guidelines developed in the UK and internationally to improve AKI awareness, detection, and quality of care in both hospital and community settings [9–18]. Mandatory incorporation of AKI alerts in test reporting across all laboratories in England has not reduced significant variations between hospitals in AKI care quality [10]. For post-discharge care, key guideline recommendations include conducting timely clinical reviews, optimising medicines management, and monitoring patients three months after AKI to identify new or worsening CKD [9, 11–18]. However, there is limited knowledge about adherence to these guidelines.

This study investigated variations in adherence to guideline-recommended post-AKI care in general practices in England. We developed and operationalised 14 post-AKI care indicators informed by national and international guidelines [9, 11–18]. These covered domains of (1) coding of AKI in primary care, (2) post-discharge primary care clinical contacts, (3) assessment of kidney health and blood pressure, and (4) guideline-indicated prescribing of medications with cardiovascular prognostic benefit as well as avoiding prescribing of potentially nephrotoxic non-steroidal anti-inflammatory drugs (NSAIDs). We evaluated care quality overall, and within subgroups based on demographic, socioeconomic, and clinical characteristics. We also examined degree of variations in indicator adherence between practices and indicator reliability.

## Methods

### Data source, study population and design

Our population-based study used linked anonymised electronic health records from the Clinical Practice Research Datalink (CPRD) Aurum database and Hospital Episode Statistics (HES). CPRD Aurum is sourced from general practices in England which use the EMIS Web^®^ software, the most commonly used primary care electronic patient record management software system in the UK [19, 20]. CPRD operates an ‘opt-in’ approach whereby practices are invited to contribute their data to the CPRD database. Around 20% of UK practices agreed to do so, covering a quarter of the UK population [19, 20]. Practices can opt out of contributing their data at any time if they wish. CPRD data contains information on diagnoses, primary health care contacts, prescribed medications, referrals, and test results. The data are broadly representative of the English population in terms of age, gender, ethnicity, geographical spread, and patients’ socioeconomic status at the small area level [20–22]. HES data include admissions, outpatient appointments and emergency department attendances at all National Health Services (NHS) hospitals in England. We also obtained CPRD-HES linked ethnicity records, Office for National Statistics-linked mortality records, and Index of Multiple Deprivation (IMD) quintiles data, which provide a small area-level measure of relative deprivation based on patient residential postcodes. Linkages of CPRD primary care data with these datasets were carried out by CPRD’s ‘Trusted Third Party’, NHS England, which is the statutory body in England legally permitted to receive identifiable patient data [23]. Study approval was granted through the CPRD Research Data Governance Process (protocol 22_001760).

Our study cohort was defined using HES Admitted Patient Care (APC) data. Patients were included if they had a recorded hospital admission with AKI, defined using International Classification of Disease 10th revision (ICD-10) codes N17 (acute renal failure) or O90.4 (postpartum acute renal failure), were aged 18 years or over, and were discharged from hospital between 1st January 2017 and 31st March 2021. To ascertain hospital discharge dates, continuous inpatient spells were constructed based on the methodology developed by the Centre for Health Economics, University of York [24]. Patients needed to have at least 6 months of continuous registration with the general practice before hospital discharge and be eligible for linkage to HES APC, HES outpatient (available up to 31st Oct 2020), HES accident and emergency, mortality and IMD data. Patients who were discharged to a destination not covered by primary care, and those with kidney failure - CKD stage 5, kidney transplant, or chronic dialysis – recorded at any time before hospital discharge, were excluded. Additional file 1: Additional Information S1 shows the list of discharged destinations included, and Additional file 1: Figure S1 shows how the study cohort was delineated. Patients were followed from the date of discharge and censored at the earliest of these dates: transfer out of general practice, last data collection of the general practice, first coding of kidney failure, or death.

### Post-AKI care indicators and outcome measures

The post-AKI care indicators investigated are shown in Box 1. Outcomes were the percentages of AKI episodes meeting the indicator criteria. Additional file 1: Table S1 shows how the numerators and denominators of the percentages were defined.

**Box 1:** Post-AKI care indicators investigated

***Coding of AKI in primary care***		References
1	Coding of AKI in the primary care record within 30 days after discharge.	[12, 14, 15]
2	Coding of AKI in the primary care record within 90 days after discharge.	
***Primary care clinical contacts***		
3	Face-to-face contact with a doctor, nurse, or pharmacist at the general practice within 30 days after discharge.	[11, 14, 15]
4	Face-to-face or telephone contact with a doctor, nurse, or pharmacist at the general practice within 30 days after discharge.	
***Monitoring of kidney health status and blood pressure***		
5	Serum creatinine measurement at 90 ± 30 days after discharge.	[5, 9, 14–18, 25]
6	Serum creatinine measurement at 365 ± 90 days after discharge.	
7	Albumin/creatinine ratio (ACR) measurement at 90 ± 30 days after discharge.	[5, 9, 14–16, 18, 25–27]
8	ACR measurement at 365 ± 90 days after discharge.	
9	Blood pressure measurement at 90 ± 30 days after discharge.	[9, 16, 18]
10	Blood pressure measurement at 365 ± 90 days after discharge.	
11	Coding of *de novo* CKD in the primary care record at 90 ± 30 days after discharge – *de novo* CKD is defined as estimated glomerular filtration rate (eGFR) measured at 90 ± 30 days after discharge < 60 mL/min/1.73m^2^ with no previous CKD code.	[9, 16, 18]
***Guideline indicated prescribing***		
12	Renin–angiotensin–aldosterone system inhibitors (RAASi) continuation – Prescribing of RAASi within 90 days after discharge for those with indication and who had at least one prescription in the 90 days before admission. Indication was defined as a history of diabetes and ACR ≥3 mg/mmol, hypertension and ACR ≥ 30 mg/mmol, or ACR ≥ 70 mg/mmol. RAASi include angiotensin-converting enzyme inhibitors (ACE inhibitors), angiotensin receptor blockers (ARBs), and combined formulations containing these.	[13–15, 28, 29]
13	RAASi initiation – Prescribing of RAASi within 90 days after discharge for those with indication (as described above) and were not prescribed these drugs in the 90 days before admission.	[13–15, 28, 29]
14	Avoidance of prescription of nonsteroidal anti-inflammatory drugs (NSAIDs) within 90 days after discharge.	[13, 14]

### Covariates

We investigated variations in outcomes by age (< 60, 60–79, >=80 years), sex, ethnicity (White, Asian, Black, Other, Unknown), IMD quintiles (1 to 5, Unknown), prior CKD (excluding stage 5), diabetes, heart failure and myocardial infarction, major surgery and sepsis during the AKI episode, outpatient contact with General Surgery, Endocrinology and Diabetes, Cardiology, or Renal Medicine within 30 and 90 days after discharge, and period of discharge (1st January 2017-31st March 2018, 1st April 2018-31st March 2019, 1st April 2019-31st March 2020, and 1st April 2020-31st March 2021 which covered approximately the first 12 months of the Covid-19 pandemic). Where applicable, variations according to whether there was primary care AKI coding or face-to-face contact with clinical staff at the general practice within 30 days of discharge, were also investigated.

Outcomes and covariates were identified using Read, Systematized Nomenclature of Medicine (SNOMED), EMIS or product codes in CPRD Aurum, and/or ICD10 and Office of Population Censuses and Surveys (OPCS) Classification of Interventions and Procedures coding in HES APC. Code lists are available in Additional file 1: pages 44–141.

### Statistical analysis

The percentage of AKI episodes meeting each post-AKI care indicator was calculated with 95% CIs using bootstrap, with sampling at patient and practice levels. Variations in outcomes according to demographics and clinical characteristics were quantified using 3-level binomial mixed regression models, adjusting for age, sex, calendar month of discharge (1–12 as a categorical variable), count of months during period of discharge January 2017-March 2021 (1–51 as a continuous variable), and with random intercepts at patient and practice levels. Results were reported as odds ratios (ORs) with 95% CI. Analyses were performed using Stata v17.

To quantify variations in indicator adherence between practices and between patients, we reported the practice- and patient-level intraclass correlation coefficients (ICC, ranging from 0 to 1), respectively, for each indicator. These were derived from the models, adjusting for age, sex, calendar month and count of months during period of discharge. Higher practice-level ICCs indicate large variation between practices in adherence to the indicator, even for patients with similar characteristics. Higher patient-level ICCs indicate greater variations in indicator adherence at the patient level and relatively consistent adherence within individuals across episodes, i.e. greater between-patient heterogeneity such as in patient characteristics but smaller differences in patterns of care across episodes for a patient. Further information is provided in Additional file 1: Additional Information S2 [30, 31].

We also examined the reliability of each indicator, which quantifies how much of the observed variation is due to practice differences, compared with within-subject variation driven by differences in patient characteristics [32–34]. Reliability was calculated using the following formula for a 3-level model [35]:

$$\begin{array}{l}reliability=\frac{{{\upsigma}}^{2}\left(practice\right)}{{{\upsigma}}^{2}\left(practice\right)+\frac{{{\upsigma}}^{2}\left(patient:practice\right)}{{n}_{k}}+\frac{{{\upsigma}}^{2}\left(AKI\,episodes:patients:practice\right)}{{n}_{k}{n}_{jk}}}\end{array}$$

where:

σ^2^ *(practice)* = variance between practices.

σ^2^ *(patient: practice)* = variance between patients within practices.

σ^2^ *(AKI episodes: patient: practice)* = variance of AKI episodes amongst patients and practices, which is π^2^/3 for a binomial mixed regression model.

n_k_ = number of patients in practice k eligible for inclusion in the analysis of the indicator.

n_jk_ = number of AKI episodes for patient j in practice k.

The larger the variance between practices, and the larger the number of patients and AKI episodes in a practice, the higher the reliability. Reliability ranges between 0 and 1, with 0.7 being generally considered as acceptable. Because each practice has its own reliability value for each indicator, to summarise the reliability for each indicator, we reported the reliability for a hypothetical practice with median numbers of eligible patients and of AKI episodes [32, 33]. For further information, see Additional file 1: Additional Information S3.

### Patient and public involvement

A Patient and Public Involvement and Engagement group was established, which included people with lived experience of AKI and a carer. They contributed to the funding application, study design, discussed study conduct and progress with the research team, and contributed to interpretation of the data by providing feedback from patient and carer perspectives.

## Results

### Study cohort

The study cohort comprised 209,222 patients (48.0% females; mean age 74.1 years, SD 15.6) from 1470 general practices, representing 279,187 AKI inpatient episodes. Table 2 details patient demographic and clinical characteristics. Diabetes, CKD, heart failure, and myocardial infarction were common amongst this population.

**Table 2:** Demographic and clinical characteristics of patients in study cohort

Number of patients discharged from hospital after an AKI diagnosis	209,222
Number of AKI episodes	279,187
*Sex (% patients)*	
Female	100,328 (48.0)
Male	108,894 (52.0)
*Ethnicity (% patients)* ^a^	
White	189,251 (90.5)
Asian	9741(4.7)
Black	7103 (3.4)
Other	1307 (0.6)
Unknown	1820(0.9)
*Age at discharge (% AKI episodes)*	
Less than 60 years	45,421 (16.3)
60–79 years	110,354 (39.5)
80 years or over	123,412(44.2)
Mean (standard deviation)	74.1 (15.6)
Median	77
*Index of multiple deprivation quintiles (% AKI episodes)*	
1 (least deprived)	49,929 (17.9)
2	53,793 (19.3)
3	53,623 (19.2)
4	57,642 (20.6)
5 (most deprived)	63,078 (22.6)
Unknown	1122 (0.4)
*Period of discharge (% AKI episodes)* ^a^	
31st January 2017 to 31st March 2018	79,560 (28.5)
30th April 2018 to 31st March 2019	66,865 (23.9)
30th April 2019 to 31st March 2020	68,476 (24.5)
30th April 2020 to 31st March 2021	64,286 (23.0)
*Clinical history (% AKI episodes)* ^b^	
CKD (excluding stage 5)	151,449 (54.2)
Diabetes	104,068 (37.3)
Heart failure	91,604 (32.8)
Myocardial infarction	58,889 (21.1)
Major surgery during AKI episode	10,756 (3.9)
Sepsis during AKI episode	43,957 (15.7)
*Outpatient contact with General Surgery*,* Endocrinology and Diabetes*,* Cardiology*,* or Renal Medicine (% AKI episodes)* ^c^	
Within 30 days of discharge	26,968 (11.0) ^d^
Within 90 days of discharge	55,867 (23.5) ^e^

^a^ Percentages did not sum to 100.0% exactly because of rounding to one decimal place

^b^ CKD, diabetes, heart failure, and myocardial infarction recorded at any time up to and including the date of discharge

^c^ Outpatient data only available up to 31st Oct 2020

^d^ Percentage of AKI episodes (*n* = 245,886) with discharged dates up to 30th Sept 2020

^e^ Percentage of AKI episodes (*n* = 237,233) with discharged dates up to 31st July 2020

### Adherence to post-AKI care indicators

Table 3 shows the percentages of AKI episodes meeting post-AKI care indicator criteria. Variations of indicator adherence by demographic and clinical characteristics are shown in Fig. 1, Additional file 1: Figures S2-S14 and Tables S2-S9.

**Fig. 1:**
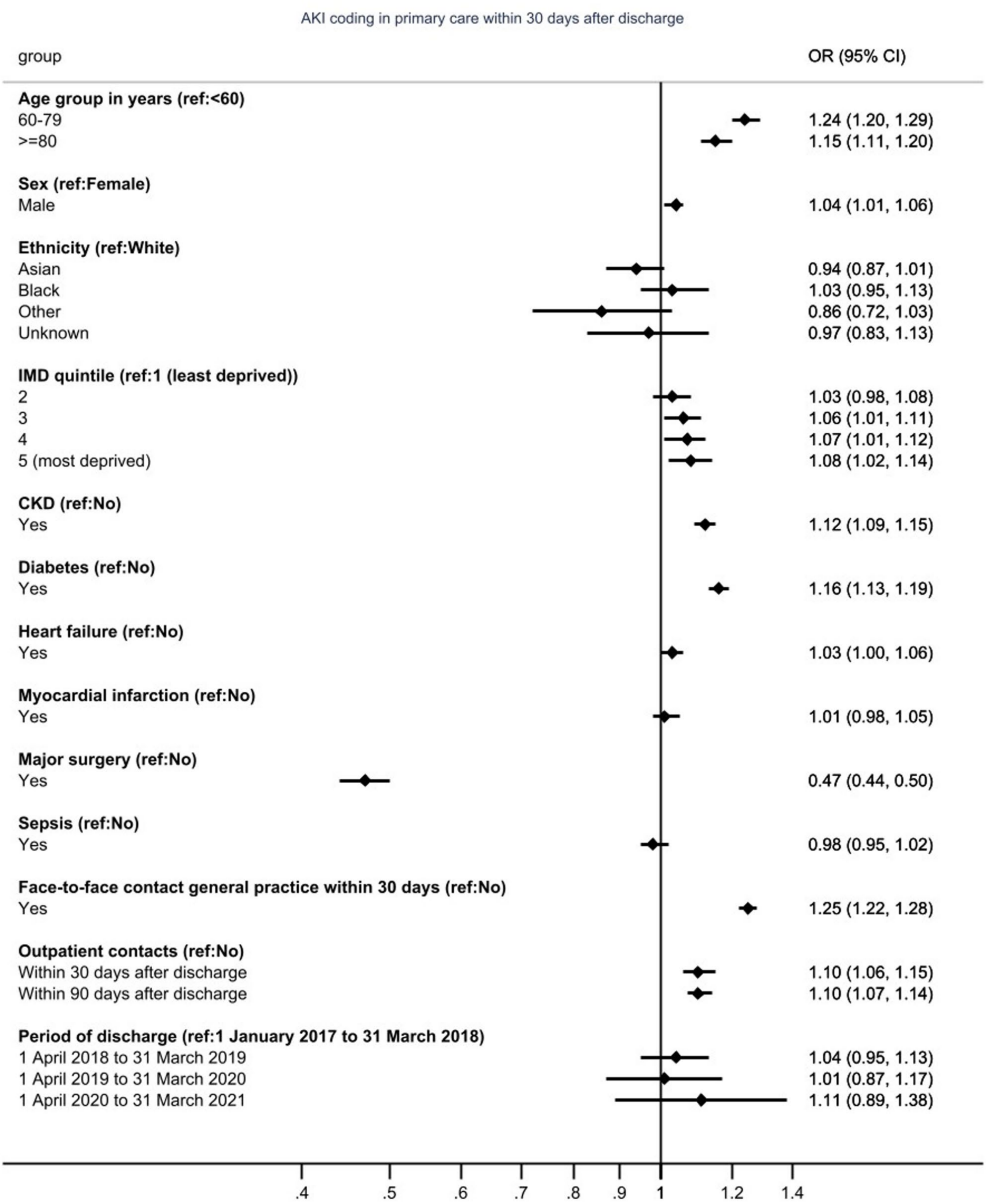
Odds ratios (95% CI) of AKI episodes recorded in primary care records within 30 days after discharge, by demographics, socioeconomics, and clinical characteristics. ORs adjusted for age, sex, month count (1–51) and calendar month (1–12) of discharge, and practice and patient-level clustering. ORs for unknown IMD quintile not shown in figure because of small number of episodes. CKD (excluding stage 5), diabetes, heart failure, and myocardial infarction: recorded at any time up to and including the date of discharge. Major surgery and sepsis: recorded during AKI episode. Face-to face contact at general practice within 30 days of discharge: with doctor, nurse, or pharmacist. Outpatient contacts with General Surgery, Endocrinology and Diabetes, Cardiology, or Renal Medicine . data available up to 31st Oct 2020

Only 19.5% (95% CI 18.5–20.5) of hospital AKI episodes had a diagnosis of AKI recorded in the primary care records within 30 days after discharge, while 72.6% (95% CI 71.8–73.4) had recorded face-to-face or telephone contact with a doctor, nurse, or pharmacist at the general practice during this period. Within 90 days after discharge, the percentage of episodes with primary care AKI coding remained low at 22.0% (95% CI 21.0-23.1). Patients aged under 60 and those who had major surgery were less likely than older patients and those without major surgery to have their AKI diagnosis coded (Fig. 1, Additional file 1: Table S2 and Fig. S2). During the Covid-19 pandemic April 2020-March 2021, patients were less likely to have face-to-face contacts and more likely to have telephone contacts with clinical staff at the general practice (Additional file 1: Table S3 and Figs. S3 and S4).

**Table 3:** Number and percentages (95%CI) of AKI episodes meeting post-AKI care indicators

Post-AKI care indicators	Number of AKI episodes meeting indicator criteria / Number of AKI episodes eligible for inclusion	Percentage of AKI episodes meeting indicator criteria (95% CI)	Practice-level ICC (95% CI) ^a^	Patient-level ICC (95% CI) ^a^	Reliability of a practice with median number of eligible patients and median number of AKI episodes	% practices with reliability > 0.7
*Recording of AKI in primary care*						
Coding of AKI in primary care record within 30 days after discharge	47,951 / 245,947	19.5 (18.5 to 20.5)	0.23 (0.21 to 0.24)	0.43 (0.41 to 0.44)	0.97	99.3
Coding of AKI in primary care record within 90 days after discharge	48,348 / 219,714	22.0 (21.0 to 23.1)	0.23 (0.21 to 0.24)	0.55 (0.54 to 0.57)	0.97	99.0
*Primary care clinical contacts within 30 days after discharge*						
Face-to-face contact with a doctor, nurse, or pharmacist at the general practice	126,928 / 245,947	51.6 (50.7 to 52.5)	0.08 (0.07 to 0.09)	0.37 (0.35 to 0.38)	0.92	95.9
Face-to-face or telephone contact with a doctor, nurse, or pharmacist at the general practice	178,591 / 245,947	72.6 (71.8 to 73.4)	0.07 (0.06 to 0.07)	0.38 (0.36 to 0.39)	0.90	94.0
*Monitoring of kidney health status and blood pressure at 90+/-30 days after discharge*						
Creatinine measurement	72,186 / 210,792	34.2 (33.7 to 34.8)	0.02 (0.02 to 0.02)	0.42 (0.41 to 0.43)	0.70	49.6
ACR measurement	8905 / 210,792	4.2 (4.0 to 4.4)	0.05 (0.04 to 0.06)	0.54 (0.51 to 0.56)	0.84	83.8
Blood pressure measurement	72,844 / 210,792	34.6 (34.0 to 35.1)	0.03 (0.02 to 0.03)	0.38 (0.37 to 0.39)	0.73	58.6
Coding of *de novo* CKD in primary care records	1455 / 8489	17.1 (15.5 to 18.8)	0.18 (0.14 to 0.22)	0.84 (0.79 to 0.87)	0.47	12.2
*Monitoring of kidney health status and blood pressure at 365+/-90 days after discharge*						
Creatinine measurement	89,971 / 157,712	57.0 (56.3 to 57.7)	0.03 (0.02 to 0.03)	0.63 (0.62 to 0.64)	0.69	46.7
ACR measurement	19,212 / 157,712	12.2 (11.8 to 12.6)	0.05 (0.04 to 0.05)	0.74 (0.72 to 0.75)	0.79	71.8
Blood pressure measurement	90,207 / 157,712	57.2 (56.4 to 57.9)	0.03 (0.03 to 0.03)	0.59 (0.58 to 0.60)	0.70	51.5
*Guideline indicated prescribing within 90 days after discharge*						
RAASi continuation	14,811 / 20,985	70.6 (69.3 to 71.8)	0.02 (0.02 to 0.04)	0.56 (0.52 to 0.60)	0.20	0.1
RAASi initiation	1187 / 12,274	9.7 (8.6 to 10.7)	0.00 (0.00 to 0.00)	0.86 (0.80 to 0.90)	0.0	0.0
No NSAID prescription	213,753 / 219,714	97.3 (97.1 to 97.4)	0.05 (0.04 to 0.06)	0.77 (0.75 to 0.79)	0.84	82.7

^a^ ICCs were derived from 3-level binomial mixed regression models adjusting for patient’s age, sex, and month count (1–51) and calendar month (1–12) during period of discharge (January 2017-March 2021), using the ‘estat icc’ post-estimation command in Stata

Serum creatinine was measured in 34.2% (95% CI 33.7–34.8) of episodes and blood pressure in 34.6% (95% CI 34.0-35.1) at 90 ± 30 days after discharge, and in 57.0% (95% CI 56.3–57.7) and 57.2% (95% CI 56.4–57.9) of episodes, respectively, at 365 ± 90 days. Checks of ACR occurred in only 4.2% (95% CI 4.0-4.4) of episodes at 90 ± 30 days, rising to 12.2% (95% CI 11.8–12.6) at 365 ± 90 days. Patients aged 60 or over, those with a primary care coding of AKI, and those with a comorbid condition, in particular diabetes, were more likely to have these assessments (Additional file 1: Figs. S5-S10; Tables S4-S6). Patients of Asian or Black ethnicity were also more likely than White patients to have measurements for ACR. Amongst patients with *de novo* CKD, only 17.1% (95% CI 15.5–18.8) had CKD recorded within 90 ± 30 days after discharge (Additional file 1: Fig. S11; Table S7).

Of patients with a guideline-recommended indication for RAASi and who were prescribed these drugs before admission, they were prescribed within 90 days after discharge in 70.6% (95% CI 69.3–71.8) of the episodes. Patients aged 60 and over, those with primary care coded AKI, CKD, and those who had outpatient contacts, were less likely than younger patients and those without primary care coded AKI, CKD, or outpatient contacts to be prescribed RAASi (Additional file 1: Fig. S12; Table S8). Investigation by period of discharge showed some evidence of improvements in prescribing over time. Amongst eligible patients not previously prescribed RAASi, only 9.7% (95% CI 8.6–10.7) received a prescription within 90 days after discharge. Older patients and those with CKD were again less likely than younger patients and those without CKD to have RAASi prescribed (Additional file 1: Fig. S13; Table S8). In 97.3% (95% CI 97.1–97.4) of episodes patients were not prescribed an NSAID within 90 days of discharge (Additional file 1: Fig. S14; Table S9). This means that these drugs were still being prescribed in 2.7% (95% CI 2.6–2.9) of cases, with younger patients and those having had major surgery being more likely to be prescribed these drugs.

### Variation between practices and reliability of the indicators

Table 3 shows the practice- and patient-level ICCs for the indicators. For AKI coding in primary care (practice-level ICC = 0.23), 23% of the variance in indicator adherence was due to between-practice differences. Conversely, there were only minor between-practice variations for RAASi initiation and most variation was due to differences between patients. The high levels of patient-level ICCs across all indicators also suggest there were more variations in adherence between different patients than between multiple episodes in a single patient.

**Fig. 2:**
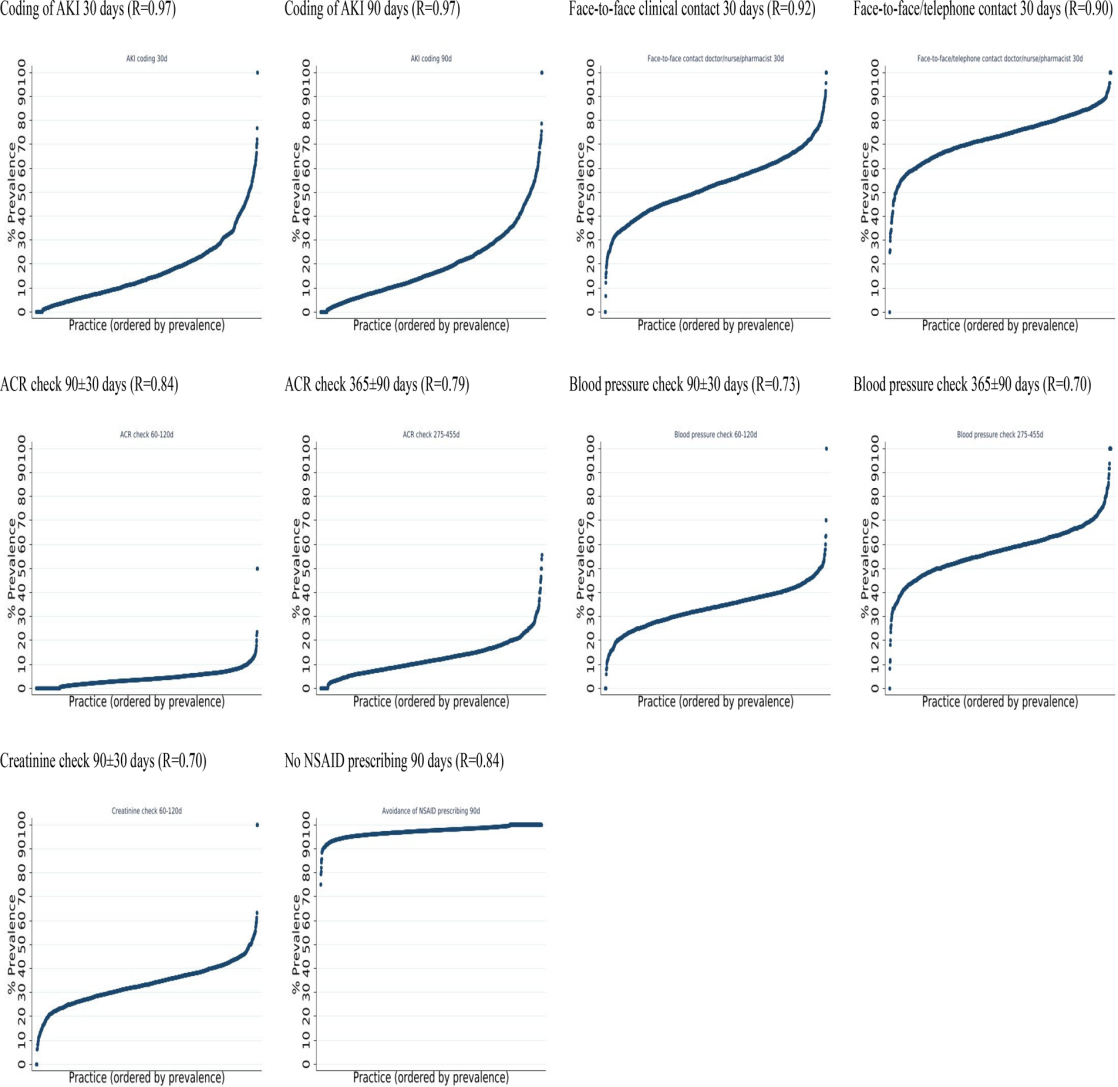
Adherence to post-AKI care indicators with reliability > = 0.7 by practice. Vertical axis shows percentage of AKI episodes meeting indicator criteria. Horizontal axis shows practice ordered by increasing percentage of episodes meeting indicator criteria. R value reported in brackets for each indicator is the reliability of a practice with median number of eligible patients and median number of AKI episodes

Table 3 also shows the reliability of each indicator, for a hypothetical practice with median numbers of patients and of AKI episodes (see Additional file 1: Table S10 for further details). Ten indicators have reliability at or above the recommended level of 0.7, with four having over 90% of practices above this value. Figure 2 shows, for each of these 10 indicators, the percentage of episodes meeting the criteria of the indicator by practice. Four indicators - creatinine measurements at 365 ± 90 days after discharge, coding of de novo CKD, and RAASi continuation and initiation – had reliability below 0.7 (Additional file 1: Fig. S15). The number of patients and AKI episodes eligible for inclusion for the latter three indicators were low due to lack of creatinine and ACR measurements in many patients, contributing to their low reliability scores.

For measurements of creatinine, ACR, and blood pressure indicators, we report the number of practices with 0% indicator adherence (Additional file 1: Table S11), and the number of practices in which there were 0, 1, 2 or more of these indicators with 0% adherence (Additional file 1: Table S12). For ACR measurements at 90 ± 30 days after discharge, 10.6% of the practices had 0% indicator adherence. Since checks for ACR occurred in only 4.2% of episodes at 90 ± 30 days after discharge (Table 3), these results imply that amongst the 89.4% of practices which had at least one record of ACR tests being conducted, there was very little between-practice variation in how commonly these tests were missed. This was also evidenced in the low practice-level ICC values for these indicators.

## Discussion

### Principal findings

This population-based study of 209,222 patients from 1470 general practices in England discharged after an inpatient AKI episode found that AKI was coded in primary care records for only a fifth of episodes. Furthermore, despite high levels of contact with primary care healthcare professionals, only a minority of patients received the recommended blood and urine monitoring to assess kidney health, with especially low rates of ACR assessments. Testing was more common amongst older patients, and in those with comorbid conditions. Over a quarter of the patients who were on RAASi prior to admission were not prescribed these medications within 90 days after discharge, and only 10% with an indication and without previous prescriptions had these medications newly initiated. Prescribing was less likely for older patients and (in conflict with current evidence) for those with AKI coding in primary care or with CKD. There was little practice variability in adherence across most indicators. Ten of the 14 indicators have reliability values at or above the recommended level.

### Comparison with previous findings

This is the first investigation of variations in post-discharge care of people with AKI in general practices across England according to recommended guidelines. Previous studies have demonstrated limited implementation of post-AKI care processes in primary care, though these studies were confined to local areas covering a limited range of care indicators [8, 36]. In England, the UK Renal Registry (UKRR) database has been established with 187 out of the 192 (97%) hospital laboratories across the country now providing AKI data [37]. The 2024 UKRR nationwide report for England showed that over 700,000 AKI warning stage test results (AKI e-alerts) were generated in 2024 from over 600,000 people, accounting for over 1% of the English population [37]. Just under 475,000 of the AKI episodes (67%) were hospitalised episodes, demonstrating the scale of the post-discharge implementation challenge. There was variation across hospital trusts in the extent to which AKI alerts were translated into a clinical diagnostic (N17) code in hospital, with increases in coding with AKI severity [37]. Evidence from other studies, both in and outside England, also indicates poor and variable documentation of AKI on discharge summaries [8, 10, 38–43]. Here our study complements this previous work by following what happens next after discharge among those with hospital-coded AKI and reports further variation in care among this subset of AKI with a clinically verified diagnosis.

In keeping with findings from this study, an audit of primary care records of discharged patients with AKI from 31 general practices in Bury in Greater Manchester, England, found that only 28% of AKI episodes were subsequently coded in primary care, although this rose to 50% following an intervention to improve post-discharge AKI care [8]. In a study of 45 general practices in Salford, Greater Manchester, the level of coding was lower at 10%, and only 3% of patients had their ACR measured within 3 months of AKI diagnosis [36]. Inadequate monitoring of kidney health status, especially for albuminuria, and undertreatment of those who could benefit from RAASi post-AKI have also been reported in Scotland, Canada and the US, suggesting that sub-optimal post-AKI care is common [44–46].

Results from our population-based cohort study taken in conjunction with findings from our qualitative research and the wider literature indicate that to date, despite policies, guidelines and standards highlighting the importance, arrangements for AKI follow-up care becomes lost in translation across care settings. Qualitative studies, including work linked directly to this study, consistently highlight the vulnerabilities being experienced by patients following an illness complicated by AKI, including challenges navigating care [47–50]. Our application of systems thinking also illuminates workforce pressures experienced by healthcare staff in secondary and primary care [48]. Implementation strategies to strengthen follow-up care may need better resourcing across multiple areas: early and ongoing patient engagement; coordination across care interfaces; and protocols to clarify delegated follow-up [48]. However, to date, a survey of AKI service provision across England indicates investment in acute management but limited resourcing of follow-up care after AKI [51].

Our analysis of outpatient contacts with General Surgery, Endocrinology and Diabetes, Cardiology, or Renal Medicine within 90-days of hospital discharge showed variable levels of diagnostic coding of an AKI, assessment of kidney health status, coding of CKD (when relevant) and RAASi initiation, but lower levels of RAASi continuation where indicated. However, we suggest cautious interpretation as it was not possible to determine the nature of these clinical encounters and the extent to which they focused on AKI-related care. In the most recent Kidney Disease: Improving Global Outcomes (KDIGO) AKI guidelines (available in draft form at time of writing) evidence was evaluated as “low certainty” that nephrology follow-up for patients with more severe AKI is associated with lower mortality and possibly lower recurrent AKI [41]. The KDIGO work group acknowledged a need to balance this approach against risks of treatment burden including long travel times, additional appointments and hospitalisation-related fatigue [41, 52–56]. Resourcing coordinated personalised follow-up according to an individual’s clinical and vulnerability risks is a priority, with trial evidence from Alberta, Canada to support a risk stratified approach in determining who will benefit from nephrology care [41, 48, 57]. In the NHS in England, general practice maintains responsibility for prescribing, highlighting the importance of primary care coding for AKI and CKD (where relevant) to enable effective care for people with complex health and care needs, both in terms of long-term conditions management and during future episodes of acute illness.

### Strengths and limitations

CPRD is broadly representative of the English population and includes all prescriptions issued in participating practices, while HES covered all Integrated Care Boards [20]. Although health care systems differ between countries, our study implications may be relevant beyond England.

Our cohort was defined using ICD-10 coding for AKI, which ensured we only considered follow-up care of those with a clinical diagnosis in hospital. However, in doing so, it limited our ability to examine patients according to staging and AKI severity and etiology. ICD10 codes do not specify the underlying causes of AKI with the exception of O90.4 which represents postpartum acute renal failure, but this only accounted for less than 0.2% of AKI coding amongst our patients. We were also only able to include patients discharged from hospitals up to March 2021, as these were the most recent data available at the time of conducting this study (2024). Organisation of post-AKI care might have changed after the Covid-19 pandemic, and future research can investigate this. In addition, we do not know the context of consultation at the general practice and were only able to examine coded clinical events. In CPRD, as in other routinely collected electronic health records, the absence of a clinical coding is interpreted as an absence of a diagnosis, test, or action [58]. For example, any tests conducted in hospitals and not reported to general practices would not be included in our study. We also did not have access to discharge summaries or the free text entries in the primary care records, where some follow-up care might have been recorded. Therefore, coding errors, missing or incomplete information could have led to an underestimation of true adherence to some indicators.

While the large cohort size was one of the key strengths of this study, associations were more likely to be of statistical significance with such a large population, even when the effect sizes were small. Given the large number of investigations being conducted on variations in indicators adherence according to demographics and clinical characteristics, there is also the potential issue with multiple comparisons leading to false positive associations (Type 1 error). The ORs should therefore be interpreted with caution, with greater emphasis being placed on the effect sizes and their clinical significance, rather than statistical significance. Our analyses were also primarily descriptive and quality improvement focused, and not intended to be formal hypothesis testing for any of the subgroup effects.

### Interpretations and implications for practice and policy

A key contribution of this study is the creation of indicators based on guideline recommendations of post-AKI care or operationalisation of existing indicators in a large English population, with almost all 14 indicators revealing shortfalls in post-AKI care. Both national and international guidelines recommend checking creatinine, eGFR, and ACR at 3 months after an episode of AKI, to monitor for the development or worsening of CKD [5, 9, 14–18, 25−27]. Nonrecovery of kidney function and albuminuria at 3 months are important predictors of long-term kidney health [59, 60]. Moreover, assessing eGFR and albuminuria post-discharge helps identify opportunities for prescribing medications that modify subsequent cardio-renal risk, particularly important with the expansion of effective therapies for CKD, such as RAASi and sodium-glucose cotransporter 2 inhibitors [61]. We found that patients with a primary care coding of AKI were more likely to have blood and urine testing performed after discharge. However, there was substantial under-coding across all subgroups. An important aspect of safe transition of care from hospital to primary care is the sharing of relevant information in discharge summaries, and better communication in these summaries is associated with improved outcomes post-AKI [42]. We did not have access to discharge summaries of our study cohort. However, despite initiatives to improve communication about AKI on discharge summaries, audits of acute hospital trusts across England and Wales have shown that 20% of discharge summaries did not mention AKI, and a third omitted information about medicine changes, recommended blood tests, or follow-up of unresolved AKI [10, 42].

Patients aged 60 or over, and those with comorbidity, especially diabetes, were more likely to have the recommended blood and urine checks in our study, suggesting that these measurements were already integrated within other long-term condition pathways as opposed to distinct post-AKI assessments. Similarly, a higher percentage of patients of Black and Asian ethnicity with ACR measurements may relate to the higher prevalence of diabetes in these groups, in keeping with wider evidence demonstrating greater levels of incentivised albuminuria testing for people with diabetes in primary care [62, 63]. Future studies would benefit from analysis that takes into account additional factors to which we were unable to explore, including patient-level such as patient awareness, health literacy, cultural beliefs, or organisational, for example, multidisciplinary care within primary care settings [64].

Recognising that AKI and CKD are related entities across a continuum of health states, international KDIGO guidelines emphasise the importance of an evaluation for development or progression of CKD 90 days post-discharge following AKI, including assessment for albuminuria [9, 18, 25, 41, 59, 60, 65, 66]. Our study highlights a gap in current care, with substantial scope for improvement. Only 17% of those who developed de novo CKD were coded in the records, and diagnosis might have been missed in some patients because of the lack of ACR measurements. Primary care health professionals need to address competing priorities within constrained time and resources and attention to kidney health may be crowded out. Nevertheless, this represents a missed opportunity to improve patient outcomes because proteinuria testing, which is relatively straightforward to perform in primary care, is a critical component of recovery/non-recovery assessment. Another major concern is that little post-AKI clinical activity occurred in those who were younger or without morbidity. Given a longer expected life course, there may be greater opportunities for benefit from long-term cardio-renal risk reduction in these groups that currently are being missed.

Amongst patients with an indication for RAASi, older patients, those with CKD, or a primary care coded AKI, were less likely to receive these medications. It is possible that these patients were perceived to be at increased risk of recurrent AKI and that primary care practitioners were less comfortable prescribing RAASi even when a guideline indication exists. This may be over-cautious, as observational data show that RAASi use after AKI discharge is associated with improved survival and fewer cardiovascular events without increased risks of recurrent AKI [28, 29]. The lack of information about medication changes and the need for medication reviews in discharge summaries may have further contributed to under-prescribing of RAASi [42]. Furthermore, the poor coding of AKI and the higher prescribing of NSAIDs amongst patients who sustained AKI during admissions for major surgery suggest that the lack of a kidney lens seems most apparent in the surgical setting.

Values of ICCs suggested that variation of indicator adherence was predominantly at the patient level, with less variation attributable to differences between general practices. This information may be used to prioritise subpopulations for improvement work, although the widespread low adherence suggests that all general practices may benefit from improvement support. Our study additionally demonstrates that some indicators have low reliability for a typically sized general practice. While 10 indicators were found to have high reliability, four indicators - creatinine measurements at 365 ± 90 days after discharge, coding of de novo CKD, and RAASi continuation and initiation - had reliability values that were below the ‘acceptable’ level which suggests that they are unsuitable for practice level profiling or benchmarking. The two indicators for RAASi prescribing were particularly unreliable. ACR testing is a prerequisite to RAASi prescription (in our study methodology). It is also more likely to occur either in the context of a pre-existing indication (diabetes) or coded AKI. Coding AKI in the primary care record therefore represents an important gateway task. Given high levels of patient contact with general practices soon after discharge, improvement in AKI care may not need more general practice appointments, but rather better integration of kidney assessments within existing care. This is important, as the resource implications are potentially less. Younger patients and those without existing comorbidities should also be monitored for development of CKD.

## Conclusions

Despite AKI being a major patient safety priority with over a decade of initiatives to improve AKI care across the NHS, adherence to guideline-recommended post-AKI care in primary care in England remains poor overall. The substantial under coding of AKI across all subgroups of patients suggests a lack of specific consideration of AKI at handover from hospitals to primary care. The variable delivery of post-discharge care was evident despite relatively high levels of contact with primary care healthcare professionals. Rates of measuring albuminuria were particularly low, despite its strong association with subsequent kidney and cardiovascular events. Shortcomings in care after patients transition from hospital back to the community represent missed opportunities to address these risks, particularly in younger patients and those without existing co-morbidities. Concerted implementation of kidney health strategies spanning primary and secondary care, including clear discharge arrangements and case-specific guidance on discharge summaries, are needed to improve post-AKI care and outcomes.

## Supplementary Information

Below is the link to the electronic supplementary material.

Supplementary Material 1: Additional Information S1-S3, Tables S1-S12, Figures S1-S15, and clinical, ICD10, and OPCS codes used.

## Data Availability

The first author (PLHM) has full access to all the data and all authors have full access to the statistical reports and tables in the study. The study is based on data from the Clinical Practice Research Datalink obtained under licence from the UK Medicines and Healthcare products Regulatory Agency (MHRA). Hospital Episode Statistics and Office of National Statistics mortality data are subject to Crown copyright (2025) protection, re-used with the permission of The Health & Social Care Information Centre, all rights reserved. Electronic health records are, by definition, considered sensitive data in the UK by the Data Protection Act and cannot be shared via public deposition because of information governance restriction in place to protect patient confidentiality. Access to CPRD data is subject to protocol approval via CPRD’s Research Data Governance Process. For more information, see https://www.cprd.com/access-data.

## Abbreviations

ACE inhibitors: Angiotensin-converting enzyme inhibitors
ACR: Albumin/creatinine ratio
AKI: Acute kidney injury
APC: Admitted Patient Care
ARBs: Angiotensin receptor blockers
CKD: Chronic kidney disease
CPRD: Clinical Practice Research Datalink
eGFR: Estimated glomerular filtration rate
HES: Hospital Episode Statistics
ICC: Intraclass correlation coefficient
ICD-10: International Classification of Disease 10th revision
IMD: Index of Multiple Deprivation
KDIGO: Kidney Disease: Improving Global Outcomes
NHS: National Health Service
NSAIDs: Non-steroidal anti-inflammatory drugs
OPCS: Office of Population Censuses and Surveys
ORs: Odds ratios
RAASi: Renin-angiotensin-aldosterone system inhibitors
SNOMED: Systematized Nomenclature of Medicine
UKRR: UK Renal Registry
