# Supplementary material for: Examining variations in adherence to guideline-recommended post-acute kidney injury care in primary care in England: a population-based cohort study
Source: BMC Med. 2026 Aug 12;24:406. doi: 10.1186/s12916-026-05042-0 (PMC13464333; doi:10.1186/s12916-026-05042-0)
Supplement: Supplementary file 1 [file 12916_2026_5042_MOESM1_ESM.docx]

**Additional file 1**

[**Additional Information S1 – Destinations on discharge included and excluded in the delineation of the study cohort**	3](#_Toc233205983)

[**Additional Information S2 – Intraclass correlation coefficients**	4](#_Toc233205984)

[**Additional Information S3 – Reliability**	5](#_Toc233205985)

[**Table S1. Definitions of the post-AKI care indicators and references to guidance**	6](#_Toc233205986)

[**Table S2. Coding of AKI in primary care record within 30 and 90 days after discharge, by demographic and clinical characteristics**	9](#_Toc233205987)

[**Table S3. Face-to-face/telephone contact with a doctor, nurse, or pharmacist from the general practice within 30 days after discharge, by demographic and clinical characteristics**	11](#_Toc233205988)

[**Table S4. Creatinine measurement at 90±30 days and 365±90 days after discharge, by demographic and clinical characteristics**	13](#_Toc233205989)

[**Table S5. ACR measurement at 90±30 days and 365±90 days after discharge, by demographic and clinical characteristics**	15](#_Toc233205990)

[**Table S6. Blood pressure measurement at 90±30 days and 365±90 days after discharge, by demographic and clinical characteristics**	17](#_Toc233205991)

[**Table S7. Coding of *de novo* CKD at 90±30 days after discharge, by demographic and clinical characteristics**	19](#_Toc233205992)

[**Table S8. RAASi continuation and initiation within 90 days after discharge, by demographic and clinical characteristics**	21](#_Toc233205993)

[**Table S9. Avoidance of prescription of NSAID within 90 days after discharge, by demographic and clinical characteristics**	23](#_Toc233205994)

[**Table S10. Reliability of post-AKI care indicators**	25](#_Toc233205995)

[**Table S11. Number of practices with 0% adherence to indicators related to creatinine, ACR, and blood pressure measurements**	27](#_Toc233205996)

[**Table S12. Number of practices for which there was 0% adherence for 0, 1, 2 or more blood and urine measurement indicators (creatinine, ACR, or blood pressure)**	28](#_Toc233205997)

[**Figure S1. Delineation of study cohort of patients**	29](#_Toc233205998)

[**Figure S2. Odds ratios (95% CI) of AKI episodes recorded in primary care records within 90 days after discharge, by demographics, socioeconomics, and clinical characteristics**	30](#_Toc233205999)

[**Figure S3. Odds ratios (95% CI) of face-to-face contact with a doctor, nurse, or pharmacist at the general practice within 30 days after discharge, by demographics, socioeconomics, and clinical characteristics**	31](#_Toc233206000)

[**Figure S4. Odds ratios (95% CI) of face-to-face or telephone contact with a doctor, nurse, or pharmacist at the general practice within 30 days after discharge, by demographics, socioeconomics, and clinical characteristics**	32](#_Toc233206001)

[**Figure S5. Odds ratios (95% CI) of creatinine measurement at 90±30 days after discharge, by demographics, socioeconomics, and clinical characteristics**	33](#_Toc233206002)

[**Figure S6. Odds ratios (95% CI) of creatinine measurement at 365±90 days after discharge, by demographics, socioeconomics, and clinical characteristics**	34](#_Toc233206003)

[**Figure S7. Odds ratios (95% CI) of ACR measurement at 90±30 days after discharge, by demographics, socioeconomics, and clinical characteristics**	35](#_Toc233206004)

[**Figure S8. Odds ratios (95% CI) of ACR measurement at 365±90 days after discharge, by demographics, socioeconomics, and clinical characteristics**	36](#_Toc233206005)

[**Figure S9. Odds ratios (95% CI) of blood pressure measurement at 90±30 days after discharge, by demographics, socioeconomics, and clinical characteristics**	37](#_Toc233206006)

[**Figure S10. Odds ratios (95% CI) of blood pressure measurement at 365±90 days after discharge, by demographics, socioeconomics, and clinical characteristics**	38](#_Toc233206007)

[**Figure S11. Odds ratios (95% CI) of coding of *de novo* CKD in primary care records at 90±30 days after discharge, by demographics, socioeconomics, and clinical characteristics**	39](#_Toc233206008)

[**Figure S12. Odds ratios (95% CI) of RAASi continuation within 90 days after discharge, by demographics, socioeconomics, and clinical characteristics**	40](#_Toc233206009)

[**Figure S13. Odds ratios (95% CI) of RAASi initiation within 90 days after discharge, by demographics, socioeconomics, and clinical characteristics**	41](#_Toc233206010)

[**Figure S14. Odds ratios (95% CI) of avoidance of prescribing of NSAID within 90 days after discharge, by demographics, socioeconomics, and clinical characteristics**	42](#_Toc233206011)

[**Figure S15. Adherence to post-AKI care indicators with reliability <0.7 by practice**	43](#_Toc233206012)

[**Clinical codes used to identify AKI in CPRD Aurum**	44](#_Toc233206013)

[**Clinical codes used to identify serum creatinine measurements in CPRD Aurum**	48](#_Toc233206014)

[**Clinical codes used to identify ACR measurements in CPRD Aurum**	49](#_Toc233206015)

[**Clinical codes used to identify blood pressure measurements in CPRD Aurum**	50](#_Toc233206016)

[**Clinical codes used to identify hypertension in CPRD Aurum**	56](#_Toc233206017)

[**ICD10 codes used to identify hypertension in HES Admitted Patient Care data**	61](#_Toc233206018)

[**Clinical codes used to identify CKD in CPRD Aurum**	62](#_Toc233206019)

[**ICD10 codes used to identify CKD in HES Admitted Patient Care data**	70](#_Toc233206020)

[**Clinical codes used to identify diabetes in CPRD Aurum**	74](#_Toc233206021)

[**ICD10 codes used to identify diabetes in HES Admitted Patient Care data**	86](#_Toc233206022)

[**Clinical codes used to identify heart failure in CPRD Aurum**	88](#_Toc233206023)

[**ICD10 codes used to identify heart failure in HES Admitted Patient Care data**	91](#_Toc233206024)

[**Clinical codes used to identify myocardial infarction in CPRD Aurum**	92](#_Toc233206025)

[**ICD10 codes used to identify myocardial infarction in HES Admitted Patient Care data**	97](#_Toc233206026)

[**ICD10 codes used to identify sepsis in HES Admitted Patient Care data**	98](#_Toc233206027)

[**Clinical codes used to identify kidney transplant in CPRD Aurum**	100](#_Toc233206028)

[**ICD10 codes used to identify kidney transplant in HES Admitted Patient Care data**	106](#_Toc233206029)

[**OPCS codes used to identify kidney transplant in HES Admitted Patient Care data**	107](#_Toc233206030)

[**Clinical codes used to identify chronic dialysis in CPRD Aurum**	108](#_Toc233206031)

[**ICD10 codes used to identify chronic dialysis in HES Admitted Patient Care data**	114](#_Toc233206032)

[**OPCS codes used to identify chronic dialysis in HES Admitted Patient Care data**	115](#_Toc233206033)

[**OPCS codes used to identify major surgery in HES Admitted Patient Care data**	116](#_Toc233206034)

[**Product codes used to identify RAASi prescribing in CPRD Aurum**	119](#_Toc233206035)

[**Product codes used to identify NSAID prescribing in CPRD Aurum**	129](#_Toc233206036)

## **Additional Information S1 – Destinations on discharge included and excluded in the delineation of the study cohort**

Information on destinations on discharge was available from the ‘DISDEST’ field in the HES Admitted Patient Care dataset. The numbers below are the values of the field. Only destinations that were applicable to our extracted data were shown.

Destinations on discharge included

- 19 = The usual place of residence, including no fixed abode
- 29 = Temporary place of residence when usually resident elsewhere, for example, hotels and residential educational establishments
- 30 = Repatriation from high security psychiatric hospital
- 37 = Penal establishment - court
- 38 = Penal establishment - police station
- 54 = NHS run care home
- 65 = Local Authority residential accommodation i.e. where care is provided
- 66 = Local Authority foster care
- 85 = Non-NHS (other than Local Authority) run care home
- 88 = Hospice
- 99 = Destination of discharge not known

Destinations on discharge excluded

- 48 = High security psychiatric hospital, Scotland
- 49 = NHS other hospital provider - high security psychiatric
- 50 = NHS other hospital provider - medium secure unit
- 51 = NHS other hospital provider - ward for general patients or the younger physically disabled
- 52 = NHS other hospital provider - ward for maternity patients or neonates
- 53 = NHS other hospital provider - ward for patients who are mentally ill or have learning disabilities
- 79 = Patient died
- 84 = Independent Sector Healthcare Provider run hospital - medium secure unit
- 87 = Independent Sector Healthcare Provider run hospital - excluding medium secure unit
- 98 = Not applicable - Hospital Provider Spell not finished at episode end (i.e. not discharged) or current episode unfinished

## **Additional Information S2 – Intraclass correlation coefficients**

The intraclass correlation coefficient (ICC) is a measure of the degree of variability (or correlations) between ‘units’ in a multilevel model.^30,31^ It is expressed as the amount of variance between the units as a proportion of the total variance in the model. For a two-level random intercept binomial mixed regression model with, say, patients (level 1) nested within practices (level 2), the ICC is:^31^

ρ = σ_2_^2^ / (γ + σ_2_^2^)

where:

σ_2_^2^ = variance between practices

γ = error variance at the patient-level which is π^2^/3 for a binomial mixed regression model

ICC ranges between 0 and 1. In the example of a 2-level model with patients nested within practices, a higher value of the ICC means larger variations (i.e. smaller correlations) in the outcome between practices.

In our study, because patients could have more than one AKI episode, we have a 3-level data structure where AKI episodes are nested within patients and within practices. There are, therefore, two types of ICC: practice level (level 3) and patient level (level 2). The practice-level ICC is defined as: ^31^

ρ^(3)^ = σ_3_^2^ / (γ + σ_2_^2^+ σ_3_^2^)

The patient-level ICC is defined as: ^31^

ρ^(2)^ = (σ_2_^2^ + σ_3_^2^) / (γ + σ_2_^2^+ σ_3_^2^)

where:

σ_3_^2^ = variance between practices

σ_2_^2^ = variance between patients within practices

γ = error variance at the episode level which is π^2^/3 for a binomial mixed regression model

The practice-level ICC is a measure of the variation between practices in adherence to an indicator. If the practice-level ICC is high, there is large variation between practices in adherence to the indicator, even for patients with similar characteristics. If the values are low, it means there is little variation between practices in adherence to the indicator; instead, much of the variation is due to differences between patients.

The patient-level ICC is a measure of the variation between patients in adherence to an indicator. Higher patient-level ICCs indicate greater variations in indicator adherence at the patient level and relatively consistent adherence within individuals across episodes, i.e. greater between-patient heterogeneity such as in patient characteristics but smaller differences in patterns of care across episodes for a patient.

ICCs were derived from 3-level binomial mixed regression models adjusting for patient’s age, sex, month count (1-51) and calendar month (1-12) during period of discharge (January 2017-March 2021), using the ‘estat icc’ post-estimation command.^31^

## **Additional Information S3 – Reliability**

The reliability of each indicator quantifies how much of the observed variation is due to practice differences, compared with within-subject variation driven by differences in patient characteristics.^32-34^ We used the formula for a 3-level model in Jeon et al (2009),^35^ applied to our study as:

$$reliability= \frac{\sigma^{2}(practice)}{\sigma^{2}\left( practice \right)+\frac{\sigma^{2}\left( patient:practice \right)}{n_{k}}+\frac{\sigma^{2}\left( AKI episodes:patients:practice \right)}{n_{k}n_{jk}}}$$

where:

σ^2^ *(practice)* = variance between practices

σ^2^ *(patient:practice)* = variance between patients within practices

σ^2^ *(AKI episodes:patient:practice)* = variance of AKI episodes amongst patients and practices, which is π^2^/3 for a binomial mixed regression model

n_k_ = number of patients in practice k who were eligible for inclusion in the analysis of the indicator

n_jk_ = number of AKI episodes for patient j in practice k

Variances were obtained from 3-level binomial mixed regression models adjusting for patient’s age, sex, and month count (1-51) and calendar month (1-12) during period of discharge (January 2017-March 2021). Reliability ranges between 0 and 1, with a value of 0.7 being generally considered as acceptable. The larger the variance between practices (i.e. σ^2^ *(practice)*), and the larger the number of patients (n_k_) and AKI episodes (n_jk_) in a practice, the higher the reliability. An indicator may therefore be more reliable for larger than for smaller practices.

Because each practice has its own reliability value for each indicator, to summarise the reliability for each indicator, we reported the reliability for a hypothetical practice with median number of eligible patients and median number of AKI episodes.^32-34^ We also reported the percentage of practices with reliability greater than 0.7.

## **Table S1. Definitions of the post-AKI care indicators and references to guidance**

|  | **Indicator** | **Numerator** | **Denominator** | **References to guidance** |
| --- | --- | --- | --- | --- |
|  | ***Recording of AKI in primary care*** | | | |
| 1 | Coding of AKI in the primary care record within 30 days after discharge | Number of AKI episodes with a coding of AKI in the primary care record within 30 days after discharge | Number of AKI episodes for which the patient had not been censored within 30 days after discharge | 12,14,15 |
| 2 | Coding of AKI in the primary care record within 90 days after discharge | Number of AKI episodes with a coding of AKI in the primary care record within 90 days after discharge | Number of AKI episodes for which the patient had not been censored within 90 days after discharge |  |
|  |  | | | |
| 3 | Face-to-face contact with a doctor, nurse, or pharmacist at the general practice within 30 days after discharge | Number of AKI episodes for which the patient had a face-to-face contact with a doctor, nurse, or pharmacist at the general practice within 30 days after discharge | Number of AKI episodes for which the patient had not been censored within 30 days after discharge | 11,14,15 |
| 4 | Face-to-face or telephone contact with a doctor, nurse, or pharmacist at the general practice within 30 days after discharge | Number of AKI episodes for which the patient had a face-to-face or telephone contact with a doctor, nurse, or pharmacist at the general practice within 30 days after discharge | Number of AKI episodes for which the patient had not been censored within 30 days after discharge |  |
|  | ***Monitoring of kidney health status and blood pressure*** | | | |
| 5 | Serum creatinine measurement at 90±30 days after discharge | Number of AKI episodes for which the patient had a creatinine measurement at 90±30 days after discharge | Number of AKI episodes for which the patient had not been censored within 120 days after discharge | 5,9,14-18,25 |
| 6 | Serum creatinine measurement at 365±90 days after discharge | Number of AKI episodes for which the patient had a creatinine measurement at 365±90 days after discharge | Number of AKI episodes for which the patient had not been censored within 455 days after discharge |  |
| 7 | Albumin/creatinine ratio (ACR) measurement at 90±30 days after discharge | Number of AKI episodes for which the patient had a ACR measurement at 90±30 days after discharge | Number of AKI episodes for which the patient had not been censored within 120 days after discharge | 5,9,14-16, 18,25-27 |
| 8 | ACR measurement at 365±90 days after discharge | Number of AKI episodes for which the patient had a ACR measurement at 365±90 days after discharge | Number of AKI episodes for which the patient had not been censored within 455 days after discharge |  |
| 9 | Blood pressure measurement at 90±30 days after discharge | Number of AKI episodes for which the patient had a blood pressure measurement at 90±30 days after discharge | Number of AKI episodes for which the patient had not been censored within 120 days after discharge | 9,16,18 |
| 10 | Blood pressure measurement at 365±90 days after discharge | Number of AKI episodes for which the patient had a blood pressure measurement at 365±90 days after discharge | Number of AKI episodes for which the patient had not been censored within 455 days after discharge |  |
| 11 | Coding of *de novo* CKD in the primary care record at 90±30 days after discharge | Number of AKI episodes for which the patient had a coding of CKD in the primary care record at 90±30 days after discharge | Number of AKI episodes for which the patient:   - had not been censored within 120 days after discharge, and - eGFR <60 mL/min/1.73m^2^ at 90±30 days after discharge, and - there was no previous CKD code in the patient’s record. | 9,16,18 |
|  | ***Guideline indicated prescribing*** | | |  |
| 12 | Renin–angiotensin–aldosterone system inhibitors (RAASi) continuation | Number of AKI episodes for which the patients were prescribed RAASi within 90 days after discharge | Number of AKI episodes for which the patient:   - had not been censored within 90 days after discharge, and - had an indication for RAASi, with indication defined as either:   - a history of diabetes and ACR>=3, or  - a history of hypertension and ACR>=30, or  - ACR>= 70, and   - had at least one prescription for RAASi in the 90 days before admission   A history of diabetes and hypertension was defined as having a code for these conditions in the patient’s record at any time up to (and including) the date of discharge. Values of ACR recorded nearest to, and within 1095 days before discharge (including the date of discharge) were used. | 13-15,28,29 |
| 13 | RAASi initiation | Number of AKI episodes for which the patients were prescribed RAASi within 90 days after discharge | Number of AKI episodes for which the patient:   - had not been censored within 90 days after discharge, and - had an indication for RAASi, with indication defined as for RAASi continuation, and - did not have a prescription for RAASi in the 90 days before admission | 13-15,28,29 |
| 14 | Avoidance of prescription of nonsteroidal anti-inflammatory drugs (NSAIDs) within 90 days after discharge | Number of AKI episodes for which the patients were not prescribed NSAID within 90 days after discharge | Number of AKI episodes for which the patient had not been censored within 90 days after discharge | 13,14 |

## **Table S2. Coding of AKI in primary care record within 30 and 90 days after discharge, by demographic and clinical characteristics**

|  | **Within 30 days after discharge** | | | **Within 90 days after discharge** | | |
| --- | --- | --- | --- | --- | --- | --- |
|  | Number of AKI episodes meeting indicator criteria / Number of AKI episodes eligible for inclusion | Percentage of AKI episodes meeting indicator criteria (95% CI) ^a^ | Odds ratio (OR) ^b^ | Number of AKI episodes meeting indicator criteria / Number of AKI episodes eligible for inclusion | Percentage of AKI episodes meeting indicator criteria (95% CI) ^a^ | Odds ratio (OR) ^b^ |
|  |  |  |  |  |  |  |
| Overall | 47,951 / 245,947 | 19.5 (18.5 to 20.5) | N/A | 48,348 / 219,714 | 22.0 (21.0 to 23.1) | N/A |
|  |  |  |  |  |  |  |
| Age |  |  |  |  |  |  |
| Less than 60 years | 7707 / 42,855 | 18.0 (16.9 to 19.1) | ref | 8273 / 40,322 | 20.5 (19.2 to 21.7) | ref |
| 60-79 years | 20,513 / 100,296 | 20.5 (19.4 to 21.6) | 1.24 (1.20 to 1.29) | 21,059 / 91,370 | 23.0 (21.8 to 24.2) | 1.29 (1.24 to 1.35) |
| 80 years or over | 19,731 / 102,796 | 19.2 (18.1 to 20.2) | 1.15 (1.11 to 1.20) | 19,016 / 88,022 | 21.6 (20.5 to 22.7) | 1.19 (1.14 to 1.25) |
|  |  |  |  |  |  |  |
| Sex |  |  |  |  |  |  |
| Female | 22,682 / 115,909 | 19.6 (18.5 to 20.7) | ref | 22,842 / 103,255 | 22.1 (20.9 to 23.2) | ref |
| Male | 25,269 / 130,038 | 19.4 (18.4 to 20.4) | 1.04 (1.01 to 1.06) | 25,506 / 116,459 | 21.9 (20.9 to 23.0) | 1.04 (1.01 to 1.08) |
|  |  |  |  |  |  |  |
| Ethnicity |  |  |  |  |  |  |
| White | 44,235 / 221,128 | 20.0 (19.0 to 21.1) | ref | 44,434 / 196,893 | 22.6 (21.4 to 23.7) | ref |
| Asian | 1786 / 12,306 | 14.5 (12.9 to 16.1) | 0.94 (0.87 to 1.01) | 1866 / 11,360 | 16.4 (14.8 to 18.2) | 0.91 (0.84 to 1.00) |
| Black | 1314 / 8873 | 14.8 (13.0 to 16.8) | 1.03 (0.95 to 1.13) | 1423 / 8134 | 17.5 (15.4 to 19.8) | 1.07 (0.97 to 1.18) |
| Other | 239 / 1579 | 15.1 (11.8 to 18.9) | 0.86 (0.72 to 1.03) | 243 / 1457 | 16.7 (12.8 to 20.8) | 0.78 (0.63 to 0.96) |
| Unknown | 377 / 2061 | 18.3 (14.9 to 21.8) | 0.97 (0.83 to 1.13) | 382 / 1870 | 20.4 (16.8 to 24.1) | 0.95 (0.79 to 1.14) |
|  |  |  |  |  |  |  |
| Patient-level IMD quintile |  |  |  |  |  |  |
| 1 (least deprived) | 8173 / 43,429 | 18.8 (17.3 to 20.5) | ref | 8181 / 38,570 | 21.2 (19.5 to 22.9) | ref |
| 2 | 9124 / 47,122 | 19.4 (18.1 to 20.7) | 1.03 (0.98 to 1.08) | 9097 / 42,042 | 21.6 (20.3 to 23.0) | 1.01 (0.96 to 1.07) |
| 3 | 8981 / 47,126 | 19.1 (17.9 to 20.4) | 1.06 (1.01 to 1.11) | 9029 / 42,032 | 21.5 (20.1 to 22.9) | 1.06 (1.00 to 1.13) |
| 4 | 9793 / 51,086 | 19.2 (18.0 to 20.4) | 1.07 (1.01 to 1.12) | 9979 / 45,799 | 21.8 (20.4 to 23.2) | 1.10 (1.03 to 1.17) |
| 5 (most deprived) | 11,835 / 56,189 | 21.1 (19.8 to 22.5) | 1.08 (1.02 to 1.14) | 12,016 / 50,359 | 23.9 (22.3 to 25.5) | 1.11 (1.04 to 1.19) |
| Unknown | 45 / 995 | 4.5 (0.0 to 78.5) | 0.21 (0.14 to 0.32) | 46 / 912 | 5.0 (0.0 to 87.1) | 0.10 (0.06 to 0.16) |
|  |  |  |  |  |  |  |
| CKD ^c^ |  |  |  |  |  |  |
| No | 21,371 / 114,314 | 18.7 (17.7 to 19.8) | ref | 21,684 / 104,313 | 20.8 (19.7 to 21.9) | ref |
| Yes | 26,580 / 131,633 | 20.2 (19.2 to 21.2) | 1.12 (1.09 to 1.15) | 26,664 / 115,401 | 23.1 (22.1 to 24.2) | 1.19 (1.15 to 1.23) |
|  |  |  |  |  |  |  |
| Diabetes ^c^ |  |  |  |  |  |  |
| No | 29,150 / 153,057 | 19.0 (18.0 to 20.1) | ref | 29,238 / 136,509 | 21.4 (20.4 to 22.5) | ref |
| Yes | 18,801 / 92,890 | 20.2 (19.2 to 21.3) | 1.16 (1.13 to 1.19) | 19,110 / 83,205 | 23.0 (21.9 to 24.1) | 1.21 (1.17 to 1.25) |
|  |  |  |  |  |  |  |
| Heart failure ^c^ |  |  |  |  |  |  |
| No | 32,044 / 166,768 | 19.2 (18.2 to 20.3) | ref | 32,546 / 150,972 | 21.6 (20.5 to 22.7) | ref |
| Yes | 15,907 / 79,179 | 20.1 (19.0 to 21.2) | 1.03 (1.00 to 1.06) | 15,802 / 68,742 | 23.0 (21.8 to 24.2) | 1.07 (1.03 to 1.11) |
|  |  |  |  |  |  |  |
| Myocardial infarction ^c^ |  |  |  |  |  |  |
| No | 37,590 / 194,268 | 19.3 (18.3 to 20.3) | ref | 38,007 / 174,079 | 21.8 (20.7 to 22.9) | ref |
| Yes | 10,361 / 51,679 | 20.0 (18.8 to 21.3) | 1.01 (0.98 to 1.05) | 10,341 / 45,635 | 22.7 (21.3 to 23.9) | 1.01 (0.97 to 1.05) |
|  |  |  |  |  |  |  |
| Major surgery during AKI episode |  |  |  |  |  |  |
| No | 46,658 / 235,682 | 19.8 (18.8 to 20.9) | ref | 46,871 / 209,897 | 22.3 (21.4 to 23.4) | ref |
| Yes | 1293 / 10,265 | 12.6 (11.1 to 14.2) | 0.47 (0.44 to 0.50) | 1477 / 9817 | 15.0 (13.6 to 16.6) | 0.46 (0.43 to 0.50) |
|  |  |  |  |  |  |  |
| Sepsis during AKI episode |  |  |  |  |  |  |
| No | 40,664 / 208,598 | 19.5 (18.5 to 20.5) | ref | 41,040 / 186,646 | 22.0 (20.9 to 23.0) | ref |
| Yes | 7287 / 37,349 | 19.5 (18.3 to 20.6) | 0.98 (0.95 to 1.02) | 7308 / 33,068 | 22.1 (20.8 to 23.4) | 0.99 (0.95 to 1.03) |
|  |  |  |  |  |  |  |
| Primary care coded AKI within 30 days of discharge |  |  |  |  |  |  |
| No | N/A | N/A | N/A | N/A | N/A | N/A |
| Yes | N/A | N/A | N/A | N/A | N/A | N/A |
|  |  |  |  |  |  |  |
| Face-to-face contact with a doctor, nurse, or pharmacist from the general practice within 30 days after discharge | |  |  |  |  |  |
| No | 22,566 / 119,019 | 19.0 (17.9 to 20.1) | ref | 22,601 / 105,226 | 21.5 (20.2 to 22.6) | ref |
| Yes | 25,385 / 126,928 | 20.0 (19.0 to 21.0) | 1.25 (1.22 to 1.28) | 25,747 / 114,488 | 22.5 (21.4 to 23.5) | 1.26 (1.22 to 1.30) |
|  |  |  |  |  |  |  |
| Outpatient contact with General Surgery, Endocrinology and Diabetes, Cardiology, or Renal Medicine within 30 days after discharge ^d^ | |  |  |  |  |  |
| No | 36,576 / 191,378 | 19.1 (18.1 to 20.1) | ref | 36,732 / 170,520 | 21.5 (20.5 to 22.6) | ref |
| Yes | 5224 / 25,651 | 20.4 (19.0 to 21.7) | 1.10 (1.06 to 1.15) | 5537 / 23,503 | 23.6 (21.9 to 25.1) | 1.15 (1.10 to 1.21) |
|  |  |  |  |  |  |  |
| Outpatient contact with General Surgery, Endocrinology and Diabetes, Cardiology, or Renal Medicine within 90 days after discharge ^d^ | |  |  |  |  |  |
| No | 29,366 / 155,670 | 18.9 (17.9 to 19.9) | ref | 29,045 / 137,100 | 21.2 (20.2 to 22.2) | ref |
| Yes | 10,780 / 53,871 | 20.0 (18.8 to 21.2) | 1.10 (1.07 to 1.14) | 11,609 / 50,358 | 23.1 (21.8 to 24.3) | 1.15 (1.11 to 1.20) |
|  |  |  |  |  |  |  |
| Period of discharge |  |  |  |  |  |  |
| 1^st^ January 2017 to 31^st^ March 2018 | 12,057 / 70,341 | 17.1 (16.1 to 18.2) | ref | 12,383 / 63,074 | 19.6 (18.5 to 20.8) | ref |
| 1^st^ April 2018 to 31^st^ March 2019 | 11,322 / 59,198 | 19.1 (17.8 to 20.4) | 1.04 (0.95 to 1.13) | 11,553 / 53,027 | 21.8 (20.5 to 23.0) | 1.04 (0.94 to 1.15) |
| 1^st^ April 2019 to 31^st^ March 2020 | 12,132 / 60,501 | 20.1 (18.9 to 21.4) | 1.01 (0.87 to 1.17) | 12,181 / 53,903 | 22.6 (21.3 to 23.9) | 0.99 (0.83 to 1.18) |
| 1^st^ April 2020 to 31^st^ March 2021 | 12,440 / 55,907 | 22.3 (21.0 to 23.5) | 1.11 (0.89 to 1.38) | 12,231 / 49,710 | 24.6 (23.3 to 25.9) | 1.08 (0.84 to 1.38) |

^a^ 95% CI of percentages calculated using bootstrap with sampling at the patient and practice levels, except for unknown IMD quintile for which sampling was done at the practice level only.

^b^ ORs adjusted for age, sex, month count (1-51) and calendar month (1-12) of discharge, and practice and patient-level clustering.

^c^ Recorded at any time up to and including the date of discharge.

^d^ Outpatient data available up to 31^st^ Oct 2020.

## **Table S3. Face-to-face/telephone contact with a doctor, nurse, or pharmacist from the general practice within 30 days after discharge, by demographic and clinical characteristics**

|  | **Face-to-face** | | | **Face-to face or telephone** | | |
| --- | --- | --- | --- | --- | --- | --- |
|  | Number of AKI episodes meeting indicator criteria / Number of AKI episodes eligible for inclusion | Percentage of AKI episodes meeting indicator criteria (95% CI) ^a^ | Odds ratio (OR) ^b^ | Number of AKI episodes meeting indicator criteria / Number of AKI episodes eligible for inclusion | Percentage of AKI episodes meeting indicator criteria (95% CI) ^a^ | Odds ratio (OR) ^b^ |
|  |  |  |  |  |  |  |
| Overall | 126,928 / 245,947 | 51.6 (50.7 to 52.5) | N/A | 178,591 / 245,947 | 72.6 (71.8 to 73.4) | N/A |
|  |  |  |  |  |  |  |
| Age |  |  |  |  |  |  |
| Less than 60 years | 22,654 / 42,855 | 52.9 (51.8 to 54.0) | ref | 29,585 / 42,855 | 69.0 (68.0 to 70.1) | ref |
| 60-79 years | 54,920 / 100,296 | 54.8 (53.7 to 55.7) | 1.11 (1.07 to 1.14) | 74,696 / 100,296 | 74.5 (73.6 to 75.3) | 1.47 (1.42 to 1.52) |
| 80 years or over | 49,354 / 102,796 | 48.0 (47.0 to 49.0) | 0.74 (0.72 to 0.76) | 74,310 / 102,796 | 72.3 (71.4 to 73.1) | 1.23 (1.19 to 1.28) |
|  |  |  |  |  |  |  |
| Sex |  |  |  |  |  |  |
| Female | 58,298 / 115,909 | 50.3 (49.3 to 51.3) | ref | 84,507 / 115,909 | 72.9 (72.0 to 73.8) | ref |
| Male | 68,630 / 130,038 | 52.8 (51.8 to 53.6) | 1.13 (1.11 to 1.16) | 94,084 / 130,038 | 72.4 (71.5 to 73.2) | 0.98 (0.95 to 1.00) |
|  |  |  |  |  |  |  |
| Ethnicity |  |  |  |  |  |  |
| White | 112,992 / 221,128 | 51.1 (50.1 to 52.0) | ref | 160,242 / 221,128 | 72.5 (71.6 to 73.2) | ref |
| Asian | 7239 / 12,306 | 58.8 (56.7 to 60.7) | 1.39 (1.32 to 1.48) | 9340 / 12,306 | 75.9 (74.2 to 77.4) | 1.28 (1.20 to 1.37) |
| Black | 4870 / 8873 | 54.9 (52.5 to 57.3) | 1.09 (1.02 to 1.17) | 6496 / 8873 | 73.2 (71.1 to 75.2) | 1.02 (0.95 to 1.10) |
| Other | 829 / 1579 | 52.5 (47.2 to 57.4) | 0.99 (0.86 to 1.14) | 1085 / 1579 | 68.7 (63.7 to 73.1) | 0.80 (0.69 to 0.93) |
| Unknown | 998 / 2061 | 48.4 (43.5 to 53.5) | 0.89 (0.78 to 1.01) | 1428 / 2061 | 69.3 (64.6 to 73.8) | 0.78 (0.68 to 0.89) |
|  |  |  |  |  |  |  |
| Patient-level IMD quintile |  |  |  |  |  |  |
| 1 (least deprived) | 22,955 / 43,429 | 52.9 (51.5 to 54.1) | ref | 32,569 / 43,429 | 75.0 (74.0 to 76.0) | ref |
| 2 | 24,645 / 47,122 | 52.3 (51.1 to 53.4) | 0.97 (0.94 to 1.01) | 34,721 / 47,122 | 73.7 (72.7 to 74.7) | 0.97 (0.93 to 1.01) |
| 3 | 24,505 / 47,126 | 52.0 (50.8 to 53.1) | 0.95 (0.91 to 0.99) | 34,457 / 47,126 | 73.1 (72.1 to 74.1) | 0.95 (0.91 to 0.99) |
| 4 | 26,115 / 51,086 | 51.1 (49.9 to 52.3) | 0.95 (0.91 to 0.99) | 36,809 / 51,086 | 72.1 (70.9 to 73.1) | 0.95 (0.91 to 1.00) |
| 5 (most deprived) | 28,604 / 56,189 | 50.9 (49.6 to 52.3) | 0.92 (0.88 to 0.97) | 39,902 / 56,189 | 71.0 (69.9 to 72.1) | 0.92 (0.88 to 0.97) |
| Unknown | 104 / 995 | 10.5 (0.1 to 91.8) | 0.14 (0.10 to 0.19) | 133 / 995 | 13.4 (0.3 to 87.6) | 0.05 (0.03 to 0.06) |
|  |  |  |  |  |  |  |
| CKD ^c^ |  |  |  |  |  |  |
| No | 58,750 / 114,314 | 51.4 (50.4 to 52.3) | ref | 81,338 / 114,314 | 71.2 (70.3 to 71.9) | ref |
| Yes | 68,178 / 131,633 | 51.8 (50.9 to 52.7) | 1.08 (1.06 to 1.11) | 97,253 / 131,633 | 73.9 (73.1 to 74.7) | 1.13 (1.10 to 1.16) |
|  |  |  |  |  |  |  |
| Diabetes ^c^ |  |  |  |  |  |  |
| No | 76,152 / 153,057 | 49.8 (48.8 to 50.7) | ref | 108,256 / 153,057 | 70.7 (69.9 to 71.5) | ref |
| Yes | 50,776 / 92,890 | 54.7 (53.6 to 55.8) | 1.32 (1.29 to 1.35) | 70,335 / 92,890 | 75.7 (74.8 to 76.6) | 1.40 (1.36 to 1.43) |
|  |  |  |  |  |  |  |
| Heart failure ^c^ |  |  |  |  |  |  |
| No | 84,518 / 166,768 | 50.7 (49.8 to 51.5) | ref | 118,355 / 166,768 | 71.0 (70.1 to 71.7) | ref |
| Yes | 42,410 / 79,179 | 53.6 (52.4 to 54.7) | 1.24 (1.21 to 1.27) | 60,236 / 79,179 | 76.1 (75.1 to 76.9) | 1.35 (1.32 to 1.39) |
|  |  |  |  |  |  |  |
| Myocardial infarction ^c^ |  |  |  |  |  |  |
| No | 98,935 / 194,268 | 50.9 (50.1 to 51.8) | ref | 139,403 / 194,268 | 71.8 (71.0 to 72.5) | ref |
| Yes | 27,993 / 51,679 | 54.2 (53.0 to 55.3) | 1.23 (1.19 to 1.26) | 39,188 / 51,679 | 75.8 (74.7 to 76.8) | 1.30 (1.26 to 1.35) |
|  |  |  |  |  |  |  |
| Major surgery during AKI episode |  |  |  |  |  |  |
| No | 120,699 / 235,682 | 51.2 (50.3 to 52.1) | ref | 170,352 / 235,682 | 72.3 (71.5 to 73.0) | ref |
| Yes | 6229 / 10,265 | 60.7 (58.9 to 62.7) | 1.59 (1.51 to 1.68) | 8239 / 10,265 | 80.3 (78.8 to 81.8) | 1.81 (1.70 to 1.93) |
|  |  |  |  |  |  |  |
| Sepsis during AKI episode |  |  |  |  |  |  |
| No | 108,187 / 208,598 | 51.9 (50.9 to 52.8) | ref | 151,777 / 208,598 | 72.8 (71.9 to 73.6) | ref |
| Yes | 18,741 / 37,349 | 50.2 (49.0 to 51.4) | 0.89 (0.87 to 0.92) | 26,814 / 37,349 | 71.8 (70.6 to 72.8) | 0.94 (0.91 to 0.97) |
|  |  |  |  |  |  |  |
| Primary care coded AKI within 30 days of discharge | |  |  |  |  |  |
| No | 101,543 / 197,996 | 51.3 (50.3 to 52.2) | ref | 142,192 / 197,996 | 71.8 (70.9 to 72.6) | ref |
| Yes | 25,385 / 47,951 | 52.9 (51.7 to 54.2) | 1.25 (1.22 to 1.29) | 36,399 / 47,951 | 75.9 (74.8 to 77.0) | 1.37 (1.33 to 1.41) |
|  |  |  |  |  |  |  |
| Face-to-face contact with a doctor, nurse, or pharmacist from the general practice within 30 days after discharge | |  |  |  |  |  |
| No | N/A | N/A | N/A | N/A | N/A | N/A |
| Yes | N/A | N/A | N/A | N/A | N/A | N/A |
|  |  |  |  |  |  |  |
| Outpatient contact with General Surgery, Endocrinology and Diabetes, Cardiology, or Renal Medicine within 30 days after discharge ^d^ | |  |  |  |  |  |
| No | 98,859 / 191,378 | 51.7 (50.7 to 52.5) | ref | 136,445 / 191,378 | 71.3 (70.4 to 72.1) | ref |
| Yes | 15,304 / 25,651 | 59.7 (58.2 to 61.0) | 1.44 (1.39 to 1.50) | 19,817 / 25,651 | 77.3 (76.1 to 78.4) | 1.47 (1.41 to 1.53) |
|  |  |  |  |  |  |  |
| Outpatient contact with General Surgery, Endocrinology and Diabetes, Cardiology, or Renal Medicine within 90 days after discharge ^d^ | |  |  |  |  |  |
| No | 78,678 / 155,670 | 50.5 (49.5 to 51.5) | ref | 109,067 / 155,670 | 70.1 (69.1 to 71.0) | ref |
| Yes | 32,250 / 53,871 | 59.9 (58.7 to 61.0) | 1.60 (1.55 to 1.64) | 41,516 / 53,871 | 77.1 (76.1 to 78.0) | 1.60 (1.55 to 1.65) |
|  |  |  |  |  |  |  |
| Period of discharge |  |  |  |  |  |  |
| 1^st^ January 2017 to 31^st^ March 2018 | 38,483 / 70,341 | 54.7 (53.7 to 55.9) | ref | 50,461 / 70,341 | 71.7 (70.6 to 72.8) | ref |
| 1^st^ April 2018 to 31^st^ March 2019 | 32,060 / 59,198 | 54.2 (53.0 to 55.3) | 1.17 (1.10 to 1.26) | 41,949 / 59,198 | 70.9 (69.7 to 71.9) | 1.10 (1.02 to 1.18) |
| 1^st^ April 2019 to 31^st^ March 2020 | 32,240 / 60,501 | 53.3 (52.1 to 54.4) | 1.33 (1.18 to 1.50) | 42,954 / 60,501 | 71.0 (70.0 to 71.9) | 1.25 (1.10 to 1.43) |
| 1^st^ April 2020 to 31^st^ March 2021 | 24,145 / 55,907 | 43.2 (41.9 to 44.4) | 0.89 (0.75 to 1.06) | 43,227 / 55,907 | 77.3 (76.3 to 78.2) | 2.13 (1.76 to 2.58) |

^a^ 95% CI of percentages calculated using bootstrap with sampling at the patient and practice levels, except for unknown IMD quintile for which sampling was done at the practice level only.

^b^ ORs adjusted for age, sex, month count (1-51) and calendar month (1-12) of discharge, and practice and patient-level clustering.

^c^ Recorded at any time up to and including the date of discharge.

^d^ Outpatient data only available up to 31^st^ Oct 2020.

## **Table S4. Creatinine measurement at 90±30 days and 365±90 days after discharge, by demographic and clinical characteristics**

|  | **At 90±30 days after discharge** | | | **At 365±90 days after discharge** | | |
| --- | --- | --- | --- | --- | --- | --- |
|  | Number of AKI episodes meeting indicator criteria / Number of AKI episodes eligible for inclusion | Percentage of AKI episodes meeting indicator criteria (95% CI) ^a^ | Odds ratio (OR) ^b^ | Number of AKI episodes meeting indicator criteria / Number of AKI episodes eligible for inclusion | Percentage of AKI episodes meeting indicator criteria (95% CI) ^a^ | Odds ratio (OR) ^b^ |
|  |  |  |  |  |  |  |
| Overall | 72,186 / 210,792 | 34.2 (33.7 to 34.8) | N/A | 89,971 / 157,712 | 57.0 (56.3 to 57.7) | N/A |
|  |  |  |  |  |  |  |
| Age |  |  |  |  |  |  |
| Less than 60 years | 9702 / 39,410 | 24.6 (23.7 to 25.5) | ref | 13,987 / 33,059 | 42.3 (41.1 to 43.4) | ref |
| 60-79 years | 32,844 / 88,330 | 37.2 (36.4 to 37.9) | 2.30 (2.21 to 2.40) | 42,113 / 69,197 | 60.9 (59.9 to 61.7) | 4.16 (3.93 to 4.42) |
| 80 years or over | 29,640 / 83,052 | 35.7 (35.0 to 36.4) | 2.13 (2.05 to 2.22) | 33,871 / 55,456 | 61.1 (60.0 to 62.0) | 4.30 (4.04 to 4.57) |
|  |  |  |  |  |  |  |
| Sex |  |  |  |  |  |  |
| Female | 34,044 / 99,139 | 34.3 (33.6 to 35.0) | ref | 42,520 / 74,500 | 57.1 (56.2 to 57.9) | ref |
| Male | 38,142 / 111,653 | 34.2 (33.5 to 34.9) | 1.02 (1.00 to 1.05) | 47,451 / 83,212 | 57.0 (56.2 to 57.9) | 1.08 (1.04 to 1.12) |
|  |  |  |  |  |  |  |
| Ethnicity |  |  |  |  |  |  |
| White | 65,471 / 188,693 | 34.7 (34.1 to 35.3) | ref | 80,608 / 140,349 | 57.4 (56.6 to 58.2) | ref |
| Asian | 3571 / 11,011 | 32.4 (30.7 to 34.3) | 1.13 (1.05 to 1.20) | 4992 / 8653 | 57.7 (55.5 to 59.9) | 1.36 (1.24 to 1.50) |
| Black | 2229 / 7864 | 28.3 (26.1 to 30.7) | 0.89 (0.82 to 0.97) | 3141 / 6093 | 51.6 (48.9 to 54.3) | 0.97 (0.87 to 1.08) |
| Other | 388 / 1408 | 27.6 (22.8 to 32.5) | 0.86 (0.72 to 1.02) | 535 / 1133 | 47.2 (41.1 to 52.8) | 0.85 (0.67 to 1.08) |
| Unknown | 527 / 1816 | 29.0 (23.8 to 33.7) | 0.72 (0.62 to 0.85) | 695 / 1484 | 46.8 (40.8 to 52.5) | 0.45 (0.36 to 0.57) |
|  |  |  |  |  |  |  |
| Patient-level IMD quintile |  |  |  |  |  |  |
| 1 (least deprived) | 13,305 / 37,006 | 36.0 (34.9 to 37.1) | ref | 16,094 / 27,693 | 58.1 (56.9 to 59.4) | ref |
| 2 | 14,277 / 40,314 | 35.4 (34.4 to 36.4) | 0.96 (0.92 to 1.01) | 17,466 / 30,121 | 58.0 (56.9 to 59.1) | 1.00 (0.93 to 1.07) |
| 3 | 14,042 / 40,312 | 34.8 (33.9 to 35.7) | 0.96 (0.91 to 1.00) | 17,291 / 29,938 | 57.8 (56.5 to 58.9) | 1.05 (0.97 to 1.12) |
| 4 | 14,630 / 43,980 | 33.3 (32.3 to 34.2) | 0.91 (0.87 to 0.96) | 18,931 / 33,117 | 57.2 (56.0 to 58.3) | 1.09 (1.01 to 1.17) |
| 5 (most deprived) | 15,876 / 48,305 | 32.9 (31.9 to 33.8) | 0.92 (0.88 to 0.97) | 20,118 / 36,188 | 55.6 (54.4 to 56.8) | 1.05 (0.97 to 1.13) |
| Unknown | 56 / 875 | 6.4 (0.1 to 88.2) | 0.13 (0.09 to 0.19) | 71 / 655 | 10.8 (0.2 to 89.1) | 0.05 (0.03 to 0.08) |
|  |  |  |  |  |  |  |
| CKD ^c^ |  |  |  |  |  |  |
| No | 29,258 / 100,849 | 29.0 (28.4 to 29.6) | ref | 40,017 / 79,950 | 50.1 (49.2 to 50.9) | ref |
| Yes | 42,928 / 109,943 | 39.0 (38.3 to 39.7) | 1.60 (1.55 to 1.64) | 49,954 / 77,762 | 64.2 (63.4 to 65.1) | 2.01 (1.92 to 2.09) |
|  |  |  |  |  |  |  |
| Diabetes ^c^ |  |  |  |  |  |  |
| No | 39,505 / 131,037 | 30.1 (29.5 to 30.8) | ref | 49,923 / 99,133 | 50.4 (49.5 to 51.1) | ref |
| Yes | 32,681 / 79,755 | 41.0 (40.1 to 41.8) | 1.97 (1.91 to 2.02) | 40,048 / 58,579 | 68.4 (67.3 to 69.3) | 3.75 (3.58 to 3.93) |
|  |  |  |  |  |  |  |
| Heart failure ^c^ |  |  |  |  |  |  |
| No | 43,735 / 145,659 | 30.0 (29.3 to 30.6) | ref | 59,429 / 113,574 | 52.3 (51.5 to 53.1) | ref |
| Yes | 28,451 / 65,133 | 43.7 (42.7 to 44.6) | 2.02 (1.96 to 2.08) | 30,542 / 44,138 | 69.2 (68.2 to 70.2) | 2.65 (2.53 to 2.77) |
|  |  |  |  |  |  |  |
| Myocardial infarction ^c^ |  |  |  |  |  |  |
| No | 54,340 / 167,288 | 32.5 (31.9 to 33.1) | ref | 69,311 / 126,868 | 54.6 (53.9 to 55.4) | ref |
| Yes | 17,846 / 43,504 | 41.0 (40.0 to 42.0) | 1.54 (1.49 to 1.59) | 20,660 / 30,844 | 67.0 (65.8 to 68.1) | 2.00 (1.90 to 2.11) |
|  |  |  |  |  |  |  |
| Major surgery during AKI episode |  |  |  |  |  |  |
| No | 68,689 / 201,158 | 34.1 (33.6 to 34.7) | ref | 85,158 / 149,303 | 57.0 (56.3 to 57.7) | ref |
| Yes | 3497 / 9634 | 36.3 (34.5 to 38.0) | 1.17 (1.10 to 1.24) | 4813 / 8409 | 57.2 (55.3 to 59.0) | 1.07 (0.99 to 1.16) |
|  |  |  |  |  |  |  |
| Sepsis during AKI episode |  |  |  |  |  |  |
| No | 61,669 / 179,142 | 34.4 (33.8 to 35.0) | ref | 76,896 / 134,313 | 57.3 (56.5 to 58.0) | ref |
| Yes | 10,517 / 31,650 | 33.2 (32.2 to 34.2) | 0.94 (0.90 to 0.97) | 13,075 / 23,399 | 55.9 (54.6 to 57.1) | 0.90 (0.86 to 0.95) |
|  |  |  |  |  |  |  |
| Primary care coded AKI within 30 days of discharge | |  |  |  |  |  |
| No | 56,250 / 169,697 | 33.1 (32.5 to 33.8) | ref | 71,423 / 126,878 | 56.3 (55.4 to 57.1) | ref |
| Yes | 15,936 / 41,095 | 38.8 (37.8 to 39.8) | 1.30 (1.25 to 1.34) | 18,548 / 30,834 | 60.2 (59.1 to 61.3) | 1.21 (1.15 to 1.27) |
|  |  |  |  |  |  |  |
| Face-to-face contact with a doctor, nurse, or pharmacist from the general practice within 30 days after discharge | |  |  |  |  |  |
| No | 30,625 / 100,600 | 30.4 (29.6 to 31.2) | ref | 38,868 / 73,349 | 53.0 (51.8 to 54.1) | ref |
| Yes | 41,561 / 110,192 | 37.7 (37.1 to 38.4) | 1.50 (1.46 to 1.54) | 51,103 / 84,363 | 60.6 (59.9 to 61.3) | 1.58 (1.52 to 1.64) |
|  |  |  |  |  |  |  |
| Outpatient contact with General Surgery, Endocrinology and Diabetes, Cardiology, or Renal Medicine within 30 days after discharge ^d^ | |  |  |  |  |  |
| No | 54,480 / 163,385 | 33.3 (32.7 to 34.0) | ref | 68,168 / 121,466 | 56.1 (55.3 to 56.8) | ref |
| Yes | 9439 / 22,703 | 41.6 (40.3 to 42.8) | 1.61 (1.55 to 1.68) | 11,374 / 17,660 | 64.4 (62.9 to 65.8) | 1.82 (1.72 to 1.93) |
|  |  |  |  |  |  |  |
| Outpatient contact with General Surgery, Endocrinology and Diabetes, Cardiology, or Renal Medicine within 90 days after discharge ^d^ | |  |  |  |  |  |
| No | 41,550 / 131,053 | 31.7 (31.1 to 32.3) | ref | 52,430 / 96,439 | 54.4 (53.5 to 55.1) | ref |
| Yes | 20,429 / 48,774 | 41.9 (40.9 to 42.9) | 1.84 (1.78 to 1.90) | 24,542 / 37,951 | 64.7 (63.6 to 65.7) | 2.04 (1.95 to 2.14) |
|  |  |  |  |  |  |  |
| Period of discharge |  |  |  |  |  |  |
| 1^st^ January 2017 to 31^st^ March 2018 | 21,551 / 60,541 | 35.6 (34.7 to 36.5) | ref | 27,173 / 45,425 | 59.8 (58.8 to 60.8) | ref |
| 1^st^ April 2018 to 31^st^ March 2019 | 18,018 / 50,901 | 35.4 (34.5 to 36.3) | 1.14 (1.05 to 1.24) | 22,032 / 37,802 | 58.3 (57.2 to 59.6) | 0.87 (0.77 to 0.97) |
| 1^st^ April 2019 to 31^st^ March 2020 | 16,737 / 51,677 | 32.4 (31.5 to 33.2) | 1.08 (0.94 to 1.24) | 20,389 / 38,603 | 52.8 (51.7 to 53.9) | 0.56 (0.46 to 0.68) |
| 1^st^ April 2020 to 31^st^ March 2021 | 15,880 / 47,673 | 33.3 (32.4 to 34.2) | 1.37 (1.11 to 1.68) | 20,377 / 35,882 | 56.8 (55.7 to 57.9) | 0.75 (0.56 to 1.01) |

^a^ 95% CI of percentages calculated using bootstrap with sampling at the patient and practice levels, except for unknown IMD quintile for which sampling was done at the practice level only.

^b^ ORs adjusted for age, sex, month count (1-51) and calendar month (1-12) of discharge, and practice and patient-level clustering.

^c^ Recorded at any time up to and including the date of discharge.

^d^ Outpatient data only available up to 31^st^ Oct 2020.

## **Table S5.** **ACR measurement at 90±30 days and 365±90 days after discharge, by demographic and clinical characteristics**

|  | **At 90±30 days after discharge** | | | **At 365±90 days after discharge** | | |
| --- | --- | --- | --- | --- | --- | --- |
|  | Number of AKI episodes meeting indicator criteria / Number of AKI episodes eligible for inclusion | Percentage of AKI episodes meeting indicator criteria (95% CI) ^a^ | Odds ratio (OR) ^b^ | Number of AKI episodes meeting indicator criteria / Number of AKI episodes eligible for inclusion | Percentage of AKI episodes meeting indicator criteria (95% CI) ^a^ | Odds ratio (OR) ^b^ |
|  |  |  |  |  |  |  |
| Overall | 8905 / 210,792 | 4.2 (4.0 to 4.4) | N/A | 19,212 / 157,712 | 12.2 (11.8 to 12.6) | N/A |
|  |  |  |  |  |  |  |
| Age |  |  |  |  |  |  |
| Less than 60 years | 1041 / 39,410 | 2.6 (2.3 to 3.0) | ref | 2617 / 33,059 | 7.9 (7.3 to 8.6) | ref |
| 60-79 years | 4546 / 88,330 | 5.1 (4.9 to 5.4) | 2.47 (2.26 to 2.71) | 10,223 / 69,197 | 14.8 (14.2 to 15.4) | 3.79 (3.45 to 4.16) |
| 80 years or over | 3318 / 83,052 | 4.0 (3.7 to 4.3) | 1.80 (1.64 to 1.97) | 6372 / 55,456 | 11.5 (10.9 to 12.1) | 2.36 (2.15 to 2.59) |
|  |  |  |  |  |  |  |
| Sex |  |  |  |  |  |  |
| Female | 3824 / 99,139 | 3.9 (3.6 to 4.1) | ref | 8081 / 74,500 | 10.8 (10.4 to 11.4) | ref |
| Male | 5081 / 111,653 | 4.6 (4.3 to 4.8) | 1.28 (1.20 to 1.35) | 11,131 / 83,212 | 13.4 (12.9 to 13.9) | 1.65 (1.55 to 1.76) |
|  |  |  |  |  |  |  |
| Ethnicity |  |  |  |  |  |  |
| White | 7714 / 188,693 | 4.1 (3.9 to 4.3) | ref | 16,297 / 140,349 | 11.6 (11.2 to 12.0) | ref |
| Asian | 630 / 11,011 | 5.7 (4.9 to 6.6) | 1.69 (1.49 to 1.91) | 1605 / 8653 | 18.5 (16.7 to 20.3) | 3.24 (2.82 to 3.73) |
| Black | 444 / 7864 | 5.6 (4.7 to 6.6) | 1.61 (1.39 to 1.86) | 974 / 6093 | 16.0 (13.9 to 18.0) | 2.35 (1.99 to 2.78) |
| Other | 53 / 1408 | 3.8 (2.2 to 5.9) | 1.05 (0.73 to 1.52) | 158 / 1133 | 13.9 (10.1 to 18.3) | 2.04 (1.43 to 2.92) |
| Unknown | 64 / 1816 | 3.5 (2.0 to 5.2) | 0.86 (0.61 to 1.21) | 178 / 1484 | 12.0 (7.9 to 16.2) | 0.91 (0.64 to 1.30) |
|  |  |  |  |  |  |  |
| Patient-level IMD quintile |  |  |  |  |  |  |
| 1 (least deprived) | 1496 / 37,006 | 4.0 (3.6 to 4.5) | ref | 3131 / 27,693 | 11.3 (10.4 to 12.1) | ref |
| 2 | 1681 / 40,314 | 4.2 (3.8 to 4.6) | 1.04 (0.95 to 1.15) | 3557 / 30,121 | 11.8 (11.0 to 12.6) | 1.07 (0.96 to 1.19) |
| 3 | >1640 / 40,312 ^c^ | >4.0 (3.7 to 4.5) ^c^ | 1.03 (0.93 to 1.14) | 3627 / 29,938 | 12.1 (11.4 to 13.0) | 1.17 (1.04 to 1.30) |
| 4 | 1874 / 43,980 | 4.3 (3.9 to 4.6) | 1.09 (0.98 to 1.20) | 4164 / 33,117 | 12.6 (11.9 to 13.4) | 1.23 (1.10 to 1.38) |
| 5 (most deprived) | 2203 / 48,305 | 4.6 (4.2 to 5.0) | 1.18 (1.06 to 1.31) | 4724 / 36,188 | 13.1 (12.3 to 13.9) | 1.37 (1.22 to 1.54) |
| Unknown | <5 / 875 | - | 0.06 (0.01 to 0.30) | 9 / 655 | 1.4 (0.0 to 42.3) | - ^d^ |
|  |  |  |  |  |  |  |
| CKD ^e^ |  |  |  |  |  |  |
| No | 3198 / 100,849 | 3.2 (3.0 to 3.4) | ref | 7342 / 79,950 | 9.2 (8.8 to 9.6) | ref |
| Yes | 5707 / 109,943 | 5.2 (4.9 to 5.5) | 1.84 (1.73 to 1.96) | 11,870 / 77,762 | 15.3 (14.6 to 15.9) | 2.51 (2.34 to 2.68) |
|  |  |  |  |  |  |  |
| Diabetes ^e^ |  |  |  |  |  |  |
| No | 2182 / 131,037 | 1.7 (1.5 to 1.8) | ref | 5025 / 99,133 | 5.1 (4.7 to 5.4) | ref |
| Yes | 6723 / 79,755 | 8.4 (8.0 to 8.8) | 8.57 (7.91 to 9.29) | 14,187 / 58,579 | 24.2 (23.4 to 25.0) | 19.1 (17.3 to 21.0) |
|  |  |  |  |  |  |  |
| Heart failure ^e^ |  |  |  |  |  |  |
| No | 5734 / 145,659 | 3.9 (3.7 to 4.2) | ref | 12,735 / 113,574 | 11.2 (10.8 to 11.6) | ref |
| Yes | 3171 / 65,133 | 4.9 (4.5 to 5.2) | 1.24 (1.16 to 1.31) | 6477 / 44,138 | 14.7 (13.9 to 15.4) | 1.50 (1.40 to 1.60) |
|  |  |  |  |  |  |  |
| Myocardial infarction ^e^ |  |  |  |  |  |  |
| No | 6673 / 167,288 | 4.0 (3.8 to 4.2) | ref | 14,428 / 126,868 | 11.4 (10.9 to 11.8) | ref |
| Yes | 2232 / 43,504 | 5.1 (4.7 to 5.5) | 1.29 (1.21 to 1.38) | 4784 / 30,844 | 15.5 (14.7 to 16.4) | 1.65 (1.53 to 1.78) |
|  |  |  |  |  |  |  |
| Major surgery during AKI episode |  |  |  |  |  |  |
| No | 8463 / 201,158 | 4.2 (4.0 to 4.4) | ref | 18,163 / 149,303 | 12.2 (11.7 to 12.6) | ref |
| Yes | 442 / 9634 | 4.6 (3.9 to 5.4) | 1.10 (0.97 to 1.24) | 1049 / 8409 | 12.5 (11.3 to 13.8) | 1.02 (0.90 to 1.15) |
|  |  |  |  |  |  |  |
| Sepsis during AKI episode |  |  |  |  |  |  |
| No | 7632 / 179,142 | 4.3 (4.1 to 4.5) | ref | 16,417 / 134,313 | 12.2 (11.8 to 12.7) | ref |
| Yes | 1273 / 31,650 | 4.0 (3.7 to 4.4) | 0.92 (0.86 to 1.00) | 2795 / 23,399 | 11.9 (11.2 to 12.8) | 0.94 (0.87 to 1.02) |
|  |  |  |  |  |  |  |
| Primary care coded AKI within 30 days of discharge | |  |  |  |  |  |
| No | 6880 / 169,697 | 4.1 (3.8 to 4.3) | ref | 14,913 / 126,878 | 11.8 (11.3 to 12.2) | ref |
| Yes | 2025 / 41,095 | 4.9 (4.5 to 5.4) | 1.22 (1.14 to 1.31) | 4299 / 30,834 | 13.9 (13.2 to 14.8) | 1.24 (1.16 to 1.33) |
|  |  |  |  |  |  |  |
| Face-to-face contact with a doctor, nurse, or pharmacist from the general practice within 30 days after discharge | |  |  |  |  |  |
| No | 3663 / 100,600 | 3.6 (3.4 to 3.9) | ref | 7556 / 73,349 | 10.3 (9.8 to 10.8) | ref |
| Yes | 5242 / 110,192 | 4.8 (4.5 to 5.0) | 1.38 (1.30 to 1.46) | 11,656 / 84,363 | 13.8 (13.3 to 14.3) | 1.60 (1.51 to 1.69) ^f^ |
|  |  |  |  |  |  |  |
| Outpatient contact with General Surgery, Endocrinology and Diabetes, Cardiology, or Renal Medicine within 30 days after discharge ^g^ | |  |  |  |  |  |
| No | 6645 / 163,385 | 4.1 (3.8 to 4.3) | ref | 14,253 / 121,466 | 11.7 (11.3 to 12.2) | ref |
| Yes | 1306 / 22,703 | 5.8 (5.2 to 6.4) | 1.56 (1.44 to 1.69) | 2735 / 17,660 | 15.5 (14.3 to 16.5) | 1.64 (1.50 to 1.78) |
|  |  |  |  |  |  |  |
| Outpatient contact with General Surgery, Endocrinology and Diabetes, Cardiology, or Renal Medicine within 90 days after discharge ^g^ | |  |  |  |  |  |
| No | 5016 / 131,053 | 3.8 (3.6 to 4.0) | ref | 10,675 / 96,439 | 11.1 (10.6 to 11.5) | ref |
| Yes | 2713 / 48,774 | 5.6 (5.2 to 6.0) | 1.61 (1.51 to 1.72) | 5786 / 37,951 | 15.2 (14.4 to 16.0) | 1.82 (1.69 to 1.95) |
|  |  |  |  |  |  |  |
| Period of discharge |  |  |  |  |  |  |
| 1^st^ January 2017 to 31^st^ March 2018 | 2654 / 60,541 | 4.4 (4.0 to 4.7) | ref | 6243 / 45,425 | 13.7 (13.1 to 14.5) | ref |
| 1^st^ April 2018 to 31^st^ March 2019 | 2447 / 50,901 | 4.8 (4.4 to 5.2) | 1.09 (0.92 to 1.30) | 4904 / 37,802 | 13.0 (12.3 to 13.7) | 0.71 (0.60 to 0.84) |
| 1^st^ April 2019 to 31^st^ March 2020 | 1987 / 51,677 | 3.8 (3.5 to 4.2) | 0.80 (0.59 to 1.08) | 3704 / 38,603 | 9.6 (9.0 to 10.2) | 0.32 (0.23 to 0.43) |
| 1^st^ April 2020 to 31^st^ March 2021 | 1817 / 47,673 | 3.8 (3.5 to 4.2) | 0.78 (0.51 to 1.21) | 4361 / 35,882 | 12.2 (11.5 to 12.8) | 0.43 (0.28 to 0.67) |

^a^ 95% CI of percentages calculated using bootstrap with sampling at the patient and practice levels, except for unknown IMD quintile (ACR measurement at 365±90 days after discharge) for which sampling was done at the practice level only.

^b^ ORs adjusted for age, sex, month count (1-51) and calendar month (1-12) of discharge, and practice and patient-level clustering.

^c^ Exact values of numerator and percentage not reported to prevent disclosure of numerator (<5) for IMD quintile unknown.

^d^ OR for IMD quintile unknown not estimated because of difficulties in the modelling.

^e^ Recorded at any time up to and including the date of discharge.

^f^ OR adjusted for age, sex, month count (1-51) and calendar month (1-12) of discharge, and patient-level clustering only. Practice-level clustering not adjusted for because of difficulties in the modelling.

^g^ Outpatient data only available up to 31^st^ Oct 2020.

## **Table S6. Blood pressure measurement at 90±30 days and 365±90 days after discharge, by demographic and clinical characteristics**

|  | **At 90±30 days after discharge** | | | **At 365±90 days after discharge** | | |
| --- | --- | --- | --- | --- | --- | --- |
|  | Number of AKI episodes meeting indicator criteria / Number of AKI episodes eligible for inclusion | Percentage of AKI episodes meeting indicator criteria (95% CI) ^a^ | Odds ratio (OR) ^b^ | Number of AKI episodes meeting indicator criteria / Number of AKI episodes eligible for inclusion | Percentage of AKI episodes meeting indicator criteria (95% CI) ^a^ | Odds ratio (OR) ^b^ |
|  |  |  |  |  |  |  |
| Overall | 72,844 / 210,792 | 34.6 (34.0 to 35.1) | N/A | 90,207 / 157,712 | 57.2 (56.4 to 57.9) | N/A |
|  |  |  |  |  |  |  |
| Age |  |  |  |  |  |  |
| Less than 60 years | 9899 / 39,410 | 25.1 (24.2 to 26.0) | ref | 14,206 / 33,059 | 43.0 (41.8 to 44.1) | ref |
| 60-79 years | 31,713 / 88,330 | 35.9 (35.2 to 36.7) | 2.01 (1.94 to 2.09) | 41,395 / 69,197 | 59.8 (58.9 to 60.7) | 3.43 (3.25 to 3.62) |
| 80 years or over | 31,232 / 83,052 | 37.6 (36.8 to 38.4) | 2.22 (2.13 to 2.31) | 34,606 / 55,456 | 62.4 (61.3 to 63.5) | 4.10 (3.87 to 4.35) |
|  |  |  |  |  |  |  |
| Sex |  |  |  |  |  |  |
| Female | 34,579 / 99,139 | 34.9 (34.2 to 35.6) | ref | 42,865 / 74,500 | 57.5 (56.6 to 58.5) | ref |
| Male | 38,265 / 111,653 | 34.3 (33.5 to 35.0) | 1.00 (0.98 to 1.03) | 47,342 / 83,212 | 56.9 (56.1 to 57.7) | 1.03 (0.99 to 1.07) |
|  |  |  |  |  |  |  |
| Ethnicity |  |  |  |  |  |  |
| White | 65,284 / 188,693 | 34.6 (34.0 to 35.2) | ref | 80,401 / 140,349 | 57.3 (56.5 to 58.0) | ref |
| Asian | 4016 / 11,011 | 36.5 (34.6 to 38.4) | 1.30 (1.22 to 1.39) | 5149 / 8653 | 59.5 (57.5 to 61.7) | 1.44 (1.32 to 1.58) |
| Black | 2589 / 7864 | 32.9 (30.9 to 35.1) | 1.15 (1.06 to 1.24) | 3394 / 6093 | 55.7 (53.1 to 58.3) | 1.24 (1.12 to 1.38) |
| Other | 411 / 1408 | 29.2 (24.8 to 33.8) | 1.00 (0.85 to 1.18) | 565 / 1133 | 49.9 (43.6 to 55.6) | 1.00 (0.80 to 1.25) |
| Unknown | 544 / 1816 | 30.0 (25.6 to 34.2) | 0.85 (0.73 to 0.99) | 698 / 1484 | 47.0 (41.8 to 52.7) | 0.59 (0.48 to 0.73) |
|  |  |  |  |  |  |  |
| Patient-level IMD quintile |  |  |  |  |  |  |
| 1 (least deprived) | 12,772 / 37,006 | 34.5 (33.5 to 35.6) | ref | 15,785 / 27,693 | 57.0 (55.8 to 58.4) | ref |
| 2 | 13,976 / 40,314 | 34.7 (33.7 to 35.6) | 1.00 (0.96 to 1.05) | 17,157 / 30,121 | 57.0 (55.7 to 58.3) | 1.02 (0.95 to 1.09) |
| 3 | 14,027 / 40,312 | 34.8 (33.8 to 35.8) | 1.04 (0.99 to 1.09) | 17,122 / 29,938 | 57.2 (56.0 to 58.3) | 1.09 (1.02 to 1.17) |
| 4 | 15,260 / 43,980 | 34.7 (33.8 to 35.7) | 1.05 (1.00 to 1.10) | 19,158 / 33,117 | 57.8 (56.7 to 59.0) | 1.19 (1.11 to 1.27) |
| 5 (most deprived) | 16,759 / 48,305 | 34.7 (33.7 to 35.6) | 1.07 (1.01 to 1.12) | 20,920 / 36,188 | 57.8 (56.6 to 59.0) | 1.26 (1.17 to 1.35) |
| Unknown | 50 / 875 | 5.7 (0.0 to 90.5) | 0.10 (0.07 to 0.15) | 65 / 655 | 9.9 (0.1 to 90.6) | 0.04 (0.02 to 0.06) |
|  |  |  |  |  |  |  |
| CKD ^c^ |  |  |  |  |  |  |
| No | 30,765 / 100,849 | 30.5 (29.9 to 31.1) | ref | 41,134 / 79,950 | 51.4 (50.6 to 52.3) | ref |
| Yes | 42,079 / 109,943 | 38.3 (37.5 to 39.0) | 1.33 (1.29 to 1.36) | 49,073 / 77,762 | 63.1 (62.2 to 64.0) | 1.61 (1.55 to 1.67) |
|  |  |  |  |  |  |  |
| Diabetes ^c^ |  |  |  |  |  |  |
| No | 40,722 / 131,037 | 31.1 (30.4 to 31.6) | ref | 51,023 / 99,133 | 51.5 (50.6 to 52.3) | ref |
| Yes | 32,122 / 79,755 | 40.3 (39.5 to 41.1) | 1.71 (1.66 to 1.76) | 39,184 / 58,579 | 66.9 (65.8 to 67.9) | 2.85 (2.73 to 2.97) |
|  |  |  |  |  |  |  |
| Heart failure ^c^ |  |  |  |  |  |  |
| No | 45,129 / 145,659 | 31.0 (30.4 to 31.6) | ref | 60,562 / 113,574 | 53.3 (52.6 to 54.1) | ref |
| Yes | 27,715 / 65,133 | 42.6 (41.7 to 43.5) | 1.72 (1.67 to 1.77) | 29,645 / 44,138 | 67.2 (66.1 to 68.1) | 2.03 (1.94 to 2.12) |
|  |  |  |  |  |  |  |
| Myocardial infarction ^c^ |  |  |  |  |  |  |
| No | 54,640 / 167,288 | 32.7 (32.1 to 33.2) | ref | 69,484 / 126,868 | 54.8 (54.0 to 55.5) | ref |
| Yes | 18,204 / 43,504 | 41.8 (40.9 to 42.9) | 1.54 (1.49 to 1.59) | 20,723 / 30,844 | 67.2 (65.9 to 68.5) | 1.92 (1.83 to 2.02) |
|  |  |  |  |  |  |  |
| Major surgery during AKI episode |  |  |  |  |  |  |
| No | 69,314 / 201,158 | 34.5 (33.8 to 35.0) | ref | 85,492 / 149,303 | 57.3 (56.5 to 58.0) | ref |
| Yes | 3530 / 9634 | 36.6 (35.0 to 38.3) | 1.21 (1.14 to 1.28) | 4715 / 8409 | 56.1 (54.3 to 57.9) | 0.98 (0.91 to 1.06) |
|  |  |  |  |  |  |  |
| Sepsis during AKI episode |  |  |  |  |  |  |
| No | 62,120 / 179,142 | 34.7 (34.1 to 35.3) | ref | 76,840 / 134,313 | 57.2 (56.4 to 58.0) | ref |
| Yes | 10,724 / 31,650 | 33.9 (32.9 to 34.9) | 0.94 (0.90 to 0.97) | 13,367 / 23,399 | 57.1 (55.9 to 58.4) | 0.94 (0.90 to 0.99) |
|  |  |  |  |  |  |  |
| Primary care coded AKI within 30 days of discharge | |  |  |  |  |  |
| No | 57,280 / 169,697 | 33.8 (33.1 to 34.4) | ref | 71,814 / 126,878 | 56.6 (55.8 to 57.4) | ref |
| Yes | 15,564 / 41,095 | 37.9 (36.9 to 38.9) | 1.19 (1.15 to 1.22) | 18,393 / 30,834 | 59.7 (58.5 to 60.8) | 1.15 (1.09 to 1.20) |
|  |  |  |  |  |  |  |
| Face-to-face contact with a doctor, nurse, or pharmacist from the general practice within 30 days after discharge | |  |  |  |  |  |
| No | 31,304 / 100,600 | 31.1 (30.3 to 31.9) | ref | 39,202 / 73,349 | 53.4 (52.3 to 54.5) | ref |
| Yes | 41,540 / 110,192 | 37.7 (37.1 to 38.4) | 1.41 (1.38 to 1.45) | 51,005 / 84,363 | 60.5 (59.7 to 61.2) | 1.44 (1.39 to 1.50) |
|  |  |  |  |  |  |  |
| Outpatient contact with General Surgery, Endocrinology and Diabetes, Cardiology, or Renal Medicine within 30 days after discharge ^d^ | |  |  |  |  |  |
| No | 56,774 / 163,385 | 34.7 (34.1 to 35.4) | ref | 69,204 / 121,466 | 57.0 (56.2 to 57.8) | ref |
| Yes | 9409 / 22,703 | 41.4 (40.2 to 42.8) | 1.50 (1.44 to 1.56) | 11,096 / 17,660 | 62.8 (61.3 to 64.5) | 1.56 (1.47 to 1.65) |
|  |  |  |  |  |  |  |
| Outpatient contact with General Surgery, Endocrinology and Diabetes, Cardiology, or Renal Medicine within 90 days after discharge ^d^ | |  |  |  |  |  |
| No | 44,024 / 131,053 | 33.6 (32.9 to 34.2) | ref | 53,793 / 96,439 | 55.8 (54.9 to 56.6) | ref |
| Yes | 20,581 / 48,774 | 42.2 (41.2 to 43.2) | 1.69 (1.63 to 1.74) | 24,021 / 37,951 | 63.3 (62.1 to 64.4) | 1.72 (1.65 to 1.80) |
|  |  |  |  |  |  |  |
| Period of discharge |  |  |  |  |  |  |
| 1^st^ January 2017 to 31^st^ March 2018 | 23,173 / 60,541 | 38.3 (37.5 to 39.2) | ref | 29,975 / 45,425 | 66.0 (65.0 to 67.0) | ref |
| 1^st^ April 2018 to 31^st^ March 2019 | 20,021 / 50,901 | 39.3 (38.4 to 40.4) | 1.22 (1.13 to 1.32) | 24,033 / 37,802 | 63.6 (62.4 to 64.7) | 0.83 (0.74 to 0.93) |
| 1^st^ April 2019 to 31^st^ March 2020 | 16,991 / 51,677 | 32.9 (32.0 to 33.8) | 0.95 (0.83 to 1.09) | 17,202 / 38,603 | 44.6 (43.5 to 45.6) | 0.22 (0.18 to 0.26) |
| 1^st^ April 2020 to 31^st^ March 2021 | 12,659 / 47,673 | 26.6 (25.7 to 27.4) | 0.76 (0.62 to 0.92) | 18,997 / 35,882 | 52.9 (51.8 to 54.1) | 0.40 (0.30 to 0.54) |

^a^ 95% CI of percentages calculated using bootstrap with sampling at the patient and practice levels, except for unknown IMD quintile for which sampling was done at the practice level only.

^b^ ORs adjusted for age, sex, month count (1-51) and calendar month (1-12) of discharge, and practice and patient-level clustering.

^c^ Recorded at any time up to and including the date of discharge.

^d^ Outpatient data only available up to 31^st^ Oct 2020.

## **Table S7.** **Coding of *de novo* CKD at 90±30 days after discharge, by demographic and clinical characteristics**

|  | Number of AKI episodes meeting indicator criteria / Number of AKI episodes eligible for inclusion | Percentage of AKI episodes meeting indicator criteria (95% CI) ^a^ | Odds ratio (OR) ^b^ |
| --- | --- | --- | --- |
|  |  |  |  |
| Overall | 1455 / 8489 | 17.1 (15.5 to 18.8) | N/A |
|  |  |  |  |
| Age |  |  |  |
| Less than 60 years | 162 / 819 | 19.8 (15.0 to 25.1) | ref |
| 60-79 years | 760 / 4179 | 18.2 (16.0 to 20.7) | 0.84 (0.51 to 1.39) |
| 80 years or over | 533 / 3491 | 15.3 (13.2 to 17.7) | 0.53 (0.32 to 0.89) |
|  |  |  |  |
| Sex |  |  |  |
| Female | 688 / 4216 | 16.3 (14.3 to 18.4) | ref |
| Male | 767 / 4273 | 17.9 (15.8 to 20.2) | 1.28 (0.95 to 1.71) |
|  |  |  |  |
| Ethnicity |  |  |  |
| White | 1343 / 7791 | 17.2 (15.6 to 19.0) | ref |
| Asian | 57 / 322 | 17.7 (10.9 to 26.4) | 0.93 (0.43 to 2.01) |
| Black | 44 / 286 | 15.4 (8.5 to 24.1) | 0.63 (0.27 to 1.52) |
| Other | <5 / 34 | - | 0.13 (0.01 to 1.99) |
| Unknown | >5 / 56 ^c^ | >8.0 (6.6 to 28.1) ^c^ | 0.58 (0.09 to 3.84) |
|  |  |  |  |
| Patient-level IMD quintile |  |  |  |
| 1 (least deprived) | 270 / 1675 | 16.1 (12.9 to 19.7) | ref |
| 2 | 308 / 1836 | 16.8 (13.9 to 20.2) | 1.05 (0.66 to 1.67) |
| 3 | >270 / 1692 ^d^ | >15.0 (13.2 to 19.7) ^d^ | 0.88 (0.54 to 1.43) |
| 4 | 264 / 1624 | 16.3 (13.1 to 20.2) | 0.99 (0.60 to 1.62) |
| 5 (most deprived) | 336 / 1655 | 20.3 (18.1 to 22.7) | 1.53 (0.93 to 2.53) |
| Unknown | <5 / 7 | - | 54.2 (0.86 to 3419.7) |
|  |  |  |  |
| CKD ^e^ |  |  |  |
| No | N/A | N/A | N/A |
| Yes | N/A | N/A | N/A |
|  |  |  |  |
| Diabetes ^e^ |  |  |  |
| No | 916 / 5187 | 17.7 (15.6 to 19.7) | ref |
| Yes | 539 / 3302 | 16.3 (13.9 to 18.6) | 0.76 (0.56 to 1.03) |
|  |  |  |  |
| Heart failure ^e^ |  |  |  |
| No | 930 / 5147 | 18.1 (15.9 to 20.2) | ref |
| Yes | 525 / 3342 | 15.7 (13.4 to 18.0) | 0.73 (0.54 to 0.99) |
|  |  |  |  |
| Myocardial infarction ^e^ |  |  |  |
| No | 1136 / 6545 | 17.4 (15.6 to 19.2) | ref |
| Yes | 319 / 1944 | 16.4 (13.4 to 19.5) | 0.96 (0.68 to 1.36) |
|  |  |  |  |
| Major surgery during AKI episode |  |  |  |
| No | 1374 / 8018 | 17.1 (15.4 to 18.9) | ref |
| Yes | 81 / 471 | 17.2 (11.8 to 23.7) | 0.89 (0.50 to 1.59) |
|  |  |  |  |
| Sepsis during AKI episode |  |  |  |
| No | 1266 / 7288 | 17.4 (15.7 to 19.2) | ref |
| Yes | 189 / 1201 | 15.7 (12.1 to 19.8) | 0.67 (0.45 to 1.00) |
|  |  |  |  |
| Primary care coded AKI within 30 days of discharge | |  |  |
| No | 1043 / 6408 | 16.3 (14.5 to 18.0) | ref |
| Yes | 412 / 2081 | 19.8 (16.7 to 23.1) | 1.55 (1.12 to 2.13) |
|  |  |  |  |
| Face-to-face contact with a doctor, nurse, or pharmacist from the general practice within 30 days after discharge | |  |  |
| No | 543 / 3545 | 15.3 (13.2 to 17.8) | ref |
| Yes | 912 / 4944 | 18.4 (16.3 to 20.5) | 1.71 (1.28 to 2.28) |
|  |  |  |  |
| Outpatient contact with General Surgery, Endocrinology and Diabetes, Cardiology, or Renal Medicine within 30 days after discharge ^f^ | |  |  |
| No | 1021 / 6307 | 16.2 (14.5 to 18.1) | ref |
| Yes | 255 / 1204 | 21.2 (16.8 to 25.9) | 2.04 (1.38 to 3.03) |
|  |  |  |  |
| Outpatient contact with General Surgery, Endocrinology and Diabetes, Cardiology, or Renal Medicine within 90 days after discharge ^f^ | |  |  |
| No | 715 / 4694 | 15.2 (13.3 to 17.3) | ref |
| Yes | 521 / 2566 | 20.3 (17.4 to 23.3) | 1.96 (1.41 to 2.71) |
|  |  |  |  |
| Period of discharge |  |  |  |
| 1^st^ January 2017 to 31^st^ March 2018 | 462 / 2669 | 17.3 (14.6 to 20.1) | ref |
| 1^st^ April 2018 to 31^st^ March 2019 | 365 / 2092 | 17.4 (14.6 to 20.7) | 1.01 (0.43 to 2.38) |
| 1^st^ April 2019 to 31^st^ March 2020 | 297 / 1873 | 15.9 (12.7 to 19.1) | 0.66 (0.15 to 2.93) |
| 1^st^ April 2020 to 31^st^ March 2021 | 331 / 1855 | 17.8 (14.6 to 21.1) | 0.95 (0.11 to 8.02) |

^a^ 95% CI of percentages calculated using bootstrap with sampling at the patient and practice levels, except for unknown ethnicity and IMD quintile 5 for which sampling was done at the practice level only.

^b^ ORs adjusted for age, sex, month count (1-51) and calendar month (1-12) of discharge, and practice and patient-level clustering.

^c^ Exact values of numerator and percentage not reported to prevent disclosure of numerator (<5) for ‘Other’ ethnicity.

^d^ Exact values of numerator and percentage not reported to prevent disclosure of numerator (<5) for unknown IMD quintile.

^e^ Recorded at any time up to and including the date of discharge.

^f^ Outpatient data only available up to 31^st^ Oct 2020.

## **Table S8. RAASi continuation and initiation within 90 days after discharge, by demographic and clinical characteristics**

|  | **RAASi continuation** | | | **RAASi initiation** | | |
| --- | --- | --- | --- | --- | --- | --- |
|  | Number of AKI episodes meeting indicator criteria / Number of AKI episodes eligible for inclusion | Percentage of AKI episodes meeting indicator criteria (95% CI) ^a^ | Odds ratio (OR) ^b^ | Number of AKI episodes meeting indicator criteria / Number of AKI episodes eligible for inclusion | Percentage of AKI episodes meeting indicator criteria (95% CI) ^a^ | Odds ratio (OR) ^b^ |
|  |  |  |  |  |  |  |
| Overall | 14,811 / 20,985 | 70.6 (69.3 to 71.8) | N/A | 1187 / 12,274 | 9.7 (8.6 to 10.7) | N/A |
|  |  |  |  |  |  |  |
| Age |  |  |  |  |  |  |
| Less than 60 years | 1727 / 2243 | 77.0 (73.6 to 80.3) | ref | 239 / 1545 | 15.5 (11.8 to 19.1) | ref |
| 60-79 years | 7540 / 10,475 | 72.0 (70.2 to 73.6) | 0.66 (0.55 to 0.79) | 559 / 4965 | 11.3 (9.7 to 13.0) | 0.36 (0.22 to 0.60) |
| 80 years or over | 5544 / 8267 | 67.1 (65.2 to 69.0) | 0.43 (0.36 to 0.52) | 389 / 5764 | 6.7 (5.5 to 8.0) | 0.13 (0.08 to 0.21) |
|  |  |  |  |  |  |  |
| Sex |  |  |  |  |  |  |
| Female | 5810 / 8285 | 70.1 (68.3 to 72.2) | ref | 465 / 5229 | 8.9 (7.7 to 10.5) | ref |
| Male | 9001 / 12,700 | 70.9 (69.3 to 72.4) | 1.03 (0.93 to 1.15) | 722 / 7045 | 10.2 (8.9 to 11.6) | 1.44 (1.08 to 1.91) |
|  |  |  |  |  |  |  |
| Ethnicity |  |  |  |  |  |  |
| White | 12,053 / 17,342 | 69.5 (68.1 to 70.9) | ref | 934 / 10,450 | 8.9 (8.0 to 10.0) | ref |
| Asian | 1689 / 2241 | 75.4 (71.8 to 78.6) | 1.46 (1.22 to 1.75) | 142 / 1030 | 13.8 (9.9 to 18.2) | 2.68 (1.56 to 4.59) |
| Black | 852 / 1105 | 77.1 (71.5 to 81.5) | 1.66 (1.29 to 2.13) | 88 / 627 | 14.0 (9.1 to 20.0) | 2.28 (1.16 to 4.46) |
| Other | 116 / 151 | 76.8 (62.8 to 88.2) | 1.34 (0.72 to 2.52) | 9 / 103 | 8.7 (1.5 to 24.6) | 1.20 (0.21 to 6.85) |
| Unknown | 101 / 146 | 69.2 (54.2 to 82.8) | 0.92 (0.49 to 1.74) | 14 / 64 | 21.9 (5.9 to 42.6) | 11.8 (1.70 to 82.1) |
|  |  |  |  |  |  |  |
| Patient-level IMD quintile |  |  |  |  |  |  |
| 1 (least deprived) | 2197 / 3238 | 67.9 (65.1 to 70.9) | ref | 181 / 1900 | 9.5 (7.2 to 12.1) | ref |
| 2 | 2617 / 3806 | 68.8 (65.7 to 71.9) | 1.09 (0.91 to 1.30) | 222 / 2267 | 9.8 (7.7 to 12.1) | 1.12 (0.70 to 1.81) |
| 3 | 2784 / 3936 | 70.7 (68.1 to 73.5) | 1.21 (1.01 to 1.45) | >230 / 2343 ^c^ | >9.0 (7.7 to 12.5) | 1.03 (0.63 to 1.66) |
| 4 | 3376 / 4670 | 72.3 (69.9 to 74.8) | 1.27 (1.06 to 1.51) | 246 / 2674 | 9.2 (7.2 to 11.5) | 0.85 (0.53 to 1.35) |
| 5 (most deprived) | 3830 / 5326 | 71.9 (70.6 to 73.2) | 1.16 (0.97 to 1.38) | 303 / 3084 | 9.8 (8.7 to 11.0) | 0.77 (0.49 to 1.22) |
| Unknown | 7 / 9 | 77.8 (49.1 to 92.7) | 2.84 (0.21 to 38.3) | <5 / 6 | - | 1.58 (0.01 to 448.2) |
|  |  |  |  |  |  |  |
| CKD ^d^ |  |  |  |  |  |  |
| No | 4223 / 5546 | 76.1 (74.0 to 78.2) | ref | 336 / 2630 | 12.8 (10.4 to 15.5) | ref |
| Yes | 10,588 / 15,439 | 68.6 (67.1 to 70.0) | 0.55 (0.49 to 0.62) | 851 / 9644 | 8.8 (7.8 to 10.0) | 0.69 (0.49 to 0.96) |
|  |  |  |  |  |  |  |
| Diabetes ^d^ |  |  |  |  |  |  |
| No | 634 / 994 | 63.8 (58.2 to 69.4) | ref | 83 / 967 | 8.6 (5.5 to 12.2) | ref |
| Yes | 14,177 / 19,991 | 70.9 (69.7 to 72.2) | 1.53 (1.21 to 1.94) | 1104 / 11,307 | 9.8 (8.7 to 10.8) | 1.31 (0.77 to 2.24) |
|  |  |  |  |  |  |  |
| Heart failure ^d^ |  |  |  |  |  |  |
| No | 8195 / 11,698 | 70.1 (68.5 to 71.7) | ref | 557 / 7156 | 7.8 (6.6 to 9.0) | ref |
| Yes | 6616 / 9287 | 71.2 (69.4 to 73.0) | 1.06 (0.96 to 1.17) | 630 / 5118 | 12.3 (10.6 to 14.2) | 4.29 (3.22 to 5.70) |
|  |  |  |  |  |  |  |
| Myocardial infarction ^d^ |  |  |  |  |  |  |
| No | 10,055 / 14,491 | 69.4 (67.8 to 70.8) | ref | 753 / 8935 | 8.4 (7.3 to 9.5) | ref |
| Yes | 4756 / 6494 | 73.2 (71.3 to 75.3) | 1.29 (1.16 to 1.45) | 434 / 3339 | 13.0 (10.9 to 15.3) | 3.60 (2.64 to 4.92) |
|  |  |  |  |  |  |  |
| Major surgery during AKI episode |  |  |  |  |  |  |
| No | 14,253 / 20,202 | 70.6 (69.3 to 71.8) | ref | 1148 / 12,005 | 9.6 (8.6 to 10.5) | ref |
| Yes | 558 / 783 | 71.3 (65.9 to 76.8) | 0.97 (0.76 to 1.25) | 39 / 269 | 14.5 (8.1 to 22.0) | 1.82 (0.81 to 4.06) |
|  |  |  |  |  |  |  |
| Sepsis during AKI episode |  |  |  |  |  |  |
| No | 12,925 / 18,124 | 71.3 (70.0 to 72.6) | ref | 1048 / 10,287 | 10.2 (9.0 to 11.3) | ref |
| Yes | 1886 / 2861 | 65.9 (62.7 to 68.9) | 0.66 (0.58 to 0.76) | 139 / 1987 | 7.0 (5.2 to 8.9) | 0.57 (0.41 to 0.81) |
|  |  |  |  |  |  |  |
| Primary care coded AKI within 30 days of discharge | |  |  |  |  |  |
| No | 11,757 / 16,398 | 71.7 (70.2 to 73.1) | ref | 988 / 9933 | 9.9 (8.9 to 11.1) | ref |
| Yes | 3054 / 4587 | 66.6 (64.0 to 69.2) | 0.71 (0.63 to 0.80) | 199 / 2341 | 8.5 (6.5 to 10.6) | 0.76 (0.55 to 1.05) |
|  |  |  |  |  |  |  |
| Face-to-face contact with a doctor, nurse, or pharmacist from the general practice within 30 days after discharge | |  |  |  |  |  |
| No | 6015 / 8495 | 70.8 (69.0 to 72.4) | ref | 483 / 5869 | 8.2 (6.8 to 9.7) | ref |
| Yes | 8796 / 12,490 | 70.4 (68.8 to 71.9) | 0.94 (0.85 to 1.04) | 704 / 6405 | 11.0 (9.6 to 12.5) | 1.81 (1.41 to 2.32) |
|  |  |  |  |  |  |  |
| Outpatient contact with General Surgery, Endocrinology and Diabetes, Cardiology, or Renal Medicine within 30 days after discharge ^e^ | |  |  |  |  |  |
| No | 10,804 / 15,290 | 70.7 (69.3 to 72.0) | ref | 786 / 9207 | 8.5 (7.5 to 9.6) | ref |
| Yes | 2246 / 3255 | 69.0 (66.0 to 71.9) | 0.84 (0.73 to 0.96) | 221 / 1647 | 13.4 (10.7 to 16.6) | 2.07 (1.35 to 3.19) |
|  |  |  |  |  |  |  |
| Outpatient contact with General Surgery, Endocrinology and Diabetes, Cardiology, or Renal Medicine within 90 days after discharge ^e^ | |  |  |  |  |  |
| No | 7931 / 11,251 | 70.5 (68.9 to 72.1) | ref | 538 / 7078 | 7.6 (6.5 to 8.7) | ref |
| Yes | 4711 / 6733 | 70.0 (67.9 to 72.1) | 0.88 (0.78 to 0.98) | 446 / 3425 | 13.0 (10.9 to 15.2) | 2.64 (1.96 to 3.54) |
|  |  |  |  |  |  |  |
| Period of discharge |  |  |  |  |  |  |
| 1^st^ January 2017 to 31^st^ March 2018 | 4139 / 5970 | 69.3 (67.0 to 71.5) | ref | 305 / 3432 | 8.9 (7.3 to 10.9) | ref |
| 1^st^ April 2018 to 31^st^ March 2019 | 3420 / 4864 | 70.3 (68.0 to 72.7) | 1.49 (1.09 to 2.04) | 287 / 2928 | 9.8 (7.8 to 11.7) | 1.80 (0.81 to 4.03) |
| 1^st^ April 2019 to 31^st^ March 2020 | 3835 / 5384 | 71.2 (68.8 to 73.6) | 2.28 (1.33 to 3.92) | 288 / 3137 | 9.2 (7.5 to 11.1) | 1.89 (0.47 to 7.53) |
| 1^st^ April 2020 to 31^st^ March 2021 | 3417 / 4767 | 71.7 (69.3 to 74.1) | 3.11 (1.43 to 6.77) | 307 / 2777 | 11.1 (9.1 to 13.7) | 2.83 (0.39 to 20.8) |

^a^ 95% CI of percentages calculated using bootstrap with sampling at the patient and practice levels, except for IMD quintiles 5 and unknown for which sampling was done at the practice level only.

^b^ ORs adjusted for age, sex, month count (1-51) and calendar month (1-12) of discharge, and practice and patient-level clustering.

^c^ Exact values of numerator and percentage not reported to prevent disclosure of numerator (<5) for unknown IMD quintile.

^d^ Recorded at any time up to and including the date of discharge.

^e^ Outpatient data only available up to 31^st^ Oct 2020.

## **Table S9. Avoidance of prescription of NSAID within 90 days after discharge, by demographic and clinical characteristics**

|  | Number of AKI episodes meeting indicator criteria / Number of AKI episodes eligible for inclusion | Percentage of AKI episodes meeting indicator criteria (95% CI) ^a^ | Odds ratio (OR) ^b^ |
| --- | --- | --- | --- |
|  |  |  |  |
| Overall | 213,753 / 219,714 | 97.3 (97.1 to 97.4) | N/A |
|  |  |  |  |
| Age |  |  |  |
| Less than 60 years | 38,357 / 40,322 | 95.1 (94.7 to 95.6) | ref |
| 60-79 years | 88,485 / 91,370 | 96.8 (96.6 to 97.1) | 2.42 (2.13 to 2.75) |
| 80 years or over | 86,911 / 88,022 | 98.7 (98.6 to 98.9) | 14.7 (11.7 to 18.4) |
|  |  |  |  |
| Sex |  |  |  |
| Female | 100,188 / 103,255 | 97.0 (96.8 to 97.2) | ref |
| Male | 113,565 / 116,459 | 97.5 (97.3 to 97.7) | 1.43 (1.30 to 1.58) |
|  |  |  |  |
| Ethnicity |  |  |  |
| White | 191,568 / 196,893 | 97.3 (97.1 to 97.5) | ref |
| Asian | 11,052 / 11,360 | 97.3 (96.7 to 97.8) | 1.22 (0.97 to 1.53) |
| Black | 7924 / 8134 | 97.4 (96.7 to 98.1) | 1.51 (1.15 to 2.00) |
| Other | 1407 / 1457 | 96.6 (94.7 to 98.0) | 1.22 (0.71 to 2.09) |
| Unknown | 1802 / 1870 | 96.4 (93.3 to 98.2) | 0.93 (0.56 to 1.54) |
|  |  |  |  |
| Patient-level IMD quintile |  |  |  |
| 1 (least deprived) | 37,648 / 38,570 | 97.6 (97.3 to 97.9) | ref |
| 2 | 40,909 / 42,042 | 97.3 (97.0 to 97.6) | 0.91 (0.77 to 1.08) |
| 3 | 40,931 / 42,032 | 97.4 (97.0 to 97.7) | 1.00 (0.85 to 1.19) |
| 4 | 44,518 / 45,799 | 97.2 (96.9 to 97.5) | 0.99 (0.83 to 1.17) |
| 5 (most deprived) | 48,840 / 50,359 | 97.0 (96.7 to 97.3) | 0.94 (0.79 to 1.12) |
| Unknown | 907 / 912 | 99.5 (94.3 to 100) | 6.96 (1.70 to 28.5) |
|  |  |  |  |
| CKD ^c^ |  |  |  |
| No | 100,427 / 104,313 | 96.3 (96.0 to 96.5) | ref |
| Yes | 113,326 / 115,401 | 98.2 (98.0 to 98.4) | 2.45 (2.19 to 2.75) |
|  |  |  |  |
| Diabetes ^c^ |  |  |  |
| No | 132,430 / 136,509 | 97.0 (96.8 to 97.2) | ref |
| Yes | 81,323 / 83,205 | 97.7 (97.5 to 97.9) | 1.41 (1.27 to 1.57) |
|  |  |  |  |
| Heart failure ^c^ |  |  |  |
| No | 145,857 / 150,972 | 96.6 (96.4 to 96.8) | ref |
| Yes | 67,896 / 68,742 | 98.8 (98.6 to 98.9) | 4.25 (3.64 to 4.95) |
|  |  |  |  |
| Myocardial infarction ^c^ |  |  |  |
| No | 168,738 / 174,079 | 96.9 (96.8 to 97.1) | ref |
| Yes | 45,015 / 45,635 | 98.6 (98.4 to 98.8) | 2.89 (2.46 to 3.38) |
|  |  |  |  |
| Major surgery during AKI episode |  |  |  |
| No | 204,307 / 209,897 | 97.3 (97.2 to 97.5) | ref |
| Yes | 9446 / 9817 | 96.2 (95.6 to 96.8) | 0.60 (0.50 to 0.73) |
|  |  |  |  |
| Sepsis during AKI episode |  |  |  |
| No | 181,669 / 186,646 | 97.3 (97.2 to 97.5) | ref |
| Yes | 32,084 / 33,068 | 97.0 (96.7 to 97.3) | 0.87 (0.77 to 0.97) |
|  |  |  |  |
| Primary care coded AKI within 30 days of discharge | |  |  |
| No | 171,948 / 176,907 | 97.2 (97.0 to 97.4) | ref |
| Yes | 41,805 / 42,807 | 97.7 (97.4 to 97.9) | 1.23 (1.09 to 1.38) |
|  |  |  |  |
| Face-to-face contact with a doctor, nurse, or pharmacist from the general practice within 30 days after discharge | |  |  |
| No | 102,835 / 105,226 | 97.7 (97.5 to 97.9) | ref |
| Yes | 110,918 / 114,488 | 96.9 (96.6 to 97.1) | 0.60 (0.55 to 0.66) |
|  |  |  |  |
| Outpatient contact with General Surgery, Endocrinology and Diabetes, Cardiology, or Renal Medicine within 30 days after discharge ^d^ | |  |  |
| No | 165,696 / 170,520 | 97.2 (97.0 to 97.3) | ref |
| Yes | 23,020 / 23,503 | 97.9 (97.6 to 98.3) | 1.74 (1.48 to 2.04) |
|  |  |  |  |
| Outpatient contact with General Surgery, Endocrinology and Diabetes, Cardiology, or Renal Medicine within 90 days after discharge ^d^ | |  |  |
| No | 133,069 / 137,100 | 97.1 (96.9 to 97.2) | ref |
| Yes | 49,272 / 50,358 | 97.8 (97.6 to 98.1) | 1.71 (1.52 to 1.93) |
|  |  |  |  |
| Period of discharge |  |  |  |
| 1^st^ January 2017 to 31^st^ March 2018 | 61,285 / 63,074 | 97.2 (96.9 to 97.4) | ref |
| 1^st^ April 2018 to 31^st^ March 2019 | 51,502 / 53,027 | 97.1 (96.8 to 97.4) | 1.02 (0.77 to 1.36) |
| 1^st^ April 2019 to 31^st^ March 2020 | 52,495 / 53,903 | 97.4 (97.1 to 97.6) | 1.22 (0.74 to 1.99) |
| 1^st^ April 2020 to 31^st^ March 2021 | 48,471 / 49,710 | 97.5 (97.2 to 97.8) | 1.40 (0.69 to 2.85) |

^a^ 95% CI of percentages calculated using bootstrap with sampling at the patient and practice levels.

^b^ ORs adjusted for age, sex, month count (1-51) and calendar month (1-12) of discharge, and practice and patient-level clustering.

^c^ Recorded at any time up to and including the date of discharge.

^d^ Outpatient data only available up to 31^st^ Oct 2020.

## **Table S10. Reliability of post-AKI care indicators**

| Post-AKI care indicators | Number of practices | Number of patients eligible for inclusion | Number of AKI episodes eligible for inclusion | Median number of patients in a practice eligible for inclusion | Median number of episodes in a practice amongst patients eligible for inclusion | Reliability of a practice with median number of eligible patients and median number of AKI episodes | % practices with reliability>0.7 |
| --- | --- | --- | --- | --- | --- | --- | --- |
|  |  |  |  |  |  |  |  |
| *Recording of AKI in primary care* |  |  |  |  |  |  |  |
| Coding of AKI in primary care record within 30 days after discharge | 1470 | 185,328 | 245,947 | 105 | 137.5 | 0.97 | 99.3 |
| Coding of AKI in primary care record within 90 days after discharge | 1470 | 167,448 | 219,714 | 95 | 124 | 0.97 | 99.0 |
|  |  |  |  |  |  |  |  |
| *Primary care clinical contacts* *within 30 days after discharge* |  |  |  |  |  |  |  |
| Face-to-face contact with a doctor, nurse, or pharmacist at the general practice | 1470 | 185,328 | 245,947 | 105 | 137.5 | 0.92 | 95.9 |
| Face-to-face or telephone contact with a doctor, nurse, or pharmacist at the general practice | 1470 | 185,328 | 245,947 | 105 | 137.5 | 0.90 | 94.0 |
|  |  |  |  |  |  |  |  |
| *Monitoring of kidney health status and blood pressure at 90±30 days after discharge* |  |  |  |  |  |  |  |
| Creatinine measurement | 1469 | 161,388 | 210,792 | 91 | 118 | 0.70 | 49.6 |
| ACR measurement | 1469 | 161,388 | 210,792 | 91 | 118 | 0.84 | 83.8 |
| Blood pressure measurement | 1469 | 161,388 | 210,792 | 91 | 118 | 0.73 | 58.6 |
| Coding of *de novo* CKD | 1341 | 7506 | 8489 | 4 | 5 | 0.47 | 12.2 |
|  |  |  |  |  |  |  |  |
| *Monitoring of kidney health status and blood pressure at 365±90 days after discharge* |  |  |  |  |  |  |  |
| Creatinine measurement | 1462 | 124,579 | 157,712 | 71 | 89 | 0.69 | 46.7 |
| ACR measurement | 1462 | 124,579 | 157,712 | 71 | 89 | 0.79 | 71.8 |
| Blood pressure measurement | 1462 | 124,579 | 157,712 | 71 | 89 | 0.70 | 51.5 |
|  |  |  |  |  |  |  |  |

| *Guideline indicated prescribing within 90 days after discharge* |  |  |  |  |  |  |  |
| --- | --- | --- | --- | --- | --- | --- | --- |
| RAASi continuation | 1421 | 16,192 | 20,985 | 9 | 12 | 0.20 | 0.1 |
| RAASi initiation | 1373 | 8947 | 12,274 | 5 | 7 | 0.0 | 0.0 |
| Avoidance of prescribing NSAID | 1470 | 167,448 | 219,714 | 95 | 124 | 0.84 | 82.7 |

## **Table S11. Number of practices with 0% adherence to indicators related to** **creatinine, ACR, and blood pressure measurements**

| Post-AKI care indicators | Total number of practices | Number of practices with 0% adherence to indicators (%) |
| --- | --- | --- |
| *Creatinine measurement* |  |  |
| At 90±30 days after discharge | 1469 | 5 (0.3) |
| At 365±90 days after discharge | 1462 | <5 (<0.4) |
|  |  |  |
| *ACR measurement* |  |  |
| At 90±30 days after discharge | 1469 | 156 (10.6) |
| At 365±90 days after discharge | 1462 | 47 (3.2) |
|  |  |  |
| *Blood pressure measurement* |  |  |
| At 90±30 days after discharge | 1469 | 7 (0.5) |
| At 365±90 days after discharge | 1462 | <5 (<0.4) |

## **Table S12. Number of practices for which there was 0% adherence for 0, 1, 2 or more blood and urine measurement indicators (creatinine, ACR, or blood pressure)**

| Number of indicators with 0% adherence | Number of practices (%) | |
| --- | --- | --- |
|  | 90±30 days after discharge ^a^ | 365±90 days after discharge ^b^ |
| 0 | 1311 (89.2) | 1415 (96.8) |
| 1 | 151 (10.3) | 42 (2.9) |
| 2 or more | 7 (0.48) | 5 (0.34) |
|  |  |  |

^a^ Percentages of 1469 practices.

^b^ Percentages of 1462 practices.

## **Figure S1. Delineation of study cohort of patients**

## **Figure S2. Odds ratios (95% CI) of AKI episodes recorded in primary care records within 90 days after discharge, by demographics, socioeconomics, and clinical characteristics**

- ORs adjusted for age, sex, month count (1-51) and calendar month (1-12) of discharge, and practice and patient-level clustering.
- ORs for unknown IMD quintile not shown in figure because of small number of episodes.
- CKD (excluding stage 5), diabetes, heart failure, and myocardial infarction: recorded at any time up to and including the date of discharge.
- Major surgery and sepsis: recorded during AKI episode.
- Face-to face contact at general practice within 30 days of discharge: with doctor, nurse, or pharmacist.
- Outpatient contacts with General Surgery, Endocrinology and Diabetes, Cardiology, or Renal Medicine. Data available up to 31^st^ Oct 2020.

## **Figure S3. Odds ratios (95% CI) of face-to-face contact with a doctor, nurse, or pharmacist at the general practice within 30 days after discharge, by demographics, socioeconomics, and clinical characteristics**

- ORs adjusted for age, sex, month count (1-51) and calendar month (1-12) of discharge, and practice and patient-level clustering.
- ORs for unknown IMD quintile not shown in figure because of small number of episodes.
- CKD (excluding stage 5), diabetes, heart failure, and myocardial infarction: recorded at any time up to and including the date of discharge.
- Major surgery and sepsis: recorded during AKI episode.
- Outpatient contacts with General Surgery, Endocrinology and Diabetes, Cardiology, or Renal Medicine. Data available up to 31^st^ Oct 2020.

## **Figure S4. Odds ratios (95% CI) of face-to-face or telephone contact with a doctor, nurse, or pharmacist at the general practice within 30 days after discharge, by demographics, socioeconomics, and clinical characteristics**

- ORs adjusted for age, sex, month count (1-51) and calendar month (1-12) of discharge, and practice and patient-level clustering.
- ORs for unknown IMD quintile not shown in figure because of small number of episodes.
- CKD (excluding stage 5), diabetes, heart failure, and myocardial infarction: recorded at any time up to and including the date of discharge.
- Major surgery and sepsis: recorded during AKI episode.
- Outpatient contacts with General Surgery, Endocrinology and Diabetes, Cardiology, or Renal Medicine. Data available up to 31^st^ Oct 2020.

## **Figure S5. Odds ratios (95% CI) of creatinine measurement at 90±30 days after discharge, by demographics, socioeconomics, and clinical characteristics**

- ORs adjusted for age, sex, month count (1-51) and calendar month (1-12) of discharge, and practice and patient-level clustering.
- ORs for unknown IMD quintile not shown in figure because of small number of episodes.
- CKD (excluding stage 5), diabetes, heart failure, and myocardial infarction: recorded at any time up to and including the date of discharge.
- Major surgery and sepsis: recorded during AKI episode.
- Face-to face contact at general practice within 30 days of discharge: with doctor, nurse, or pharmacist.
- Outpatient contacts with General Surgery, Endocrinology and Diabetes, Cardiology, or Renal Medicine. Data available up to 31^st^ Oct 2020.

## **Figure S6. Odds ratios (95% CI) of creatinine measurement at 365±90 days after discharge, by demographics, socioeconomics, and clinical characteristics**

- ORs adjusted for age, sex, month count (1-51) and calendar month (1-12) of discharge, and practice and patient-level clustering.
- ORs for unknown IMD quintile not shown in figure because of small number of episodes.
- CKD (excluding stage 5), diabetes, heart failure, and myocardial infarction: recorded at any time up to and including the date of discharge.
- Major surgery and sepsis: recorded during AKI episode.
- Face-to face contact at general practice within 30 days of discharge: with doctor, nurse, or pharmacist.
- Outpatient contacts with General Surgery, Endocrinology and Diabetes, Cardiology, or Renal Medicine. Data available up to 31^st^ Oct 2020.

## **Figure S7. Odds ratios (95% CI) of ACR measurement at 90±30 days after discharge, by demographics, socioeconomics, and clinical characteristics**

- ORs adjusted for age, sex, month count (1-51) and calendar month (1-12) of discharge, and practice and patient-level clustering.
- ORs for unknown IMD quintile not shown in figure because of small number of episodes.
- CKD (excluding stage 5), diabetes, heart failure, and myocardial infarction: recorded at any time up to and including the date of discharge.
- Major surgery and sepsis: recorded during AKI episode.
- Face-to face contact at general practice within 30 days of discharge: with doctor, nurse, or pharmacist.
- Outpatient contacts with General Surgery, Endocrinology and Diabetes, Cardiology, or Renal Medicine. Data available up to 31^st^ Oct 2020.

## **Figure S8. Odds ratios (95% CI) of ACR measurement at 365±90 days after discharge, by demographics, socioeconomics, and clinical characteristics**

- ORs adjusted for age, sex, month count (1-51) and calendar month (1-12) of discharge, and practice and patient-level clustering, except for face-to-face contact at general practice within 30 days of discharge. For this model, no practice-level clustering was adjusted for because of difficulties in running the model when both practice and patient-level clustering were included.
- IMD quintile unknown was excluded from the modelling because of difficulties in running the model when this group was included.
- CKD (excluding stage 5), diabetes, heart failure, and myocardial infarction: recorded at any time up to and including the date of discharge.
- Major surgery and sepsis: recorded during AKI episode.
- Face-to face contact at general practice within 30 days of discharge: with doctor, nurse, or pharmacist.
- Outpatient contacts with General Surgery, Endocrinology and Diabetes, Cardiology, or Renal Medicine. Data available up to 31^st^ Oct 2020.

## **Figure S9. Odds ratios (95% CI) of blood pressure measurement at 90±30 days after discharge, by demographics, socioeconomics, and clinical characteristics**

- ORs adjusted for age, sex, month count (1-51) and calendar month (1-12) of discharge, and practice and patient-level clustering.
- ORs for unknown IMD quintile not shown in figure because of small number of episodes.
- CKD (excluding stage 5), diabetes, heart failure, and myocardial infarction: recorded at any time up to and including the date of discharge.
- Major surgery and sepsis: recorded during AKI episode.
- Face-to face contact at general practice within 30 days of discharge: with doctor, nurse, or pharmacist.
- Outpatient contacts with General Surgery, Endocrinology and Diabetes, Cardiology, or Renal Medicine. Data available up to 31^st^ Oct 2020.

## **Figure S10. Odds ratios (95% CI) of blood pressure measurement at 365±90 days after discharge, by demographics, socioeconomics, and clinical characteristics**

- ORs adjusted for age, sex, month count (1-51) and calendar month (1-12) of discharge, and practice and patient-level clustering.
- ORs for unknown IMD quintile not shown in figure because of small number of episodes.
- CKD (excluding stage 5), diabetes, heart failure, and myocardial infarction: recorded at any time up to and including the date of discharge.
- Major surgery and sepsis: recorded during AKI episode.
- Face-to face contact at general practice within 30 days of discharge: with doctor, nurse, or pharmacist.
- Outpatient contacts with General Surgery, Endocrinology and Diabetes, Cardiology, or Renal Medicine. Data available up to 31^st^ Oct 2020.

## **Figure S11. Odds ratios (95% CI) of coding of *de novo* CKD in primary care records at 90±30 days after discharge, by demographics, socioeconomics, and clinical characteristics**

- ORs adjusted for age, sex, month count (1-51) and calendar month (1-12) of discharge, and practice and patient-level clustering.
- ORs for unknown IMD quintile not shown in figure because of small number of episodes.
- CKD (excluding stage 5), diabetes, heart failure, and myocardial infarction: recorded at any time up to and including the date of discharge.
- Major surgery and sepsis: recorded during AKI episode.
- Face-to face contact at general practice within 30 days of discharge: with doctor, nurse, or pharmacist.
- Outpatient contacts with General Surgery, Endocrinology and Diabetes, Cardiology, or Renal Medicine. Data available up to 31^st^ Oct 2020.

## **Figure S12. Odds ratios (95% CI) of RAASi continuation within 90 days after discharge, by demographics, socioeconomics, and clinical characteristics**

- ORs adjusted for age, sex, month count (1-51) and calendar month (1-12) of discharge, and practice and patient-level clustering.
- ORs for unknown IMD quintile not shown in figure because of small number of episodes.
- CKD (excluding stage 5), diabetes, heart failure, and myocardial infarction: recorded at any time up to and including the date of discharge.
- Major surgery and sepsis: recorded during AKI episode.
- Face-to face contact at general practice within 30 days of discharge: with doctor, nurse, or pharmacist.
- Outpatient contacts with General Surgery, Endocrinology and Diabetes, Cardiology, or Renal Medicine. Data available up to 31^st^ Oct 2020.
-

## **Figure S13. Odds ratios (95% CI) of RAASi initiation within 90 days after discharge, by demographics, socioeconomics, and clinical characteristics**

- ORs adjusted for age, sex, month count (1-51) and calendar month (1-12) of discharge, and practice and patient-level clustering.
- ORs for unknown IMD quintile and unknown ethnicity not shown in figure because of small number of episodes.
- CKD (excluding stage 5), diabetes, heart failure, and myocardial infarction: recorded at any time up to and including the date of discharge.
- Major surgery and sepsis: recorded during AKI episode.
- Face-to face contact at general practice within 30 days of discharge: with doctor, nurse, or pharmacist.
- Outpatient contacts with General Surgery, Endocrinology and Diabetes, Cardiology, or Renal Medicine. Data available up to 31^st^ Oct 2020.

## **Figure S14. Odds ratios (95% CI) of avoidance of prescribing of NSAID within 90 days after discharge, by demographics, socioeconomics, and clinical characteristics**

- ORs adjusted for age, sex, month count (1-51) and calendar month (1-12) of discharge, and practice and patient-level clustering.
- ORs for unknown IMD quintile not shown in figure because of small number of episodes.
- CKD (excluding stage 5), diabetes, heart failure, and myocardial infarction: recorded at any time up to and including the date of discharge.
- Major surgery and sepsis: recorded during AKI episode.
- Face-to face contact at general practice within 30 days of discharge: with doctor, nurse, or pharmacist.
- Outpatient contacts with General Surgery, Endocrinology and Diabetes, Cardiology, or Renal Medicine. Data available up to 31^st^ Oct 2020.

| Creatinine check 365±90 days (R=0.69)   | Coding of de novo CKD (R=0.47)   |
| --- | --- |
| RAASi continuation within 90 days (R=0.20)   | RAASI initiation within 90 days (R=0.0)   |

## **Figure S15. Adherence to post-AKI care indicators with reliability <0.7 by practice**

Vertical axis shows percentage of AKI episodes meeting indicator criteria. Horizontal axis shows practice ordered by increasing percentage of episodes meeting indicator criteria. R value reported in brackets for each indicator is the reliability of a practice with median number of eligible patients and median number of AKI episodes.

## **Clinical codes used to identify AKI in CPRD Aurum**

| medcodeid | readcode | snomedctconceptid | snomedctdescriptionid | term |
| --- | --- | --- | --- | --- |
| 2732241000006113 |  | 14669001 | 24910011 | Acute renal failure syndrome |
| 2732281000006119 |  | 14669001 | 2204201000000112 | AKI - acute kidney injury |
| 2775871000006110 |  | 17380002 | 29387012 | Failed attempted abortion with acute renal failure |
| 2775881000006113 |  | 17380002 | 29388019 | Failed attempted abortion with renal shutdown |
| 2775861000006115 |  | 17380002 | 2967097017 | Failed attempted termination of pregnancy with acute renal failure |
| 2878291000006115 |  | 23697004 | 39796018 | Renal failure following crushing injury |
| 2878351000006115 |  | 23697004 | 481691015 | Myoglobinuric acute renal failure |
| 3039991000006110 |  | 33561005 | 56004010 | Illegal abortion with acute renal failure |
| 3040001000006113 |  | 33561005 | 56005011 | Illegal abortion with renal shutdown |
| 3039981000006112 |  | 33561005 | 2966760010 | Illegal termination of pregnancy with acute renal failure |
| 3071071000006113 |  | 35455006 | 59155017 | Acute renal tubular necrosis |
| 3083861000006118 |  | 36225005 | 60449014 | Acute renal failure due to procedure |
| 3083871000006113 |  | 36225005 | 60453011 | Acute renal insufficiency due to procedure |
| 3348711000006113 |  | 52342006 | 87107017 | Legal abortion with acute renal failure |
| 3348721000006117 |  | 52342006 | 87108010 | Legal abortion with renal shutdown |
| 3348701000006110 |  | 52342006 | 2966730017 | Legal termination of pregnancy with acute renal failure |
| 3433901000006114 |  | 57469000 | 95574019 | Spontaneous abortion with acute renal failure |
| 3433911000006112 |  | 57469000 | 95575018 | Spontaneous abortion with renal shutdown |
| 3433921000006116 |  | 57469000 | 2966335013 | Miscarriage with renal shutdown |
| 3433891000006110 |  | 57469000 | 2966367011 | Miscarriage with acute renal failure |
| 3669681000006116 |  | 71909003 | 119482017 | Acute renal failure following molar AND/OR ectopic pregnancy |
| 3669691000006118 |  | 71909003 | 119485015 | Renal shutdown following molar AND/OR ectopic pregnancy |
| 5094041000006117 |  | 236424009 | 354408015 | Acute renal impairment |
| 5094071000006113 |  | 236428007 | 354412014 | Nephrotoxic acute renal failure |
| 5094141000006118 |  | 236433006 | 3037716017 | Acute on chronic renal failure |
| 5511151000006119 |  | 269257004 | 403057012 | Acute renal failure due to crush syndrome |
| 5847371000006111 |  | 298015003 | 438612016 | Acute renal papillary necrosis with renal failure |
| 5966771000006111 |  | 307309005 | 450541012 | Transient acute renal failure |
| 6992471000006118 |  | 422593004 | 2649446019 | Acute renal failure due to angiotensin-converting-enzyme inhibitor |
| 6992481000006115 |  | 422593004 | 3290589015 | Acute renal failure caused by ACE inhibitor |
| 6992491000006117 |  | 422593004 | 3290639019 | Acute renal failure caused by angiotensin-converting-enzyme inhibitor |
| 7009281000006118 |  | 423533009 | 2645797016 | Acute renal failure due to ischemia |
| 7009271000006116 |  | 423533009 | 2645798014 | Acute renal failure due to ischaemia |
| 7009291000006115 |  | 423533009 | 3035519014 | Acute ischemic renal failure |
| 7009301000006119 |  | 423533009 | 3035569018 | Acute ischaemic renal failure |
| 7100101000006115 |  | 429224003 | 2692740012 | Acute renal failure due to acute cortical necrosis |
| 7122851000006116 |  | 430535006 | 2765843017 | Acute renal failure with oliguria |
| 7213941000006111 |  | 438783006 | 2792921014 | Acute postoperative renal failure |
| 7297961000006117 |  | 444794000 | 2873011015 | Acute necrosis of cortex of kidney |
| 12704041000006118 |  | 444794000 | 2873012010 | Acute renal cortical necrosis |
| 7498381000006114 |  | 609455009 | 2966764018 | Termination of pregnancy complicated by acute renal failure |
| 7498371000006111 |  | 609455009 | 2966971018 | Induced termination of pregnancy complicated by acute renal failure |
| 7498751000006117 |  | 609472002 | 2967117015 | Induced termination of pregnancy complicated by acute renal failure with oliguria |
| 7827231000006119 |  | 722095005 | 3330670013 | Acute kidney injury due to circulatory failure |
| 7827241000006112 |  | 722096006 | 3330673010 | Acute kidney injury due to hypovolaemia |
| 7827251000006114 |  | 722096006 | 3330774012 | Acute kidney injury due to hypovolemia |
| 7829401000006116 |  | 722278006 | 3331407014 | Acute kidney injury due to sepsis |
| 7841561000006115 |  | 723189000 | 3334973015 | Acute renal insufficiency |
| 7879771000006116 |  | 726541005 | 3452123014 | Acute kidney injury due to trauma |
| 7879781000006118 |  | 726541005 | 3452124015 | AKI (acute kidney injury) due to trauma |
| 7954311000006119 |  | 733137002 | 3498837018 | Acute kidney failure stage 1 |
| 7954321000006110 |  | 733137002 | 3498838011 | Acute renal failure stage 1 |
| 7954341000006115 |  | 733138007 | 3498840018 | Acute renal failure stage 2 |
| 7954331000006113 |  | 733138007 | 3498842014 | Acute kidney failure stage 2 |
| 7954361000006116 |  | 733139004 | 3498843016 | Acute renal failure stage 3 |
| 7954351000006118 |  | 733139004 | 3498845011 | Acute kidney failure stage 3 |
| 7961041000006113 |  | 733839001 | 3474857017 | Postpartum acute renal failure |
| 13485221000006115 |  | 870589006 | 3970764011 | Acute kidney injury due to disease caused by 2019 novel coronavirus |
| 13485231000006117 |  | 870589006 | 3972966011 | Acute kidney injury due to disease caused by Severe acute respiratory syndrome coronavirus 2 |
| 13485211000006111 |  | 870589006 | 2838151000000110 | Acute kidney injury due to disease caused by SARS-CoV-2 (severe acute respiratory syndrome coronavirus 2) |
| 13485241000006110 |  | 870589006 | 2838161000000113 | Acute kidney injury due to disease caused by 2019-nCoV (novel coronavirus) |
| 8040771000006116 |  | 129561000119108 | 3013582015 | Prerenal renal failure |
| 8040781000006118 |  | 129561000119108 | 3013999013 | Pre-renal acute kidney injury |
| 8040791000006115 |  | 129561000119108 | 3014026017 | Acute renal failure |
| 8040811000006116 |  | 129721000119106 | 3012686019 | Acute renal failure on dialysis |
| 8043971000006111 |  | 140031000119103 | 3012709019 | Acute nontraumatic kidney injury |
| 8043981000006114 |  | 140031000119103 | 3491571011 | Nontraumatic AKI (acute kidney injury) |
| 8046301000006111 |  | 145681000119101 | 3012678019 | Acute renal failure due to tubular necrosis |
| 8046291000006110 |  | 145681000119101 | 2641381000000117 | Acute kidney injury due to tubular necrosis |
| 8120581000006115 |  | 368951000119105 | 3289780013 | Acute renal failure caused by contrast agent |
| 2185551000000111 | K046000 | 842511000000101 | 2185551000000111 | Acute renal failure induced by animal toxin |
| 2185591000000115 | K046100 | 842531000000109 | 2185591000000115 | Acute renal failure induced by plant toxin |
| 2185631000000115 | K047.00 | 842551000000102 | 2185631000000115 | Acute renal failure induced by heavy metal |
| 2191381000000114 | K04A.00 | 844741000000100 | 2191381000000114 | Acute renal failure induced by solvent |
| 8425491000006113 |  | 1048451000000103 | 2641181000000115 | Acute kidney injury due to acute tubular necrosis due to hypovolaemia |
| 8425511000006119 |  | 1048481000000109 | 2641241000000118 | Acute kidney injury due to acute tubular necrosis due to circulatory failure |
| 8425521000006110 |  | 1048491000000106 | 2641261000000117 | Acute kidney injury due to acute tubular necrosis due to sepsis |
| 8425531000006113 |  | 1048501000000100 | 2641281000000114 | Acute kidney injury due to acute tubular necrosis with histological evidence |
| 8425541000006115 |  | 1048511000000103 | 2641301000000110 | Acute kidney injury due to acute tubular necrosis due to hypovolaemia with histological evidence |
| 8425551000006118 |  | 1048521000000109 | 2641321000000118 | Acute kidney injury due to acute tubular necrosis due to sepsis with histological evidence |
| 8425561000006116 |  | 1048541000000102 | 2641361000000114 | Acute kidney injury due to acute tubular necrosis due to circulatory failure with histological evidence |
| 1726511000006111 |  | 1726511000006107 | 1726511000006111 | Acute-on-chronic renal failure |
| 1221119015 | K04..11 | 14669001 | 1221119015 | ARF - Acute renal failure |
| 460091000006111 | K04..00 | 14669001 | 2920634011 | Acute renal failure |
| 303915016 | K04y.00 | 14669001 | 2920634011 | Other acute renal failure |
| 303916015 | K04z.00 | 14669001 | 2920634011 | Acute renal failure NOS |
| 305191012 | Kyu2000 | 14669001 | 2920634011 | [X]Other acute renal failure |
| 2204191000000110 | K04..12 | 14669001 | 2204191000000110 | Acute kidney injury |
| 481692010 | SK08.00 | 23697004 | 481692010 | Acute renal failure due to rhabdomyolysis |
| 460151000006118 | K040.00 | 23697004 | 481694011 | Acute tubular necrosis |
| 481693017 | K040.11 | 35455006 | 59152019 | Acute tubular necrosis |
| 216921000006111 | L393100 | 200118004 | 307552013 | Post-delivery acute renal failure - delivered with p/n prob |
| 307552013 | L393200 | 200118004 | 307552013 | Post-delivery acute renal failure with postnatal problem |
| 2214211000000112 | K046.00 | 236428007 | 2214211000000112 | Acute renal failure induced by toxin |
| 354413016 | K043.00 | 236429004 | 354413016 | Acute drug-induced renal failure |
| 354417015 | K0E..00 | 236433006 | 354417015 | Acute-on-chronic renal failure |
| 354513019 | K0H..00 | 236503001 | 354513019 | Acute scleroderma renal crisis |
| 2645796013 | K043000 | 422593004 | 2645796013 | Acute renal failure due to ACE inhibitor |
| 1705781000006113 | K044.00 | 429489008 | 2694055011 | Acute renal failure due to obstruction |
| 460081000006113 | K041.00 | 444794000 | 2873011015 | Acute renal cortical necrosis |
| 305571011 | L093100 | 609455009 | 2966764018 | Acute renal failure following abortive pregnancy |
| 460121000006110 | L393.00 | 733839001 | 3474857017 | Acute renal failure following labour and delivery |
| 307550017 | L393000 | 733839001 | 3474857017 | Post-delivery acute renal failure unspecified |
| 307553015 | L393z00 | 733839001 | 3474857017 | Post-delivery acute renal failure NOS |
| 2180741000000113 | K043100 | 840371000000100 | 2180741000000113 | Acute renal failure induced by aminoglycoside |
| 2180861000000117 | K043200 | 840431000000109 | 2180861000000117 | Acute renal failure induced by cisplatin |
| 2180901000000112 | K043300 | 840451000000102 | 2180901000000112 | Acute renal failure induced by cyclosporin A |
| 1847641000006114 | K043400 | 840471000000106 | 2180941000000110 | Acute renal failure induced by non-steroidal anti-inflammatory drug |
| 2184271000000115 | K045.00 | 841961000000100 | 2184271000000115 | Acute renal failure due to non-traumatic rhabdomyolysis |
| 2191151000000117 | K048.00 | 844631000000104 | 2191151000000117 | Acute renal failure induced by poison |
| 2191301000000116 | K049.00 | 844701000000103 | 2191301000000116 | Acute renal failure induced by radiographic contrast media |
| 2198081000000118 | K04B.00 | 847851000000101 | 2198081000000118 | Acute renal failure due to traumatic rhabdomyolysis |
| 2206791000000119 | K04C.00 | 851931000000107 | 2206791000000119 | Acute kidney injury stage 1 |
| 2206811000000118 | K04D.00 | 851941000000103 | 2206811000000118 | Acute kidney injury stage 2 |
| 2206831000000114 | K04E.00 | 851951000000100 | 2206831000000114 | Acute kidney injury stage 3 |

## **Clinical codes used to identify serum creatinine measurements in CPRD Aurum**

| medcodeid | readcode | snomedctconceptid | snomedctdescriptionid | term |
| --- | --- | --- | --- | --- |
| 4217561000006115 |  | 113075003 | 186102016 | Creatinine measurement, serum |
| 4217571000006110 |  | 113075003 | 380389013 | Serum creatinine |
| 4217581000006113 |  | 113075003 | 128041000000119 | Serum creatinine level |
| 259050010 | 44J3000 | 166714005 | 259050010 | Serum creatinine abnormal |
| 259051014 | 44J3100 | 166715006 | 259051014 | Serum creatinine low |
| 259052019 | 44J3200 | 166716007 | 259052019 | Serum creatinine normal |
| 259053012 | 44J3300 | 166717003 | 259053012 | Serum creatinine raised |
| 6036311000006119 |  | 313817004 | 457918014 | Corrected plasma creatinine measurement |
| 6036321000006110 |  | 313817004 | 457919018 | Corrected plasma creatinine level |
| 6036331000006113 |  | 313817004 | 129701000000110 | Cor plasma creatinine level |
| 6036401000006114 |  | 313822004 | 457926018 | Corrected serum creatinine measurement |
| 6036411000006112 |  | 313822004 | 457927010 | Corrected serum creatinine level |
| 6036421000006116 |  | 313822004 | 132331000000113 | Cor serum creatinine level |
| 6039721000006117 |  | 313936008 | 458156012 | Plasma creatinine level |
| 6039711000006113 |  | 313936008 | 458157015 | Plasma creatinine measurement |
| 6277971000006118 |  | 365757006 | 489163014 | Serum creatinine level - finding |
| 6277981000006115 |  | 365757006 | 489164015 | Serum creatinine level |
| 6277991000006117 |  | 365757006 | 2768710011 | Finding of serum creatinine level |
| 109761000006111 | 44J3.00 | 109761000006107 | 109761000006111 | Test request : Creatinine |
| 380389013 | 44J3.00 | 1000731000000107 | 2577271000000111 | Serum creatinine level |
| 259054018 | 44J3z00 | 1000731000000107 | 2577271000000111 | Serum creatinine NOS |
| 457919018 | 44JC.00 | 1000981000000109 | 2584361000000111 | Corrected plasma creatinine level |
| 457927010 | 44JD.00 | 1000991000000106 | 2571851000000113 | Corrected serum creatinine level |
| 458156012 | 44JF.00 | 1001011000000107 | 2584381000000119 | Plasma creatinine level |

## **Clinical codes used to identify ACR measurements in CPRD Aurum**

| medcodeid | readcode | snomedctconceptid | snomedctdescriptionid | term |
| --- | --- | --- | --- | --- |
| 5289591000006113 |  | 250745003 | 373820014 | Albumin/creatinine ratio measurement |
| 5289601000006117 |  | 250745003 | 373821013 | Albumin/creatinine ratio |
| 5520181000006110 |  | 271075006 | 405655017 | Urine albumin/creatinine ratio |
| 5520171000006112 |  | 271075006 | 405656016 | Urine albumin/creatinine ratio measurement |
| 5520191000006113 |  | 271076007 | 405657013 | Urine microalbumin/creatinine ratio measurement |
| 5520201000006111 |  | 271076007 | 405658015 | Urine microalbumin/creatinine ratio |
| 5520211000006114 |  | 271076007 | 134901000000113 | Urine microalb/creatnine ratio |
| 74961000006113 | 46TC.00 | 1023491000000104 | 2553591000000116 | Urine albumin:creatinine ratio |
| 12681291000006112 |  | 1023491000000104 | 2768311000000117 | Albumin/creatinine ratio in urine |
| 68971000006117 | 46TD.00 | 1027791000000103 | 2602161000000110 | Urine microalbumin/creatinine ratio |
| 476291000006112 | 44J7.00 | 1028271000000109 | 2602141000000114 | Albumin/creatinine ratio |
| 8409231000006115 |  | 1028271000000109 | 2602151000000112 | Albumin / creatinine ratio |
| 1756971000006114 | 46TC.00 | 1756971000006105 | 1756971000006114 | Test request : Urine albumin:creatinine ratio |

## **Clinical codes used to identify blood pressure measurements in CPRD Aurum**

| medcodeid | readcode | snomedctconceptid | snomedctdescriptionid | term | BPtype |
| --- | --- | --- | --- | --- | --- |
| 2869181000006114 |  | 23154005 | 38882014 | High diastolic arterial pressure | Diastolic |
| 2869171000006111 |  | 23154005 | 38883016 | Increased diastolic arterial pressure | Diastolic |
| 2869191000006112 |  | 23154005 | 38886012 | Increased diastolic blood pressure | Diastolic |
| 2869201000006110 |  | 23154005 | 38887015 | Increased diastolic arterial | Diastolic |
| 2869211000006113 |  | 23154005 | 38888013 | High diastolic arterial | Diastolic |
| 3188131000006115 |  | 42689008 | 71227012 | Low diastolic arterial pressure | Diastolic |
| 3188121000006118 |  | 42689008 | 71228019 | Decreased diastolic arterial pressure | Diastolic |
| 3188141000006113 |  | 42689008 | 71231018 | Decreased diastolic blood pressure | Diastolic |
| 3188151000006110 |  | 42689008 | 71232013 | Decreased diastolic arterial | Diastolic |
| 3188161000006112 |  | 42689008 | 71233015 | Low diastolic arterial | Diastolic |
| 3306781000006110 |  | 49844009 | 83026014 | Abnormal diastolic arterial pressure | Diastolic |
| 3306791000006113 |  | 49844009 | 83028010 | Abnormal diastolic blood pressure | Diastolic |
| 3306801000006114 |  | 49844009 | 83029019 | Abnormal diastolic arterial | Diastolic |
| 3372531000006111 |  | 53813002 | 89435017 | Normal diastolic arterial pressure | Diastolic |
| 3372541000006118 |  | 53813002 | 89437013 | Normal diastolic blood pressure | Diastolic |
| 3372551000006116 |  | 53813002 | 89438015 | Normal diastolic arterial | Diastolic |
| 3470001000006117 |  | 59720008 | 99206014 | Sustained diastolic hypertension | Diastolic |
| 3565371000006115 |  | 65518004 | 108859012 | Labile diastolic hypertension | Diastolic |
| 11924381000006114 |  | 163031004 | 254076013 | O/E - Diastolic BP reading | Diastolic |
| 4556621000006110 |  | 163031004 | 2667439010 | On examination - Diastolic blood pressure reading | Diastolic |
| 4556631000006113 |  | 163031004 | 2667440012 | On examination - Diastolic BP reading | Diastolic |
| 4660711000006111 |  | 174255007 | 269956016 | Non-invasive diastolic arterial pressure | Diastolic |
| 4660721000006115 |  | 174255007 | 1221536017 | Non-invasive diastolic blood pressure | Diastolic |
| 5290001000006119 |  | 250769004 | 373855011 | Pulmonary artery diastolic pressure | Diastolic |
| 5290011000006116 |  | 250769004 | 1224438018 | PADP - Pulmonary artery diastolic pressure | Diastolic |
| 5295081000006115 |  | 251073000 | 374208016 | Invasive diastolic arterial pressure | Diastolic |
| 5295091000006117 |  | 251073000 | 1224584013 | Invasive diastolic blood pressure | Diastolic |
| 5527211000006110 |  | 271650006 | 406508013 | Diastolic blood pressure | Diastolic |
| 5527221000006119 |  | 271650006 | 1225212013 | DAP - Diastolic arterial pressure | Diastolic |
| 5527231000006116 |  | 271650006 | 1225213015 | DBP - Diastolic blood pressure | Diastolic |
| 5527241000006114 |  | 271650006 | 2620223019 | Diastolic arterial pressure | Diastolic |
| 6046181000006111 |  | 314451001 | 458814010 | Minimum diastolic blood pressure | Diastolic |
| 458816012 | 246m.00 | 314453003 | 458816012 | Average diastolic blood pressure | Diastolic |
| 6046211000006110 |  | 314454009 | 458817015 | Minimum day interval diastolic blood pressure | Diastolic |
| 6046221000006119 |  | 314455005 | 458818013 | Minimum night interval diastolic blood pressure | Diastolic |
| 6046231000006116 |  | 314456006 | 458819017 | Minimum 24 hour diastolic blood pressure | Diastolic |
| 458823013 | 246a.00 | 314460009 | 458823013 | Average night interval diastolic blood pressure | Diastolic |
| 458824019 | 246X.00 | 314461008 | 458824019 | Average day interval diastolic blood pressure | Diastolic |
| 458825018 | 246V.00 | 314462001 | 458825018 | Average 24 hour diastolic blood pressure | Diastolic |
| 6046321000006114 |  | 314465004 | 458828016 | 24 hour diastolic blood pressure | Diastolic |
| 1780183016 | 246P.00 | 400975005 | 1780183016 | Standing diastolic blood pressure | Diastolic |
| 2159156012 | 246R.00 | 407555005 | 2159156012 | Sitting diastolic blood pressure | Diastolic |
| 2159158013 | 246T.00 | 407557002 | 2159158013 | Lying diastolic blood pressure | Diastolic |
| 2533636015 | 246c.00 | 413605002 | 2533636015 | Average home diastolic blood pressure | Diastolic |
| 7321231000006110 |  | 446226005 | 2883277012 | Diastolic blood pressure on admission | Diastolic |
| 2418921000000115 | 246n000 | 716632005 | 3306936015 | Baseline diastolic blood pressure | Diastolic |
| 125231000006119 |  | 125231000006103 | 125231000006119 | Standing Diastolic Bp | Diastolic |
| 298311000000112 | 246f.00 | 198091000000104 | 298311000000112 | Ambulatory diastolic blood pressure | Diastolic |
| 8057991000006115 |  | 198091000000104 | 1677381000000111 | Ambulatory diastolic BP (blood pressure) | Diastolic |
| 2616401000000117 |  | 1036571000000105 | 2616401000000117 | Non-invasive central diastolic blood pressure | Diastolic |
| 619931000006119 | 246A.00 | 1091811000000102 | 2734671000000117 | Diastolic arterial pressure | Diastolic |
| 1808021000006115 |  | 1808021000006104 | 1808021000006115 | Diastolic blood pressure - left arm | Diastolic |
| 1808051000006112 |  | 1808051000006108 | 1808051000006112 | Diastolic blood pressure - right arm | Diastolic |
| 1928521000006112 |  | 1928521000006108 | 1928521000006112 | Baseline diastolic blood pressure | Diastolic |
| 1993951000006110 |  | 1993951000006106 | 1993951000006110 | Home diastolic blood pressure | Diastolic |
| 2705401000006110 |  | 12929001 | 22163017 | Normal systolic blood pressure | Systolic |
| 2705391000006113 |  | 12929001 | 22164011 | Normal systolic arterial pressure | Systolic |
| 2786491000006114 |  | 18050000 | 30496010 | High systolic arterial pressure | Systolic |
| 2786481000006111 |  | 18050000 | 30497018 | Increased systolic arterial pressure | Systolic |
| 2786501000006118 |  | 18050000 | 30499015 | High systolic arterial pulse pressure | Systolic |
| 2786511000006115 |  | 18050000 | 30500012 | Increased systolic blood pressure | Systolic |
| 2786521000006111 |  | 18050000 | 30501011 | Increased systolic arterial | Systolic |
| 2786531000006114 |  | 18050000 | 30502016 | High systolic arterial | Systolic |
| 2791701000006118 |  | 18352002 | 30984012 | Abnormal systolic blood pressure | Systolic |
| 2791691000006118 |  | 18352002 | 30985013 | Abnormal systolic arterial pressure | Systolic |
| 93494011 | G202.00 | 56218007 | 93494011 | Systolic hypertension | Systolic |
| 114311000006111 | 2469.00 | 72313002 | 120159016 | Systolic arterial pressure | Systolic |
| 3676271000006117 |  | 72313002 | 120163011 | Systolic blood pressure | Systolic |
| 3676261000006112 |  | 72313002 | 3676261000006112 | Systolic arterial pressure | Systolic |
| 3817651000006111 |  | 81010002 | 134379017 | Low systolic arterial pressure | Systolic |
| 3817641000006114 |  | 81010002 | 134380019 | Decreased systolic arterial pressure | Systolic |
| 3817661000006113 |  | 81010002 | 134382010 | Low systolic arterial pulse pressure | Systolic |
| 3817671000006118 |  | 81010002 | 134383017 | Decreased systolic blood pressure | Systolic |
| 3817681000006115 |  | 81010002 | 134384011 | Decreased systolic arterial | Systolic |
| 3817691000006117 |  | 81010002 | 134385012 | Low systolic arterial | Systolic |
| 11903371000006115 |  | 163030003 | 254075012 | O/E - Systolic BP reading | Systolic |
| 4556591000006113 |  | 163030003 | 2667438019 | On examination - Systolic BP reading | Systolic |
| 4556601000006117 |  | 163030003 | 2972649012 | On examination - Systolic blood pressure reading | Systolic |
| 5289991000006115 |  | 250768007 | 373854010 | Pulmonary artery systolic pressure | Systolic |
| 5295021000006119 |  | 251070002 | 374205018 | Non-invasive systolic arterial pressure | Systolic |
| 5295031000006116 |  | 251070002 | 1224579019 | SAP - Systolic arterial pressure | Systolic |
| 5295041000006114 |  | 251070002 | 1224580016 | Non-invasive systolic blood pressure | Systolic |
| 5295051000006111 |  | 251071003 | 374206017 | Invasive systolic arterial pressure | Systolic |
| 5295061000006113 |  | 251071003 | 1224581017 | SAP - Systolic arterial pressure | Systolic |
| 5295071000006118 |  | 251071003 | 1224582012 | Invasive systolic blood pressure | Systolic |
| 5527191000006114 |  | 271649006 | 406507015 | Systolic blood pressure | Systolic |
| 5527201000006112 |  | 271649006 | 1225211018 | SAP - Systolic arterial pressure | Systolic |
| 5590371000006116 |  | 276772001 | 413057017 | Right ventricular systolic pressure | Systolic |
| 5590461000006117 |  | 276780008 | 413065019 | Left ventricular systolic pressure | Systolic |
| 6046051000006119 |  | 314438006 | 458801014 | Minimum systolic blood pressure | Systolic |
| 6046061000006117 |  | 314439003 | 458802019 | Maximum systolic blood pressure | Systolic |
| 458803012 | 246l.00 | 314440001 | 458803012 | Average systolic blood pressure | Systolic |
| 6046081000006110 |  | 314441002 | 458804018 | Minimum day interval systolic blood pressure | Systolic |
| 6046091000006113 |  | 314442009 | 458805017 | Minimum night interval systolic blood pressure | Systolic |
| 6046101000006119 |  | 314443004 | 458806016 | Maximum night interval systolic blood pressure | Systolic |
| 6046111000006116 |  | 314444005 | 458807013 | Maximum day interval systolic blood pressure | Systolic |
| 458808015 | 246b.00 | 314445006 | 458808015 | Average night interval systolic blood pressure | Systolic |
| 458809011 | 246Y.00 | 314446007 | 458809011 | Average day interval systolic blood pressure | Systolic |
| 6046141000006117 |  | 314447003 | 458810018 | Minimum 24 hour systolic blood pressure | Systolic |
| 6046151000006115 |  | 314448008 | 458811019 | Maximum 24 hour systolic blood pressure | Systolic |
| 458812014 | 246W.00 | 314449000 | 458812014 | Average 24 hour systolic blood pressure | Systolic |
| 6046311000006118 |  | 314464000 | 458827014 | 24 hour systolic blood pressure | Systolic |
| 6620591000006114 |  | 399304008 | 1778756019 | Systolic blood pressure on admission | Systolic |
| 1780182014 | 246N.00 | 400974009 | 1780182014 | Standing systolic blood pressure | Systolic |
| 2159155011 | 246Q.00 | 407554009 | 2159155011 | Sitting systolic blood pressure | Systolic |
| 2159157015 | 246S.00 | 407556006 | 2159157015 | Lying systolic blood pressure | Systolic |
| 2533615017 | 246d.00 | 413606001 | 2533615017 | Average home systolic blood pressure | Systolic |
| 7103581000006114 |  | 429457004 | 2695883018 | Systolic essential hypertension | Systolic |
| 2418961000000111 | 246n100 | 716579001 | 3306694016 | Baseline systolic blood pressure | Systolic |
| 125251000006114 |  | 125251000006105 | 125251000006114 | Standing Systolic Bp | Systolic |
| 298281000000114 | 246e.00 | 198081000000101 | 298281000000114 | Ambulatory systolic blood pressure | Systolic |
| 8057971000006116 |  | 198081000000101 | 1677251000000113 | Ambulatory systolic BP (blood pressure) | Systolic |
| 1760931000000113 | 5858300 | 787541000000108 | 1760931000000113 | Highest brachial systolic pressure | Systolic |
| 2122411000000113 | 246j.00 | 814101000000107 | 2122411000000113 | Systolic blood pressure centile | Systolic |
| 2616361000000114 |  | 1036551000000101 | 2616361000000114 | Non-invasive central systolic blood pressure | Systolic |
| 2725431000000119 |  | 1087991000000109 | 2725431000000119 | Level of reduction in systolic blood pressure on standing | Systolic |
| 12618711000006119 |  | 1104341000000101 | 2764451000000116 | Royal College of Physicians NEWS2 (National Early Warning Score 2) - systolic blood pressure score | Systolic |
| 1808011000006111 |  | 1808011000006107 | 1808011000006111 | Systolic blood pressure - left arm | Systolic |
| 1808041000006110 |  | 1808041000006106 | 1808041000006110 | Systolic blood pressure - right arm | Systolic |
| 1928511000006116 |  | 1928511000006100 | 1928511000006116 | Baseline systolic blood pressure | Systolic |
| 1994021000006115 |  | 1994021000006104 | 1994021000006115 | Home systolic blood pressure | Systolic |
| 64172013 | G20..11 | 24184005 | 196353013 | Elevated blood pressure | Unspecified |
| 2885771000006113 |  | 24184005 | 196354019 | Finding of increased blood pressure | Unspecified |
| 317910016 | R1y2.00 | 24184005 | 1784950015 | Raised blood pressure | Unspecified |
| 2885791000006114 |  | 24184005 | 2920698012 | Blood pressure elevation | Unspecified |
| 523801000006119 | G2...11 | 38341003 | 64168014 | BP - hypertensive disease | Unspecified |
| 3117411000006118 |  | 38341003 | 64172013 | High blood pressure | Unspecified |
| 3117451000006117 |  | 38341003 | 490277011 | BP - High blood pressure | Unspecified |
| 3117471000006110 |  | 38341003 | 490280012 | HBP - High blood pressure | Unspecified |
| 216600016 | 662V.00 | 135840009 | 216600016 | Blood pressure monitoring | Unspecified |
| 254091010 | 246Z.00 | 163020007 | 254063019 | O/E-blood pressure reading NOS | Unspecified |
| 254063019 | 246..00 | 163020007 | 254063019 | O/E - blood pressure reading | Unspecified |
| 254064013 | 246..12 | 163020007 | 254064013 | O/E - blood pressure | Unspecified |
| 254065014 | 246..11 | 163020007 | 254065014 | O/E - BP reading | Unspecified |
| 4556301000006111 |  | 163020007 | 2667419011 | On examination - blood pressure reading | Unspecified |
| 254067018 | 2461.00 | 163022004 | 254067018 | O/E - BP reading very low | Unspecified |
| 4556351000006110 |  | 163022004 | 2667422013 | On examination - blood pressure reading very low | Unspecified |
| 4556361000006112 |  | 163022004 | 2667423015 | On examination - BP reading very low | Unspecified |
| 254068011 | 2462.00 | 163023009 | 254068011 | O/E - BP reading low | Unspecified |
| 4556381000006119 |  | 163023009 | 2667424014 | On examination - blood pressure reading low | Unspecified |
| 4556391000006116 |  | 163023009 | 2667425010 | On examination - BP reading low | Unspecified |
| 254069015 | 2463.00 | 163024003 | 254069015 | O/E - BP borderline low | Unspecified |
| 4556411000006116 |  | 163024003 | 2667426011 | On examination - blood pressure borderline low | Unspecified |
| 4556421000006112 |  | 163024003 | 2667427019 | On examination - BP borderline low | Unspecified |
| 254070019 | 2464.00 | 163025002 | 254070019 | O/E - BP reading normal | Unspecified |
| 4556441000006117 |  | 163025002 | 2667428012 | On examination - blood pressure reading normal | Unspecified |
| 4556451000006115 |  | 163025002 | 2667429016 | On examination - BP reading normal | Unspecified |
| 254071015 | 2465.00 | 163026001 | 254071015 | O/E - BP borderline raised | Unspecified |
| 4556471000006113 |  | 163026001 | 2667430014 | On examination - blood pressure borderline raised | Unspecified |
| 4556481000006111 |  | 163026001 | 2667431013 | On examination - BP borderline raised | Unspecified |
| 254072010 | 2466.00 | 163027005 | 254072010 | O/E - BP reading raised | Unspecified |
| 4556501000006118 |  | 163027005 | 2667432018 | On examination - blood pressure reading raised | Unspecified |
| 4556511000006115 |  | 163027005 | 2667433011 | On examination - BP reading raised | Unspecified |
| 254073017 | 2467.00 | 163028000 | 254073017 | O/E - BP reading very high | Unspecified |
| 4556531000006114 |  | 163028000 | 2667434017 | On examination - blood pressure reading very high | Unspecified |
| 4556541000006116 |  | 163028000 | 2667435016 | On examination - BP reading very high | Unspecified |
| 254074011 | 2468.00 | 163029008 | 254074011 | O/E - BP reading:postural drop | Unspecified |
| 4556561000006117 |  | 163029008 | 2667436015 | On examination - blood pressure reading: postural drop | Unspecified |
| 4556571000006112 |  | 163029008 | 2667437012 | On examination - BP reading:postural drop | Unspecified |
| 254077016 | 246B.00 | 163032006 | 254077016 | O/E - BP stable | Unspecified |
| 4556651000006118 |  | 163032006 | 2667441011 | On examination - blood pressure stable | Unspecified |
| 4556661000006116 |  | 163032006 | 2667626019 | On examination - BP stable | Unspecified |
| 4556671000006111 |  | 163033001 | 254078014 | Lying blood pressure | Unspecified |
| 254079018 | 246C.00 | 163033001 | 254079018 | Lying blood pressure reading | Unspecified |
| 254080015 | 246D.00 | 163034007 | 254080015 | Standing blood pressure reading | Unspecified |
| 4556691000006112 |  | 163034007 | 254081016 | Standing blood pressure | Unspecified |
| 4556711000006110 |  | 163035008 | 254082011 | Sitting blood pressure | Unspecified |
| 254083018 | 246E.00 | 163035008 | 254083018 | Sitting blood pressure reading | Unspecified |
| 254084012 | 246F.00 | 163036009 | 254084012 | O/E - blood pressure decreased | Unspecified |
| 4556741000006114 |  | 163036009 | 2667627011 | On examination - blood pressure decreased | Unspecified |
| 254085013 | 246G.00 | 163037000 | 254085013 | O/E - BP labile | Unspecified |
| 4556761000006113 |  | 163037000 | 2667628018 | On examination - blood pressure labile | Unspecified |
| 4556771000006118 |  | 163037000 | 2667629014 | On examination - BP labile | Unspecified |
| 256343017 | 315B.00 | 164783007 | 256343017 | Ambulatory blood pressure recording | Unspecified |
| 264466017 | 6623.00 | 170573004 | 264466017 | Pre-treatment BP reading | Unspecified |
| 4634651000006115 |  | 170573004 | 2972104013 | Pre-treatment blood pressure reading | Unspecified |
| 264475015 | 662B.00 | 170581003 | 264475015 | O/E - initial high BP | Unspecified |
| 4634761000006110 |  | 170581003 | 2667129010 | On examination - initial high blood pressure | Unspecified |
| 4634771000006115 |  | 170581003 | 2667130017 | On examination - initial high BP | Unspecified |
| 264476019 | 662C.00 | 170582005 | 264476019 | O/E - check high BP | Unspecified |
| 4634791000006119 |  | 170582005 | 2667131018 | On examination - check high blood pressure | Unspecified |
| 4634801000006118 |  | 170582005 | 2667132013 | On examination - check high BP | Unspecified |
| 264498011 | 662L.00 | 170599006 | 264498011 | 24 hr blood pressure monitoring | Unspecified |
| 12758121000006114 |  | 170599006 | 1859511000000113 | 24 hour blood pressure monitoring | Unspecified |
| 317911017 | R1y3.00 | 271870002 | 406863012 | Low blood pressure reading | Unspecified |
| 317912012 | R1y4.00 | 271871003 | 406864018 | BP reading labile | Unspecified |
| 5530451000006114 |  | 271871003 | 2972308015 | Blood pressure reading labile | Unspecified |
| 5566331000006119 |  | 274785000 | 410648013 | Examination of blood pressure | Unspecified |
| 461166013 | ZV70B00 | 274785000 | 410648013 | [V]Examination of blood pressure | Unspecified |
| 459379010 | 662Q.00 | 314956000 | 459379010 | Borderline blood pressure | Unspecified |
| 2474699019 | 662j.00 | 413153004 | 2474699019 | Blood pressure recorded by patient at home | Unspecified |
| 622341000000118 | 246g.00 | 335661000000109 | 622341000000118 | Self measured blood pressure reading | Unspecified |
| 8107481000006110 |  | 335661000000109 | 1692821000000111 | Self measured BP (blood pressure) reading | Unspecified |
| 895081000006112 | R1y3.99 | 501631000000108 | 895081000006112 | Nonspecific low BP reading [D] | Unspecified |
| 12717981000006117 |  | 501631000000108 | 1096241000000113 | [D]Low blood pressure reading | Unspecified |

## **Clinical codes used to identify hypertension in CPRD Aurum**

| medcodeid | readcode | snomedctconceptid | snomedctdescriptionid | term |
| --- | --- | --- | --- | --- |
| 1409014 | G221.00 | 193003 | 1409014 | Benign hypertensive renal disease |
| 1529013 | F404200 | 264008 | 1529013 | Blind hypertensive eye |
| 3135013 | G201.00 | 1201005 | 3135013 | Benign essential hypertension |
| 12496011 | F421300 | 6962006 | 12496011 | Hypertensive retinopathy |
| 2681651000006116 |  | 11399002 | 19722017 | Pulmonary hypertensive arterial disease |
| 2681661000006119 |  | 11399002 | 19723010 | Hypertensive pulmonary arterial disease |
| 43850011 | G410.00 | 11399002 | 2968228010 | Pulmonary arterial hypertension |
| 64172013 | G20..11 | 24184005 | 196353013 | Elevated blood pressure |
| 158241000006117 | G24z.00 | 31992008 | 53452019 | Secondary hypertension NOS |
| 300871017 | Gyu2100 | 31992008 | 53452019 | [X]Hypertension secondary to other renal disorders |
| 299682016 | G24zz00 | 31992008 | 53452019 | Secondary hypertension NOS |
| 53452019 | G24..00 | 31992008 | 53452019 | Secondary hypertension |
| 300870016 | Gyu2000 | 31992008 | 53452019 | [X]Other secondary hypertension |
| 57989014 | J623.00 | 34742003 | 57989014 | Portal hypertension |
| 3059491000006115 |  | 34742003 | 1228208015 | PHT - Portal hypertension |
| 60444016 | G211.00 | 36221001 | 60444016 | Benign hypertensive heart disease |
| 299654011 | G211z00 | 36221001 | 60444016 | Benign hypertensive heart disease NOS |
| 728681000006116 | G210000 | 36315003 | 60617018 | Malignant hypertensive heart disease without congestive heart failure |
| 523801000006119 | G2...11 | 38341003 | 64168014 | BP - hypertensive disease |
| 64168014 | G2...00 | 38341003 | 64168014 | Hypertensive disease |
| 3117411000006118 |  | 38341003 | 64172013 | High blood pressure |
| 3117421000006114 |  | 38341003 | 64173015 | Hypertensive vascular disease |
| 3117431000006112 |  | 38341003 | 64174014 | Hypertensive vascular degeneration |
| 790121000006116 | G20z.11 | 38341003 | 64176011 | Hypertension |
| 3117451000006117 |  | 38341003 | 490277011 | BP - High blood pressure |
| 3117461000006115 |  | 38341003 | 490278018 | Systemic arterial hypertension |
| 3117471000006110 |  | 38341003 | 490280012 | HBP - High blood pressure |
| 3117481000006113 |  | 38341003 | 490281011 | HT - Hypertension |
| 3117491000006111 |  | 38341003 | 490282016 | High blood pressure disorder |
| 3117501000006115 |  | 38341003 | 490283014 | BP+ - Hypertension |
| 300869017 | Gyu2.00 | 38341003 | 1215744012 | [X]Hypertensive diseases |
| 299686018 | G2y..00 | 38341003 | 1215744012 | Other specified hypertensive disease |
| 299687010 | G2z..00 | 38341003 | 1215744012 | Hypertensive disorder |
| 3117511000006117 |  | 38341003 | 2164904016 | HTN - Hypertension |
| 3117521000006113 |  | 38341003 | 2671386015 | Hypertensive disorder, systemic arterial |
| 47076011 | G22z.11 | 38481006 | 64282015 | Renal hypertension |
| 64282015 | G22..00 | 38481006 | 64282015 | Hypertensive renal disease |
| 395753015 | G22z.00 | 38481006 | 64282015 | Hypertensive renal disease NOS |
| 54939014 | G22..11 | 38481006 | 64282015 | Nephrosclerosis |
| 3119661000006114 |  | 38481006 | 64285018 | Hypertensive nephropathy |
| 11777411000006113 |  | 38481006 | 3512257015 | Renal disease due to hypertension |
| 80224019 | G203.00 | 48146000 | 80224019 | Diastolic hypertension |
| 3296351000006114 |  | 49220004 | 81984019 | Hypertensive renal failure |
| 299665018 | G222.00 | 49220004 | 81984019 | Hypertensive renal disease with renal failure |
| 84112010 | G672.11 | 50490005 | 84111015 | Hypertensive crisis |
| 84111015 | G672.00 | 50490005 | 84111015 | Hypertensive encephalopathy |
| 90135019 | G210.00 | 54225002 | 90135019 | Malignant hypertensive heart disease |
| 299650019 | G210z00 | 54225002 | 90135019 | Malignant hypertensive heart disease NOS |
| 93494011 | G202.00 | 56218007 | 93494011 | Systolic hypertension |
| 99042012 | G20..00 | 59621000 | 99042012 | Essential hypertension |
| 395751018 | G20z.00 | 59621000 | 99042012 | Essential hypertension NOS |
| 3468501000006117 |  | 59621000 | 99046010 | Systemic primary arterial hypertension |
| 99047018 | G20..12 | 59621000 | 99047018 | Primary hypertension |
| 107545013 | G21..00 | 64715009 | 107545013 | Hypertensive heart disease |
| 741681000006111 | G21z100 | 64715009 | 107545013 | Hypertensive heart disease NOS with CCF |
| 741691000006114 | G21z000 | 64715009 | 107545013 | Hypertensive heart disease NOS without CCF |
| 299655012 | G21zz00 | 64715009 | 107545013 | Hypertensive heart disease NOS |
| 741661000006118 | G21z.00 | 64715009 | 107545013 | Hypertensive heart disease NOS |
| 3552501000006119 |  | 64715009 | 107547017 | Hypertensive cardiopathy |
| 3552511000006116 |  | 64715009 | 107548010 | Hypertensive cardiomegaly |
| 3552521000006112 |  | 64715009 | 107549019 | Hypertensive cardiovascular disease |
| 3552531000006110 |  | 64715009 | 499930018 | HHD - Hypertensive heart disease |
| 108730018 | G220.00 | 65443008 | 108730018 | Malignant hypertensive renal disease |
| 109700019 | G231.00 | 66052004 | 109700019 | Benign hypertensive heart AND renal disease |
| 110659019 | G230.00 | 66610008 | 110659019 | Malignant hypertensive heart AND renal disease |
| 3642801000006112 |  | 70272006 | 116721012 | Malignant hypertension |
| 3654631000006113 |  | 70995007 | 117919017 | Pulmonary hypertension |
| 3654641000006115 |  | 70995007 | 1233340012 | PHT - Pulmonary hypertension |
| 3693711000006119 |  | 73410007 | 121910014 | Benign secondary renovascular hypertension |
| 2478822013 | G241000 | 73410007 | 2478822013 | Secondary benign renovascular hypertension |
| 3768511000006114 |  | 77970009 | 129404019 | Benign hypertensive heart disease without congestive heart failure |
| 504911000006115 | G211000 | 77970009 | 129404019 | Benign hypertensive heart disease without CCF |
| 131046010 | G200.00 | 78975002 | 131046010 | Malignant essential hypertension |
| 3784371000006115 |  | 78975002 | 503982017 | Accelerated essential hypertension |
| 884121000006111 | G200.99 | 78975002 | 884121000006111 | Malignant hypertension |
| 3852501000006115 |  | 83105008 | 137848017 | Malignant hypertensive heart disease with congestive heart failure |
| 728671000006119 | G210100 | 83105008 | 1236017010 | Malignant hypertensive heart disease with congestive cardiac failure |
| 19451000006111 | L122000 | 86041002 | 142647015 | Other pre-existing hypertension in preg/childb/puerp unspec |
| 305764016 | L122.00 | 86041002 | 142647015 | Other pre-existing hypertension in preg/childbirth/puerp |
| 19421000006119 | L122300 | 86041002 | 142647015 | Other pre-exist hypertension in preg/childb/puerp-not deliv |
| 19431000006116 | L122100 | 86041002 | 142647015 | Other pre-existing hypertension in preg/childb/puerp - deliv |
| 19411000006110 | L122400 | 86041002 | 142647015 | Other pre-exist hypertension in preg/childb/puerp + p/n comp |
| 3898601000006112 |  | 86041002 | 142647015 | Pre-existing hypertension in obstetric context |
| 39871000006113 | L122200 | 86041002 | 142647015 | Oth pre-exist hypert in preg/childb/puerp -del with p/n comp |
| 19441000006114 | L122z00 | 86041002 | 142647015 | Other pre-existing hypertension in preg/childb/puerp NOS |
| 3898611000006110 |  | 86041002 | 506901015 | Pre-existing hypertension complicating pregnancy |
| 143003017 | G23..00 | 86234004 | 143003017 | Hypertensive heart AND renal disease |
| 299675015 | G23z.00 | 86234004 | 143003017 | Hypertensive heart and renal disease NOS |
| 3902031000006117 |  | 86234004 | 143006013 | Cardiorenal disease |
| 884131000006114 | G23..99 | 86234004 | 884131000006114 | Hypertensive renal + heart dis |
| 146259010 | G41y000 | 88223008 | 146259010 | Secondary pulmonary hypertension |
| 299677011 | G240z00 | 89242004 | 147988014 | Secondary malignant hypertension NOS |
| 151161000006115 | G240.00 | 89242004 | 147988014 | Malignant secondary hypertension |
| 3950631000006119 |  | 89242004 | 508416019 | Accelerated secondary hypertension |
| 422681000006111 | SyuFT00 | 111769007 | 178964016 | Poisoning by antihypertensive agent |
| 4199551000006110 |  | 111769007 | 1219690017 | Antihypertensive poisoning |
| 4199561000006112 |  | 111769007 | 3296950011 | Poisoning caused by antihypertensive agent |
| 299683014 | G24z000 | 123799005 | 192288012 | Renovascular hypertension |
| 4356081000006115 |  | 123799005 | 1220673019 | Hypertension due to renovascular disease |
| 251674014 | 14A2.00 | 161501007 | 251674014 | H/O: hypertension |
| 4540441000006117 |  | 161501007 | 2987063016 | History of hypertension |
| 262960017 | 6146200 | 169465000 | 262960017 | Hypertension induced by oral contraceptive pill |
| 4623221000006114 |  | 169465000 | 3290297011 | Hypertension caused by oral contraceptive pill |
| 264471012 | 6627.00 | 170577003 | 264471012 | Good hypertension control |
| 264472017 | 6628.00 | 170578008 | 264472017 | Poor hypertension control |
| 12488211000006112 |  | 170586008 | 264485019 | Hypertension treatm. started |
| 264485019 | 662F.00 | 170586008 | 2694699015 | Treatment for hypertension started |
| 4634851000006119 |  | 170586008 | 4634851000006119 | Treatment for hypertension started |
| 12487991000006113 |  | 170587004 | 264486018 | Hypertensive treatm.changed |
| 264486018 | 662G.00 | 170587004 | 2871342011 | Hypertensive treatment changed |
| 4634871000006112 |  | 170587004 | 4634871000006112 | Hypertensive treatment changed |
| 504901000006118 | G211100 | 194767001 | 299653017 | Benign hypertensive heart disease with congestive cardiac failure |
| 741701000006114 | G232.00 | 194779001 | 299672017 | Hypertensive heart and renal disease with (congestive) heart failure |
| 299673010 | G233.00 | 194780003 | 299673010 | Hypertensive heart and renal disease with renal failure |
| 789941000006117 | G234.00 | 194781004 | 299674016 | Hypertensive heart and renal disease with both (congestive) heart failure and renal failure |
| 299676019 | G240000 | 194783001 | 299676019 | Secondary malignant renovascular hypertension |
| 4775791000006113 |  | 194783001 | 2470030014 | Malignant secondary renovascular hypertension |
| 299678018 | G241.00 | 194785008 | 299678018 | Secondary benign hypertension |
| 299680012 | G241z00 | 194785008 | 2470031013 | Benign secondary hypertension |
| 299681011 | G244.00 | 194788005 | 299681011 | Hypertension secondary to endocrine disorders |
| 4775831000006118 |  | 194788005 | 2532161014 | Hypertension secondary to endocrine disorder |
| 299684015 | G24z100 | 194791005 | 299684015 | Hypertension secondary to drug |
| 4775861000006110 |  | 194791005 | 3290640017 | Hypertension caused by drug |
| 887811000006117 | L120.99 | 198942000 | 887811000006117 | Preg.+ pre-existing hypertensn |
| 305818015 | L127.00 | 198997005 | 305818015 | Pre-eclampsia or eclampsia with pre-existing hypertension |
| 212961000006118 | L127z00 | 198997005 | 305818015 | Pre-eclampsia or eclampsia + pre-existing hypertension NOS |
| 212991000006114 | L127100 | 198999008 | 305820017 | Pre-eclampsia or eclampsia with pre-existing hypertension - delivered |
| 212981000006111 | L127200 | 199000005 | 305821018 | Pre-eclampsia or eclampsia with pre-existing hypertension - delivered with postnatal complication |
| 213001000006110 | L127300 | 199002002 | 305823015 | Pre-eclampsia or eclampsia with pre-existing hypertension - not delivered |
| 212971000006113 | L127400 | 199003007 | 305824014 | Pre-eclampsia or eclampsia with pre-existing hypertension with postnatal complication |
| 213111000006112 | L128.00 | 199005000 | 305826011 | Pre-existing hypertension complicating pregnancy, childbirth and puerperium |
| 213091000006115 | L128000 | 199006004 | 305827019 | Pre-existing hypertensive heart disease complicating pregnancy, childbirth and the puerperium |
| 213101000006114 | L128100 | 199007008 | 305828012 | Pre-existing hypertensive heart and renal disease complicating pregnancy, childbirth and the puerperium |
| 213081000006118 | L128200 | 199008003 | 305829016 | Pre-existing secondary hypertension complicating pregnancy, childbirth and puerperium |
| 350517010 | G41y100 | 233947005 | 350517010 | Thromboembolic pulmonary hypertension |
| 5057801000006113 |  | 233947005 | 2968345015 | Chronic thromboembolic pulmonary hypertension |
| 411508017 | G21z011 | 275516004 | 411508017 | Cardiomegaly - hypertensive |
| 443764015 | 662O.00 | 302192008 | 443764015 | On treatment for hypertension |
| 451424017 | 8B26.00 | 308116003 | 451424017 | Antihypertensive therapy |
| 1846941000006119 | G25..00 | 843821000000102 | 2189411000000111 | Stage 1 hypertension (NICE - National Institute for Health and Clinical Excellence 2011) |
| 2193031000000112 | G25..11 | 843821000000102 | 2193031000000112 | Stage 1 hypertension |
| 1846961000006115 | G26..00 | 843841000000109 | 2189451000000110 | Severe hypertension (NICE - National Institute for Health and Clinical Excellence 2011) |
| 2193021000000110 | G26..11 | 843841000000109 | 2193021000000110 | Severe hypertension |
| 2193971000000110 | G27..00 | 845891000000103 | 2193971000000110 | Hypertension resistant to drug therapy |
| 8286321000006117 |  | 845891000000103 | 2194001000000116 | Resistant hypertension |
| 1846991000006111 | G28..00 | 846371000000103 | 2194941000000119 | Stage 2 hypertension (NICE - National Institute for Health and Clinical Excellence 2011) |
| 8286581000006114 |  | 846371000000103 | 2211211000000110 | Stage 2 hypertension |
| 2297871000000111 | 7Q01400 | 892401000000105 | 2297871000000111 | Pulmonary arterial hypertension drugs Band 1 |
| 2297911000000113 | 7Q01500 | 892421000000101 | 2297911000000113 | Pulmonary arterial hypertension drugs Band 2 |
| 2297951000000112 | 7Q01600 | 892441000000108 | 2297951000000112 | Pulmonary arterial hypertension drugs Band 3 |
| 2297991000000116 | 7Q01700 | 892461000000109 | 2297991000000116 | Pulmonary arterial hypertension drugs Band 4 |
| 905451000006118 |  | 905451000006102 | 905451000006118 | [RFC] Pulmonary embolism/pulmonary hypertension |
| 1908711000006115 | G250.00 | 908631000000108 | 2335761000000116 | Stage 1 hypertension (NICE 2011) without evidence of end organ damage |
| 1908721000006111 | G251.00 | 908651000000101 | 2335801000000114 | Stage 1 hypertension (NICE 2011) with evidence of end organ damage |
| 909441000006118 |  | 909441000006102 | 909441000006118 | [RFC] Hypertension |
| 1806071000006118 |  | 1806071000006102 | 1806071000006118 | Stage 1 hypertension |
| 1806081000006115 |  | 1806081000006104 | 1806081000006115 | Stage 2 hypertension |
| 1806141000006113 |  | 1806141000006109 | 1806141000006113 | Severe hypertension |
| 1823901000006112 |  | 1823901000006108 | 1823901000006112 | Hypertension confirmed |

## **ICD10 codes used to identify hypertension in HES Admitted Patient Care data**

| icd | description |
| --- | --- |
| I10 | Essential (primary) hypertension |
| I11.0 | Hypertensive heart disease with heart failure |
| I11.9 | Hypertensive heart disease without heart failure |
| I12.0 | Hypertensive chronic kidney disease with stage 5 chronic kidney disease or end stage renal disease |
| I12.9 | Hypertensive chronic kidney disease with stage 1 through stage 4 chronic kidney disease, or unspecified chronic kidney disease |
| I13.0 | Hypertensive heart and chronic kidney disease with heart failure and stage 1 through stage 4 chronic kidney disease, or unspecified chronic kidney disease |
| I13.1 | Hypertensive heart and renal disease with renal failure |
| I13.10 | Hypertensive heart and chronic kidney disease without heart failure, with stage 1 through stage 4 chronic kidney disease, or unspecified chronic kidney disease |
| I13.11 | Hypertensive heart and chronic kidney disease without heart failure, with stage 5 chronic kidney disease, or end stage renal disease |
| I13.2 | Hypertensive heart and chronic kidney disease with heart failure and with stage 5 chronic kidney disease, or end stage renal disease |
| I13.9 | Hypertensive heart and renal disease, unspecified |
| I15 | Secondary hypertension |
| I15.0 | Renovascular hypertension |
| I15.1 | Hypertension secondary to other renal disorders |
| I15.2 | Hypertension secondary to endocrine disorders |
| I15.8 | Other secondary hypertension |
| I15.9 | Secondary hypertension, unspecified |
| I67.4 | Hypertensive encephalopathy |

## **Clinical codes used to identify CKD in CPRD Aurum**

| medcodeid | readcode | snomedctconceptid | snomedctdescriptionid | term | CKDstage |
| --- | --- | --- | --- | --- | --- |
| 2767383018 | K051.00 | 431855005 | 2767383018 | Chronic kidney disease stage 1 | 1 |
| 304031000000117 | 1Z10.00 | 431855005 | 2767383018 | Chronic kidney disease stage 1 | 1 |
| 7147741000006114 |  | 431855005 | 2871055019 | CKD stage 1 | 1 |
| 7966791000006111 |  | 751000119104 | 3013056018 | Chronic kidney disease stage 1 due to type 2 diabetes mellitus | 1 |
| 7966801000006112 |  | 751000119104 | 3013102011 | Diabetic stage 1 chronic renal impairment due to type 2 diabetes mellitus | 1 |
| 7966811000006110 |  | 751000119104 | 3035118012 | Chronic kidney disease stage 1 due to type II diabetes mellitus | 1 |
| 8027391000006115 |  | 90721000119101 | 3010567012 | Chronic kidney disease stage 1 due to type 1 diabetes mellitus | 1 |
| 8027401000006118 |  | 90721000119101 | 3035122019 | Chronic kidney disease stage 1 due to type I diabetes mellitus | 1 |
| 8030451000006117 |  | 96751000119106 | 2968355016 | Hypertensive heart AND chronic kidney disease stage 1 | 1 |
| 8036941000006111 |  | 117681000119102 | 2968242019 | Chronic kidney disease stage 1 due to hypertension | 1 |
| 8087051000006119 |  | 284971000119100 | 3040949015 | Chronic kidney disease stage 1 due to benign hypertension | 1 |
| 8087661000006111 |  | 285851000119102 | 2922173018 | Malignant hypertensive chronic kidney disease stage 1 | 1 |
| 595541000000119 | 1Z17.00 | 324121000000109 | 595541000000119 | Chronic kidney disease stage 1 with proteinuria | 1 |
| 618391000000118 | 1Z17.11 | 324121000000109 | 618391000000118 | CKD stage 1 with proteinuria | 1 |
| 8104101000006118 |  | 324121000000109 | 1677051000000115 | CKD (chronic kidney disease stage) 1 with proteinuria | 1 |
| 595601000000118 | 1Z18.00 | 324151000000104 | 595601000000118 | Chronic kidney disease stage 1 without proteinuria | 1 |
| 618401000000115 | 1Z18.11 | 324151000000104 | 618401000000115 | CKD stage 1 without proteinuria | 1 |
| 8104131000006114 |  | 324151000000104 | 1677041000000118 | CKD (chronic kidney disease) stage 1 without proteinuria | 1 |
| 1940561000006118 | 1Z1M.00 | 949401000000103 | 2426331000000114 | CKD G1A1 - chronic kidney disease with glomerular filtration rate category G1 and albuminuria category A1 | 1 |
| 8345541000006113 |  | 949401000000103 | 2426361000000116 | Chronic kidney disease with glomerular filtration rate category G1 and albuminuria category A1 | 1 |
| 1940571000006113 | 1Z1N.00 | 949421000000107 | 2426381000000113 | CKD G1A2 - chronic kidney disease with glomerular filtration rate category G1 and albuminuria category A2 | 1 |
| 8345571000006117 |  | 949421000000107 | 2426411000000110 | Chronic kidney disease with glomerular filtration rate category G1 and albuminuria category A2 | 1 |
| 1940581000006111 | 1Z1P.00 | 949481000000108 | 2426511000000114 | CKD G1A3 - chronic kidney disease with glomerular filtration rate category G1 and albuminuria category A3 | 1 |
| 8345611000006110 |  | 949481000000108 | 2426541000000110 | Chronic kidney disease with glomerular filtration rate category G1 and albuminuria category A3 | 1 |
| 994401000006118 |  | 994401000006102 | 994401000006118 | Chronic kidney disease stage 1 | 1 |
| 304051000000112 | 1Z11.00 | 431856006 | 2767384012 | Chronic kidney disease stage 2 | 2 |
| 2767384012 | K052.00 | 431856006 | 2767384012 | Chronic kidney disease stage 2 | 2 |
| 7147761000006113 |  | 431856006 | 2870828017 | CKD stage 2 | 2 |
| 7966761000006115 |  | 741000119101 | 3013063018 | Chronic kidney disease stage 2 due to type 2 diabetes mellitus | 2 |
| 7966771000006110 |  | 741000119101 | 3013120019 | Diabetic stage 2 chronic renal impairment due to type 2 diabetes mellitus | 2 |
| 7966781000006113 |  | 741000119101 | 3035128015 | Chronic kidney disease stage 2 due to type II diabetes mellitus | 2 |
| 8027411000006115 |  | 90731000119103 | 3010552013 | Chronic kidney disease stage 2 due to type 1 diabetes mellitus | 2 |
| 8027421000006111 |  | 90731000119103 | 3035107012 | Chronic kidney disease stage 2 due to type I diabetes mellitus | 2 |
| 8030441000006119 |  | 96741000119109 | 2922230015 | Hypertensive heart AND chronic kidney disease stage 2 | 2 |
| 8040691000006110 |  | 129181000119109 | 2968276011 | Chronic kidney disease stage 2 due to hypertension | 2 |
| 8044081000006112 |  | 140131000119102 | 3013117010 | Hypertension in chronic kidney disease stage 2 due to type 2 diabetes mellitus | 2 |
| 8044091000006110 |  | 140131000119102 | 3035152010 | Hypertension in chronic kidney disease stage 2 due to type II diabetes mellitus | 2 |
| 8087061000006117 |  | 284981000119102 | 3040859015 | Chronic kidney disease stage 2 due to benign hypertension | 2 |
| 8087681000006118 |  | 285861000119100 | 2922194015 | Malignant hypertensive chronic kidney disease stage 2 | 2 |
| 595661000000119 | 1Z19.00 | 324181000000105 | 595661000000119 | Chronic kidney disease stage 2 with proteinuria | 2 |
| 618411000000118 | 1Z19.11 | 324181000000105 | 618411000000118 | CKD stage 2 with proteinuria | 2 |
| 8104161000006117 |  | 324181000000105 | 1677031000000110 | CKD (chronic kidney disease) stage 2 with proteinuria | 2 |
| 595721000000116 | 1Z1A.00 | 324211000000106 | 595721000000116 | Chronic kidney disease stage 2 without proteinuria | 2 |
| 618421000000112 | 1Z1A.11 | 324211000000106 | 618421000000112 | CKD stage 2 without proteinuria | 2 |
| 8104191000006113 |  | 324211000000106 | 1676881000000118 | CKD (chronic kidney disease) stage 2 without proteinuria | 2 |
| 1940591000006114 | 1Z1Q.00 | 949521000000108 | 2426601000000111 | CKD G2A1 - chronic kidney disease with glomerular filtration rate category G2 and albuminuria category A1 | 2 |
| 8345651000006111 |  | 949521000000108 | 2426671000000115 | Chronic kidney disease with glomerular filtration rate category G2 and albuminuria category A1 | 2 |
| 1940601000006118 | 1Z1R.00 | 949561000000100 | 2426691000000116 | CKD G2A2 - chronic kidney disease with glomerular filtration rate category G2 and albuminuria category A2 | 2 |
| 8345681000006115 |  | 949561000000100 | 2426801000000110 | Chronic kidney disease with glomerular filtration rate category G2 and albuminuria category A2 | 2 |
| 1940611000006115 | 1Z1S.00 | 949621000000109 | 2426821000000118 | CKD G2A3 - chronic kidney disease with glomerular filtration rate category G2 and albuminuria category A3 | 2 |
| 8345731000006113 |  | 949621000000109 | 2426851000000111 | Chronic kidney disease with glomerular filtration rate category G2 and albuminuria category A3 | 2 |
| 994411000006115 |  | 994411000006104 | 994411000006115 | Chronic kidney disease stage 2 | 2 |
| 304071000000115 | 1Z12.00 | 433144002 | 2773184015 | Chronic kidney disease stage 3 | 3 |
| 2773184015 | K053.00 | 433144002 | 2773184015 | Chronic kidney disease stage 3 | 3 |
| 7174791000006111 |  | 433144002 | 2873059015 | CKD stage 3 | 3 |
| 557811000000119 | 1Z15.00 | 700378005 | 2990386017 | Chronic kidney disease stage 3A | 3 |
| 557831000000110 | 1Z16.00 | 700379002 | 2990361014 | Chronic kidney disease stage 3B | 3 |
| 7966701000006116 |  | 731000119105 | 3013036019 | Diabetic stage 3 chronic renal impairment due to type 2 diabetes mellitus | 3 |
| 7966691000006116 |  | 731000119105 | 3013103018 | Chronic kidney disease stage 3 due to type 2 diabetes mellitus | 3 |
| 7966711000006118 |  | 731000119105 | 3035131019 | Chronic kidney disease stage 3 due to type II diabetes mellitus | 3 |
| 8027451000006119 |  | 90741000119107 | 3013167015 | Chronic kidney disease stage 3 due to type 1 diabetes mellitus | 3 |
| 8027461000006117 |  | 90741000119107 | 3035149019 | Chronic kidney disease stage 3 due to type I diabetes mellitus | 3 |
| 8030431000006112 |  | 96731000119100 | 2922231016 | Hypertensive heart AND chronic kidney disease stage 3 | 3 |
| 8040681000006112 |  | 129171000119106 | 2968262010 | Chronic kidney disease stage 3 due to hypertension | 3 |
| 8044061000006119 |  | 140121000119100 | 3013055019 | Hypertension in chronic kidney disease stage 3 due to type 2 diabetes mellitus | 3 |
| 8044071000006114 |  | 140121000119100 | 3035146014 | Hypertension in chronic kidney disease stage 3 due to type II diabetes mellitus | 3 |
| 8087071000006112 |  | 284991000119104 | 3040933017 | Chronic kidney disease stage 3 due to benign hypertension | 3 |
| 8087691000006115 |  | 285871000119106 | 2922170015 | Malignant hypertensive chronic kidney disease stage 3 | 3 |
| 595811000000117 | 1Z1B.00 | 324251000000105 | 595811000000117 | Chronic kidney disease stage 3 with proteinuria | 3 |
| 618431000000114 | 1Z1B.11 | 324251000000105 | 618431000000114 | CKD stage 3 with proteinuria | 3 |
| 8104231000006115 |  | 324251000000105 | 1676871000000115 | CKD (chronic kidney disease) stage 3 with proteinuria | 3 |
| 595871000000110 | 1Z1C.00 | 324281000000104 | 595871000000110 | Chronic kidney disease stage 3 without proteinuria | 3 |
| 618441000000117 | 1Z1C.11 | 324281000000104 | 618441000000117 | CKD stage 3 without proteinuria | 3 |
| 8104261000006112 |  | 324281000000104 | 1676861000000110 | CKD (chronic kidney disease) stage 3 without proteinuria | 3 |
| 595931000000116 | 1Z1D.00 | 324311000000101 | 595931000000116 | Chronic kidney disease stage 3A with proteinuria | 3 |
| 618451000000119 | 1Z1D.11 | 324311000000101 | 618451000000119 | CKD stage 3A with proteinuria | 3 |
| 8104301000006115 |  | 324311000000101 | 1692921000000118 | CKD (chronic kidney disease) stage 3A with proteinuria | 3 |
| 595991000000115 | 1Z1E.00 | 324341000000100 | 595991000000115 | Chronic kidney disease stage 3A without proteinuria | 3 |
| 618461000000116 | 1Z1E.11 | 324341000000100 | 618461000000116 | CKD stage 3A without proteinuria | 3 |
| 8104351000006116 |  | 324341000000100 | 1692931000000116 | CKD (chronic kidney disease) stage 3A without proteinuria | 3 |
| 596051000000115 | 1Z1F.00 | 324371000000106 | 596051000000115 | Chronic kidney disease stage 3B with proteinuria | 3 |
| 618471000000111 | 1Z1F.11 | 324371000000106 | 618471000000111 | CKD stage 3B with proteinuria | 3 |
| 8104381000006112 |  | 324371000000106 | 1692941000000113 | CKD (chronic kidney disease) stage 3B with proteinuria | 3 |
| 596131000000114 | 1Z1G.00 | 324411000000105 | 596131000000114 | Chronic kidney disease stage 3B without proteinuria | 3 |
| 618481000000113 | 1Z1G.11 | 324411000000105 | 618481000000113 | CKD stage 3B without proteinuria | 3 |
| 8104411000006110 |  | 324411000000105 | 1692951000000111 | CKD (chronic kidney disease) stage 3B without proteinuria | 3 |
| 8212401000006115 |  | 691421000119108 | 3289583012 | Anaemia co-occurrent and due to chronic kidney disease stage 3 | 3 |
| 8212411000006117 |  | 691421000119108 | 3289658015 | Anemia co-occurrent and due to chronic kidney disease stage 3 | 3 |
| 8345881000006116 |  | 949881000000106 | 2427351000000116 | Chronic kidney disease with glomerular filtration rate category G3a and albuminuria category A1 | 3 |
| 1940621000006111 | 1Z1T.00 | 949881000000106 | 2427381000000110 | CKD G3aA1 - chronic kidney disease with glomerular filtration rate category G3a and albuminuria category A1 | 3 |
| 1940631000006114 | 1Z1V.00 | 949901000000109 | 2427401000000110 | CKD G3aA2 - chronic kidney disease with glomerular filtration rate category G3a and albuminuria category A2 | 3 |
| 8345901000006119 |  | 949901000000109 | 2427431000000116 | Chronic kidney disease with glomerular filtration rate category G3a and albuminuria category A2 | 3 |
| 1940641000006116 | 1Z1W.00 | 949921000000100 | 2427451000000111 | CKD G3aA3 - chronic kidney disease with glomerular filtration rate category G3a and albuminuria category A3 | 3 |
| 8345931000006110 |  | 949921000000100 | 2427481000000117 | Chronic kidney disease with glomerular filtration rate category G3a and albuminuria category A3 | 3 |
| 1940651000006119 | 1Z1X.00 | 950061000000103 | 2427751000000117 | CKD G3bA1 - chronic kidney disease with glomerular filtration rate category G3b and albuminuria category A1 | 3 |
| 8346001000006110 |  | 950061000000103 | 2427781000000111 | Chronic kidney disease with glomerular filtration rate category G3b and albuminuria category A1 | 3 |
| 1940661000006117 | 1Z1Y.00 | 950081000000107 | 2427801000000112 | CKD G3bA2 - chronic kidney disease with glomerular filtration rate category G3b and albuminuria category A2 | 3 |
| 8346021000006117 |  | 950081000000107 | 2427831000000118 | Chronic kidney disease with glomerular filtration rate category G3b and albuminuria category A2 | 3 |
| 1940671000006112 | 1Z1Z.00 | 950101000000101 | 2427851000000113 | CKD G3bA3 - chronic kidney disease with glomerular filtration rate category G3b and albuminuria category A3 | 3 |
| 8346041000006112 |  | 950101000000101 | 2427881000000119 | Chronic kidney disease with glomerular filtration rate category G3b and albuminuria category A3 | 3 |
| 994421000006111 |  | 994421000006107 | 994421000006111 | Chronic kidney disease stage 3 | 3 |
| 2767385013 | K054.00 | 431857002 | 2767385013 | Chronic kidney disease stage 4 | 4 |
| 304091000000116 | 1Z13.00 | 431857002 | 2767385013 | Chronic kidney disease stage 4 | 4 |
| 7147781000006115 |  | 431857002 | 2872614018 | CKD stage 4 | 4 |
| 7966651000006110 |  | 721000119107 | 3013038018 | Diabetic stage 4 chronic renal impairment due to type 2 diabetes mellitus | 4 |
| 7966641000006113 |  | 721000119107 | 3013100015 | Chronic kidney disease stage 4 due to type 2 diabetes mellitus | 4 |
| 7966661000006112 |  | 721000119107 | 3035100014 | Chronic kidney disease stage 4 due to type II diabetes mellitus | 4 |
| 8027471000006112 |  | 90751000119109 | 3013465019 | Chronic kidney disease stage 4 due to type 1 diabetes mellitus | 4 |
| 8027481000006110 |  | 90751000119109 | 3035115010 | Chronic kidney disease stage 4 due to type I diabetes mellitus | 4 |
| 8030421000006114 |  | 96721000119103 | 2968177010 | Hypertensive heart AND chronic kidney disease stage 4 | 4 |
| 8040661000006119 |  | 129151000119102 | 2968350014 | Chronic kidney disease stage 4 due to hypertension | 4 |
| 8044041000006118 |  | 140111000119107 | 3014010018 | Hypertension in chronic kidney disease stage 4 due to type 2 diabetes mellitus | 4 |
| 8044051000006116 |  | 140111000119107 | 3035138013 | Hypertension in chronic kidney disease stage 4 due to type II diabetes mellitus | 4 |
| 8087081000006110 |  | 285001000119105 | 3040829013 | Chronic kidney disease stage 4 due to benign hypertension | 4 |
| 8087701000006115 |  | 285881000119109 | 2922631010 | Malignant hypertensive chronic kidney disease stage 4 | 4 |
| 596191000000110 | 1Z1H.00 | 324441000000106 | 596191000000110 | Chronic kidney disease stage 4 with proteinuria | 4 |
| 618491000000110 | 1Z1H.11 | 324441000000106 | 618491000000110 | CKD stage 4 with proteinuria | 4 |
| 8104451000006111 |  | 324441000000106 | 1693001000000116 | CKD (chronic kidney disease) stage 4 with proteinuria | 4 |
| 596261000000111 | 1Z1J.00 | 324471000000100 | 596261000000111 | Chronic kidney disease stage 4 without proteinuria | 4 |
| 618501000000116 | 1Z1J.11 | 324471000000100 | 618501000000116 | CKD stage 4 without proteinuria | 4 |
| 8104481000006115 |  | 324471000000100 | 1693051000000115 | CKD (chronic kidney disease) stage 4 without proteinuria | 4 |
| 8212321000006119 |  | 691401000119104 | 3312506010 | Anemia co-occurrent and due to chronic kidney disease stage 4 | 4 |
| 8212331000006116 |  | 691401000119104 | 3312514016 | Anemia in chronic kidney disease stage 4 | 4 |
| 8212341000006114 |  | 691401000119104 | 3312515015 | Anaemia co-occurrent and due to chronic kidney disease stage 4 | 4 |
| 8212311000006110 |  | 691401000119104 | 3312516019 | Anaemia in chronic kidney disease stage 4 | 4 |
| 1940501000006119 | 1Z1a.00 | 950181000000106 | 2428021000000111 | CKD G4A1 - chronic kidney disease with glomerular filtration rate category G4 and albuminuria category A1 | 4 |
| 8346101000006114 |  | 950181000000106 | 2428071000000110 | Chronic kidney disease with glomerular filtration rate category G4 and albuminuria category A1 | 4 |
| 1940511000006116 | 1Z1b.00 | 950211000000107 | 2428091000000114 | CKD G4A2 - chronic kidney disease with glomerular filtration rate category G4 and albuminuria category A2 | 4 |
| 8346121000006116 |  | 950211000000107 | 2428121000000110 | Chronic kidney disease with glomerular filtration rate category G4 and albuminuria category A2 | 4 |
| 1940521000006112 | 1Z1c.00 | 950231000000104 | 2428141000000115 | CKD G4A3 - chronic kidney disease with glomerular filtration rate category G4 and albuminuria category A3 | 4 |
| 8346151000006113 |  | 950231000000104 | 2428171000000114 | Chronic kidney disease with glomerular filtration rate category G4 and albuminuria category A3 | 4 |
| 994431000006114 |  | 994431000006105 | 994431000006114 | Chronic kidney disease stage 4 | 4 |
| 641471000006117 | K05..12 | 46177005 | 76995019 | End stage renal failure | 5 |
| 215521000000116 | K050.00 | 46177005 | 76995019 | End stage renal disease | 5 |
| 3245841000006115 |  | 46177005 | 76996018 | End stage kidney disease | 5 |
| 641491000006116 | K0D..00 | 46177005 | 76996018 | End-stage renal disease | 5 |
| 3245851000006118 |  | 46177005 | 1230314019 | ESRF - End stage renal failure | 5 |
| 3245861000006116 |  | 46177005 | 1230315018 | ESCRF - End stage chronic renal failure | 5 |
| 3245871000006111 |  | 46177005 | 1230316017 | ESRD - End stage renal disease | 5 |
| 3245881000006114 |  | 46177005 | 2647096016 | End stage chronic renal failure | 5 |
| 12704471000006116 |  | 46177005 | 3517959016 | End-stage renal disease | 5 |
| 11924551000006114 |  | 46177005 | 11924551000006114 | End-stage renal disease | 5 |
| 5094151000006116 |  | 236434000 | 354418013 | End stage renal failure untreated by renal replacement therapy | 5 |
| 2767154014 | K055.00 | 433146000 | 2767154014 | Chronic kidney disease stage 5 | 5 |
| 304111000000114 | 1Z14.00 | 433146000 | 2767154014 | Chronic kidney disease stage 5 | 5 |
| 7174851000006111 |  | 433146000 | 2870834012 | CKD stage 5 | 5 |
| 7513101000006118 |  | 698810000 | 2981366013 | Hypertensive renal disease with end stage renal failure | 5 |
| 7691491000006110 |  | 712487000 | 3082248014 | End stage renal disease due to benign hypertension | 5 |
| 7691501000006119 |  | 712487000 | 3083579019 | ESRD (End stage renal disease) due to benign hypertension | 5 |
| 7714901000006119 |  | 714152005 | 3298467018 | Chronic kidney disease 5d | 5 |
| 7966611000006114 |  | 711000119100 | 3013043013 | Chronic kidney disease stage 5 due to type 2 diabetes mellitus | 5 |
| 7966621000006118 |  | 711000119100 | 3013094018 | Diabetic stage 5 chronic renal impairment due to type 2 diabetes mellitus | 5 |
| 7966631000006115 |  | 711000119100 | 3035119016 | Chronic kidney disease stage 5 due to type II diabetes mellitus | 5 |
| 8027491000006113 |  | 90761000119106 | 3013545012 | Chronic kidney disease stage 5 due to type 1 diabetes mellitus | 5 |
| 8027501000006117 |  | 90761000119106 | 3035095012 | Chronic kidney disease stage 5 due to type I diabetes mellitus | 5 |
| 8030411000006118 |  | 96711000119105 | 2968323016 | Hypertensive heart AND chronic kidney disease stage 5 | 5 |
| 8035251000006111 |  | 111411000119103 | 3288969010 | End stage renal disease due to hypertension | 5 |
| 8035261000006113 |  | 111411000119103 | 3289001017 | ESRD (End stage renal disease) due to hypertension | 5 |
| 8040671000006114 |  | 129161000119100 | 2968176018 | Chronic kidney disease stage 5 due to hypertension | 5 |
| 8044011000006117 |  | 140101000119109 | 3013074010 | Hypertension in chronic kidney disease stage 5 due to type 2 diabetes mellitus | 5 |
| 8044021000006113 |  | 140101000119109 | 3035151015 | Hypertension in chronic kidney disease stage 5 due to type II diabetes mellitus | 5 |
| 8047971000006118 |  | 153851000119106 | 2922196018 | Malignant hypertensive chronic kidney disease stage 5 | 5 |
| 8087091000006113 |  | 285011000119108 | 3040952011 | Chronic kidney disease stage 5 due to benign hypertension | 5 |
| 8087651000006114 |  | 285841000119104 | 2922202015 | Malignant hypertensive end stage renal disease | 5 |
| 596321000000110 | 1Z1K.00 | 324501000000107 | 596321000000110 | Chronic kidney disease stage 5 with proteinuria | 5 |
| 618511000000119 | 1Z1K.11 | 324501000000107 | 618511000000119 | CKD stage 5 with proteinuria | 5 |
| 8104511000006111 |  | 324501000000107 | 1693061000000117 | CKD (chronic kidney disease) stage 5 with proteinuria | 5 |
| 596401000000111 | 1Z1L.00 | 324541000000105 | 596401000000111 | Chronic kidney disease stage 5 without proteinuria | 5 |
| 618521000000113 | 1Z1L.11 | 324541000000105 | 618521000000113 | CKD stage 5 without proteinuria | 5 |
| 8104551000006112 |  | 324541000000105 | 1692911000000112 | CKD (chronic kidney disease) stage 5 without proteinuria | 5 |
| 1940531000006110 | 1Z1d.00 | 950251000000106 | 2428191000000113 | CKD G5A1 - chronic kidney disease with glomerular filtration rate category G5 and albuminuria category A1 | 5 |
| 8346171000006115 |  | 950251000000106 | 2428261000000114 | Chronic kidney disease with glomerular filtration rate category G5 and albuminuria category A1 | 5 |
| 1940541000006117 | 1Z1e.00 | 950291000000103 | 2428281000000117 | CKD G5A2 - chronic kidney disease with glomerular filtration rate category G5 and albuminuria category A2 | 5 |
| 8346201000006116 |  | 950291000000103 | 2428311000000119 | Chronic kidney disease with glomerular filtration rate category G5 and albuminuria category A2 | 5 |
| 1940551000006115 | 1Z1f.00 | 950311000000102 | 2428331000000110 | CKD G5A3 - chronic kidney disease with glomerular filtration rate category G5 and albuminuria category A3 | 5 |
| 8346221000006114 |  | 950311000000102 | 2428361000000117 | Chronic kidney disease with glomerular filtration rate category G5 and albuminuria category A3 | 5 |
| 994441000006116 |  | 994441000006100 | 994441000006116 | Chronic kidney disease stage 5 | 5 |
| 3183501000006111 |  | 42399005 | 70742017 | Renal failure syndrome | Unspecified |
| 396711013 | K06..00 | 42399005 | 1229860014 | RF - Renal failure | Unspecified |
| 1816581000006110 | K06..12 | 42399005 | 1229860014 | Kidney failure unspecified | Unspecified |
| 424731000006111 | Kyu2.00 | 42399005 | 3014127011 | Renal failure | Unspecified |
| 3221331000006116 |  | 44730006 | 74620017 | Uremia | Unspecified |
| 73171000006118 | K06..11 | 44730006 | 494005015 | Uraemia | Unspecified |
| 299665018 | G222.00 | 49220004 | 81984019 | Hypertensive renal disease with renal failure | Unspecified |
| 3296351000006114 |  | 49220004 | 81984019 | Hypertensive renal failure | Unspecified |
| 3304611000006115 |  | 49708008 | 82792016 | Anemia of chronic renal failure | Unspecified |
| 480871000006117 | D215000 | 49708008 | 495474017 | Anaemia of chronic renal failure | Unspecified |
| 3304621000006111 |  | 49708008 | 2969200013 | Anemia of chronic renal insufficiency | Unspecified |
| 3304631000006114 |  | 49708008 | 2969201012 | Anaemia of chronic renal insufficiency | Unspecified |
| 303855018 | K01z.00 | 52254009 | 86957016 | Nephrotic syndrome NOS | Unspecified |
| 86957016 | K01..00 | 52254009 | 86957016 | Nephrotic syndrome | Unspecified |
| 3347091000006118 |  | 52254009 | 1231098019 | NS - Nephrotic syndrome | Unspecified |
| 3566321000006113 |  | 65579002 | 108953014 | Chronic renal failure diet | Unspecified |
| 3738091000006117 |  | 76114004 | 126416014 | Decreased renal function | Unspecified |
| 3738101000006111 |  | 76114004 | 3013823015 | Decreased kidney function | Unspecified |
| 3973811000006118 |  | 90688005 | 150286014 | Chronic renal failure syndrome | Unspecified |
| 509108014 | K05..11 | 90688005 | 509108014 | Chronic uraemia | Unspecified |
| 3973831000006112 |  | 90688005 | 509109018 | Chronic uremia | Unspecified |
| 3973841000006119 |  | 90688005 | 509110011 | CRF - Chronic renal failure | Unspecified |
| 305192017 | Kyu2100 | 90688005 | 509111010 | [X]Other chronic renal failure | Unspecified |
| 509111010 | K05..00 | 90688005 | 509111010 | Chronic renal failure | Unspecified |
| 4203621000006116 |  | 112066009 | 179509012 | Absent renal function | Unspecified |
| 188004010 | K13z000 | 155876003 | 243006012 | Non-functioning kidney | Unspecified |
| 4515021000006116 |  | 155876003 | 3034376018 | NFK - Non-functioning kidney | Unspecified |
| 11926091000006118 |  | 194774006 | 299665018 | Hypertensive renal disease with renal failure | Unspecified |
| 299673010 | G233.00 | 194780003 | 299673010 | Hypertensive heart and renal disease with renal failure | Unspecified |
| 789941000006117 | G234.00 | 194781004 | 299674016 | Hypertensive heart and renal disease with both (congestive) heart failure and renal failure | Unspecified |
| 887731000006115 | L093.99 | 198841006 | 887731000006115 | Renal failure | Unspecified |
| 354406016 | K060.00 | 236423003 | 354406016 | Renal impairment | Unspecified |
| 354407013 | K060.11 | 236423003 | 354407013 | Impaired renal function | Unspecified |
| 5094031000006110 |  | 236423003 | 3013972011 | Renal dysfunction | Unspecified |
| 354417015 | K0E..00 | 236433006 | 354417015 | Acute-on-chronic renal failure | Unspecified |
| 5094141000006118 |  | 236433006 | 3037716017 | Acute on chronic renal failure | Unspecified |
| 5481301000006114 |  | 264867001 | 393369011 | Nephrotic range proteinuria | Unspecified |
| 459420012 | 4519.00 | 314983004 | 459420012 | Deteriorating renal function | Unspecified |
| 6858011000006117 |  | 414417004 | 2535178010 | History of renal failure | Unspecified |
| 7043231000006112 |  | 425369003 | 2645799018 | Chronic progressive renal failure | Unspecified |
| 7043241000006119 |  | 425369003 | 3035617018 | Chronic progressive renal insufficiency | Unspecified |
| 7303111000006115 |  | 445120004 | 2871022012 | Uraemia due to inadequate renal perfusion | Unspecified |
| 7303121000006111 |  | 445120004 | 2871023019 | Uremia due to inadequate renal perfusion | Unspecified |
| 7480381000006111 |  | 472953006 | 2952277011 | History of chronic kidney disease | Unspecified |
| 7480391000006114 |  | 472953006 | 2952295014 | History of chronic renal impairment | Unspecified |
| 7480371000006113 |  | 472953006 | 2244571000000110 | H/O chronic kidney disease | Unspecified |
| 7510311000006111 |  | 698591006 | 2974754013 | Benign hypertensive renal disease with renal failure | Unspecified |
| 2771041011 | K05..13 | 709044004 | 3036750018 | Chronic kidney disease | Unspecified |
| 7643311000006115 |  | 709044004 | 3037959015 | CKD - chronic kidney disease | Unspecified |
| 354409011 | 1Z1..00 | 709044004 | 3037970010 | Chronic renal impairment | Unspecified |
| 7643331000006114 |  | 709044004 | 3038200016 | Chronic renal disease | Unspecified |
| 7841541000006119 |  | 723188008 | 3334970017 | Renal insufficiency | Unspecified |
| 7841551000006117 |  | 723188008 | 3334971018 | Renal insufficiency syndrome | Unspecified |
| 7841571000006110 |  | 723190009 | 3334975010 | Chronic renal insufficiency | Unspecified |
| 7966881000006115 |  | 771000119108 | 3013064012 | Chronic renal impairment due to type II diabetes mellitus | Unspecified |
| 7966891000006117 |  | 771000119108 | 3013087016 | Diabetic chronic renal impairment due to type 2 diabetes mellitus | Unspecified |
| 7966871000006118 |  | 771000119108 | 3013487011 | Chronic kidney disease due to type 2 diabetes mellitus | Unspecified |
| 7966901000006118 |  | 771000119108 | 3013552014 | Chronic renal impairment due to type 2 diabetes mellitus | Unspecified |
| 7970471000006113 |  | 1801000119106 | 2920810016 | Anemia, pre-ESRD erythropoietin protocol | Unspecified |
| 7970461000006118 |  | 1801000119106 | 2921010011 | Anemia, pre-end stage renal disease on erythropoietin protocol | Unspecified |
| 7990031000006110 |  | 8501000119104 | 2981284012 | Hypertensive heart and chronic kidney disease | Unspecified |
| 8001631000006110 |  | 15781000119107 | 2985876012 | Hypertensive heart AND chronic kidney disease with congestive heart failure | Unspecified |
| 8022361000006113 |  | 71421000119105 | 3014033017 | Hypertension in chronic kidney disease due to type 2 diabetes mellitus | Unspecified |
| 8022371000006118 |  | 71421000119105 | 3035111018 | Hypertension in chronic kidney disease due to type II diabetes mellitus | Unspecified |
| 8022381000006115 |  | 71441000119104 | 3013377011 | Nephrotic syndrome due to type 2 diabetes mellitus | Unspecified |
| 8022401000006115 |  | 71701000119105 | 3013910011 | Hypertension in chronic kidney disease due to type 1 diabetes mellitus | Unspecified |
| 8022411000006117 |  | 71701000119105 | 3035136012 | Hypertension in chronic kidney disease due to type I diabetes mellitus | Unspecified |
| 8022431000006111 |  | 71721000119101 | 3013474017 | Nephrotic syndrome due to type 1 diabetes mellitus | Unspecified |
| 3517310014 |  | 73901000119107 | 3517310014 | History of renal insufficiency | Unspecified |
| 3517311013 |  | 73901000119107 | 3517311013 | History of reduced function of kidney | Unspecified |
| 8030331000006117 |  | 96441000119101 | 3013075011 | Chronic kidney disease due to type 1 diabetes mellitus | Unspecified |
| 8030341000006110 |  | 96441000119101 | 3035113015 | Chronic kidney disease due to type I diabetes mellitus | Unspecified |
| 8032721000006117 |  | 104931000119100 | 2983529012 | Chronic kidney disease due to hypertension | Unspecified |
| 8087041000006116 |  | 284961000119106 | 3040880014 | Chronic kidney disease due to benign hypertension | Unspecified |
| 8087641000006112 |  | 285831000119108 | 2922212010 | Malignant hypertensive chronic kidney disease | Unspecified |
| 886631000006110 | K06..99 | 690191000000102 | 886631000006110 | Renal failure NOS | Unspecified |
| 12728651000006112 |  | 690191000000102 | 1510531000000118 | Renal failure unspecified | Unspecified |
| 853271000006110 |  | 853271000006106 | 853271000006110 | Non-functioning kidney | Unspecified |
| 906301000006115 |  | 906301000006104 | 906301000006115 | [RFC] Renal probs including renal failure | Unspecified |
| 994391000006115 |  | 994391000006104 | 994391000006115 | Chronic kidney disease stage | Unspecified |
| 1726511000006111 |  | 1726511000006107 | 1726511000006111 | Acute-on-chronic renal failure | Unspecified |
| 1823941000006114 |  | 1823941000006105 | 1823941000006114 | Chronic kidney disease confirmed | Unspecified |

## **ICD10 codes used to identify CKD in HES Admitted Patient Care data**

| icd | description | CKD stage |
| --- | --- | --- |
| N18.1 | Chronic kidney disease, stage 1 | 1 |
| N18.2 | Chronic kidney disease, stage 2 (mild) | 2 |
| N18.3 | Chronic kidney disease, stage 3 (moderate) | 3 |
| N18.4 | Chronic kidney disease, stage 4 | 4 |
| I12.0 | Hypertensive chronic kidney disease with stage 5 chronic kidney disease or end stage renal disease | 5 |
| I13.11 | Hypertensive heart and chronic kidney disease without heart failure, with stage 5 chronic kidney disease, or end stage renal disease | 5 |
| I13.2 | Hypertensive heart and chronic kidney disease with heart failure and with stage 5 chronic kidney disease, or end stage renal disease | 5 |
| N18.0 | End-stage renal disease | 5 |
| N18.5 | Chronic kidney disease, stage 5 | 5 |
| N18.6 | End stage renal disease | 5 |
| N26.1 | Atrophy of kidney (terminal) | 5 |
| A18.11 | Tuberculosis of kidney and ureter | Unspecified |
| A52.75 | Syphilis of kidney and ureter | Unspecified |
| B52.0 | Plasmodium malariae malaria with nephropathy | Unspecified |
| E08.2 | Diabetes mellitus due to underlying condition with kidney complications | Unspecified |
| E09.2 | Drug or chemical induced diabetes mellitus with kidney complications | Unspecified |
| E10.2 | Type 1 diabetes mellitus with kidney complications | Unspecified |
| E11.2 | Type 2 diabetes mellitus with kidney complications | Unspecified |
| E13.2 | Other specified diabetes mellitus with kidney complications | Unspecified |
| I12 | Hypertensive chronic kidney disease | Unspecified |
| I12.9 | Hypertensive chronic kidney disease with stage 1 through stage 4 chronic kidney disease, or unspecified chronic kidney disease | Unspecified |
| I13 | Hypertensive heart and chronic kidney disease | Unspecified |
| I13.0 | Hypertensive heart and chronic kidney disease with heart failure and stage 1 through stage 4 chronic kidney disease, or unspecified chronic kidney disease | Unspecified |
| I13.10 | Hypertensive heart and chronic kidney disease without heart failure, with stage 1 through stage 4 chronic kidney disease, or unspecified chronic kidney disease | Unspecified |
| I13.9 | Hypertensive heart and renal disease, unspecified | Unspecified |
| K76.7 | Hepatorenal syndrome | Unspecified |
| M32.14 | Glomerular disease in systemic lupus erythematosus | Unspecified |
| M32.15 | Tubulo-interstitial nephropathy in systemic lupus erythematosus | Unspecified |
| N01.0 | Rapidly progressive nephritic syndrome with minor glomerular abnormality | Unspecified |
| N01.2 | Rapidly progressive nephritic syndrome with diffuse membranous glomerulonephritis | Unspecified |
| N01.3 | Rapidly progressive nephritic syndrome with diffuse mesangial proliferative glomerulonephritis | Unspecified |
| N01.4 | Rapidly progressive nephritic syndrome with diffuse endocapillary proliferative glomerulonephritis | Unspecified |
| N01.5 | Rapidly progressive nephritic syndrome with diffuse mesangiocapillary glomerulonephritis | Unspecified |
| N01.6 | Rapidly progressive nephritic syndrome with dense deposit disease | Unspecified |
| N01.7 | Rapidly progressive nephritic syndrome with diffuse crescentic glomerulonephritis | Unspecified |
| N01.8 | Rapidly progressive nephritic syndrome with other morphologic changes | Unspecified |
| N01.9 | Rapidly progressive nephritic syndrome with unspecified morphologic changes | Unspecified |
| N02.0 | Recurrent and persistent hematuria with minor glomerular abnormality | Unspecified |
| N02.1 | Recurrent and persistent hematuria with focal and segmental glomerular lesions | Unspecified |
| N02.2 | Recurrent and persistent hematuria with diffuse membranous glomerulonephritis | Unspecified |
| N02.3 | Recurrent and persistent hematuria with diffuse mesangial proliferative glomerulonephritis | Unspecified |
| N02.4 | Recurrent and persistent hematuria with diffuse endocapillary proliferative glomerulonephritis | Unspecified |
| N02.5 | Recurrent and persistent hematuria with diffuse mesangiocapillary glomerulonephritis | Unspecified |
| N02.6 | Recurrent and persistent hematuria with dense deposit disease | Unspecified |
| N02.7 | Recurrent and persistent hematuria with diffuse crescentic glomerulonephritis | Unspecified |
| N02.8 | Recurrent and persistent hematuria with other morphologic changes | Unspecified |
| N02.9 | Recurrent and persistent hematuria with unspecified morphologic changes | Unspecified |
| N03.0 | Chronic nephritic syndrome with minor glomerular abnormality | Unspecified |
| N03.1 | Chronic nephritic syndrome with focal and segmental glomerular lesions | Unspecified |
| N03.2 | Chronic nephritic syndrome with diffuse membranous glomerulonephritis | Unspecified |
| N03.3 | Chronic nephritic syndrome with diffuse mesangial proliferative glomerulonephritis | Unspecified |
| N03.4 | Chronic nephritic syndrome with diffuse endocapillary proliferative glomerulonephritis | Unspecified |
| N03.5 | Chronic nephritic syndrome with diffuse mesangiocapillary glomerulonephritis | Unspecified |
| N03.6 | Chronic nephritic syndrome with dense deposit disease | Unspecified |
| N03.7 | Chronic nephritic syndrome with diffuse crescentic glomerulonephritis | Unspecified |
| N03.8 | Chronic nephritic syndrome with other morphologic changes | Unspecified |
| N03.9 | Chronic nephritic syndrome with unspecified morphologic changes | Unspecified |
| N04.0 | Nephrotic syndrome with minor glomerular abnormality | Unspecified |
| N04.1 | Nephrotic syndrome with focal and segmental glomerular lesions | Unspecified |
| N04.2 | Nephrotic syndrome with diffuse membranous glomerulonephritis | Unspecified |
| N04.3 | Nephrotic syndrome with diffuse mesangial proliferative glomerulonephritis | Unspecified |
| N04.4 | Nephrotic syndrome with diffuse endocapillary proliferative glomerulonephritis | Unspecified |
| N04.5 | Nephrotic syndrome with diffuse mesangiocapillary glomerulonephritis | Unspecified |
| N04.6 | Nephrotic syndrome with dense deposit disease | Unspecified |
| N04.7 | Nephrotic syndrome with diffuse crescentic glomerulonephritis | Unspecified |
| N04.8 | Nephrotic syndrome with other morphologic changes | Unspecified |
| N04.9 | Nephrotic syndrome with unspecified morphologic changes | Unspecified |
| N05.0 | Unspecified nephritic syndrome with minor glomerular abnormality | Unspecified |
| N05.1 | Unspecified nephritic syndrome with focal and segmental glomerular lesions | Unspecified |
| N05.2 | Unspecified nephritic syndrome with diffuse membranous glomerulonephritis | Unspecified |
| N05.3 | Unspecified nephritic syndrome with diffuse mesangial proliferative glomerulonephritis | Unspecified |
| N05.4 | Unspecified nephritic syndrome with diffuse endocapillary proliferative glomerulonephritis | Unspecified |
| N05.5 | Unspecified nephritic syndrome with diffuse mesangiocapillary glomerulonephritis | Unspecified |
| N05.6 | Unspecified nephritic syndrome with dense deposit disease | Unspecified |
| N05.7 | Unspecified nephritic syndrome with diffuse crescentic glomerulonephritis | Unspecified |
| N05.8 | Unspecified nephritic syndrome with other morphologic changes | Unspecified |
| N05.9 | Unspecified nephritic syndrome with unspecified morphologic changes | Unspecified |
| N06.0 | Isolated proteinuria with minor glomerular abnormality | Unspecified |
| N06.1 | Isolated proteinuria with focal and segmental glomerular lesions | Unspecified |
| N06.2 | Isolated proteinuria with diffuse membranous glomerulonephritis | Unspecified |
| N06.3 | Isolated proteinuria with diffuse mesangial proliferative glomerulonephritis | Unspecified |
| N06.4 | Isolated proteinuria with diffuse endocapillary proliferative glomerulonephritis | Unspecified |
| N06.5 | Isolated proteinuria with diffuse mesangiocapillary glomerulonephritis | Unspecified |
| N06.6 | Isolated proteinuria with dense deposit disease | Unspecified |
| N06.7 | Isolated proteinuria with diffuse crescentic glomerulonephritis | Unspecified |
| N06.8 | Isolated proteinuria with other morphologic lesion | Unspecified |
| N06.9 | Isolated proteinuria with unspecified morphologic lesion | Unspecified |
| N07.0 | Hereditary nephropathy, not elsewhere classified with minor glomerular abnormality | Unspecified |
| N07.1 | Hereditary nephropathy, not elsewhere classified with focal and segmental glomerular lesions | Unspecified |
| N07.2 | Hereditary nephropathy, not elsewhere classified with diffuse membranous glomerulonephritis | Unspecified |
| N07.3 | Hereditary nephropathy, not elsewhere classified with diffuse mesangial proliferative glomerulonephritis | Unspecified |
| N07.4 | Hereditary nephropathy, not elsewhere classified with diffuse endocapillary proliferative glomerulonephritis | Unspecified |
| N07.5 | Hereditary nephropathy, not elsewhere classified with diffuse mesangiocapillary glomerulonephritis | Unspecified |
| N07.6 | Hereditary nephropathy, not elsewhere classified with dense deposit disease | Unspecified |
| N07.7 | Hereditary nephropathy, not elsewhere classified with diffuse crescentic glomerulonephritis | Unspecified |
| N07.8 | Hereditary nephropathy, not elsewhere classified with other morphologic lesions | Unspecified |
| N07.9 | Hereditary nephropathy, not elsewhere classified with unspecified morphologiclesions | Unspecified |
| N08. | Glomerular disorders in diseases classified elsewhere | Unspecified |
| N13.1 | Hydronephrosis with ureteral stricture, not elsewhere classified | Unspecified |
| N13.2 | Hydronephrosis with renal and ureteral calculous obstruction | Unspecified |
| N13.3 | Other and unspecified hydronephrosis | Unspecified |
| N13.4 | Hydroureter | Unspecified |
| N13.5 | Crossing vessel and stricture of ureter without hydronephrosis | Unspecified |
| N13.6 | Pyonephrosis | Unspecified |
| N13.7 | Vesicoureteral-reflux | Unspecified |
| N13.8 | Other obstructive and reflux uropathy | Unspecified |
| N13.9 | Obstructive and reflux uropathy, unspecified | Unspecified |
| N14.0 | Analgesic nephropathy | Unspecified |
| N14.1 | Nephropathy induced by other drugs, medicaments and biological substances | Unspecified |
| N14.2 | Nephropathy induced by unspecified drug, medicament or biological substance | Unspecified |
| N14.3 | Nephropathy induced by heavy metals | Unspecified |
| N14.4 | Toxic nephropathy, not elsewhere classified | Unspecified |
| N15.0 | Balkan nephropathy | Unspecified |
| N15.8 | Other specified renal tubulo-interstitial diseases | Unspecified |
| N15.9 | Renal tubulo-interstitial disease, unspecified | Unspecified |
| N16. | Renal tubulo-interstitial disorders in diseases classified elsewhere | Unspecified |
| N16.0 | Renal tubulo-interstitial disorders in infectious and parasitic diseases classified elsewhere | Unspecified |
| N16.1 | Renal tubulo-interstitial disorders in neoplastic diseases | Unspecified |
| N16.2 | Renal tubulo-interstitial disorders in blood diseases and disorders involving the immune mechanism | Unspecified |
| N16.3 | Renal tubulo-interstitial disorders in metabolic diseases | Unspecified |
| N16.4 | Renal tubulo-interstitial disorders in systemic connective tissue disorders | Unspecified |
| N16.5 | Renal tubulo-interstitial disorders in transplant rejection | Unspecified |
| N16.8 | Renal tubulo-interstitial disorders in other diseases classified elsewhere | Unspecified |
| N18 | Chronic kidney disease (CKD) | Unspecified |
| N18.8 | Other chronic renal failure | Unspecified |
| N18.9 | Chronic kidney disease, unspecified | Unspecified |
| N19. | Unspecified kidney failure | Unspecified |
| N25.0 | Renal osteodystrophy | Unspecified |
| N25.1 | Nephrogenic diabetes insipidus | Unspecified |
| N25.8 | Other disorders resulting from impaired renal tubular function | Unspecified |
| N25.9 | Disorder resulting from impaired renal tubular function, unspecified | Unspecified |
| N26.9 | Renal sclerosis, unspecified | Unspecified |
| Q61.1 | Polycystic kidney, infantile type | Unspecified |
| Q61.2 | Polycystic kidney, adult type | Unspecified |
| Q61.3 | Polycystic kidney, unspecified | Unspecified |
| Q61.4 | Renal dysplasia | Unspecified |
| Q61.5 | Medullary cystic kidney | Unspecified |
| Q61.8 | Other cystic kidney diseases | Unspecified |

## **Clinical codes used to identify diabetes in CPRD Aurum**

| medcodeid | readcode | snomedctconceptid | snomedctdescriptionid | term | type |
| --- | --- | --- | --- | --- | --- |
| 913651000006115 | C10E712 | 4855003 | 9093013 | Insulin dependent diabetes mellitus with retinopathy | T1 |
| 771411000006117 | C108700 | 4855003 | 9093013 | Insulin dependent diabetes mellitus with retinopathy | T1 |
| 429972014 | C10E412 | 11530004 | 19931010 | Unstable insulin dependent diabetes mellitus | T1 |
| 72651000006114 | C108400 | 11530004 | 19931010 | Unstable insulin dependent diabetes mellitus | T1 |
| 11927551000006115 |  | 19429009 | 913591000006110 | Insulin dependent diabetes mellitus with ulcer | T1 |
| 913471000006110 | C10E112 | 25093002 | 3688467012 | Insulin-dependent diabetes mellitus with ophthalmic comps | T1 |
| 771491000006110 | C108100 | 25093002 | 3688467012 | Insulin-dependent diabetes mellitus with ophthalmic comps | T1 |
| 459293018 | C10EH12 | 39710007 | 1229552016 | Insulin dependent diabetes mellitus with arthropathy | T1 |
| 771331000006116 | C108H00 | 39710007 | 1229552016 | Insulin dependent diabetes mellitus with arthropathy | T1 |
| 913891000006113 | C10EF12 | 43959009 | 73294018 | Insulin dependent diabetes mellitus with diabetic cataract | T1 |
| 771341000006114 | C108F00 | 43959009 | 73294018 | Insulin dependent diabetes mellitus with diabetic cataract | T1 |
| 197984010 | C10E.00 | 46635009 | 197984010 | Type 1 diabetes mellitus | T1 |
| 84281000006115 | C108.12 | 46635009 | 197984010 | Type 1 diabetes mellitus | T1 |
| 84651000006114 | C108.13 | 46635009 | 494564012 | Type I diabetes mellitus | T1 |
| 494564012 | C10E.11 | 46635009 | 494564012 | Type I diabetes mellitus | T1 |
| 913801000006118 | C10EC12 | 49455004 | 82373015 | Insulin dependent diabetes mellitus with polyneuropathy | T1 |
| 771401000006115 | C108C00 | 49455004 | 82373015 | Insulin dependent diabetes mellitus with polyneuropathy | T1 |
| 77727018 | C10E.12 | 73211009 | 121589010 | Insulin dependent diabetes mellitus | T1 |
| 772151000006116 | C108900 | 73211009 | 121589010 | Insulin dependent diabetes maturity onset | T1 |
| 787111000006112 | C108.11 | 73211009 | 121589010 | IDDM-Insulin dependent diabetes mellitus | T1 |
| 913711000006112 | C10E912 | 73211009 | 121589010 | Insulin dependent diabetes maturity onset | T1 |
| 772171000006114 | C108.00 | 73211009 | 121589010 | Insulin dependent diabetes mellitus | T1 |
| 616681000006116 | C10y000 | 73211009 | 121589010 | Diabetes mellitus, juvenile, + other specified manifestation | T1 |
| 772161000006119 | C100011 | 73211009 | 121589010 | Insulin dependent diabetes mellitus | T1 |
| 881441000006111 | C100099 | 73211009 | 881441000006111 | Diabetes mellitus - juvenile | T1 |
| 457327017 | C10EA12 | 111552007 | 178796018 | Insulin-dependent diabetes without complication | T1 |
| 746791000006111 | C108A00 | 111552007 | 178796018 | Insulin-dependent diabetes without complication | T1 |
| 913831000006114 | C10ED12 | 127013003 | 301016 | Insulin dependent diabetes mellitus with nephropathy | T1 |
| 771501000006119 | C108000 | 127013003 | 301016 | Insulin-dependent diabetes mellitus with renal complications | T1 |
| 913441000006119 | C10E012 | 127013003 | 301016 | Insulin-dependent diabetes mellitus with renal complications | T1 |
| 771391000006117 | C108D00 | 127013003 | 301016 | Insulin dependent diabetes mellitus with nephropathy | T1 |
| 292482014 | C102000 | 190330002 | 292482014 | Diabetes mellitus, juvenile type, with hyperosmolar coma | T1 |
| 292538019 | C10E511 | 190368000 | 292538019 | Type I diabetes mellitus with ulcer | T1 |
| 84821000006118 | C108511 | 190368000 | 292538019 | Type I diabetes mellitus with ulcer | T1 |
| 84451000006112 | C108512 | 190368000 | 292540012 | Type 1 diabetes mellitus with ulcer | T1 |
| 292540012 | C10E500 | 190368000 | 292540012 | Type 1 diabetes mellitus with ulcer | T1 |
| 12489581000006110 |  | 190369008 | 292541011 | Type 1 diabetes mellitus with gangrene | T1 |
| 12450761000006110 |  | 190369008 | 292543014 | Type I diabetes mellitus with gangrene | T1 |
| 84671000006116 | C108911 | 190372001 | 292551012 | Type I diabetes mellitus maturity onset | T1 |
| 292551012 | C10E911 | 190372001 | 292551012 | Type I diabetes mellitus maturity onset | T1 |
| 292553010 | C10E900 | 190372001 | 292553010 | Type 1 diabetes mellitus maturity onset | T1 |
| 84301000006116 | C108912 | 190372001 | 292553010 | Type 1 diabetes mellitus maturity onset | T1 |
| 772131000006111 | C108J00 | 201724008 | 309740011 | Insulin dependent diab mell with neuropathic arthropathy | T1 |
| 913981000006115 | C10EJ12 | 201724008 | 309740011 | Insulin dependent diab mell with neuropathic arthropathy | T1 |
| 771371000006118 | C108B00 | 230577008 | 345492010 | Insulin dependent diabetes mellitus with mononeuropathy | T1 |
| 913771000006115 | C10EB12 | 230577008 | 345492010 | Insulin dependent diabetes mellitus with mononeuropathy | T1 |
| 771361000006113 | C108E00 | 237632004 | 356141011 | Insulin dependent diabetes mellitus with hypoglycaemic coma | T1 |
| 459162010 | C10EE12 | 237632004 | 356141011 | Insulin dependent diabetes mellitus with hypoglycaemic coma | T1 |
| 772181000006112 | C108800 | 268519009 | 401531012 | Insulin dependent diabetes mellitus - poor control | T1 |
| 913681000006111 | C10E812 | 268519009 | 401531012 | Insulin dependent diabetes mellitus - poor control | T1 |
| 72721000006113 | C108411 | 290002008 | 429970018 | Unstable type I diabetes mellitus | T1 |
| 429970018 | C10E411 | 290002008 | 429970018 | Unstable type I diabetes mellitus | T1 |
| 72711000006117 | C108412 | 290002008 | 429971019 | Unstable type 1 diabetes mellitus | T1 |
| 429971019 | C10E400 | 290002008 | 429971019 | Unstable type 1 diabetes mellitus | T1 |
| 457325013 | C10EA11 | 313435000 | 457325013 | Type I diabetes mellitus without complication | T1 |
| 84831000006115 | C108A11 | 313435000 | 457325013 | Type I diabetes mellitus without complication | T1 |
| 84461000006114 | C108A12 | 313435000 | 457326014 | Type 1 diabetes mellitus without complication | T1 |
| 616621000006115 | C100000 | 313435000 | 457326014 | Type 1 diabetes mellitus without complication | T1 |
| 457326014 | C10EA00 | 313435000 | 457326014 | Type 1 diabetes mellitus without complication | T1 |
| 6030001000006116 |  | 313435000 | 457327017 | Insulin-dependent diabetes without complication | T1 |
| 459161015 | C10EE00 | 314771006 | 459161015 | Type 1 diabetes mellitus with hypoglycaemic coma | T1 |
| 84341000006119 | C108E12 | 314771006 | 459161015 | Type 1 diabetes mellitus with hypoglycaemic coma | T1 |
| 6050041000006115 |  | 314771006 | 459162010 | Insulin dependent diabetes mellitus with hypoglycaemic coma | T1 |
| 459163017 | C10EE11 | 314771006 | 459163017 | Type I diabetes mellitus with hypoglycaemic coma | T1 |
| 84711000006117 | C108E11 | 314771006 | 459163017 | Type I diabetes mellitus with hypoglycaemic coma | T1 |
| 459292011 | C10EH00 | 314893005 | 459292011 | Type 1 diabetes mellitus with arthropathy | T1 |
| 84311000006118 | C108H12 | 314893005 | 459292011 | Type 1 diabetes mellitus with arthropathy | T1 |
| 6051171000006117 |  | 314893005 | 459293018 | Insulin dependent diabetes mellitus with arthropathy | T1 |
| 84681000006118 | C108H11 | 314893005 | 459294012 | Type I diabetes mellitus with arthropathy | T1 |
| 459294012 | C10EH11 | 314893005 | 459294012 | Type I diabetes mellitus with arthropathy | T1 |
| 1780311019 | C10EL00 | 401110002 | 1780311019 | Type 1 diabetes mellitus with persistent microalbuminuria | T1 |
| 928491000006110 | C10EL11 | 401110002 | 1780311019 | Type I diabetes mellitus with persistent microalbuminuria | T1 |
| 616641000006110 | C101000 | 420270002 | 2618216015 | Diabetes mellitus, juvenile type, with ketoacidosis | T1 |
| 928501000006119 | C10EM00 | 420270002 | 2967817017 | Ketoacidosis in type 1 diabetes mellitus | T1 |
| 928511000006116 | C10EM11 | 420270002 | 2967817017 | Type I diabetes mellitus with ketoacidosis | T1 |
| 938311000006112 | C10EP11 | 420486006 | 2618236019 | Type I diabetes mellitus with exudative maculopathy | T1 |
| 938301000006114 | C10EP00 | 420486006 | 3698521013 | Exudative maculopathy with type 1 diabetes mellitus | T1 |
| 12331221000006112 |  | 420486006 | 12331221000006112 | Exudative maculopathy with type 1 diabetes mellitus | T1 |
| 928461000006119 | C10EK00 | 420514000 | 2967914011 | Persistent proteinuria associated with type 1 diabetes mellitus | T1 |
| 928471000006114 | C10EK11 | 420514000 | 2967914011 | Type I diabetes mellitus with persistent proteinuria | T1 |
| 913661000006118 | C10E700 | 420789003 | 3699407019 | Retinopathy with type 1 diabetes mellitus | T1 |
| 84811000006114 | C108711 | 420789003 | 3699407019 | Type I diabetes mellitus with retinopathy | T1 |
| 84441000006110 | C108712 | 420789003 | 3699407019 | Type 1 diabetes mellitus with retinopathy | T1 |
| 913671000006113 | C10E711 | 420789003 | 3699407019 | Type I diabetes mellitus with retinopathy | T1 |
| 12332271000006117 |  | 420789003 | 12332271000006117 | Retinopathy with type 1 diabetes mellitus | T1 |
| 84331000006112 | C108612 | 420825003 | 2618207019 | Type 1 diabetes mellitus with gangrene | T1 |
| 84701000006115 | C108611 | 420825003 | 2618207019 | Type I diabetes mellitus with gangrene | T1 |
| 292543014 | C10E611 | 420825003 | 2618207019 | Type I diabetes mellitus with gangrene | T1 |
| 292541011 | C10E600 | 420825003 | 2618207019 | Type 1 diabetes mellitus with gangrene | T1 |
| 616611000006111 | C10z000 | 420868002 | 3013528019 | Disorder due to type 1 diabetes mellitus | T1 |
| 84351000006117 | C108B12 | 420918009 | 2618199015 | Type 1 diabetes mellitus with mononeuropathy | T1 |
| 913781000006117 | C10EB00 | 420918009 | 2618199015 | Type 1 diabetes mellitus with mononeuropathy | T1 |
| 913791000006119 | C10EB11 | 420918009 | 3697664014 | Type I diabetes mellitus with mononeuropathy | T1 |
| 84721000006113 | C108B11 | 420918009 | 3697664014 | Mononeuropathy with type 1 diabetes mellitus | T1 |
| 12332751000006115 |  | 420918009 | 12332751000006115 | Mononeuropathy with type 1 diabetes mellitus | T1 |
| 616651000006112 | C103000 | 421075007 | 2618217012 | Diabetes mellitus, juvenile type, with ketoacidotic coma | T1 |
| 928531000006110 | C10EN11 | 421075007 | 2967758019 | Type I diabetes mellitus with ketoacidotic coma | T1 |
| 928521000006112 | C10EN00 | 421075007 | 2967758019 | Ketoacidotic coma in type 1 diabetes mellitus | T1 |
| 616591000006117 | C107000 | 421365002 | 2618205010 | Diabetes mellitus, juvenile +peripheral circulatory disorder | T1 |
| 616671000006119 | C106000 | 421468001 | 2618197018 | Diabetes mellitus, juvenile, + neurological manifestation | T1 |
| 913511000006117 | C10E200 | 421468001 | 2618197018 | Type 1 diabetes mellitus with neurological complications | T1 |
| 84381000006113 | C108212 | 421468001 | 2618197018 | Type 1 diabetes mellitus with neurological complications | T1 |
| 84751000006116 | C108211 | 421468001 | 3695401013 | Type I diabetes mellitus with neurological complications | T1 |
| 913521000006113 | C10E211 | 421468001 | 3695401013 | Neurological disorder with type 1 diabetes mellitus | T1 |
| 12334611000006115 |  | 421468001 | 12334611000006115 | Neurological disorder with type 1 diabetes mellitus | T1 |
| 913451000006117 | C10E000 | 421893009 | 2618211013 | Type 1 diabetes mellitus with renal complications | T1 |
| 616661000006114 | C104000 | 421893009 | 2618211013 | Diabetes mellitus, juvenile type, with renal manifestation | T1 |
| 84431000006117 | C108012 | 421893009 | 2618211013 | Type 1 diabetes mellitus with renal complications | T1 |
| 913461000006115 | C10E011 | 421893009 | 2623054013 | Type I diabetes mellitus with renal complications | T1 |
| 84371000006110 | C108D12 | 421893009 | 2623054013 | Type 1 diabetes mellitus with nephropathy | T1 |
| 913851000006119 | C10ED11 | 421893009 | 2623054013 | Type I diabetes mellitus with nephropathy | T1 |
| 913841000006116 | C10ED00 | 421893009 | 2623054013 | Renal disorder associated with type 1 diabetes mellitus | T1 |
| 84801000006111 | C108011 | 421893009 | 2623054013 | Type I diabetes mellitus with renal complications | T1 |
| 84741000006118 | C108D11 | 421893009 | 2623054013 | Type I diabetes mellitus with nephropathy | T1 |
| 772141000006118 | C108G00 | 421895002 | 2618203015 | Insulin dependent diab mell with peripheral angiopathy | T1 |
| 787101000006114 | C107300 | 421895002 | 2618203015 | IDDM with peripheral circulatory disorder | T1 |
| 913921000006119 | C10EG12 | 421895002 | 2618203015 | Insulin dependent diab mell with peripheral angiopathy | T1 |
| 84321000006114 | C108F12 | 421920002 | 2618234016 | Type 1 diabetes mellitus with diabetic cataract | T1 |
| 913901000006112 | C10EF00 | 421920002 | 2618234016 | Type 1 diabetes mellitus with diabetic cataract | T1 |
| 84691000006115 | C108F11 | 421920002 | 3688521010 | Cataract due to diabetes mellitus type 1 | T1 |
| 913911000006110 | C10EF11 | 421920002 | 3688521010 | Type I diabetes mellitus with diabetic cataract | T1 |
| 12336111000006119 |  | 421920002 | 12336111000006119 | Cataract due to diabetes mellitus type 1 | T1 |
| 771481000006112 | C108200 | 422088007 | 3688477014 | Insulin-dependent diabetes mellitus with neurological comps | T1 |
| 913501000006115 | C10E212 | 422088007 | 3688477014 | Insulin-dependent diabetes mellitus with neurological comps | T1 |
| 771421000006113 | C108500 | 422183001 | 2623058011 | Insulin dependent diabetes mellitus with ulcer | T1 |
| 913541000006118 | C10E300 | 422228004 | 2618232017 | Type 1 diabetes mellitus with multiple complications | T1 |
| 84361000006115 | C108312 | 422228004 | 2618232017 | Type 1 diabetes mellitus with multiple complications | T1 |
| 913551000006116 | C10E311 | 422228004 | 2967771016 | Type I diabetes mellitus with multiple complications | T1 |
| 84731000006111 | C108311 | 422228004 | 2967771016 | Multiple complications of type 1 diabetes mellitus | T1 |
| 771351000006111 | C108600 | 422275004 | 2616612015 | Insulin dependent diabetes mellitus with gangrene | T1 |
| 913621000006112 | C10E612 | 422275004 | 2616612015 | Insulin dependent diabetes mellitus with gangrene | T1 |
| 292550013 | C10E811 | 444073006 | 2872487013 | Type I diabetes mellitus - poor control | T1 |
| 84291000006117 | C108812 | 444073006 | 2872487013 | Type 1 diabetes mellitus - poor control | T1 |
| 84661000006111 | C108811 | 444073006 | 2872487013 | Type I diabetes mellitus poorly controlled | T1 |
| 292548017 | C10E800 | 444073006 | 2872487013 | Type 1 diabetes mellitus - poor control | T1 |
| 299601000000114 | C10EQ00 | 713702000 | 3698406016 | Gastroparesis with type 1 diabetes mellitus | T1 |
| 1667941000000112 | C10EQ11 | 713702000 | 3698406016 | Type I diabetes mellitus with gastroparesis | T1 |
| 12363661000006113 |  | 713702000 | 12363661000006113 | Gastroparesis with type 1 diabetes mellitus | T1 |
| 84421000006115 | C108C12 | 713705003 | 3297342019 | Type 1 diabetes mellitus with polyneuropathy | T1 |
| 913811000006115 | C10EC00 | 713705003 | 3297342019 | Type 1 diabetes mellitus with polyneuropathy | T1 |
| 913821000006111 | C10EC11 | 713705003 | 3297390014 | Type I diabetes mellitus with polyneuropathy | T1 |
| 84791000006110 | C108C11 | 713705003 | 3297390014 | Polyneuropathy due to diabetes mellitus type I | T1 |
| 616601000006113 | C105000 | 739681000 | 3537386015 | Diabetes mellitus, juvenile type, + ophthalmic manifestation | T1 |
| 913481000006113 | C10E100 | 739681000 | 3537386015 | Type 1 diabetes mellitus with ophthalmic complications | T1 |
| 84401000006113 | C108112 | 739681000 | 3537386015 | Type 1 diabetes mellitus with ophthalmic complications | T1 |
| 913491000006111 | C10E111 | 739681000 | 3698438014 | Type I diabetes mellitus with ophthalmic complications | T1 |
| 84771000006114 | C108111 | 739681000 | 3698438014 | Disorder of eye with type 1 diabetes mellitus | T1 |
| 12370271000006116 |  | 739681000 | 12370271000006116 | Disorder of eye with type 1 diabetes mellitus | T1 |
| 913941000006114 | C10EG11 | 31211000119101 | 3315042015 | Peripheral angiopathy due to type 1 diabetes mellitus | T1 |
| 913931000006116 | C10EG00 | 31211000119101 | 3315042015 | Type 1 diabetes mellitus with peripheral angiopathy | T1 |
| 84781000006112 | C108G11 | 31211000119101 | 3315042015 | Type I diabetes mellitus with peripheral angiopathy | T1 |
| 84411000006111 | C108G12 | 31211000119101 | 3315042015 | Type 1 diabetes mellitus with peripheral angiopathy | T1 |
| 84761000006119 | C108J11 | 71771000119100 | 3010513018 | Type I diabetes mellitus with neuropathic arthropathy | T1 |
| 459296014 | C10EJ00 | 71771000119100 | 3010513018 | Type 1 diabetes mellitus with neuropathic arthropathy | T1 |
| 84391000006111 | C108J12 | 71771000119100 | 3010513018 | Type 1 diabetes mellitus with neuropathic arthropathy | T1 |
| 459295013 | C10EJ11 | 71771000119100 | 3010513018 | Type I diabetes mellitus with neuropathic arthropathy | T1 |
| 771381000006115 | C108300 | 385041000000108 | 760111000000115 | Insulin dependent diabetes mellitus with multiple complicatn | T1 |
| 913531000006111 | C10E312 | 385041000000108 | 760111000000115 | Insulin dependent diabetes mellitus with multiple complicat | T1 |
| 84471000006119 | C109.12 | 44054006 | 197761014 | Type 2 diabetes mellitus | T2 |
| 197761014 | C10F.00 | 44054006 | 197761014 | Type 2 diabetes mellitus | T2 |
| 280571000006116 | C109.00 | 44054006 | 493773010 | Non-insulin dependent diabetes mellitus | T2 |
| 73466011 | C100112 | 44054006 | 493773010 | Non-insulin dependent diabetes mellitus | T2 |
| 493773010 | C109.11 | 44054006 | 493773010 | NIDDM - Non-insulin dependent diabetes mellitus | T2 |
| 84841000006113 | C109.13 | 44054006 | 493774016 | Type II diabetes mellitus | T2 |
| 223291000000111 | C100111 | 44054006 | 493774016 | Maturity onset diabetes | T2 |
| 493774016 | C10F.11 | 44054006 | 493774016 | Type II diabetes mellitus | T2 |
| 616571000006118 | C10y100 | 73211009 | 121589010 | Diabetes mellitus, adult, + other specified manifestation | T2 |
| 292483016 | C102100 | 190331003 | 292483016 | Diabetes mellitus, adult onset, with hyperosmolar coma | T2 |
| 292576013 | C10F311 | 190388001 | 292576013 | Type II diabetes mellitus with multiple complications | T2 |
| 84911000006119 | C109311 | 190388001 | 292576013 | Type II diabetes mellitus with multiple complications | T2 |
| 292577016 | C10F300 | 190388001 | 292577016 | Type 2 diabetes mellitus with multiple complications | T2 |
| 84541000006111 | C109312 | 190388001 | 292577016 | Type 2 diabetes mellitus with multiple complications | T2 |
| 280581000006118 | C109300 | 190388001 | 292578014 | Non-insulin-dependent diabetes mellitus with multiple complications | T2 |
| 292579018 | C10F400 | 190389009 | 292579018 | Type 2 diabetes mellitus with ulcer | T2 |
| 84631000006119 | C109412 | 190389009 | 292579018 | Type 2 diabetes mellitus with ulcer | T2 |
| 280551000006114 | C109400 | 190389009 | 292580015 | Non-insulin-dependent diabetes mellitus with ulcer | T2 |
| 85001000006117 | C109411 | 190389009 | 292581016 | Type II diabetes mellitus with ulcer | T2 |
| 292581016 | C10F411 | 190389009 | 292581016 | Type II diabetes mellitus with ulcer | T2 |
| 12450771000006115 |  | 190390000 | 292582011 | Type II diabetes mellitus with gangrene | T2 |
| 12450751000006113 |  | 190390000 | 292583018 | Type 2 diabetes mellitus with gangrene | T2 |
| 841351000006110 | C109J12 | 237599002 | 1223147012 | Insulin treated Type II diabetes mellitus | T2 |
| 1223147012 | C10FJ11 | 237599002 | 1223147012 | Insulin treated Type II diabetes mellitus | T2 |
| 1223148019 | C109J11 | 237599002 | 1223148019 | Insulin treated non-insulin dependent diabetes mellitus | T2 |
| 914391000006116 | C10FJ00 | 237599002 | 2967820013 | Insulin treated Type 2 diabetes mellitus | T2 |
| 840951000006119 | C109J00 | 237599002 | 2967820013 | Insulin treated Type 2 diabetes mellitus | T2 |
| 457328010 | C109900 | 313436004 | 457328010 | Non-insulin-dependent diabetes mellitus without complication | T2 |
| 616511000006110 | C100100 | 313436004 | 457329019 | Diabetes mellitus, adult onset, no mention of complication | T2 |
| 84641000006112 | C109912 | 313436004 | 457329019 | Type 2 diabetes mellitus without complication | T2 |
| 457329019 | C10F900 | 313436004 | 457329019 | Type 2 diabetes mellitus without complication | T2 |
| 85011000006119 | C109911 | 313436004 | 457330012 | Type II diabetes mellitus without complication | T2 |
| 457330012 | C10F911 | 313436004 | 457330012 | Type II diabetes mellitus without complication | T2 |
| 84961000006116 | C109F11 | 314902007 | 459306016 | Type II diabetes mellitus with peripheral angiopathy | T2 |
| 459306016 | C10FF11 | 314902007 | 459306016 | Type II diabetes mellitus with peripheral angiopathy | T2 |
| 280561000006111 | C109F00 | 314902007 | 459307013 | Non-insulin-dependent diabetes mellitus with peripheral angiopathy | T2 |
| 459308015 | C10FF00 | 314902007 | 459308015 | Type 2 diabetes mellitus with peripheral angiopathy | T2 |
| 84591000006119 | C109F12 | 314902007 | 459308015 | Type 2 diabetes mellitus with peripheral angiopathy | T2 |
| 459309011 | C109G00 | 314903002 | 459309011 | Non-insulin dependent diabetes mellitus with arthropathy | T2 |
| 84491000006118 | C109G12 | 314903002 | 459310018 | Type 2 diabetes mellitus with arthropathy | T2 |
| 459310018 | C10FG00 | 314903002 | 459310018 | Type 2 diabetes mellitus with arthropathy | T2 |
| 459311019 | C10FG11 | 314903002 | 459311019 | Type II diabetes mellitus with arthropathy | T2 |
| 84861000006112 | C109G11 | 314903002 | 459311019 | Type II diabetes mellitus with arthropathy | T2 |
| 84941000006115 | C109H11 | 314904008 | 459312014 | Type II diabetes mellitus with neuropathic arthropathy | T2 |
| 459312014 | C10FH11 | 314904008 | 459312014 | Type II diabetes mellitus with neuropathic arthropathy | T2 |
| 459313016 | C10FH00 | 314904008 | 459313016 | Type 2 diabetes mellitus with neuropathic arthropathy | T2 |
| 84571000006115 | C109H12 | 314904008 | 459313016 | Type 2 diabetes mellitus with neuropathic arthropathy | T2 |
| 281181000006116 | C109H00 | 314904008 | 459314010 | Non-insulin dependent diabetes mellitus with neuropathic arthropathy | T2 |
| 1488898011 | C10FK00 | 395204000 | 1488898011 | Hyperosmolar non-ketotic state in type 2 diabetes mellitus | T2 |
| 850691000006118 | C109K00 | 395204000 | 1488898011 | Hyperosmolar non-ketotic state in type 2 diabetes mellitus | T2 |
| 1667891000000113 | C10FK11 | 395204000 | 1667891000000113 | Hyperosmolar non-ketotic state in type II diabetes mellitus | T2 |
| 587521000006111 | C109000 | 420279001 | 2615535015 | Non-insulin-dependent diabetes mellitus with renal comps | T2 |
| 616551000006111 | C104100 | 420279001 | 2615535015 | Diabetes mellitus, adult onset, with renal manifestation | T2 |
| 914031000006118 | C10F000 | 420279001 | 2618208012 | Renal disorder associated with type II diabetes mellitus | T2 |
| 84611000006113 | C109012 | 420279001 | 2618208012 | Type 2 diabetes mellitus with renal complications | T2 |
| 84921000006110 | C109C11 | 420279001 | 3013392012 | Renal disorder due to type 2 diabetes mellitus | T2 |
| 914041000006111 | C10F011 | 420279001 | 3013392012 | Type II diabetes mellitus with renal complications | T2 |
| 84981000006114 | C109011 | 420279001 | 3013392012 | Type II diabetes mellitus with renal complications | T2 |
| 914271000006114 | C10FC11 | 420279001 | 3013392012 | Type II diabetes mellitus with nephropathy | T2 |
| 914261000006119 | C10FC00 | 420279001 | 3035432019 | Diabetes type 2 with nephropathy | T2 |
| 84551000006113 | C109C12 | 420279001 | 3035432019 | Type 2 diabetes mellitus with nephropathy | T2 |
| 280531000006119 | C109C00 | 420279001 | 3035432019 | Non-insulin dependent diabetes mellitus with nephropathy | T2 |
| 84531000006118 | C109A12 | 420436000 | 2618201018 | Type 2 diabetes mellitus with mononeuropathy | T2 |
| 914221000006113 | C10FA00 | 420436000 | 2618201018 | Type 2 diabetes mellitus with mononeuropathy | T2 |
| 914231000006111 | C10FA11 | 420436000 | 3697667019 | Type II diabetes mellitus with mononeuropathy | T2 |
| 84901000006117 | C109A11 | 420436000 | 3697667019 | Mononeuropathy with type 2 diabetes mellitus | T2 |
| 280521000006117 | C109A00 | 420436000 | 3697667019 | Non-insulin dependent diabetes mellitus with mononeuropathy | T2 |
| 12331051000006112 |  | 420436000 | 12331051000006112 | Mononeuropathy with type 2 diabetes mellitus | T2 |
| 928571000006113 | C10FM11 | 420715001 | 2967769016 | Type II diabetes mellitus with persistent microalbuminuria | T2 |
| 928561000006118 | C10FM00 | 420715001 | 2967769016 | Persistent microalbuminuria associated with type 2 diabetes mellitus | T2 |
| 281161000006114 | C109E00 | 420756003 | 3035281015 | Non-insulin depend diabetes mellitus with diabetic cataract | T2 |
| 914301000006111 | C10FE00 | 420756003 | 3688522015 | Type 2 diabetes mellitus with diabetic cataract | T2 |
| 914311000006114 | C10FE11 | 420756003 | 3688522015 | Type II diabetes mellitus with diabetic cataract | T2 |
| 84501000006114 | C109E12 | 420756003 | 3688522015 | Type 2 diabetes mellitus with diabetic cataract | T2 |
| 84871000006117 | C109E11 | 420756003 | 3688522015 | Cataract due to diabetes mellitus type 2 | T2 |
| 12332101000006118 |  | 420756003 | 12332101000006118 | Cataract due to diabetes mellitus type 2 | T2 |
| 616481000006119 | C106100 | 421326000 | 2618196010 | Diabetes mellitus, adult onset, + neurological manifestation | T2 |
| 12704971000006113 |  | 421326000 | 2623027010 | Neurologic disorder associated with type 2 diabetes mellitus | T2 |
| 84561000006110 | C109212 | 421326000 | 3695406015 | Type 2 diabetes mellitus with neurological complications | T2 |
| 84931000006113 | C109211 | 421326000 | 3695406015 | Neurological disorder with diabetes type 2 | T2 |
| 914081000006117 | C10F211 | 421326000 | 3695406015 | Type II diabetes mellitus with neurological complications | T2 |
| 914071000006115 | C10F200 | 421326000 | 3695406015 | Neurologic disorder associated with type 2 diabetes mellitus | T2 |
| 280591000006115 | C109200 | 421326000 | 3695406015 | Non-insulin-dependent diabetes mellitus with neuro comps | T2 |
| 12702411000006115 |  | 421326000 | 84931000006113 | Type II diabetes mellitus with neurological complications | T2 |
| 12334141000006111 |  | 421326000 | 12334141000006111 | Neurological disorder with diabetes type 2 | T2 |
| 292582011 | C10F511 | 421631007 | 2618206011 | Type II diabetes mellitus with gangrene | T2 |
| 84511000006112 | C109512 | 421631007 | 2618206011 | Type 2 diabetes mellitus with gangrene | T2 |
| 616561000006113 | C107200 | 421631007 | 2618206011 | Diabetes mellitus, adult with gangrene | T2 |
| 84881000006119 | C109511 | 421631007 | 2618206011 | Type II diabetes mellitus with gangrene | T2 |
| 292583018 | C10F500 | 421631007 | 2618206011 | Type 2 diabetes mellitus with gangrene | T2 |
| 281211000006117 | C109500 | 421631007 | 2967779019 | Gangrene associated with type 2 diabetes mellitus | T2 |
| 6975211000006112 |  | 421631007 | 6975211000006112 | Gangrene associated with type 2 diabetes mellitus | T2 |
| 616531000006116 | C101100 | 421750000 | 2618220016 | Diabetes mellitus, adult onset, with ketoacidosis | T2 |
| 928581000006111 | C10FN00 | 421750000 | 2967754017 | Ketoacidosis in type 2 diabetes mellitus | T2 |
| 928591000006114 | C10FN11 | 421750000 | 2967754017 | Type II diabetes mellitus with ketoacidosis | T2 |
| 938321000006116 | C10FQ00 | 421779007 | 2618237011 | Exudative maculopathy associated with type 2 diabetes mellitus | T2 |
| 12705011000006116 |  | 421779007 | 2967775013 | Exudative maculopathy associated with type 2 diabetes mellitus | T2 |
| 938331000006118 | C10FQ11 | 421779007 | 3698529010 | Exudative maculopathy with type 2 diabetes mellitus | T2 |
| 12335671000006113 |  | 421779007 | 12335671000006113 | Exudative maculopathy with type 2 diabetes mellitus | T2 |
| 928601000006118 | C10FP00 | 421847006 | 2618218019 | Ketoacidotic coma in type 2 diabetes mellitus | T2 |
| 616541000006114 | C103100 | 421847006 | 2618218019 | Diabetes mellitus, adult onset, with ketoacidotic coma | T2 |
| 928611000006115 | C10FP11 | 421847006 | 2618218019 | Ketoacidotic coma in type II diabetes mellitus | T2 |
| 12704991000006114 |  | 421847006 | 2967777017 | Ketoacidotic coma in type 2 diabetes mellitus | T2 |
| 928541000006117 | C10FL00 | 421986006 | 2967751013 | Persistent proteinuria associated with type 2 diabetes mellitus | T2 |
| 928551000006115 | C10FL11 | 421986006 | 2967751013 | Type II diabetes mellitus with persistent proteinuria | T2 |
| 616501000006112 | C10z100 | 422014003 | 3013072014 | Disorder due to type 2 diabetes mellitus | T2 |
| 641581000006115 | C109600 | 422034002 | 3035295017 | Non-insulin-dependent diabetes mellitus with retinopathy | T2 |
| 914161000006114 | C10F611 | 422034002 | 3699410014 | Type II diabetes mellitus with retinopathy | T2 |
| 84991000006112 | C109611 | 422034002 | 3699410014 | Type II diabetes mellitus with retinopathy | T2 |
| 914151000006112 | C10F600 | 422034002 | 3699410014 | Retinopathy with type 2 diabetes mellitus | T2 |
| 84621000006117 | C109612 | 422034002 | 3699410014 | Type 2 diabetes mellitus with retinopathy | T2 |
| 12336531000006116 |  | 422034002 | 12336531000006116 | Retinopathy with type 2 diabetes mellitus | T2 |
| 84951000006118 | C109111 | 422099009 | 3698440016 | Disorder of eye with type 2 diabetes mellitus | T2 |
| 587111000006111 | C109100 | 422099009 | 3698440016 | Non-insulin-dependent diabetes mellitus with ophthalm comps | T2 |
| 84581000006117 | C109112 | 422099009 | 3698440016 | Type 2 diabetes mellitus with ophthalmic complications | T2 |
| 914051000006113 | C10F100 | 422099009 | 3698440016 | Disorder of eye with type 2 diabetes mellitus | T2 |
| 914061000006110 | C10F111 | 422099009 | 3698440016 | Type II diabetes mellitus with ophthalmic complications | T2 |
| 12336871000006115 |  | 422099009 | 12336871000006115 | Disorder of eye with type 2 diabetes mellitus | T2 |
| 12702421000006111 |  | 422099009 | 12702421000006111 | Type II diabetes mellitus with ophthalmic complications | T2 |
| 616581000006115 | C107100 | 422166005 | 2618204014 | Diabetes mellitus, adult, + peripheral circulatory disorder | T2 |
| 674961000006118 | C107400 | 422166005 | 2623039019 | Peripheral circulatory disorder associated with type 2 diabetes mellitus | T2 |
| 6985001000006110 |  | 422166005 | 6985001000006110 | Peripheral circulatory disorder associated with type 2 diabetes mellitus | T2 |
| 281171000006119 | C109700 | 443694000 | 2842387018 | Type II diabetes mellitus uncontrolled | T2 |
| 292590011 | C10F700 | 443694000 | 2921019012 | Type II diabetes mellitus poorly controlled | T2 |
| 84851000006110 | C109711 | 443694000 | 2921019012 | Type II diabetes mellitus - poor control | T2 |
| 292589019 | C10F711 | 443694000 | 2921019012 | Type II diabetes mellitus - poor control | T2 |
| 84481000006116 | C109712 | 443694000 | 2921019012 | Type 2 diabetes mellitus - poor control | T2 |
| 1667921000000117 | C10FR11 | 713703005 | 3698412014 | Type II diabetes mellitus with gastroparesis | T2 |
| 299621000000117 | C10FR00 | 713703005 | 3698412014 | Gastroparesis with type 2 diabetes mellitus | T2 |
| 12363681000006115 |  | 713703005 | 12363681000006115 | Gastroparesis with type 2 diabetes mellitus | T2 |
| 84971000006111 | C109B11 | 713706002 | 3297353013 | Type II diabetes mellitus with polyneuropathy | T2 |
| 84601000006110 | C109B12 | 713706002 | 3297353013 | Type 2 diabetes mellitus with polyneuropathy | T2 |
| 280541000006112 | C109B00 | 713706002 | 3297353013 | Non-insulin dependent diabetes mellitus with polyneuropathy | T2 |
| 914251000006116 | C10FB11 | 713706002 | 3297353013 | Type II diabetes mellitus with polyneuropathy | T2 |
| 914241000006118 | C10FB00 | 713706002 | 3297353013 | Polyneuropathy due to type 2 diabetes mellitus | T2 |
| 459169018 | C10FD11 | 719216001 | 3316336015 | Hypoglycaemic coma co-occurrent and due to diabetes mellitus type II | T2 |
| 280511000006113 | C109D00 | 719216001 | 3316336015 | Non-insulin dependent diabetes mellitus with hypoglyca coma | T2 |
| 84521000006116 | C109D12 | 719216001 | 3316336015 | Type 2 diabetes mellitus with hypoglycaemic coma | T2 |
| 84891000006116 | C109D11 | 719216001 | 3316336015 | Type II diabetes mellitus with hypoglycaemic coma | T2 |
| 459167016 | C10FD00 | 719216001 | 3316336015 | Type 2 diabetes mellitus with hypoglycaemic coma | T2 |
| 7783451000006114 |  | 719216001 | 7783451000006114 | Hypoglycaemic coma co-occurrent and due to diabetes mellitus type II | T2 |
| 7966941000006116 |  | 791000119109 | 2967590010 | Angina associated with type II diabetes mellitus | T2 |
| 7966951000006119 |  | 791000119109 | 2967662015 | Diabetic angina pectoris associated with type 2 diabetes mellitus | T2 |
| 7966931000006114 |  | 791000119109 | 2967773018 | Angina associated with type 2 diabetes mellitus | T2 |
| 881451000006113 | C100199 | 532411000000102 | 881451000006113 | Diabetes mellitus -adult onset | T2 |
| 9093013 | F420.00 | 4855003 | 9093013 | Diabetic retinopathy | Unspecified |
| 297758012 | F420z00 | 4855003 | 9093013 | Diabetic retinopathy NOS | Unspecified |
| 169731000006118 | 2BBF.00 | 4855003 | 1230609013 | Retinal abnormality - diabetes-related | Unspecified |
| 292503016 | C105z00 | 25093002 | 42062018 | Diabetes mellitus NOS with ophthalmic manifestation | Unspecified |
| 13771000006110 | C105y00 | 25093002 | 42062018 | Other specified diabetes mellitus with ophthalmic complicatn | Unspecified |
| 616421000006118 | C105.00 | 25093002 | 3688467012 | Eye disorder due to diabetes mellitus | Unspecified |
| 881491000006119 | C105.99 | 25093002 | 881491000006119 | Diabetes + eye manifestation | Unspecified |
| 12174801000006114 |  | 25093002 | 12174801000006114 | Eye disorder due to diabetes mellitus | Unspecified |
| 2906401000006113 |  | 25412000 | 42612012 | Diabetic retinal microaneurysm | Unspecified |
| 616381000006111 | C103.00 | 26298008 | 44045012 | Diabetes mellitus with ketoacidotic coma | Unspecified |
| 881471000006115 | C103.99 | 26298008 | 881471000006115 | Diabetes with coma | Unspecified |
| 65526011 | F381311 | 39058009 | 65526011 | Diabetic amyotrophy | Unspecified |
| 616921000006113 | C106.11 | 39058009 | 65526011 | Diabetic amyotrophy | Unspecified |
| 73294018 | F464000 | 43959009 | 73294018 | Diabetic cataract | Unspecified |
| 214921000006116 | F372.00 | 49455004 | 82373015 | Polyneuropathy in diabetes | Unspecified |
| 616451000006110 | C106.13 | 49455004 | 82373015 | Diabetes mellitus with polyneuropathy | Unspecified |
| 82373015 | F372.11 | 49455004 | 82373015 | Diabetic polyneuropathy | Unspecified |
| 1230890017 | F171100 | 50620007 | 1230890017 | Autonomic neuropathy due to diabetes | Unspecified |
| 98476015 | F420100 | 59276001 | 98476015 | Proliferative diabetic retinopathy | Unspecified |
| 377001000006117 | Cyu2.00 | 73211009 | 121589010 | [X]Diabetes mellitus | Unspecified |
| 121589010 | C10..00 | 73211009 | 121589010 | Diabetes mellitus | Unspecified |
| 293756010 | Cyu2000 | 73211009 | 121589010 | [X]Other specified diabetes mellitus | Unspecified |
| 292621010 | C10yz00 | 73211009 | 121589010 | Diabetes mellitus NOS with other specified manifestation | Unspecified |
| 13781000006113 | C10yy00 | 73211009 | 121589010 | Other specified diabetes mellitus with other spec comps | Unspecified |
| 292622015 | C10z.00 | 74627003 | 123939015 | Diabetes mellitus with unspecified complication | Unspecified |
| 292617015 | C10y.00 | 74627003 | 123939015 | Diabetes mellitus with other specified manifestation | Unspecified |
| 292626017 | C10zz00 | 74627003 | 123939015 | Diabetic complication | Unspecified |
| 13811000006110 | C10zy00 | 74627003 | 123939015 | Other specified diabetes mellitus with unspecified comps | Unspecified |
| 292475010 | C100z00 | 111552007 | 178796018 | Diabetes mellitus NOS with no mention of complication | Unspecified |
| 292466013 | C100.00 | 111552007 | 178796018 | Diabetes mellitus without complication | Unspecified |
| 178798017 |  | 111556005 | 178798017 | Diabetic ketoacidosis without coma | Unspecified |
| 292495016 | C104y00 | 127013003 | 301016 | Other specified diabetes mellitus with renal complications | Unspecified |
| 293759015 | Cyu2300 | 127013003 | 301016 | Diabetic renal disease | Unspecified |
| 292496015 | C104z00 | 127013003 | 301016 | Diabetes mellitus with nephropathy NOS | Unspecified |
| 616461000006112 | C104.00 | 127013003 | 301016 | Diabetes mellitus with renal manifestation | Unspecified |
| 205225016 | C104.11 | 127013003 | 205225016 | Diabetic nephropathy | Unspecified |
| 881481000006117 | C104.99 | 127013003 | 881481000006117 | Diabetes + nephropathy | Unspecified |
| 302011 | G73y000 | 127014009 | 302011 | Diabetic peripheral angiopathy | Unspecified |
| 251591016 | 1434.00 | 161445009 | 251591016 | H/O: diabetes mellitus | Unspecified |
| 264707013 | 66AI.00 | 170763003 | 264707013 | Diabetic - good control | Unspecified |
| 297492014 | F345000 | 193141005 | 297492014 | Diabetic mononeuritis multiplex | Unspecified |
| 297510017 | F35z000 | 193141005 | 297492014 | Diabetic mononeuritis NOS | Unspecified |
| 297550019 | F372000 | 193183000 | 297550019 | Acute painful diabetic neuropathy | Unspecified |
| 297551015 | F372100 | 193184006 | 297551015 | Chronic painful diabetic neuropathy | Unspecified |
| 297552010 | F372200 | 193185007 | 297552010 | Asymptomatic diabetic neuropathy | Unspecified |
| 297754014 | F420200 | 193349004 | 297754014 | Preproliferative diabetic retinopathy | Unspecified |
| 297755010 | F420300 | 193350004 | 297755010 | Advanced diabetic maculopathy | Unspecified |
| 317347010 | R054200 | 195302000 | 300505017 | [D]Gangrene of toe in diabetic | Unspecified |
| 317348017 | R054300 | 195303005 | 300506016 | [D]Widespread diabetic foot gangrene | Unspecified |
| 303846016 | K01x100 | 197605007 | 303846016 | Nephrotic syndrome in diabetes mellitus | Unspecified |
| 303847013 | K01x111 | 197605007 | 303847013 | Kimmelstiel - Wilson disease | Unspecified |
| 616151000006118 | L180000 | 199223000 | 306106018 | Diabetes mellitus - unspec whether in pregnancy/puerperium | Unspecified |
| 309177010 | M271000 | 201250006 | 309177010 | Ischaemic ulcer diabetic foot | Unspecified |
| 309179013 | M271100 | 201251005 | 309179013 | Neuropathic diabetic ulcer - foot | Unspecified |
| 309180011 | M271200 | 201252003 | 309180011 | Mixed diabetic ulcer - foot | Unspecified |
| 309737011 | N030000 | 201723002 | 309737011 | Diabetic cheiroarthropathy | Unspecified |
| 309738018 | N030011 | 201723002 | 309738018 | Diabetic cheiropathy | Unspecified |
| 616971000006114 | N030100 | 201724008 | 309739014 | Diabetic Charcot's arthropathy | Unspecified |
| 345486016 | F372.12 | 230572002 | 345486016 | Diabetic neuropathy | Unspecified |
| 345487013 | C106.12 | 230572002 | 345487013 | Diabetes mellitus with neuropathy | Unspecified |
| 881501000006110 | C106.99 | 230572002 | 881501000006110 | Diabetes + neuropathy | Unspecified |
| 345492010 | F3y0.00 | 230577008 | 345492010 | Diabetic mononeuropathy | Unspecified |
| 347657010 | F420400 | 232020009 | 347657010 | Diabetic maculopathy | Unspecified |
| 354508018 | K08yA11 | 236500003 | 354508018 | Clinical diabetic nephropathy | Unspecified |
| 354509014 | K08yA00 | 236500003 | 354509014 | Proteinuric diabetic nephropathy | Unspecified |
| 264716012 | 66AJz00 | 268519009 | 401531012 | Diabetic - poor control NOS | Unspecified |
| 401531012 | 66AJ.00 | 268519009 | 401531012 | Diabetic - poor control | Unspecified |
| 764201000006110 | 2G5C.00 | 280137006 | 417658010 | Foot abnormality - diabetes related | Unspecified |
| 764191000006112 | 2G51000 | 280137006 | 417658010 | Foot abnormality - diabetes related | Unspecified |
| 451410016 | 2G5A.00 | 308105005 | 451410016 | O/E - Right diabetic foot at risk | Unspecified |
| 451411017 | 2G5B.00 | 308106006 | 451411017 | O/E - Left diabetic foot at risk | Unspecified |
| 305158015 | Kyu0300 | 309426007 | 452844015 | Diabetic glomerulopathy | Unspecified |
| 454092018 |  | 310505005 | 454092018 | Diabetic hyperosmolar non-ketotic state | Unspecified |
| 454093011 |  | 310505005 | 454093011 | HONKS - Diabetic hyperosmolar non-ketotic state | Unspecified |
| 3330159019 |  | 310505005 | 3330159019 | HHS - Hyperosmolar hyperglycemic syndrome | Unspecified |
| 3330160012 |  | 310505005 | 3330160012 | HHS - Hyperosmolar hyperglycaemic syndrome | Unspecified |
| 455408014 | F420500 | 311782002 | 455408014 | Advanced diabetic retinal disease | Unspecified |
| 841011000006112 | F420800 | 312905005 | 1775725011 | Severe nonproliferative diabetic retinopathy | Unspecified |
| 1785163015 | F420700 | 312907002 | 1785163015 | High risk proliferative diabetic retinopathy | Unspecified |
| 1484867016 | F420600 | 390834004 | 1484867016 | Non proliferative diabetic retinopathy | Unspecified |
| 1785332013 | F420000 | 390834004 | 1785332013 | Background diabetic retinopathy | Unspecified |
| 1484887015 | 2BBL.00 | 390854003 | 1484887015 | O/E - diabetic maculopathy present both eyes | Unspecified |
| 1488393013 | 2G5E.00 | 394671009 | 1488393013 | O/E - Right diabetic foot at low risk | Unspecified |
| 1488394019 | 2G5G.00 | 394672002 | 1488394019 | O/E - Right diabetic foot at high risk | Unspecified |
| 1488395018 | 2G5H.00 | 394673007 | 1488395018 | O/E - Right diabetic foot - ulcerated | Unspecified |
| 1488396017 | 2G5L.00 | 394674001 | 1488396017 | O/E - Left diabetic foot - ulcerated | Unspecified |
| 1488397014 | 2G5I.00 | 394675000 | 1488397014 | O/E - Left diabetic foot at low risk | Unspecified |
| 1488398016 | 2G5K.00 | 394676004 | 1488398016 | O/E - Left diabetic foot at high risk | Unspecified |
| 1488403015 | 2G5J.00 | 394681008 | 1488403015 | O/E - Left diabetic foot at moderate risk | Unspecified |
| 1488404014 | 2G5F.00 | 394682001 | 1488404014 | O/E - Right diabetic foot at moderate risk | Unspecified |
| 1694761000006113 |  | 405749004 | 2157525015 | Newly diagnosed diabetes | Unspecified |
| 2159973010 | 2BBP.00 | 408409007 | 2159973010 | O/E - right eye background diabetic retinopathy | Unspecified |
| 2159974016 | 2BBQ.00 | 408410002 | 2159974016 | O/E - left eye background diabetic retinopathy | Unspecified |
| 2159975015 | 2BBR.00 | 408411003 | 2159975015 | O/E - right eye preproliferative diabetic retinopathy | Unspecified |
| 2159976019 | 2BBS.00 | 408412005 | 2159976019 | O/E - left eye preproliferative diabetic retinopathy | Unspecified |
| 2159977011 | 2BBT.00 | 408413000 | 2159977011 | O/E - right eye proliferative diabetic retinopathy | Unspecified |
| 2159978018 | 2BBV.00 | 408414006 | 2159978018 | O/E - left eye proliferative diabetic retinopathy | Unspecified |
| 2532976019 | 2G5W.00 | 414890007 | 2532976019 | O/E - left chronic diabetic foot ulcer | Unspecified |
| 975261000006113 | 2BBl.00 | 414894003 | 2532964019 | O/E - left eye stable treated proliferative diabetic retinopathy | Unspecified |
| 2532977011 | 2G5V.00 | 414906009 | 2532977011 | O/E - right chronic diabetic foot ulcer | Unspecified |
| 975251000006111 | 2BBk.00 | 414910007 | 2532963013 | O/E - right eye stable treated proliferative diabetic retinopathy | Unspecified |
| 2549896013 | 2BBo.00 | 417677008 | 2549896013 | O/E - sight threatening diabetic retinopathy | Unspecified |
| 292489017 | C103z00 | 420422005 | 2616611010 | Diabetes mellitus NOS with ketoacidotic coma | Unspecified |
| 292478012 | C101y00 | 420422005 | 2616611010 | Other specified diabetes mellitus with ketoacidosis | Unspecified |
| 292479016 | C101z00 | 420422005 | 2616611010 | Diabetes mellitus NOS with ketoacidosis | Unspecified |
| 2622193012 | C101.00 | 420422005 | 2622193012 | Diabetes mellitus with ketoacidosis | Unspecified |
| 881461000006110 | C101.99 | 420422005 | 881461000006110 | Diabetes+ketoacidosis -no coma | Unspecified |
| 292488013 | C103y00 | 420662003 | 2618230013 | Coma associated with diabetes mellitus | Unspecified |
| 13791000006111 | C107y00 | 421895002 | 2618203015 | Other specified diabetes mellitus with periph circ comps | Unspecified |
| 292523015 | C107z00 | 421895002 | 2618203015 | Diabetes mellitus NOS with peripheral circulatory disorder | Unspecified |
| 616441000006113 | C107.00 | 421895002 | 3688501014 | Peripheral vascular disorder due to diabetes mellitus | Unspecified |
| 12335991000006117 |  | 421895002 | 12335991000006117 | Peripheral vascular disorder due to diabetes mellitus | Unspecified |
| 6981271000006112 |  | 421966007 | 2618222012 | Non-ketotic non-hyperosmolar coma associated with diabetes mellitus | Unspecified |
| 292512019 | C106z00 | 422088007 | 2618195014 | Diabetes mellitus NOS with neurological manifestation | Unspecified |
| 13761000006115 | C106y00 | 422088007 | 2618195014 | Other specified diabetes mellitus with neurological comps | Unspecified |
| 616391000006114 | C106.00 | 422088007 | 3688477014 | Nervous system disorder due to diabetes mellitus | Unspecified |
| 12336851000006113 |  | 422088007 | 12336851000006113 | Nervous system disorder due to diabetes mellitus | Unspecified |
| 616491000006116 | C105100 | 422099009 | 2618233010 | Diabetes mellitus, adult onset, + ophthalmic manifestation | Unspecified |
| 292480018 | C102.00 | 422126006 | 2618221017 | Diabetes mellitus with hyperosmolar coma | Unspecified |
| 292484010 | C102z00 | 422126006 | 2618221017 | Hyperosmolar coma associated with diabetes mellitus | Unspecified |
| 913591000006110 | C10E512 | 422183001 | 2623058011 | Skin ulcer associated with diabetes mellitus | Unspecified |
| 6985421000006113 |  | 422183001 | 6985421000006113 | Skin ulcer associated with diabetes mellitus | Unspecified |
| 616831000006118 | C107.12 | 422275004 | 2616612015 | Diabetes with gangrene | Unspecified |
| 616351000006115 | C107.11 | 422275004 | 2616612015 | Diabetes mellitus with gangrene | Unspecified |
| 881511000006113 | C107.99 | 422275004 | 881511000006113 | Diabetes + periph.circulat.dis | Unspecified |
| 2159979014 | 2BBW.00 | 769244003 | 3698428016 | Maculopathy of right eye with diabetes mellitus | Unspecified |
| 2159980012 | 2BBX.00 | 769245002 | 3698426017 | Maculopathy of left eye with diabetes mellitus | Unspecified |
| 733161000000116 | 2BBr.00 | 373041000000101 | 733161000000116 | Impaired vision due to diabetic retinopathy | Unspecified |
| 13751000006117 | C108y00 | 385041000000108 | 760111000000115 | Other specified diabetes mellitus with multiple comps | Unspecified |
| 292565014 | C108z00 | 385041000000108 | 760111000000115 | Diabetes mellitus with multiple complications | Unspecified |
| 616891000006119 |  | 616891000006103 | 616891000006119 | Diabetic - moderate control | Unspecified |
| 932641000006116 | C103y99 | 630521000000101 | 932641000006116 | Diabetes with coma | Unspecified |
| 881521000006117 | C10y.99 | 658011000000104 | 881521000006117 | Diabetes + other complications | Unspecified |
| 12730971000006110 |  | 658011000000104 | 1445721000000115 | Diabetes mellitus with other specified manifestation | Unspecified |
| 933211000006112 | C10yz99 | 658061000000102 | 933211000006112 | DM + persist microalbuminuria | Unspecified |
| 933221000006116 | C10yz98 | 658061000000102 | 933221000006116 | DM + persistent proteinuria | Unspecified |
| 12730981000006113 |  | 658061000000102 | 1445831000000112 | Diabetes mellitus NOS with other specified manifestation | Unspecified |
| 1666911000000113 | 2G5d.00 | 756381000000100 | 1666911000000113 | O/E - Left diabetic foot at increased risk | Unspecified |
| 1666931000000117 | 2G5e.00 | 756391000000103 | 1666931000000117 | O/E - Right diabetic foot at increased risk | Unspecified |
| 856611000006114 |  | 856611000006105 | 856611000006114 | Laser treated diabetic retinopathy | Unspecified |
| 856621000006118 |  | 856621000006102 | 856621000006118 | Left proliferative diabetic retinopathy | Unspecified |
| 856631000006115 |  | 856631000006104 | 856631000006115 | Left laser treated diabetic retinopathy | Unspecified |
| 856641000006113 |  | 856641000006109 | 856641000006113 | Left diabetic maculopathy | Unspecified |
| 856651000006110 |  | 856651000006106 | 856651000006110 | Left advanced diabetic retinal disease | Unspecified |
| 856661000006112 |  | 856661000006108 | 856661000006112 | Left diabetic foot active ulceration | Unspecified |
| 857031000006113 |  | 857031000006109 | 857031000006113 | Left non-proliferative diabetic retinopathy | Unspecified |
| 857051000006118 |  | 857051000006102 | 857051000006118 | Left preproliferative diabetic retinopathy | Unspecified |
| 857411000006110 |  | 857411000006106 | 857411000006110 | Right proliferative diabetic retinopathy | Unspecified |
| 857421000006119 |  | 857421000006103 | 857421000006119 | Right laser treated diabetic retinopathy | Unspecified |
| 857431000006116 |  | 857431000006100 | 857431000006116 | Right diabetic maculopathy | Unspecified |
| 857441000006114 |  | 857441000006105 | 857441000006114 | Right advanced diabetic retinal disease | Unspecified |
| 857691000006119 |  | 857691000006103 | 857691000006119 | Right diabetic foot active ulceration | Unspecified |
| 857971000006110 |  | 857971000006106 | 857971000006110 | Right non-proliferative diabetic retinopathy | Unspecified |
| 857981000006113 |  | 857981000006109 | 857981000006113 | Right preproliferative diabetic retinopathy | Unspecified |
| 905621000006113 |  | 905621000006109 | 905621000006113 | [RFC] Diabetes | Unspecified |
| 906711000006116 |  | 906711000006100 | 906711000006116 | [RFC] Diabetic foot/leg ulcer | Unspecified |
| 908831000006118 |  | 908831000006102 | 908831000006118 | [RFC] Diabetes mellitus | Unspecified |
| 910491000006117 |  | 910491000006101 | 910491000006117 | [RFC] Diabetic leg ulcer | Unspecified |
| 910501000006113 |  | 910501000006109 | 910501000006113 | [RFC] Diabetic foot ulcer | Unspecified |
| 2415191000000113 | 1M8..00 | 944141000000108 | 2415191000000113 | Diabetic peripheral neuropathic pain | Unspecified |
| 2014951000006115 |  | 2014951000006104 | 2014951000006115 | New diabetic foot ulcer | Unspecified |

## **ICD10 codes used to identify diabetes in HES Admitted Patient Care data**

| icd | description | type |
| --- | --- | --- |
| E10 | Insulin-dependent diabetes mellitus | T1 |
| E10.0 | Insulin-dependent diabetes mellitus with coma | T1 |
| E10.1 | Insulin-dependent diabetes mellitus with ketoacidosis | T1 |
| E10.2 | Insulin-dependent diabetes mellitus with renal complications | T1 |
| E10.3 | Insulin-dependent diabetes mellitus with ophthalmic comps | T1 |
| E10.4 | Insulin-dependent diabetes mellitus with neurological comps | T1 |
| E10.5 | Insulin-dependent diabetes mellitus with periph circ comps | T1 |
| E10.6 | Insulin-dependent diabetes mellitus with other spec comps | T1 |
| E10.7 | Insulin-dependent diabetes mellitus with multiple comps | T1 |
| E10.8 | Insulin-dependent diabetes mellitus with unspec comps | T1 |
| E10.9 | Insulin-dependent diabetes mellitus without complications | T1 |
| O24.0 | Pre-existing diabetes mellitus, insulin-dependent | T1 |
| E11 | Non-insulin-dependent diabetes mellitus | T2 |
| E11.0 | Non-insulin-dependent diabetes mellitus with coma | T2 |
| E11.1 | Non-insulin-dependent diabetes mellitus with ketoacidosis | T2 |
| E11.2 | Non-insulin-dependent diabetes mellitus with renal comps | T2 |
| E11.3 | Non-insulin-dependent diabetes mellitus with ophthalm comps | T2 |
| E11.4 | Non-insulin-dependent diabetes mellitus with neuro comps | T2 |
| E11.5 | Non-insulin-depend diabetes mellitus with periph circ comp | T2 |
| E11.6 | Non-insulin-depend diabetes mellitus with other spec comp | T2 |
| E11.7 | Non-insulin-dependent diabetes mellitus with multiple comps | T2 |
| E11.8 | Non-insulin-dependent diabetes mellitus with unspec comps | T2 |
| E11.9 | Non-insulin-depend diabetes mellitus without complication | T2 |
| O24.1 | Pre-existing diabetes mellitus, non-insulin-dependent | T2 |
| E13 | Other specified diabetes mellitus | unspecified |
| E13.0 | Other specified diabetes mellitus | unspecified |
| E13.1 | Other specified diabetes mellitus | unspecified |
| E13.2 | Other specified diabetes mellitus | unspecified |
| E13.3 | Other specified diabetes mellitus | unspecified |
| E13.4 | Other specified diabetes mellitus | unspecified |
| E13.5 | Other specified diabetes mellitus | unspecified |
| E13.6 | Other specified diabetes mellitus | unspecified |
| E13.7 | Other specified diabetes mellitus | unspecified |
| E13.8 | Other specified diabetes mellitus | unspecified |
| E13.9 | Other specified diabetes mellitus | unspecified |
| E14 | Unspecified diabetes mellitus | unspecified |
| E14.0 | Unspecified diabetes mellitus with coma | unspecified |
| E14.1 | Unspecified diabetes mellitus with ketoacidosis | unspecified |
| E14.2 | Unspecified diabetes mellitus with renal complications | unspecified |
| E14.3 | Unspecified diabetes mellitus with ophthalmic complications | unspecified |
| E14.4 | Unspecified diabetes mellitus with neurological comps | unspecified |
| E14.5 | Unspecified diabetes mellitus with periph circulatory comps | unspecified |
| E14.6 | Unspecified diabetes mellitus with other specified comps | unspecified |
| E14.7 | Unspecified diabetes mellitus with multiple complications | unspecified |
| E14.8 | Unspecified diabetes mellitus with unspecified complications | unspecified |
| E14.9 | Unspecified diabetes mellitus without complications | unspecified |
| G59.0 | Diabetic mononeuropathy | unspecified |
| G63.2 | Diabetic polyneuropathy | unspecified |
| H28.0 | Diabetic cataract | unspecified |
| H36.0 | Diabetic retinopathy | unspecified |
| M14.2 | Diabetic arthropathy | unspecified |
| N08.3 | Glomerular disorders in diabetes mellitus | unspecified |
| O24.3 | Pre-existing diabetes mellitus, unspecified | unspecified |

## **Clinical codes used to identify heart failure in CPRD Aurum**

| medcodeid | readcode | snomedctconceptid | snomedctdescriptionid | term |
| --- | --- | --- | --- | --- |
| 2585431000006117 |  | 5375005 | 9994016 | Chronic left-sided congestive heart failure |
| 2660881000006116 |  | 10091002 | 17613015 | High output heart failure |
| 18472010 | G580000 | 10633002 | 18472010 | Acute congestive heart failure |
| 2805341000006119 |  | 19242006 | 32441014 | Pulmonary edema |
| 70653017 | G580.00 | 42343007 | 70653017 | Congestive heart failure |
| 493287011 | G580.11 | 42343007 | 70653017 | Congestive cardiac failure |
| 3182531000006110 |  | 42343007 | 70654011 | Congestive heart disease |
| 3182541000006117 |  | 42343007 | 493287011 | Congestive cardiac failure |
| 3182551000006115 |  | 42343007 | 493288018 | CCF - Congestive cardiac failure |
| 3182561000006118 |  | 42343007 | 493289014 | CHF - Congestive heart failure |
| 72934016 | G1yz100 | 43736008 | 72934016 | Rheumatic left ventricular failure |
| 3213831000006118 |  | 44313006 | 73920011 | Right heart failure secondary to left heart failure |
| 3283871000006117 |  | 48447003 | 80720010 | Chronic heart failure |
| 94251011 | G582.00 | 56675007 | 94251011 | Acute heart failure |
| 741681000006111 | G21z100 | 64715009 | 107545013 | Hypertensive heart disease NOS with CCF |
| 3552501000006119 |  | 64715009 | 107547017 | Hypertensive cardiopathy |
| 728671000006119 | G210100 | 83105008 | 1236017010 | Malignant hypertensive heart disease with congestive cardiac failure |
| 395772015 | G58z.00 | 84114007 | 139475013 | Heart failure NOS |
| 139475013 | G58..00 | 84114007 | 139475013 | Heart failure |
| 223981000000118 | G58z.12 | 84114007 | 139482012 | Cardiac failure NOS |
| 139482012 | G58..11 | 84114007 | 139482012 | Cardiac failure |
| 3868341000006118 |  | 84114007 | 1234906013 | HF - Heart failure |
| 3886041000006118 |  | 85232009 | 141303019 | Left heart failure |
| 141306010 | G581.00 | 85232009 | 141306010 | Left ventricular failure |
| 3886061000006119 |  | 85232009 | 201199018 | Left-sided heart failure |
| 3886071000006114 |  | 85232009 | 1235017018 | LVF - Left ventricular failure |
| 147247018 | G580100 | 88805009 | 147247018 | Chronic congestive heart failure |
| 510016018 | G580.14 | 92506005 | 153058012 | Biventricular congestive heart failure |
| 4005301000006110 |  | 92506005 | 510016018 | Biventricular failure |
| 4193361000006119 |  | 111283005 | 178503015 | Chronic left-sided heart failure |
| 216207010 | G5yy900 | 134401001 | 216207010 | Left ventricular systolic dysfunction |
| 251680018 | 14A6.00 | 161505003 | 251680018 | H/O: heart failure |
| 4540521000006111 |  | 161505003 | 2986453013 | History of heart failure |
| 504901000006118 | G211100 | 194767001 | 299653017 | Benign hypertensive heart disease with congestive cardiac failure |
| 741701000006114 | G232.00 | 194779001 | 299672017 | Hypertensive heart and renal disease with (congestive) heart failure |
| 789941000006117 | G234.00 | 194781004 | 299674016 | Hypertensive heart and renal disease with both (congestive) heart failure and renal failure |
| 300179017 | G580200 | 195111005 | 300179017 | Decompensated cardiac failure |
| 300180019 | G580300 | 195112003 | 300180019 | Compensated cardiac failure |
| 300190010 | G581000 | 195114002 | 300190010 | Acute left ventricular failure |
| 301695010 | H54z.00 | 196115007 | 301689014 | Pulmonary congestion and hypostasis NOS |
| 350413012 | G554011 | 233871002 | 350413012 | Congestive obstructive cardiomyopathy |
| 350484012 | SP11111 | 233924009 | 350484012 | Heart failure as a complication of care |
| 411506018 | G581.13 | 275514001 | 411506018 | Impaired left ventricular function |
| 784191000006110 | 33BA.00 | 275514001 | 411506018 | Impaired left ventricular function |
| 5573991000006118 |  | 275514001 | 1495323018 | Impaired left ventricular func |
| 451426015 | 8B29.00 | 308118002 | 451426015 | Cardiac failure therapy |
| 453099015 | 14AM.00 | 309634009 | 453099015 | H/O: Heart failure in last year |
| 1488804017 | 1O1..00 | 395105005 | 1488804017 | Heart failure confirmed |
| 1489358014 | G5yyA00 | 395704004 | 1489358014 | Left ventricular diastolic dysfunction |
| 1778488011 | G554000 | 399020009 | 1778488011 | Congestive cardiomyopathy |
| 6615851000006116 |  | 399020009 | 1786618013 | Dilated cardiomyopathy |
| 6615891000006110 |  | 399020009 | 1786621010 | DCM - Dilated cardiomyopathy |
| 2159197017 | 585f.00 | 407596008 | 2159197017 | Echocardiogram shows left ventricular systolic dysfunction |
| 2159198010 | 585g.00 | 407597004 | 2159198010 | Echocardiogram shows left ventricular diastolic dysfunction |
| 317955011 | R2y1000 | 410431009 | 2472092014 | Cardiorespiratory failure |
| 2549208013 | 8H2S.00 | 416683003 | 3082850014 | Emergency hospital admission for heart failure |
| 6914191000006115 |  | 417996009 | 2577903013 | Systolic heart failure |
| 6919191000006119 |  | 418304008 | 2577902015 | Diastolic heart failure |
| 7025691000006110 |  | 424404003 | 2645367010 | Decompensated chronic heart failure |
| 7056281000006118 |  | 426263006 | 2675256017 | Congestive heart failure due to left ventricular systolic dysfunction |
| 2675255018 | G580400 | 426611007 | 2675255018 | Congestive heart failure due to valvular disease |
| 2694523019 | G5yyD00 | 429589006 | 2694523019 | Left ventricular cardiac dysfunction |
| 7250571000006113 |  | 441481004 | 2816275019 | Chronic systolic heart failure |
| 7251291000006119 |  | 441530006 | 2816764017 | Chronic diastolic heart failure |
| 7475721000006114 |  | 471880001 | 2950981012 | Heart failure due to end stage congenital heart disease |
| 7507301000006110 |  | 698296002 | 2973838013 | Acute exacerbation of chronic congestive heart failure |
| 7510341000006110 |  | 698594003 | 2974742013 | Symptomatic congestive heart failure |
| 7573171000006116 |  | 703272007 | 3008211011 | Heart failure with reduced ejection fraction |
| 7573181000006118 |  | 703273002 | 3008136010 | Heart failure with reduced ejection fraction due to coronary artery disease |
| 7573191000006115 |  | 703274008 | 3008268013 | Heart failure with reduced ejection fraction due to myocarditis |
| 7573201000006117 |  | 703275009 | 3008202011 | Heart failure with reduced ejection fraction due to cardiomyopathy |
| 7573211000006119 |  | 703276005 | 3008139015 | Heart failure with reduced ejection fraction due to heart valve disease |
| 1734081000000112 | 8IE1.00 | 704097008 | 3011446018 | Referral to heart failure exercise programme declined |
| 13910671000006110 |  | 788950000 | 3786234019 | Heart failure with mid range ejection fraction |
| 8011111000006111 |  | 23341000119109 | 2984250012 | Congestive heart failure with right heart failure |
| 8030311000006111 |  | 96311000119109 | 2921293019 | Exacerbation of congestive heart failure |
| 226181000000110 | 9On..00 | 713781000000103 | 1565021000000117 | Left ventricular dysfunction monitoring administration |
| 905391000006119 |  | 905391000006103 | 905391000006119 | [RFC] Cardiac failure |

## **ICD10 codes used to identify heart failure in HES Admitted Patient Care data**

| icd | description |
| --- | --- |
| I11.0 | Hypertensive heart disease with heart failure |
| I13.0 | Hypertensive heart and chronic kidney disease with heart failure and stage 1 through stage 4 chronic kidney disease, or unspecified chronic kidney disease |
| I13.2 | Hypertensive heart and chronic kidney disease with heart failure and with stage 5 chronic kidney disease, or end stage renal disease |
| I50 | Heart failure |
| I50.0 | Congestive heart failure |
| I50.1 | Left ventricular failure, unspecified |
| I50.2 | Systolic (congestive) heart failure |
| I50.3 | Diastolic (congestive) heart failure |
| I50.4 | Combined systolic (congestive) and diastolic (congestive) heart failure |
| I50.8 | Other heart failure |
| I50.9 | Heart failure, unspecified |

## **Clinical codes used to identify myocardial infarction in CPRD Aurum**

| medcodeid | readcode | snomedctconceptid | snomedctdescriptionid | term |
| --- | --- | --- | --- | --- |
| 100681000006116 | G366.00 | 194868001 | 299824016 | Thrombosis of atrium, auricular appendage, and ventricle as current complications following acute myocardial infarction |
| 109915012 | G310.00 | 66189004 | 500341014 | Post-myocardial infarction syndrome |
| 109916013 | G310.11 | 66189004 | 109916013 | Dressler's syndrome |
| 116992017 | G307.00 | 70422006 | 116992017 | Acute subendocardial infarction |
| 118831000006118 | G35X.00 | 194856005 | 299808017 | Subsequent myocardial infarction of unspecified site |
| 1218860015 | G30y100 | 10273003 | 1218860015 | Acute papillary muscle infarction |
| 12220751000006113 |  | 194862000 | 3673281015 | Hemopericardium due to and following acute myocardial infarction |
| 12220761000006110 |  | 194862000 | 3673282010 | Haemopericardium due to and following acute myocardial infarction |
| 12220771000006115 |  | 194863005 | 3673273016 | Atrial septal defect due to and following acute myocardial infarction |
| 12220781000006117 |  | 194866002 | 3673278013 | Rupture of chordae tendinae due to and following acute myocardial infarction |
| 12220791000006119 |  | 194868001 | 3673277015 | Thrombosis of atrium, auricular appendage, and ventricle due to and following acute myocardial infarction |
| 12221301000006119 |  | 233847009 | 3673284011 | Cardiac rupture due to and following acute myocardial infarction |
| 1229885017 | G31y100 | 42531007 | 1229885017 | Microinfarction of heart |
| 1234005010 | G303.00 | 76593002 | 1234005010 | Acute inferoposterior infarction |
| 1234306015 | G30y200 | 79009004 | 1234306015 | Acute septal infarction |
| 1488382011 | G311500 | 394659003 | 1488382011 | Acute coronary syndrome |
| 158601000006116 | G363.00 | 194865003 | 299821012 | Rupture of cardiac wall without haemopericardium as current complication following acute myocardial infarction |
| 158611000006118 | G364.00 | 194866002 | 299822017 | Rupture of chordae tendinae as current complication following acute myocardial infarction |
| 159001000006119 | G365.00 | 194867006 | 299823010 | Rupture of papillary muscle as current complication following acute myocardial infarction |
| 1738171000006114 | 14AT.00 | 399211009 | 2986492018 | History of myocardial infarction |
| 1780491019 | G30X000 | 401303003 | 1780491019 | Acute ST segment elevation myocardial infarction |
| 1780501013 | G307100 | 401314000 | 1780501013 | Acute non-ST segment elevation myocardial infarction |
| 1786197015 | G30..12 | 398274000 | 1786197015 | Coronary thrombosis |
| 1786198013 | G30..16 | 398274000 | 1786198013 | Thrombosis - coronary |
| 208365015 | G38..00 | 129574000 | 208365015 | Postoperative myocardial infarction |
| 212061000006119 | G380.00 | 311792005 | 455418016 | Postoperative transmural myocardial infarction of anterior wall |
| 212071000006114 | G381.00 | 311793000 | 455419012 | Postoperative transmural myocardial infarction of inferior wall |
| 212081000006112 | G382.00 | 129574000 | 208365015 | Postoperative transmural myocardial infarction other sites |
| 212091000006110 | G383.00 | 129574000 | 208365015 | Postoperative transmural myocardial infarction unspec site |
| 216351000006118 | G501.00 | 233885007 | 350431013 | Post infarction pericarditis |
| 219521000000119 | G30..11 | 22298006 | 37443015 | Attack - heart |
| 219531000000117 | G30..15 | 57054005 | 94884017 | MI - acute myocardial infarction |
| 230021000006115 | G32..12 | 399211009 | 2986492018 | Personal history of myocardial infarction |
| 256452010 | 323..00 | 164865005 | 256452010 | ECG: myocardial infarction |
| 256454011 | 3232.00 | 164867002 | 256454011 | ECG: old myocardial infarction |
| 256455012 | 3233.00 | 164868007 | 256455012 | ECG: antero-septal infarct. |
| 256457016 | 3234.00 | 164869004 | 256457016 | ECG:posterior/inferior infarct |
| 256458014 | 3235.00 | 164870003 | 256458014 | ECG: subendocardial infarct |
| 256459018 | 3236.00 | 164871004 | 256459018 | ECG: lateral infarction |
| 256460011 | 323Z.00 | 164865005 | 256452010 | ECG: myocardial infarct NOS |
| 2619484018 | G311011 | 194821006 | 2619484018 | MI - Myocardial infarction aborted |
| 2663441000006117 |  | 10273003 | 17900011 | Acute infarction of papillary muscle |
| 2855301000006112 |  | 22298006 | 37436014 | Myocardial infarction |
| 2855311000006110 |  | 22298006 | 37441018 | Infarction of heart |
| 2855321000006119 |  | 22298006 | 37442013 | Cardiac infarction |
| 2855341000006114 |  | 22298006 | 1784872019 | MI - Myocardial infarction |
| 2855351000006111 |  | 22298006 | 1784873012 | Myocardial infarct |
| 299707016 | G301.00 | 54329005 | 1231324017 | Acute anterior myocardial infarction |
| 299708014 | G301000 | 52035003 | 3038718019 | Acute anteroapical infarction |
| 299709018 | G301z00 | 54329005 | 1231324017 | Anterior myocardial infarction NOS |
| 299710011 | G304.00 | 233838001 | 350371016 | Acute posterior myocardial infarction |
| 299711010 | G305.00 | 58612006 | 1231860015 | Acute lateral myocardial infarction |
| 299712015 | G306.00 | 194802003 | 299712015 | True posterior myocardial infarction |
| 299714019 | G308.00 | 73795002 | 122557015 | Acute myocardial infarction of inferior wall |
| 299718016 | G30y.00 | 57054005 | 94884017 | Other acute myocardial infarction |
| 299719012 | G30y000 | 194809007 | 299719012 | Acute atrial infarction |
| 299720018 | G30yz00 | 57054005 | 94884017 | Other acute myocardial infarction NOS |
| 299721019 | G30z.00 | 57054005 | 94884017 | Acute myocardial infarction NOS |
| 299808017 | G35..00 | 194856005 | 299808017 | Subsequent myocardial infarction |
| 299811016 | G350.00 | 194857001 | 299811016 | Subsequent myocardial infarction of anterior wall |
| 299812011 | G351.00 | 194858006 | 299812011 | Subsequent myocardial infarction of inferior wall |
| 299813018 | G353.00 | 194856005 | 299808017 | Subsequent myocardial infarction of other sites |
| 300881018 | Gyu3500 | 194856005 | 299808017 | [X]Subsequent myocardial infarction of other sites |
| 300882013 | Gyu3600 | 194856005 | 299808017 | [X]Subsequent myocardial infarction of unspecified site |
| 3185771000006112 |  | 42531007 | 70952012 | Microinfarct of heart |
| 3343471000006116 |  | 52035003 | 86618010 | Acute anteroapical myocardial infarction |
| 3381601000006117 |  | 54329005 | 90302019 | Acute myocardial infarction of anterior wall |
| 3427201000006111 |  | 57054005 | 1231678011 | AMI - Acute myocardial infarction |
| 3452181000006112 |  | 58612006 | 97399018 | Acute myocardial infarction of lateral wall |
| 350376014 | G30..17 | 233843008 | 350376014 | Silent myocardial infarction |
| 3565871000006113 |  | 65547006 | 108912018 | Acute myocardial infarction of inferolateral wall |
| 3576371000006117 |  | 66189004 | 109915012 | Postmyocardial infarction syndrome |
| 3576391000006116 |  | 66189004 | 109917016 | Postmyocardial infarction pericarditis |
| 362461000006119 | Gyu3400 | 57054005 | 94884017 | [X]Acute transmural myocardial infarction of unspecif site |
| 3641641000006116 |  | 70211005 | 116613011 | Acute myocardial infarction of anterolateral wall |
| 3645351000006114 |  | 70422006 | 116993010 | Acute nontransmural infarction |
| 3699911000006119 |  | 73795002 | 122559017 | Acute myocardial infarction of diaphragmatic wall |
| 3699921000006110 |  | 73795002 | 1233665015 | Acute inferior myocardial infarction |
| 37443015 | G30..14 | 22298006 | 37443015 | Heart attack |
| 3745741000006117 |  | 76593002 | 127193015 | Acute myocardial infarction of inferoposterior wall |
| 3784911000006111 |  | 79009004 | 131106018 | Acute myocardial infarction of septum |
| 3784921000006115 |  | 79009004 | 131107010 | Acute myocardial infarction of septum alone |
| 4031011 | G32..00 | 1755008 | 4031011 | Old myocardial infarction |
| 4032016 | G32..11 | 1755008 | 4032016 | Healed myocardial infarction |
| 408571000006116 | Gyu3100 | 302049001 | 443587013 | [X]Other current complicatns following acute myocard infarct |
| 447324018 | G309.00 | 304914007 | 447324018 | Acute Q-wave infarct |
| 450322013 | G307000 | 307140009 | 450322013 | Acute non-Q wave infarction |
| 451369010 | 14AH.00 | 308065005 | 451369010 | H/O: Myocardial infarction in last year |
| 4540461000006118 |  | 161502000 | 2986996013 | History of myocardial infarct at age less than sixty |
| 4540481000006111 |  | 161503005 | 2986493011 | History of myocardial infarct at age greater than sixty |
| 455422014 | G384.00 | 311796008 | 455422014 | Postoperative subendocardial myocardial infarction |
| 455423016 | G38z.00 | 129574000 | 208365015 | Postoperative myocardial infarction, unspecified |
| 455641000006112 | G300.00 | 70211005 | 1233238016 | Acute anterolateral myocardial infarction |
| 455651000006114 | G301100 | 62695002 | 104192010 | Acute anteroseptal myocardial infarction |
| 457531000006110 | G302.00 | 65547006 | 1232697013 | Acute inferolateral myocardial infarction |
| 458410010 | G33z500 | 314116003 | 458410010 | Post infarct angina |
| 4586041000006115 |  | 164865005 | 3300376010 | Electrocardiographic myocardial infarction |
| 4586051000006118 |  | 164865005 | 3300377018 | EKG: myocardial infarction |
| 4586091000006112 |  | 164867002 | 3300311011 | EKG: old myocardial infarction |
| 4586101000006118 |  | 164867002 | 3300312016 | Electrocardiographic old myocardial infarction |
| 4586111000006115 |  | 164868007 | 2695135018 | Anteroseptal infarction on electrocardiogram |
| 4586131000006114 |  | 164868007 | 256456013 | ECG: anteroseptal infarction |
| 4586141000006116 |  | 164868007 | 2619217019 | Electrocardiogram: anteroseptal infarction |
| 4586151000006119 |  | 164868007 | 2820892012 | ECG: antero-septal infarct |
| 4586171000006112 |  | 164869004 | 3300251011 | EKG:posterior/inferior infarct |
| 4586181000006110 |  | 164869004 | 3300252016 | Electrocardiographic posterior/inferior infarct |
| 4586201000006111 |  | 164870003 | 3300441012 | EKG: subendocardial infarct |
| 4586211000006114 |  | 164870003 | 3300442017 | Electrocardiographic subendocardial infarct |
| 4586221000006118 |  | 164871004 | 2692104017 | Lateral infarction on electrocardiogram |
| 4586241000006113 |  | 164871004 | 2619220010 | Electrocardiogram: lateral infarction |
| 460681000006116 | G30X.00 | 57054005 | 94884017 | Acute transmural myocardial infarction of unspecif site |
| 4775881000006117 |  | 194802003 | 2535851011 | True posterior wall infarction |
| 4775891000006119 |  | 194809007 | 2470032018 | Acute myocardial infarction of atrium |
| 4775931000006111 |  | 194821006 | 2619483012 | Aborted myocardial infarction |
| 4776171000006116 |  | 194856005 | 299809013 | Reinfarction of myocardium |
| 4776221000006110 |  | 194862000 | 299817017 | Hemopericardium as current complication following acute myocardial infarction |
| 4776251000006118 |  | 194865003 | 299820013 | Rupture of cardiac wall without hemopericardium as current complication following acute myocardial infarction |
| 498031000006112 | G361.00 | 194863005 | 299818010 | Atrial septal defect as current complication following acute myocardial infarction |
| 5056461000006113 |  | 233843008 | 350377017 | MI - Silent myocardial infarction |
| 5056501000006113 |  | 233846000 | 350382012 | Post-infarction ventricular septal defect |
| 5056521000006115 |  | 233846000 | 3333538017 | Defect of ventricular septum following myocardial infarction |
| 5056941000006119 |  | 233885007 | 350431013 | Post-infarction pericarditis |
| 5056951000006117 |  | 233885007 | 350432018 | Pericarditis following myocardial infarction |
| 537751000006115 | G30..13 | 233847009 | 350383019 | Cardiac rupture after acute myocardial infarction |
| 543291000006110 | G36..00 | 371068009 | 1209776017 | Myocardial infarction with complication |
| 5935321000006111 |  | 304914007 | 447323012 | Acute Q wave myocardial infarction |
| 5974481000006116 |  | 308065005 | 2986726013 | History of myocardial infarction in last year |
| 6056601000006112 |  | 315287002 | 459792010 | DIGAMI - diabetes mellitus insulin-glucose infusion in acute myocardial infarction |
| 616081000006113 | 889A.00 | 315287002 | 459791015 | Diabetes mellitus insulin-glucose infusion in acute myocardial infarction |
| 6348651000006112 |  | 371068009 | 6348651000006112 | Myocardial infarction with complication |
| 6619191000006115 |  | 399211009 | 1786753012 | Past history of myocardial infarction |
| 6619201000006117 |  | 399211009 | 545891000000115 | Previous myocardial infarction |
| 6651221000006117 |  | 401303003 | 2840840013 | STEMI - ST elevation myocardial infarction |
| 6651391000006114 |  | 401314000 | 1787486017 | NSTEMI - Non-ST segment elevation MI |
| 67081000006119 | G362.00 | 233846000 | 350381017 | Ventricular septal defect as current complication following acute myocardial infarction |
| 682481000006118 | G311000 | 194821006 | 2619484018 | Myocardial infarction aborted |
| 810811000006116 | 14A3.00 | 161502000 | 251675010 | H/O: myocardial infarct at less than 60 |
| 810821000006112 | 14A4.00 | 161503005 | 251678012 | H/O: myocardial infarct at greater than 60 |
| 813961000006116 | G360.00 | 194862000 | 299816014 | Haemopericardium as current complication following acute myocardial infarction |
| 884141000006116 | G30..98 | 57054005 | 884141000006116 | Coronary thrombosis |
| 884151000006119 | G30..99 | 57054005 | 884151000006119 | Myocardial Infarction |
| 905351000006113 |  | 905351000006109 | 905351000006113 | [RFC] Myocardial infarction (MI) |
| 931961000006117 | G31y099 | 194823009 | 931961000006117 | Acute coronary syndrome |
| 932081000006118 |  | 932081000006102 | 932081000006118 | First myocardial infarction |
| 94884017 | G30..00 | 57054005 | 94884017 | Acute myocardial infarction |
| 967931000006114 | G30B.00 | 15990001 | 27071012 | Acute myocardial infarction of posterolateral wall |
| 1576271000006117 |  | 1576271000006101 | 1576271000006117 | cause of death- myocardial infarction |
| 1576301000006115 |  | 1576301000006104 | 1576301000006115 | cause of death- acute myocardial infarction |

## **ICD10 codes used to identify myocardial infarction in HES Admitted Patient Care data**

| icd | description |
| --- | --- |
| I21 | Acute myocardial dysfunction |
| I21.0 | Acute transmural myocardial infarction of anterior wall |
| I21.1 | Acute transmural myocardial infarction of interior wall |
| I21.2 | Acute transmural myocardial infarction of other sites |
| I21.3 | Acute transmural myocardial infarction of unspecified site |
| I21.4 | Acute subendocardial myocardial infarction |
| I21.9 | Acute myocardial infarction, unspecified |
| I22 | Subsequent myocardial infarction |
| I22.0 | Subsequent myocardial infarction of anterior wall |
| I22.1 | Subsequent myocardial infarction of inferior wall |
| I22.2 | Subsequent non-ST elevation (NSTEMI) myocardial infarction |
| I22.8 | Subsequent myocardial infarction of other sites |
| I22.9 | Subsequent myocardial infarction of unspecified site |
| I23 | Certain current complications following acute myocardial infection |
| I23.0 | Haemopericardium as current complication following acute myocardial infarction |
| I23.2 | Ventricular septal defect as current complication following acute myocardial infarction |
| I23.4 | Rupture of chordae tendineae as current complication following acute myocardial infarction |
| I23.5 | Rupture of papillary muscle as current complication following acute myocardial infarction |
| I23.6 | Thrombosis of atrium, auricular appendage, and ventricle as current complications following acute myocardial infarction |
| I23.7 | Postinfarction angina |
| I23.8 | Other current complications following acute myocardial infarction |
| I24.1 | Dressler syndrome |
| I25.2 | Old myocardial infarction |
| I25.6 | Silent myocardial ischaemia |

## **ICD10 codes used to identify sepsis in HES Admitted Patient Care data**

| icd | description |
| --- | --- |
| A02.1 | salmonella sepsis |
| A20.7 | septicaemic plague |
| A22.7 | anthrax sepsis |
| A24.1 | acute and fulminating melioidosis |
| A26.7 | erysipelothrix sepsis |
| A32.7 | listerial sepsis |
| A39.1 | waterhouse-friderichsen syndrome |
| A39.2 | acute meningococcaemia, unspecified |
| A39.3 | Chronic meningococcaemia |
| A39.4 | Meningococcaemia, unspecified |
| A40.0 | Sepsis due to streptococcus, group A |
| A40.1 | Sepsis due to streptococcus, group B |
| A40.2 | sepsis due to streptococcus, group d |
| A40.3 | Sepsis due to Streptococcus pneumoniae |
| A40.8 | Other streptococcal sepsis |
| A40.9 | Streptococcal sepsis, unspecified |
| A41.0 | sepsis due to staphylococcus aureus |
| A41.01 | Sepsis due to Methicillin susceptible Staphylococcus aureus |
| A41.02 | Sepsis due to Methicillin resistant Staphylococcus aureus |
| A41.1 | Sepsis due to other specified staphylococcus |
| A41.2 | Sepsis due to unspecified staphylococcus |
| A41.3 | Sepsis due to Haemophilus influenzae |
| A41.4 | Sepsis due to anaerobes |
| A41.5 | sepsis due to other gram-negative organisms |
| A41.50 | Sepsis due to other Gram-negative organisms |
| A41.51 | Sepsis due to Escherichia coli [E. coli] |
| A41.52 | Sepsis due to Pseudomonas |
| A41.53 | Sepsis due to Serratia |
| A41.59 | Other Gram-negative sepsis |
| A41.8 | other specified sepsis |
| A41.81 | Sepsis due to Enterococcus |
| A41.89 | Other specified sepsis |
| A41.9 | Sepsis, unspecified organism |
| A42.7 | Actinomycotic sepsis |
| A48.3 | toxic shock syndrome |
| A54.86 | Gonococcal sepsis |
| B00.7 | Disseminated herpesviral disease |
| B37.7 | Candidal sepsis |
| R57.2 | septic shock |
| R57.8 | other shock |
| R65.0 | systemic inflammatory response syndrome of infectious origin without organ failure |
| R65.1 | systemic inflammatory response syndrome of infectious origin with organ failure |
| R65.20 | Severe sepsis without septic shock |
| R65.21 | Severe sepsis with septic shock |

## **Clinical codes used to identify kidney transplant in CPRD Aurum**

| medcodeid | readcode | snomedctconceptid | snomedctdescriptionid | term |
| --- | --- | --- | --- | --- |
| 2814151000006118 |  | 19784002 | 33316011 | Implant of mechanical kidney |
| 2814161000006116 |  | 19784002 | 2579159012 | Artificial kidney implant |
| 2868911000006114 |  | 23137002 | 38855018 | Anesthesia for renal transplant, recipient |
| 2868901000006111 |  | 23137002 | 481524011 | Anaesthesia for renal transplant, recipient |
| 2898681000006112 |  | 24955000 | 41829010 | Removal of mechanical kidney |
| 3292751000006114 |  | 48994000 | 81622016 | Renal homotransplantation with unilateral recipient nephrectomy |
| 3455181000006115 |  | 58797008 | 97706015 | Complication of transplanted kidney |
| 3455171000006118 |  | 58797008 | 2695164010 | Disorder of transplanted kidney |
| 272326017 | 7B00y00 | 70536003 | 117163013 | Other specified transplantation of kidney |
| 3647021000006114 |  | 70536003 | 117163013 | Transplant of kidney |
| 3647031000006112 |  | 70536003 | 117167014 | Kidney transplantation |
| 117168016 | 7B00.98 | 70536003 | 117168016 | Renal transplant |
| 3647051000006117 |  | 70536003 | 501591017 | Tx - Renal transplantation |
| 3647061000006115 |  | 70536003 | 501592012 | Kidney transplant |
| 3647071000006110 |  | 70536003 | 501593019 | Tx - Kidney transplantation |
| 272327014 | 7B00z00 | 70536003 | 501594013 | Transplantation of kidney NOS |
| 501594013 | 7B00.00 | 70536003 | 501594013 | Transplantation of kidney |
| 3647091000006111 |  | 70536003 | 501595014 | Renal graft |
| 866731000006115 | 7B00.97 | 70536003 | 866731000006115 | Kidney replacement NOS |
| 866751000006110 | 7B00.99 | 70536003 | 866751000006110 | Kidney replacement |
| 3653841000006116 |  | 70948008 | 117847019 | Transplanted kidney |
| 3653831000006114 |  | 70948008 | 501724016 | Structure of transplanted kidney |
| 3732051000006114 |  | 75780002 | 125856010 | Artificial kidney |
| 3732061000006111 |  | 75780002 | 2672346013 | Artificial kidney, device |
| 754061000006111 | TB00100 | 88797001 | 147234011 | Kidney transplant with complication, without blame |
| 177251000006119 | TB00111 | 88797001 | 147234011 | Renal transplant with complication, without blame |
| 4215521000006118 |  | 112896000 | 184355018 | Replacement of mechanical kidney |
| 4218231000006113 |  | 113127005 | 186851015 | Kidney transplant evaluation |
| 4322131000006118 |  | 119980000 | 184352015 | Kidney implantation |
| 251923016 | 14S2.00 | 161665007 | 251923016 | H/O: kidney recipient |
| 4542521000006112 |  | 161665007 | 2666912015 | History of - kidney recipient |
| 460644012 | ZV42000 | 161665007 | 2793932016 | History of renal transplant |
| 4542501000006119 |  | 161665007 | 4542501000006119 | History of renal transplant |
| 4668951000006113 |  | 175899003 | 272315011 | Autotransplantation of kidney |
| 500371000006119 | 7B00000 | 175899003 | 272315011 | Autotransplant of kidney |
| 4668961000006110 |  | 175899003 | 2475528018 | Autotransplant kidney |
| 4668971000006115 |  | 175899003 | 2475529014 | Renal autotransplantation |
| 4668981000006117 |  | 175899003 | 2475530016 | Renal autotransplantation or reimplantation of kidney |
| 90471000006113 | 7B00100 | 175901007 | 272318013 | Live donor renal transplant |
| 4669011000006116 |  | 175901007 | 272319017 | Live donor renal allotransplant |
| 272320011 | 7B00111 | 175901007 | 272320011 | Allotransplantation of kidney from live donor |
| 4669031000006110 |  | 175901007 | 272321010 | Live donor renal allograft |
| 399381000000115 | 7B00500 | 175902000 | 272323013 | Allotransplantation of kidney from cadaver NEC |
| 4669051000006115 |  | 175902000 | 272323013 | Cadaver renal allotransplant |
| 4669061000006118 |  | 175902000 | 272324019 | Cadaver renal allograft |
| 478131000006115 | 7B00211 | 175902000 | 272325018 | Allotransplantation of kidney from cadaver |
| 272325018 | 7B00212 | 175902000 | 272325018 | Cadaveric renal transplant |
| 90461000006118 | 7B00200 | 175902000 | 272325018 | Transplantation of kidney from cadaver |
| 272389014 | 7B06300 | 175948009 | 272389014 | Exploration of renal transplant |
| 4669451000006113 |  | 175948009 | 272390017 | Exploration of kidney transplant |
| 424791000006110 | Kyu1C00 | 197747000 | 304045012 | [X]Renal tubulo-interstitial disorders/transplant rejection |
| 177341000006111 | K0B5.00 | 197747000 | 304045012 | Renal tubulo-interstitial disorders in transplant rejection |
| 324973015 | SP08300 | 213150003 | 324973015 | Kidney transplant failure and rejection |
| 988961000006115 | SP08399 | 213150003 | 988961000006115 | Renal transplant rejected |
| 5059341000006114 |  | 234046009 | 350673013 | Transplant renal vein thrombosis |
| 353974015 | 7B00600 | 236138007 | 353974015 | Xenograft renal transplant |
| 5094171000006114 |  | 236436003 | 354420011 | End stage renal failure with renal transplant |
| 5096031000006117 |  | 236569000 | 354609015 | Primary non-function of renal transplant |
| 615611000006116 | SP08011 | 236570004 | 354610013 | Det.ren.func.after ren.transpl |
| 354610013 | SP08R00 | 236570004 | 354610013 | Renal transplant rejection |
| 900121000006113 | SP08199 | 236570004 | 900121000006113 | Renal transplant rejected |
| 5096051000006112 |  | 236571000 | 354611012 | Hyperacute rejection of renal transplant |
| 354612017 | SP08C00 | 236572007 | 354612017 | Accelerated rejection of renal transplant |
| 354613010 | SP08V00 | 236573002 | 354613010 | Very mild acute rejection of renal transplant |
| 354614016 | SP08V11 | 236573002 | 354614016 | Borderline changes of acute rejection |
| 354615015 | SP08H00 | 236574008 | 354615015 | Acute rejection of renal transplant |
| 354616019 | SP08E00 | 236575009 | 354616019 | Acute rejection of renal transplant - grade I |
| 354617011 | SP08F00 | 236576005 | 354617011 | Acute rejection of renal transplant - grade II |
| 354618018 | SP08G00 | 236577001 | 354618018 | Acute rejection of renal transplant - grade III |
| 354619014 | SP08J00 | 236578006 | 354619014 | Chronic rejection of renal transplant |
| 5096141000006114 |  | 236578006 | 354620015 | Chronic allograft nephropathy |
| 354621016 | SP08J11 | 236578006 | 354621016 | Chronic transplant nephropathy |
| 5096171000006118 |  | 236579003 | 354622011 | Mild chronic transplant nephropathy |
| 354623018 | SP08K00 | 236579003 | 354623018 | Chronic rejection of renal transplant - grade 1 |
| 354624012 | SP08L00 | 236580000 | 354624012 | Chronic rejection of renal transplant - grade II |
| 5096191000006117 |  | 236580000 | 354625013 | Moderate chronic transplant nephropathy |
| 5096211000006116 |  | 236581001 | 354626014 | Severe chronic transplant nephropathy |
| 354627017 | SP08M00 | 236581001 | 354627017 | Chronic rejection of renal transplant - grade III |
| 354628010 | SP08D00 | 236582008 | 354628010 | Acute-on-chronic rejection of renal transplant |
| 5096231000006110 |  | 236583003 | 354629019 | Failed renal transplant |
| 5096241000006117 |  | 236584009 | 354630012 | Perfusion injury of renal transplant |
| 354631011 | SP08c00 | 236585005 | 354631011 | De novo transplant disease |
| 354632016 | SP08b00 | 236586006 | 354632016 | De novo glomerulonephritis |
| 5096271000006113 |  | 236587002 | 354633014 | Transplant glomerulopathy |
| 5096281000006111 |  | 236588007 | 354634015 | Transplant glomerulopathy - early form |
| 5096291000006114 |  | 236589004 | 354635019 | Transplant glomerulopathy - late form |
| 5157591000006112 |  | 241249008 | 361284015 | Renal transplant arteriogram |
| 5157601000006116 |  | 241250008 | 361285019 | Renal transplant venogram |
| 5157611000006118 |  | 241251007 | 361286018 | Renal transplant venous sampling |
| 5161281000006112 |  | 241484009 | 361595010 | US scan of transplant kidney |
| 5161291000006110 |  | 241484009 | 2619719015 | Ultrasound scan of transplant kidney |
| 394365013 | 7B01500 | 265550007 | 394365013 | Transplant nephrectomy |
| 5486891000006118 |  | 265550007 | 394366014 | Removal of transplanted kidney |
| 877641000006113 | 7L1A099 | 265764009 | 877641000006113 | Extracorporeal kidney |
| 413415016 | SP08N00 | 277010001 | 413415016 | Unexplained episode of renal transplant dysfunction |
| 5593651000006110 |  | 277011002 | 413416015 | Pre-existing disease in renal transplant |
| 5647611000006119 |  | 281112005 | 419040013 | Entire transplanted kidney |
| 5647621000006110 |  | 281112005 | 3329266012 | Entire transplant kidney |
| 5686451000006112 |  | 284348003 | 422991013 | Removal of rejected kidney |
| 1905531000006112 | 7B01900 | 284348003 | 422992018 | Excision of rejected transplanted kidney |
| 422992018 | 7B01511 | 284348003 | 422992018 | Excision of rejected transplanted kidney |
| 5717311000006112 |  | 286618009 | 426012017 | Xenograft kidney |
| 5717321000006116 |  | 286619001 | 426013010 | Pig xenograft kidney |
| 5717341000006111 |  | 286621006 | 426015015 | Primate xenograft kidney |
| 5717381000006117 |  | 286624003 | 426019014 | Allograft kidney |
| 5717391000006119 |  | 286625002 | 426020015 | Cadaver allograft kidney |
| 5717401000006117 |  | 286626001 | 426021016 | Live donor allograft kidney |
| 5717551000006117 |  | 286636009 | 426034015 | Autograft kidney |
| 5730251000006114 |  | 288182009 | 427777019 | Extracorporeal kidney |
| 6024541000006117 |  | 313030004 | 456855017 | Donor renal transplantation |
| 2160027017 | 9b8K.00 | 408477008 | 2160027017 | Transplantation surgery |
| 6932431000006113 |  | 419139005 | 2578274014 | Fluoroscopic angiography of renal transplant |
| 6932441000006115 |  | 419139005 | 2580477012 | Renal transplant angiogram |
| 6933851000006114 |  | 419224002 | 2578421018 | Fluoroscopic angioplasty of renal transplant |
| 6933861000006111 |  | 419224002 | 2580569011 | Fluoroscopy and angioplasty of renal transplant |
| 6948341000006114 |  | 420106004 | 2578093016 | Fluoroscopic venography of renal transplant |
| 6948351000006111 |  | 420106004 | 2580404018 | Renal transplant venogram |
| 6961631000006114 |  | 420852008 | 2616388015 | Kidney graft - material |
| 6963731000006116 |  | 420970005 | 2616394011 | Donor kidney graft |
| 7054541000006117 |  | 426136000 | 2674089016 | Delayed renal graft function |
| 7054551000006115 |  | 426136000 | 2674090013 | Delayed kidney graft function |
| 7090481000006112 |  | 428575007 | 2692589017 | Hypertension secondary to kidney transplant |
| 380101000000116 | 7B0F300 | 428645009 | 2693113012 | Examination of recipient after kidney transplant |
| 7091541000006113 |  | 428645009 | 2296631000000110 | Post-transplantation of kidney examination of recipient |
| 7094321000006114 |  | 428830000 | 2694234017 | Pretransplant evaluation of kidney recipient |
| 7103511000006119 |  | 429451003 | 2693948016 | Disorder related to renal transplantation |
| 7104121000006117 |  | 429490004 | 2695597017 | Disorder affecting transplanted structure |
| 7150611000006118 |  | 431988002 | 2766420010 | Doppler ultrasonography (US) of transplanted kidney |
| 7150621000006114 |  | 431988002 | 2766421014 | Doppler ultrasonography of transplanted kidney |
| 7150631000006112 |  | 431988002 | 3307531014 | Doppler ultrasound scan of transplanted kidney |
| 7150641000006119 |  | 431988002 | 3307532019 | Doppler ultrasound of transplanted kidney |
| 7150601000006116 |  | 431988002 | 694521000000116 | US doppler scan of transplanted kidney |
| 7212381000006113 |  | 438661003 | 2790986011 | Renal transplant biopsy sample |
| 7212371000006110 |  | 438661003 | 2794543012 | Specimen from transplanted kidney obtained by biopsy |
| 7214301000006113 |  | 438804005 | 2794675019 | Tissue specimen from transplanted kidney |
| 7237321000006119 |  | 440449002 | 2790971017 | Magnetic resonance angiography of transplanted kidney |
| 7237331000006116 |  | 440449002 | 3027721011 | MR angiography of transplanted kidney |
| 7238471000006113 |  | 440512006 | 2792687013 | Biopsy of transplanted kidney using ultrasound guidance |
| 7238481000006111 |  | 440512006 | 1647441000000113 | Ultrasonography guided biopsy of transplanted kidney |
| 7279701000006119 |  | 443578000 | 2838725016 | Radionuclide imaging of transplanted kidney |
| 7279711000006116 |  | 443578000 | 1178741000000116 | Radionuclide study of transplanted kidney |
| 7484221000006112 |  | 473195006 | 2956209012 | Normal renal function of transplanted kidney |
| 7502751000006112 |  | 697982000 | 2968385014 | Referral to surgical transplant service for renal transplant evaluation |
| 7569831000006116 |  | 703048006 | 3007007010 | Post-renal transplant vesicoureteric reflux |
| 7569841000006114 |  | 703048006 | 3007010015 | Vesicoureteric reflux after renal transplant |
| 7613121000006116 |  | 707148007 | 3028876010 | Recurrent post-transplant renal disease |
| 372621000000114 | 7B00300 | 711411006 | 3078356013 | Allotransplantation of kidney from beating heart cadaver |
| 7688001000006116 |  | 711411006 | 2700121000000114 | Allotransplantation of kidney with DBD (donation after brain stem death) |
| 1549841000006116 | 7B00400 | 711413009 | 3078484016 | Allotransplantation of kidney from non-beating heart cadaver |
| 7688041000006119 |  | 711413009 | 2700131000000111 | Allotransplantation of kidney with DCD (donation after cardiac death) |
| 7688541000006114 |  | 711446003 | 3078442012 | Transplantation of kidney regime |
| 1550021000006110 | 7B0Fy00 | 711446003 | 3078450015 | OS interventions associated with transplantation of kidney |
| 380061000000118 | 7B0Fz00 | 711446003 | 3078450015 | Interventions associated with transplantation of kidney NOS |
| 366671000000118 | 7B0F.00 | 711446003 | 3078450015 | Interventions associated with transplantation of kidney |
| 7700671000006112 |  | 713183000 | 3288326010 | Cadaver donor for kidney transplant |
| 7710031000006116 |  | 713824006 | 3297830016 | Structure of renal artery of transplanted kidney |
| 7710051000006111 |  | 713825007 | 3297789015 | Renal artery stenosis of transplanted kidney |
| 7710041000006114 |  | 713825007 | 3297818010 | Transplant renal artery stenosis |
| 7714921000006112 |  | 714153000 | 3298559011 | Chronic kidney disease stage 5 with transplant |
| 7714931000006110 |  | 714153000 | 3298659016 | Chronic kidney disease 5t |
| 7714941000006117 |  | 714153000 | 3298692010 | CKD (chronic kidney disease) stage 5t |
| 7766381000006118 |  | 718030008 | 3310956017 | Antegrade insertion of ureteric stent into ureter of transplanted kidney using fluoroscopic guidance |
| 7766371000006116 |  | 718030008 | 3310957014 | Fluoroscopy guided antegrade insertion of ureteric stent into ureter of transplanted kidney |
| 7766401000006118 |  | 718031007 | 3310953013 | Retrograde insertion of ureteric stent into ureter of transplanted kidney using fluoroscopic guidance |
| 7766391000006115 |  | 718031007 | 3310954019 | Fluoroscopy guided retrograde insertion of ureteric stent into ureter of transplanted kidney |
| 7785581000006115 |  | 719352000 | 3316067010 | Kidney transplant recipient |
| 7932051000006112 |  | 731627000 | 3462259012 | Entire renal artery of transplanted kidney |
| 3527360016 |  | 737295003 | 3527360016 | Transplanted kidney present |
| 12019461000006111 |  | 764643003 | 3654384018 | Fluoroscopy guided removal of nephrostomy tube from transplanted kidney |
| 12019471000006116 |  | 764643003 | 3654386016 | Removal of nephrostomy tube from transplanted kidney using fluoroscopic guidance |
| 12031641000006115 |  | 765478004 | 3658922018 | Allotransplantation of left kidney |
| 12031651000006118 |  | 765479007 | 3658924017 | Allotransplantation of right kidney |
| 7985891000006113 |  | 6471000179103 | 3313005013 | Transplantation of kidney and pancreas |
| 7985971000006118 |  | 6501000179109 | 3315654019 | Laparoscopic biopsy of transplanted kidney |
| 386181000000111 | 7B0F100 | 238251000000109 | 386181000000111 | Pre-transplantation of kidney work-up, recipient |
| 387911000000115 | 7B0F200 | 239101000000109 | 387911000000115 | Pre-transplantation of kidney work-up, live donor |
| 8115641000006112 |  | 362361000000102 | 683061000000116 | Kidney transplant tissue sample |
| 8117811000006119 |  | 366961000000102 | 692851000000118 | Renal transplant recipient |
| 11910651000006112 |  | 399241000000100 | 941921000000117 | [V]Kidney transplanted |
| 866961000006110 | 7B06.99 | 591431000000109 | 866961000006110 | Artificial kidney implant |
| 2189151000000117 | SP08T00 | 843691000000100 | 2189151000000117 | Urological complication of renal transplant |
| 2191221000000118 | SP08W00 | 844661000000109 | 2191221000000118 | Vascular complication of renal transplant |
| 2197961000000113 | SP08X00 | 847791000000101 | 2197961000000113 | Rupture of artery of transplanted kidney |
| 2198001000000111 | SP08Y00 | 847811000000100 | 2198001000000111 | Rupture of vein of transplanted kidney |
| 2198141000000110 | SP08P00 | 847881000000107 | 2198141000000110 | Stenosis of vein of transplanted kidney |
| 2209021000000112 | SP08S00 | 852981000000100 | 2209021000000112 | Aneurysm of vein of transplanted kidney |
| 2209101000000119 | SP08Q00 | 853021000000108 | 2209101000000119 | Aneurysm of artery of transplanted kidney |
| 855641000006115 |  | 855641000006104 | 855641000006115 | Chronic kidney rejection |
| 2234541000000115 | SP08Z00 | 864271000000105 | 2234541000000115 | Thrombosis of artery of transplanted kidney |
| 2234621000000111 | SP08a00 | 864311000000105 | 2234621000000111 | Thrombosis of vein of transplanted kidney |
| 8321131000006112 |  | 905871000000102 | 2329241000000111 | Ultrasound guided insertion of nephrostomy tube into transplanted kidney |
| 8321121000006114 |  | 905871000000102 | 2329251000000114 | Ultrasonography guided insertion of nephrostomy tube into transplanted kidney |
| 8321141000006119 |  | 905881000000100 | 2329271000000117 | Fluoroscopy guided insertion of nephrostomy tube into transplanted kidney |
| 906351000006116 |  | 906351000006100 | 906351000006116 | [RFC] Renal transplant |
| 8335261000006114 |  | 927531000000100 | 2377621000000112 | Ultrasound scan of transplanted kidney with contrast |
| 8335251000006112 |  | 927531000000100 | 2377631000000114 | Ultrasonography of transplanted kidney with contrast |
| 8348921000006119 |  | 955621000000105 | 2437031000000111 | Fluoroscopy guided antegrade insertion of stent into ureter of transplanted kidney |
| 8348931000006116 |  | 955631000000107 | 2437051000000116 | Fluoroscopy guided retrograde insertion of stent into ureter of transplanted kidney |
| 8450921000006111 |  | 1076401000000104 | 2699151000000117 | Pretransplant evaluation of kidney and pancreas recipient |
| 2743241000000110 |  | 1095231000000108 | 2743241000000110 | Transplant of kidney using robotic assistance |
| 1713331000006114 |  | 1713331000006105 | 1713331000006114 | Transplantation surgery |

## **ICD10 codes used to identify kidney transplant in HES Admitted Patient Care data**

| icd | description |
| --- | --- |
| N16.5 | Renal tubulo-interstitial disorders in transplant rejection |
| T86.1 | Complications of kidney transplant |
| T86.10 | Unspecified complication of kidney transplant |
| T86.11 | Kidney transplant rejection |
| T86.12 | Kidney transplant failure |
| T86.13 | Kidney transplant infection |
| T86.19 | Other complication of kidney transplant |
| Z94.0 | Kidney transplant status |

## **OPCS codes used to identify kidney transplant in HES Admitted Patient Care data**

| opcs | term |
| --- | --- |
| M01 | Transplantation of kidney |
| M018 | Other specified transplantation of kidney |
| M019 | Unspecified transplantation of kidney |
| M012 | Allotransplantation of kidney from live donor |
| M013 | Allotransplantation of kidney from cadaver NEC |
| M014 | Allotransplantation of kidney from cadaver heart beating |
| M015 | Allotransplantation of kidney from cadaver heart non-beating |
| M084 | Exploration of transplanted kidney |
| M17 | Interventions associated with transplantation of kidney |
| M174 | Post-transplantation of kidney examination - recipient |
| M178 | Other specified interventions associated with transplantation of kidney |
| M179 | Unspecified interventions associated with transplantation of kidney |

## **Clinical codes used to identify chronic dialysis in CPRD Aurum**

| medcodeid | readcode | snomedctconceptid | snomedctdescriptionid | term |
| --- | --- | --- | --- | --- |
| 2509281000006118 |  | 676002 | 2198018 | Peritoneal dialysis including cannulation |
| 2550761000006119 |  | 3257008 | 6497016 | Empty and measure peritoneal dialysis fluid |
| 2672571000006112 |  | 10848006 | 18842012 | Removal of peritoneal dialysis catheter |
| 2672581000006110 |  | 10848006 | 18843019 | Removal of ambulatory peritoneal catheter |
| 2732451000006111 |  | 14684005 | 24946019 | Peritoneal dialysis excluding cannulation |
| 2751511000006115 |  | 15859004 | 26870017 | Chronic hemodialysis diet |
| 2751501000006118 |  | 15859004 | 477547013 | Chronic haemodialysis diet |
| 2938571000006111 |  | 27366005 | 45755014 | Megaloblastic anemia due to hemodialysis |
| 2938561000006116 |  | 27366005 | 483508018 | Megaloblastic anaemia due to haemodialysis |
| 3025081000006114 |  | 32599008 | 54422018 | Hemodialysis-associated amyloidosis |
| 3025091000006112 |  | 32599008 | 54423011 | beta-2-Microglobulin amyloidosis |
| 3025101000006118 |  | 32599008 | 54424017 | AH type amyloidosis |
| 3025111000006115 |  | 32599008 | 485266017 | Dialysis-associated amyloidosis |
| 485267014 | C373J00 | 32599008 | 485267014 | Beta-2 microglobulin amyloidosis |
| 3025071000006111 |  | 32599008 | 485268016 | Haemodialysis-associated amyloidosis |
| 1848741000006116 | SP0E.00 | 33461007 | 55841017 | Complication of peritoneal dialysis |
| 3062161000006115 |  | 34897002 | 58237014 | Hemodialysis, maintenance in hospital |
| 3062151000006117 |  | 34897002 | 485989017 | Haemodialysis, maintenance in hospital |
| 3135211000006116 |  | 39482009 | 66225019 | Removal of arteriovenous shunt for renal dialysis |
| 3503081000006117 |  | 61740001 | 102593013 | Insertion of cannula for hemodialysis, vein to vein |
| 3503071000006115 |  | 61740001 | 499107011 | Insertion of cannula for haemodialysis, vein to vein |
| 3531551000006110 |  | 63421002 | 105419019 | Insertion of cannula for hemodialysis |
| 3531541000006113 |  | 63421002 | 499590013 | Insertion of cannula for haemodialysis |
| 3605681000006118 |  | 67970008 | 112904010 | Hemodialysis, maintenance at home |
| 3605671000006116 |  | 67970008 | 500883012 | Haemodialysis, maintenance at home |
| 3612031000006118 |  | 68341005 | 113517012 | Hemodialysis, supervision at home |
| 3612021000006116 |  | 68341005 | 500994010 | Haemodialysis, supervision at home |
| 375841000000114 | 7L1A600 | 71192002 | 118218017 | Peritoneal dialysis NEC |
| 118218017 | 7L1A100 | 71192002 | 118218017 | Peritoneal dialysis |
| 3657891000006116 |  | 71192002 | 1233361017 | PD - Peritoneal dialysis |
| 3691341000006115 |  | 73257006 | 121658019 | Peritoneal dialysis catheter maintenance |
| 290737016 |  | 105502003 | 290737016 | Dependence on renal dialysis |
| 228131000006117 | 7L1B.11 | 180272001 | 278839019 | Placement ambulatory dialysis apparatus - compens renal fail |
| 773041000006111 | 7L1B000 | 180272001 | 278839019 | Insertion of chronic ambulatory peritoneal dialysis catheter |
| 4694051000006116 |  | 180273006 | 278840017 | Removal of chronic ambulatory peritoneal dialysis catheter |
| 181021000006119 | 7L1B100 | 180273006 | 278840017 | Removal of ambulatory peritoneal dialysis catheter |
| 278844014 | 7L1C000 | 180277007 | 278844014 | Insertion of temporary peritoneal dialysis catheter |
| 4937911000006117 |  | 225228006 | 338462016 | Putting additive into peritoneal dialysis bag |
| 4937921000006113 |  | 225229003 | 338463014 | Peritoneal dialysis bag procedure |
| 4937931000006111 |  | 225230008 | 338464015 | Chronic peritoneal dialysis |
| 4937941000006118 |  | 225230008 | 338465019 | PD - Chronic peritoneal dialysis |
| 4937961000006119 |  | 225231007 | 338466018 | PD - Stab peritoneal dialysis |
| 4937951000006116 |  | 225231007 | 338467010 | Stab peritoneal dialysis |
| 5094161000006119 |  | 236435004 | 354419017 | End stage renal failure on dialysis |
| 354591016 | SP0E000 | 236556004 | 354591016 | Bloodstained peritoneal dialysis effluent |
| 5095881000006119 |  | 236557008 | 354592011 | Peritoneal dialysis catheter exit site infection |
| 5095891000006116 |  | 236557008 | 354593018 | Peritoneal dialysis cannula exit site infection |
| 5095911000006119 |  | 236558003 | 354594012 | Peritoneal dialysis cannula tunnel infection |
| 5095901000006117 |  | 236558003 | 354595013 | Peritoneal dialysis catheter tunnel infection |
| 5095941000006115 |  | 236561002 | 354598010 | Disorder of peritoneal dialysis catheter |
| 5095951000006118 |  | 236561002 | 2551042019 | Disorder of peritoneal dialysis cannula |
| 5095971000006111 |  | 236562009 | 354600016 | Blockage of peritoneal dialysis catheter |
| 5095961000006116 |  | 236562009 | 354601017 | Obstruction of peritoneal dialysis catheter |
| 5095981000006114 |  | 236563004 | 354602012 | Migration of peritoneal dialysis catheter |
| 5095991000006112 |  | 236564005 | 354603019 | Omental wrapping around peritoneal dialysis catheter |
| 5096001000006113 |  | 236565006 | 354604013 | Poor drainage of peritoneal dialysis catheter |
| 5096011000006111 |  | 236566007 | 354605014 | Extrusion of peritoneal dialysis catheter cuff |
| 5096021000006115 |  | 236567003 | 354606010 | Misplacement of acute peritoneal dialysis catheter |
| 5120111000006110 |  | 238314006 | 357215018 | Renewal of chronic ambulatory peritoneal dialysis catheter |
| 5120121000006119 |  | 238315007 | 357216017 | Adjustment of chronic ambulatory peritoneal dialysis catheter |
| 5120131000006116 |  | 238316008 | 357217014 | Aspiration of chronic ambulatory peritoneal dialysis catheter |
| 5120141000006114 |  | 238317004 | 357218016 | Flushing of chronic ambulatory peritoneal dialysis catheter |
| 1705621000000113 | 7L1B200 | 238317004 | 1705621000000113 | Flushing of peritoneal dialysis catheter |
| 357219012 | 7L1A500 | 238318009 | 357219012 | Continuous ambulatory peritoneal dialysis |
| 5120171000006118 |  | 238318009 | 357220018 | CAPD - Continuous ambulatory peritoneal dialysis |
| 5120191000006117 |  | 238319001 | 357221019 | CCPD - Continuous cycling peritoneal dialysis |
| 5120181000006115 |  | 238319001 | 357222014 | Continuous cycling peritoneal dialysis |
| 5120211000006116 |  | 238321006 | 357225011 | Intermittent peritoneal dialysis |
| 5120221000006112 |  | 238321006 | 357226012 | IPD - Intermittent peritoneal dialysis |
| 5120241000006117 |  | 238322004 | 357227015 | PD - Peritoneal dialysis, tidal |
| 5120231000006110 |  | 238322004 | 357228013 | Tidal peritoneal dialysis |
| 5120251000006115 |  | 238323009 | 357229017 | Night-time intermittent peritoneal dialysis |
| 5120261000006118 |  | 238323009 | 357230010 | IPD - Intermittent peritoneal dialysis, night-time |
| 5120271000006113 |  | 238323009 | 357231014 | NIPD - Night-time intermittent peritoneal dialysis |
| 5307121000006117 |  | 251860000 | 3491847010 | Peritoneal dialysis observable |
| 5307221000006110 |  | 251867002 | 375195014 | Adequacy of peritoneal dialysis |
| 5307251000006118 |  | 251869004 | 375197018 | Adequacy of hemodialysis |
| 5307241000006115 |  | 251869004 | 375198011 | Adequacy of haemodialysis |
| 5524381000006119 |  | 271418008 | 406192014 | Chronic ambulatory peritoneal dialysis catheter procedure |
| 453312018 | SP05613 | 276883000 | 413231010 | Peritoneal dialysis-associated peritonitis |
| 5592051000006113 |  | 276883000 | 2871057010 | Peritonitis secondary to peritoneal dialysis |
| 5618941000006116 |  | 278904002 | 415953019 | Haemodialysis diet |
| 5618951000006119 |  | 278904002 | 415954013 | Hemodialysis diet |
| 5618961000006117 |  | 278905001 | 415955014 | CAPD diet |
| 5618971000006112 |  | 278905001 | 2620332015 | Continuous ambulatory peritoneal dialysis (CAPD) diet |
| 5618981000006110 |  | 278905001 | 2913474012 | Continuous ambulatory peritoneal dialysis diet |
| 6210341000006119 |  | 360132007 | 475787016 | Peritoneal dialysis catheter |
| 6210351000006117 |  | 360133002 | 475788014 | Chronic ambulatory peritoneal dialysis catheter |
| 6665621000006110 |  | 402458002 | 1773624015 | Haemodialysis-associated secondary amyloidosis of skin |
| 6665631000006113 |  | 402458002 | 1774910011 | Hemodialysis-associated secondary amyloidosis of skin |
| 6686181000006114 |  | 403732009 | 1773752015 | Haemodialysis-associated pseudoporphyria |
| 6686191000006112 |  | 403732009 | 1775038012 | Hemodialysis-associated pseudoporphyria |
| 6686201000006110 |  | 403732009 | 1783799019 | Pseudoporphyria in haemodialysis |
| 6686211000006113 |  | 403732009 | 1784185019 | Pseudoporphyria in hemodialysis |
| 6686221000006117 |  | 403733004 | 1773753013 | Haemodialysis-associated pruritus |
| 6686231000006119 |  | 403733004 | 1775039016 | Hemodialysis-associated pruritus |
| 6686241000006112 |  | 403733004 | 1783800015 | Pruritus due to haemodialysis |
| 6686251000006114 |  | 403733004 | 1784186018 | Pruritus due to hemodialysis |
| 6703951000006115 |  | 404813001 | 2156695011 | Peritoneal dialysis leakage |
| 6832691000006118 |  | 412863004 | 2474419012 | Dialysis fluid creatinine measurement |
| 6832701000006118 |  | 412863004 | 2478490017 | Dialysis fluid creatinine level |
| 7083761000006118 |  | 428118009 | 2694765010 | Procedure involving peritoneal dialysis catheter |
| 2692168016 | 7L1A400 | 428648006 | 2692168016 | Automated peritoneal dialysis |
| 7095851000006114 |  | 428937001 | 2692026019 | Dependence on peritoneal dialysis due to end stage renal disease |
| 7096531000006112 |  | 428982002 | 2693614019 | Dependence on hemodialysis due to end stage renal disease |
| 7096521000006114 |  | 428982002 | 2694168017 | Dependence on haemodialysis due to end stage renal disease |
| 7097941000006112 |  | 429075005 | 2694500017 | Dependence on dialysis due to end stage renal disease |
| 7130251000006116 |  | 431028002 | 2765424012 | Mechanical complication of peritoneal dialysis catheter |
| 7148101000006112 |  | 431873008 | 2770082015 | Peritonitis due to infected peritoneal dialysis catheter |
| 7220891000006111 |  | 439278006 | 2791992014 | Measurement of static venous pressure in haemodialysis vascular access |
| 7220901000006110 |  | 439278006 | 2795000016 | Measurement of static venous pressure in hemodialysis vascular access |
| 7224371000006114 |  | 439534001 | 2790106010 | Shaving of peritoneal dialysis catheter cuff |
| 7230311000006111 |  | 439976001 | 2790364015 | Measurement of recirculation in haemodialysis vascular access |
| 7230321000006115 |  | 439976001 | 2790365019 | Measurement of recirculation in hemodialysis vascular access |
| 7234171000006114 |  | 440228000 | 2793258014 | Leakage of peritoneal dialysate into subcutaneous tissue of abdominal wall |
| 7235291000006117 |  | 440310004 | 2792227017 | Leakage of peritoneal dialysate into pleural cavity |
| 7240511000006118 |  | 440663004 | 2790121016 | Encapsulating peritoneal sclerosis associated with peritoneal dialysis |
| 7240521000006114 |  | 440663004 | 2793694019 | Encapsulating peritoneal sclerosis (EPS) associated with peritoneal dialysis |
| 7262731000006112 |  | 442326005 | 2820289016 | Cloudy peritoneal dialysis effluent |
| 7267351000006115 |  | 442723000 | 2819796012 | Measurement of creatinine clearance normalized to body surface area in peritoneal dialysis fluid specimen |
| 7267341000006117 |  | 442723000 | 2819797015 | Measurement of creatinine clearance normalised to body surface area in peritoneal dialysis fluid specimen |
| 7279961000006115 |  | 443596009 | 2838885010 | Dependence on peritoneal dialysis |
| 7285721000006115 |  | 443946002 | 2841322011 | Quantitative measurement of mass concentration of glucose in peritoneal dialysis fluid specimen |
| 7285711000006111 |  | 443946002 | 2841806018 | Quantitative measurement of glucose in peritoneal dialysis fluid specimen |
| 7286221000006116 |  | 443978005 | 2840860017 | Quantitative measurement of mass concentration of creatinine in predialysis serum or plasma |
| 7286231000006118 |  | 443978005 | 2840861018 | Quantitative measurement of mass concentration of creatinine in predialysis serum or plasma specimen |
| 7286241000006111 |  | 443979002 | 2840862013 | Quantitative measurement of creatinine in postdialysis serum or plasma specimen |
| 7286251000006113 |  | 443979002 | 2842433011 | Quantitative measurement of mass concentration of creatinine in postdialysis serum or plasma specimen |
| 7286491000006115 |  | 443993009 | 2841166018 | Quantitative measurement of mass concentration of creatinine in 2 hour postperitoneal dialysis fluid specimen |
| 7286481000006118 |  | 443993009 | 2841820013 | Quantitative measurement of creatinine in 2 hour postperitoneal dialysis fluid specimen |
| 7286501000006111 |  | 443994003 | 2841167010 | Quantitative measurement of mass concentration of creatinine corrected for glucose concentration in overnight peritoneal dialysis fluid |
| 7286511000006114 |  | 443994003 | 2841168017 | Quantitative measurement of mass concentration of creatinine corrected for glucose concentration in overnight peritoneal dialysis fluid specimen |
| 7289841000006118 |  | 444224006 | 2841400015 | Quantitative measurement of creatinine in 4 hour postperitoneal dialysis fluid specimen |
| 7289851000006116 |  | 444224006 | 2841401016 | Quantitative measurement of mass concentration of creatinine in 4 hour postperitoneal dialysis fluid specimen |
| 7293001000006112 |  | 444423002 | 2841561016 | Quantitative measurement of glucose in predialysis peritoneal dialysis fluid specimen |
| 7293011000006110 |  | 444423002 | 2841562011 | Quantitative measurement of mass concentration of glucose in predialysis peritoneal dialysis fluid specimen |
| 7293281000006110 |  | 444450007 | 2841320015 | Quantitative measurement of concentration of glucose in peritoneal dialysis fluid at 4 hours postperitoneal dialysis |
| 7293291000006113 |  | 444450007 | 2841321016 | Quantitative measurement of mass concentration of glucose in peritoneal dialysis fluid specimen at 4 hours postperitoneal dialysis |
| 7293931000006117 |  | 444499006 | 2841333014 | Quantitative measurement of glucose in first peritoneal dialysis fluid specimen |
| 7293941000006110 |  | 444499006 | 2841334015 | Quantitative measurement of mass concentration of glucose in first peritoneal dialysis fluid specimen |
| 7300651000006115 |  | 444959000 | 2871077015 | Peritoneal dialysis fluid specimen obtained before dialysis |
| 7300661000006118 |  | 444959000 | 2871078013 | Predialysis peritoneal dialysis fluid specimen |
| 7302331000006118 |  | 445069004 | 2871530017 | Peritoneal dialysis fluid specimen obtained after dialysis |
| 7302341000006111 |  | 445069004 | 2871531018 | Postdialysis peritoneal dialysis fluid specimen |
| 7338641000006112 |  | 447355008 | 2883363011 | Dialysis fluid specimen obtained after continuous ambulatory peritoneal dialysis |
| 7344691000006110 |  | 447693007 | 2899341010 | Peritoneal dialysis technique |
| 7400641000006114 |  | 462231004 | 2937194015 | Haemodialysis fistula dressing |
| 7400651000006111 |  | 462231004 | 3033163010 | Hemodialysis fistula dressing |
| 7400821000006115 |  | 462251000 | 2941584010 | Haemodialysis chair, manual |
| 7400831000006117 |  | 462251000 | 3033084015 | Hemodialysis chair, manual |
| 7402641000006113 |  | 462465001 | 2947729010 | Haemodialysis chair, electric |
| 7402651000006110 |  | 462465001 | 3033183011 | Hemodialysis chair, electric |
| 7403941000006112 |  | 462617005 | 2945522016 | Haemodialysis needle |
| 7403951000006114 |  | 462617005 | 3031525019 | Hemodialysis needle |
| 7483511000006116 |  | 473147002 | 2955983012 | Malfunction of peritoneal dialysis catheter |
| 7484161000006113 |  | 473190001 | 2956113013 | Leakage of peritoneal dialysis catheter |
| 7518811000006118 |  | 699235009 | 2983033012 | CAPD (Continuous ambulatory peritoneal dialysis) associated peritonitis |
| 1743241000006119 | SP06B00 | 699235009 | 2983127018 | Continuous ambulatory peritoneal dialysis associated peritonitis |
| 7593681000006118 |  | 704667004 | 3013929017 | Hypertension concurrent and due to end stage renal disease on dialysis |
| 7612311000006115 |  | 707090004 | 3028437018 | Spondyloarthropathy due to hemodialysis-associated amyloidosis |
| 7641791000006111 |  | 708931003 | 3036621018 | Maintenance hemodialysis |
| 7641781000006113 |  | 708931003 | 3036926014 | Maintenance haemodialysis |
| 7708331000006119 |  | 713720009 | 3297449010 | Sclerosing peritonitis as complication of peritoneal dialysis |
| 7708341000006112 |  | 713720009 | 3297510014 | Sclerosing peritonitis due to peritoneal dialysis |
| 7708351000006114 |  | 713721008 | 3297485010 | Hydrothorax as complication of peritoneal dialysis |
| 7708361000006111 |  | 713721008 | 3297567017 | Hydrothorax due to peritoneal dialysis |
| 7708411000006115 |  | 713724000 | 3297498018 | Abdominal hernia due to peritoneal dialysis |
| 7708401000006118 |  | 713724000 | 3297500017 | Abdominal hernia as complication of peritoneal dialysis |
| 7708451000006119 |  | 713727007 | 3297543013 | Hemoperitoneum due to peritoneal dialysis |
| 7708461000006117 |  | 713727007 | 3297546017 | Hemoperitoneum as complication of peritoneal dialysis |
| 7708441000006116 |  | 713727007 | 3297633016 | Haemoperitoneum as complication of peritoneal dialysis |
| 7708471000006112 |  | 713727007 | 3297646014 | Haemoperitoneum due to peritoneal dialysis |
| 7714911000006116 |  | 714152005 | 3298670011 | CKD (chronic kidney disease) stage 5d |
| 7714891000006118 |  | 714152005 | 3298729015 | Chronic kidney disease stage 5 on dialysis |
| 7761641000006110 |  | 717738008 | 3310130013 | Peritoneal dialysis care assessment |
| 7770551000006113 |  | 718308002 | 3311852014 | Peritoneal dialysis care |
| 7960171000006111 |  | 733699001 | 3473270012 | Home-use peritoneal dialysis connection kit |
| 7960181000006114 |  | 733700000 | 3473274015 | Home-use peritoneal dialysis connection kit housing |
| 7960191000006112 |  | 733701001 | 3473276018 | Home-use peritoneal dialysis connection kit frame |
| 3524930011 |  | 736795005 | 3524930011 | Hemodialysis needle gauge |
| 3525029011 |  | 736795005 | 3525029011 | Haemodialysis needle gauge |
| 7965451000006115 |  | 381000124105 | 3037530013 | Increase dialyzer size for hemodialysis |
| 7965441000006117 |  | 381000124105 | 3083959017 | Increase dialyser size for haemodialysis |
| 7965521000006111 |  | 411000124108 | 3030694010 | Haemodialysis residual renal function |
| 7965531000006114 |  | 411000124108 | 3030697015 | Hemodialysis residual renal function |
| 7965641000006115 |  | 461000124106 | 3030679011 | Increase in haemodialysis dialyzer size |
| 7965651000006118 |  | 461000124106 | 3030695011 | Increase in hemodialysis dialyzer size |
| 7965711000006110 |  | 471000124104 | 3030690018 | Increase in hemodialysis session time |
| 7965701000006112 |  | 471000124104 | 3030704013 | Increase in haemodialysis session time |
| 7966231000006117 |  | 631000124104 | 3285793011 | Compliant with peritoneal dialysis prescription |
| 7966301000006117 |  | 641000124109 | 3285794017 | Non-compliant with peritoneal dialysis prescription |
| 7966361000006116 |  | 651000124106 | 3028849010 | Peritoneal dialysis prescription compliance |
| 8027511000006119 |  | 90771000119100 | 3014101011 | End stage renal disease on dialysis due to type 1 diabetes mellitus |
| 8027531000006113 |  | 90791000119104 | 3013806012 | End stage renal disease on dialysis due to type 2 diabetes mellitus |
| 8030391000006118 |  | 96701000119107 | 2985615019 | Hypertensive heart AND chronic kidney disease on dialysis |
| 8040331000006118 |  | 127991000119101 | 3013758011 | Hypertension concurrent and due to end stage renal disease on dialysis due to type 2 diabetes mellitus |
| 8040341000006111 |  | 128001000119105 | 3013944016 | Hypertension concurrent and due to end stage renal disease on dialysis due to type 1 diabetes mellitus |
| 8041741000006117 |  | 133031000119106 | 3041189016 | Thrombosis of arteriovenous graft due to hemodialysis arteriovenous access device |
| 8041731000006110 |  | 133031000119106 | 3041266015 | Thrombosis of arteriovenous graft due to haemodialysis arteriovenous access device |
| 8041751000006115 |  | 133031000119106 | 3292003015 | Thrombosis of arteriovenous graft caused by haemodialysis arteriovenous access device |
| 8041761000006118 |  | 133031000119106 | 3293436018 | Thrombosis of arteriovenous graft caused by hemodialysis arteriovenous access device |
| 8088131000006111 |  | 286371000119107 | 2922234012 | Malignant hypertensive end stage renal disease on dialysis |
| 8117011000006113 |  | 365001000000107 | 688631000000114 | Peritoneal dialysis associated encapsulating peritoneal sclerosis |
| 8117021000006117 |  | 365001000000107 | 1692451000000115 | Peritoneal dialysis associated EPS (encapsulating peritoneal sclerosis) |
| 2478488018 | 4N0..00 | 991511000000108 | 2570421000000112 | Dialysis fluid urea level |
| 2478492013 | 4N2..00 | 991521000000102 | 2558991000000119 | Dialysis fluid glucose level |
| 2478490017 | 4N1..00 | 1011911000000108 | 2552161000000112 | Dialysis fluid creatinine level |
| 9351511000006113 |  | 11000731000119102 | 3045266011 | Dependence on continuous ambulatory peritoneal dialysis |
| 9351521000006117 |  | 11000771000119104 | 3036677016 | Dependence on continuous cycling peritoneal dialysis |
| 9351531000006119 |  | 11000771000119104 | 3037224014 | Continuous cycling peritoneal dialysis dependence |
| 9351541000006112 |  | 11000771000119104 | 3037613015 | Dependence on continuous cyclic peritoneal dialysis |
| 9896121000006112 |  | 16218721000119107 | 3315040011 | Hyperparathyroidism due to end stage renal disease on dialysis |

## **ICD10 codes used to identify chronic dialysis in HES Admitted Patient Care data**

| icd | description |
| --- | --- |
| Z99.2 | Dependence on renal dialysis |

## **OPCS codes used to identify chronic dialysis in HES Admitted Patient Care data**

| opcs | term |
| --- | --- |
| X402 | Peritoneal dialysis NEC |
| X405 | Automated peritoneal dialysis |
| X406 | Continuous ambulatory peritoneal dialysis |
| X41 | Placement of ambulatory apparatus for compensation for renal failure |
| X411 | Insertion of ambulatory peritoneal dialysis catheter |

## **OPCS codes used to identify major surgery in HES Admitted Patient Care data**

| surgery_type | procedure | opcs |
| --- | --- | --- |
| Cardiac | Transplantation of heart or heart+lung | K01 |
| Cardiac | Transplantation of heart or heart+lung | K02 |
| Cardiac | Valve surgery | K25 |
| Cardiac | Valve surgery | K26 |
| Cardiac | Valve surgery | K27 |
| Cardiac | Valve surgery | K28 |
| Cardiac | Valve surgery | K29 |
| Cardiac | Valve surgery | K30 |
| Cardiac | Valve surgery | K31 |
| Cardiac | Valve surgery | K32 |
| Cardiac | Valve surgery | K34 |
| Cardiac | Valve surgery | K35 |
| Cardiac | Valve surgery | K36 |
| Cardiac | Coronary artery bypass | K40 |
| Cardiac | Coronary artery bypass | K41 |
| Cardiac | Coronary artery bypass | K42 |
| Cardiac | Coronary artery bypass | K43 |
| Cardiac | Coronary artery bypass | K44 |
| Cardiac | Coronary artery bypass | K45 |
| Cardiac | Coronary artery bypass | K46 |
| Cardiac | Operations for atrial fibrillation | K52 |
| Cardiac | Cardiopulmonary bypass | Y73 |
| Gastrointestinal | Adrenal gland surgery | B22 |
| Gastrointestinal | Adrenal gland surgery | B23 |
| Gastrointestinal | Resection of oesophagus | G0 |
| Gastrointestinal | Gastrectomy | G27 |
| Gastrointestinal | Gastrectomy | G28 |
| Gastrointestinal | Gastrectomy | G50 |
| Gastrointestinal | Excision of intestine, enterostomy | G60 |
| Gastrointestinal | Anastomosis of intestine | G72 |
| Gastrointestinal | Excision of intestine, enterostomy | G74 |
| Gastrointestinal | Excision of intestine, enterostomy | G75 |
| Gastrointestinal | Excision of intestine, enterostomy | H04 |
| Gastrointestinal | Excision of intestine, enterostomy | H05 |
| Gastrointestinal | Excision of intestine, enterostomy | H06 |
| Gastrointestinal | Excision of intestine, enterostomy | H07 |
| Gastrointestinal | Excision of intestine, enterostomy | H08 |
| Gastrointestinal | Excision of intestine, enterostomy | H09 |
| Gastrointestinal | Excision of intestine, enterostomy | H10 |
| Gastrointestinal | Excision of intestine, enterostomy | H11 |
| Gastrointestinal | Excision of intestine, enterostomy | H15 |
| Gastrointestinal | Excision of intestine, enterostomy | H29 |
| Gastrointestinal | Excision of intestine, enterostomy | H33 |
| Gastrointestinal | Excision of intestine, enterostomy | H34 |
| Gastrointestinal | Resection of liver | J02 |
| Gastrointestinal | Resection of liver | J03 |
| Gastrointestinal | Transduodenal operations on bile duct or ampulla of Vater | J27 |
| Gastrointestinal | Excision of pancreas | J55 |
| Gastrointestinal | Excision of pancreas | J56 |
| Gastrointestinal | Excision of pancreas | J57 |
| Gastrointestinal | Excision of pancreas | J58 |
| Gastrointestinal | Splenectomy | J69 |
| Gastrointestinal | Splenectomy | J70 |
| Gastrointestinal | Omentectomy | T36 |
| Orthopedic surgery | Knee arthroplasty | W40 |
| Orthopedic surgery | Knee arthroplasty | W41 |
| Orthopedic surgery | Knee arthroplasty | W42 |
| Orthopedic surgery | Hip arthroplasty | W93 |
| Orthopedic surgery | Hip arthroplasty | W94 |
| Orthopedic surgery | Hip arthroplasty | W95 |
| Orthopedic surgery | Amputation | X09 |
| Pulmonary | Transplantation of lung | E53 |
| Pulmonary | Resection of lung | E54 |
| Pulmonary | Resection of lung | E55 |
| Vascular surgery | Arterial surgical procedures on aorta and associated arteries, lower extremities aneurysm repair/bypass | L19 |
| Vascular surgery | Arterial surgical procedures on aorta and associated arteries, lower extremities aneurysm repair/bypass | L21 |
| Vascular surgery | Arterial surgical procedures on aorta and associated arteries, lower extremities aneurysm repair/bypass | L23 |
| Vascular surgery | Arterial surgical procedures on aorta and associated arteries, lower extremities aneurysm repair/bypass | L25 |
| Vascular surgery | Thrombendarterectomy | L25 |
| Vascular surgery | Insertion of stent into abdominal aorta, visceral arteries, and iliac arteries | L261 |
| Vascular surgery | Insertion of stent into abdominal aorta, visceral arteries, and iliac arteries | L262 |
| Vascular surgery | Insertion of stent into abdominal aorta, visceral arteries, and iliac arteries | L263 |
| Vascular surgery | Insertion of stent into abdominal aorta, visceral arteries, and iliac arteries | L265 |
| Vascular surgery | Insertion of stent into abdominal aorta, visceral arteries, and iliac arteries | L266 |
| Vascular surgery | Insertion of stent into abdominal aorta, visceral arteries, and iliac arteries | L267 |
| Vascular surgery | Insertion of stent into abdominal aorta, visceral arteries, and iliac arteries | L269 |
| Vascular surgery | Arterial surgical procedures on aorta and associated arteries, lower extremities aneurysm repair/bypass | L27 |
| Vascular surgery | Insertion of stent into abdominal aorta, visceral arteries, and iliac arteries | L27 |
| Vascular surgery | Arterial surgical procedures on aorta and associated arteries, lower extremities aneurysm repair/bypass | L28 |
| Vascular surgery | Insertion of stent into abdominal aorta, visceral arteries, and iliac arteries | L28 |
| Vascular surgery | Thrombendarterectomy | L45 |
| Vascular surgery | Insertion of stent into abdominal aorta, visceral arteries, and iliac arteries | L474 |
| Vascular surgery | Arterial surgical procedures on aorta and associated arteries, lower extremities aneurysm repair/bypass | L49 |
| Vascular surgery | Arterial surgical procedures on aorta and associated arteries, lower extremities aneurysm repair/bypass | L51 |
| Vascular surgery | Thrombendarterectomy | L52 |
| Vascular surgery | Arterial surgical procedures on aorta and associated arteries, lower extremities aneurysm repair/bypass | L53 |
| Vascular surgery | Insertion of stent into abdominal aorta, visceral arteries, and iliac arteries | L544 |
| Vascular surgery | Arterial surgical procedures on aorta and associated arteries, lower extremities aneurysm repair/bypass | L57 |
| Vascular surgery | Arterial surgical procedures on aorta and associated arteries, lower extremities aneurysm repair/bypass | L59 |
| Vascular surgery | Resection of vein | L79 |
| Vascular surgery | Resection of vein | L82 |
| Vascular surgery | Resection of vein | L84 |
| Vascular surgery | Resection of vein | L87 |
| Vascular surgery | Resection of vein | L88 |
| Vascular surgery | Resection of vein | L90 |
| Vascular surgery | Resection of vein | L93 |

## **Product codes used to identify RAASi prescribing in CPRD Aurum**

| prodcodeid | term | drugclass | Combined type |
| --- | --- | --- | --- |
| 13241000033114 | Accupro 10mg tablets | ACE inhibitors |  |
| 13341000033116 | Accupro 20mg tablets | ACE inhibitors |  |
| 11641000033111 | Accupro 40mg tablets | ACE inhibitors |  |
| 13441000033110 | Accupro 5mg tablets | ACE inhibitors |  |
| 10841000033119 | Acepril 12.5mg tablets | ACE inhibitors |  |
| 10941000033110 | Acepril 25mg tablets | ACE inhibitors |  |
| 11041000033117 | Acepril 50mg tablets | ACE inhibitors |  |
| 214141000033113 | Capoten 12.5mg tablets | ACE inhibitors |  |
| 214241000033118 | Capoten 25mg tablets | ACE inhibitors |  |
| 214341000033111 | Capoten 50mg tablets | ACE inhibitors |  |
| 5008141000033111 | Captopril 10mg/5ml oral suspension | ACE inhibitors |  |
| 213441000033119 | Captopril 12.5mg tablets | ACE inhibitors |  |
| 5898741000033114 | Captopril 12.5mg/5ml oral solution | ACE inhibitors |  |
| 5898841000033116 | Captopril 12.5mg/5ml oral suspension | ACE inhibitors |  |
| 5401641000033118 | Captopril 15mg/5ml oral suspension | ACE inhibitors |  |
| 2719841000033113 | Captopril 20mg/5ml oral suspension | ACE inhibitors |  |
| 214441000033117 | Captopril 25mg tablets | ACE inhibitors |  |
| 3012741000033115 | Captopril 25mg/5ml oral solution | ACE inhibitors |  |
| 9108041000033112 | Captopril 25mg/5ml oral solution sugar free | ACE inhibitors |  |
| 9108041000033111 | Captopril 25mg/5ml oral solution sugar free | ACE inhibitors |  |
| 5132041000033112 | Captopril 25mg/5ml oral suspension | ACE inhibitors |  |
| 3940841000033110 | Captopril 2mg capsules | ACE inhibitors |  |
| 3851341000033117 | Captopril 3mg/5ml oral solution | ACE inhibitors |  |
| 5454041000033117 | Captopril 4mg capsules | ACE inhibitors |  |
| 214541000033116 | Captopril 50mg tablets | ACE inhibitors |  |
| 6001041000033115 | Captopril 5mg/5ml oral solution | ACE inhibitors |  |
| 9108141000033110 | Captopril 5mg/5ml oral solution sugar free | ACE inhibitors |  |
| 2656341000033110 | Captopril 5mg/5ml oral suspension | ACE inhibitors |  |
| 5898941000033112 | Captopril 6.25mg/5ml oral solution | ACE inhibitors |  |
| 5899041000033115 | Captopril 6.25mg/5ml oral suspension | ACE inhibitors |  |
| 3333941000033117 | Captopril 8mg/5ml oral suspension | ACE inhibitors |  |
| 5595341000033117 | Captopril Oral Suspension Sugar Free 2 mg/ml | ACE inhibitors |  |
| 210541000033113 | Captopril Tablets 1 mg | ACE inhibitors |  |
| 210741000033117 | Captopril Tablets 2 mg | ACE inhibitors |  |
| 215941000033111 | Carace 10mg tablets | ACE inhibitors |  |
| 216041000033118 | Carace 2.5mg tablets | ACE inhibitors |  |
| 216141000033119 | Carace 20mg tablets | ACE inhibitors |  |
| 216241000033114 | Carace 5mg tablets | ACE inhibitors |  |
| 258141000033110 | Cilazapril 1mg tablets | ACE inhibitors |  |
| 258241000033115 | Cilazapril 2.5mg tablets | ACE inhibitors |  |
| 258441000033119 | Cilazapril 500microgram tablets | ACE inhibitors |  |
| 258641000033117 | Cilazapril 5mg tablets | ACE inhibitors |  |
| 258341000033113 | Cilazapril Tablets 250 micrograms | ACE inhibitors |  |
| 377541000033114 | Coversyl 2mg tablets | ACE inhibitors |  |
| 377641000033110 | Coversyl 4mg tablets | ACE inhibitors |  |
| 2846641000033111 | Coversyl 8mg tablets | ACE inhibitors |  |
| 4454441000033119 | Coversyl Arginine 10mg tablets | ACE inhibitors |  |
| 4454241000033115 | Coversyl Arginine 2.5mg tablets | ACE inhibitors |  |
| 4454341000033113 | Coversyl Arginine 5mg tablets | ACE inhibitors |  |
| 2945841000033112 | Ecopace 12.5mg tablets | ACE inhibitors |  |
| 2945941000033116 | Ecopace 25mg tablets | ACE inhibitors |  |
| 2946041000033114 | Ecopace 50mg tablets | ACE inhibitors |  |
| 1918441000033110 | Ednyt Tablets 10 mg | ACE inhibitors |  |
| 1918241000033114 | Ednyt Tablets 2.5 mg | ACE inhibitors |  |
| 1918541000033111 | Ednyt Tablets 20 mg | ACE inhibitors |  |
| 1918341000033116 | Ednyt Tablets 5 mg | ACE inhibitors |  |
| 6012441000033118 | Enalapril 1.25mg/5ml oral solution | ACE inhibitors |  |
| 6012541000033117 | Enalapril 1.25mg/5ml oral suspension | ACE inhibitors |  |
| 522641000033110 | Enalapril 10mg tablets | ACE inhibitors |  |
| 5967041000033117 | Enalapril 10mg/5ml oral solution | ACE inhibitors |  |
| 5967141000033118 | Enalapril 10mg/5ml oral suspension | ACE inhibitors |  |
| 523541000033115 | Enalapril 2.5mg tablets | ACE inhibitors |  |
| 522741000033118 | Enalapril 20mg tablets | ACE inhibitors |  |
| 522841000033111 | Enalapril 5mg tablets | ACE inhibitors |  |
| 5992541000033111 | Enalapril 5mg/5ml oral solution | ACE inhibitors |  |
| 5992641000033112 | Enalapril 5mg/5ml oral suspension | ACE inhibitors |  |
| 3279241000033117 | Enalapril 5mg/5ml oral suspension sugar free | ACE inhibitors |  |
| 609141000033111 | Fosinopril 10mg tablets | ACE inhibitors |  |
| 609241000033116 | Fosinopril 20mg tablets | ACE inhibitors |  |
| 648341000033112 | Gopten 1mg capsules | ACE inhibitors |  |
| 648441000033118 | Gopten 2mg capsules | ACE inhibitors |  |
| 3014841000033118 | Gopten 4mg capsules | ACE inhibitors |  |
| 648541000033117 | Gopten 500microgram capsules | ACE inhibitors |  |
| 1739741000033112 | Imidapril 10mg tablets | ACE inhibitors |  |
| 2145841000033114 | Imidapril 20mg tablets | ACE inhibitors |  |
| 1739841000033119 | Imidapril 5mg tablets | ACE inhibitors |  |
| 769041000033116 | Innovace 10mg tablets | ACE inhibitors |  |
| 769141000033117 | Innovace 2.5mg tablets | ACE inhibitors |  |
| 769241000033112 | Innovace 20mg tablets | ACE inhibitors |  |
| 769341000033119 | Innovace 5mg tablets | ACE inhibitors |  |
| 1622041000033110 | Kaplon 12.5mg tablets | ACE inhibitors |  |
| 1622141000033114 | Kaplon 25mg tablets | ACE inhibitors |  |
| 1622241000033119 | Kaplon 50mg tablets | ACE inhibitors |  |
| 837841000033110 | Lisinopril 10mg tablets | ACE inhibitors |  |
| 837941000033119 | Lisinopril 2.5mg tablets | ACE inhibitors |  |
| 5971341000033117 | Lisinopril 2.5mg/5ml oral solution | ACE inhibitors |  |
| 5971441000033111 | Lisinopril 2.5mg/5ml oral suspension | ACE inhibitors |  |
| 838041000033116 | Lisinopril 20mg tablets | ACE inhibitors |  |
| 6044641000033119 | Lisinopril 20mg/5ml oral solution | ACE inhibitors |  |
| 6044541000033115 | Lisinopril 20mg/5ml oral suspension | ACE inhibitors |  |
| 5568741000033113 | Lisinopril 40mg/5ml oral suspension | ACE inhibitors |  |
| 838141000033117 | Lisinopril 5mg tablets | ACE inhibitors |  |
| 5890241000033117 | Lisinopril 5mg/5ml oral solution | ACE inhibitors |  |
| 10645041000033114 | Lisinopril 5mg/5ml oral solution sugar free | ACE inhibitors |  |
| 3152141000033115 | Lisinopril 5mg/5ml oral suspension | ACE inhibitors |  |
| 5971541000033112 | Lisinopril 7.5mg/5ml oral solution | ACE inhibitors |  |
| 5971641000033113 | Lisinopril 7.5mg/5ml oral suspension | ACE inhibitors |  |
| 3159941000033114 | Lopace 10mg capsules | ACE inhibitors |  |
| 3159741000033111 | Lopace 2.5mg capsules | ACE inhibitors |  |
| 3159841000033118 | Lopace 5mg capsules | ACE inhibitors |  |
| 938041000033113 | Moexipril 15mg tablets | ACE inhibitors |  |
| 938141000033112 | Moexipril 7.5mg tablets | ACE inhibitors |  |
| 8961341000033119 | Noyada 25mg/5ml oral solution | ACE inhibitors |  |
| 8961241000033112 | Noyada 5mg/5ml oral solution | ACE inhibitors |  |
| 998541000033110 | Odrik 1mg capsules | ACE inhibitors |  |
| 998641000033111 | Odrik 2mg capsules | ACE inhibitors |  |
| 998741000033119 | Odrik 500microgram capsules | ACE inhibitors |  |
| 1065941000033115 | Perdix 15mg tablets | ACE inhibitors |  |
| 1066041000033113 | Perdix 7.5mg tablets | ACE inhibitors |  |
| 4454141000033110 | Perindopril arginine 10mg tablets | ACE inhibitors |  |
| 4453941000033114 | Perindopril arginine 2.5mg tablets | ACE inhibitors |  |
| 4454041000033111 | Perindopril arginine 5mg tablets | ACE inhibitors |  |
| 1067841000033115 | Perindopril erbumine 2mg tablets | ACE inhibitors |  |
| 1067941000033111 | Perindopril erbumine 4mg tablets | ACE inhibitors |  |
| 8869441000033119 | Perindopril erbumine 4mg/5ml oral solution | ACE inhibitors |  |
| 8276041000033117 | Perindopril erbumine 4mg/5ml oral suspension | ACE inhibitors |  |
| 2846741000033119 | Perindopril erbumine 8mg tablets | ACE inhibitors |  |
| 8275941000033110 | Perindopril erbumine 8mg/5ml oral solution | ACE inhibitors |  |
| 8276141000033118 | Perindopril erbumine 8mg/5ml oral suspension | ACE inhibitors |  |
| 5997541000033113 | Perindopril Oral solution 8 mg/5 ml | ACE inhibitors |  |
| 5888241000033116 | Perindopril Oral suspension 4 mg/5 ml | ACE inhibitors |  |
| 4153041000033114 | Perindopril Oral suspension 8 mg/5 ml | ACE inhibitors |  |
| 8263541000033111 | Perindopril tosilate 10mg tablets | ACE inhibitors |  |
| 8263341000033116 | Perindopril tosilate 2.5mg tablets | ACE inhibitors |  |
| 8263441000033110 | Perindopril tosilate 5mg tablets | ACE inhibitors |  |
| 1918841000033113 | Pralenal 10 tablets | ACE inhibitors |  |
| 1918641000033112 | Pralenal 2.5 tablets | ACE inhibitors |  |
| 1918941000033117 | Pralenal 20 tablets | ACE inhibitors |  |
| 1918741000033115 | Pralenal 5 tablets | ACE inhibitors |  |
| 1149341000033118 | Quinapril 10mg tablets | ACE inhibitors |  |
| 1149441000033112 | Quinapril 20mg tablets | ACE inhibitors |  |
| 1149541000033113 | Quinapril 40mg tablets | ACE inhibitors |  |
| 1149241000033111 | Quinapril 5mg tablets | ACE inhibitors |  |
| 3283241000033113 | Quinil 10mg tablets | ACE inhibitors |  |
| 3283341000033115 | Quinil 20mg tablets | ACE inhibitors |  |
| 3283441000033114 | Quinil 40mg tablets | ACE inhibitors |  |
| 3283141000033118 | Quinil 5mg tablets | ACE inhibitors |  |
| 1151341000033117 | Ramipril 1.25mg capsules | ACE inhibitors |  |
| 2989541000033115 | Ramipril 1.25mg tablets | ACE inhibitors |  |
| 5998241000033112 | Ramipril 1.25mg/5ml oral solution | ACE inhibitors |  |
| 4152241000033113 | Ramipril 1.25mg/5ml oral suspension | ACE inhibitors |  |
| 1769841000033111 | Ramipril 10mg capsules | ACE inhibitors |  |
| 2989841000033118 | Ramipril 10mg tablets | ACE inhibitors |  |
| 5887841000033118 | Ramipril 10mg/5ml oral solution | ACE inhibitors |  |
| 5887941000033114 | Ramipril 10mg/5ml oral suspension | ACE inhibitors |  |
| 1151441000033111 | Ramipril 2.5mg capsules | ACE inhibitors |  |
| 2989641000033119 | Ramipril 2.5mg tablets | ACE inhibitors |  |
| 5890541000033115 | Ramipril 2.5mg/5ml oral solution | ACE inhibitors |  |
| 6517041000033110 | Ramipril 2.5mg/5ml oral solution sugar free | ACE inhibitors |  |
| 4805941000033114 | Ramipril 2.5mg/5ml oral suspension | ACE inhibitors |  |
| 1151541000033112 | Ramipril 5mg capsules | ACE inhibitors |  |
| 2989741000033111 | Ramipril 5mg tablets | ACE inhibitors |  |
| 5890341000033110 | Ramipril 5mg/5ml oral solution | ACE inhibitors |  |
| 2883341000033118 | Ramipril 5mg/5ml oral suspension | ACE inhibitors |  |
| 3890041000033117 | Ramipril Powder | ACE inhibitors |  |
| 2724541000033115 | Ramipril Titration pack | ACE inhibitors |  |
| 1387341000033114 | Staril 10mg tablets | ACE inhibitors |  |
| 1387441000033115 | Staril 20mg tablets | ACE inhibitors |  |
| 1753841000033118 | Tanatril 10mg tablets | ACE inhibitors |  |
| 2145741000033116 | Tanatril 20mg tablets | ACE inhibitors |  |
| 1753941000033114 | Tanatril 5mg tablets | ACE inhibitors |  |
| 2928341000033114 | Tensopril 12.5mg tablets | ACE inhibitors |  |
| 2928441000033115 | Tensopril 25mg tablets | ACE inhibitors |  |
| 2928541000033119 | Tensopril 50mg tablets | ACE inhibitors |  |
| 1453941000033115 | Trandolapril 1mg capsules | ACE inhibitors |  |
| 1454041000033118 | Trandolapril 2mg capsules | ACE inhibitors |  |
| 3014741000033111 | Trandolapril 4mg capsules | ACE inhibitors |  |
| 1454141000033119 | Trandolapril 500microgram capsules | ACE inhibitors |  |
| 1455541000033110 | Tritace 1.25mg capsules | ACE inhibitors |  |
| 2989941000033114 | Tritace 1.25mg tablets | ACE inhibitors |  |
| 1769941000033115 | Tritace 10mg capsules | ACE inhibitors |  |
| 2990241000033112 | Tritace 10mg tablets | ACE inhibitors |  |
| 1455641000033111 | Tritace 2.5mg capsules | ACE inhibitors |  |
| 2990041000033116 | Tritace 2.5mg tablets | ACE inhibitors |  |
| 1455741000033119 | Tritace 5mg capsules | ACE inhibitors |  |
| 2990141000033117 | Tritace 5mg tablets | ACE inhibitors |  |
| 4521441000033112 | Tritace Tablet Titration Pack | ACE inhibitors |  |
| 2724641000033119 | Tritace titration pack capsules | ACE inhibitors |  |
| 12090641000033120 | Tritace titration pack tablets | ACE inhibitors |  |
| 12090641000033119 | Tritace titration pack tablets | ACE inhibitors |  |
| 1504641000033112 | Vascace 1mg tablets | ACE inhibitors |  |
| 1504741000033115 | Vascace 2.5mg tablets | ACE inhibitors |  |
| 1504941000033117 | Vascace 500microgram tablets | ACE inhibitors |  |
| 1503441000033113 | Vascace 5mg tablets | ACE inhibitors |  |
| 1504841000033113 | Vascace Tablets 250 micrograms | ACE inhibitors |  |
| 1551941000033114 | Zestril 10mg tablets | ACE inhibitors |  |
| 1552041000033115 | Zestril 2.5mg tablets | ACE inhibitors |  |
| 1552141000033116 | Zestril 20mg tablets | ACE inhibitors |  |
| 1552241000033111 | Zestril 5mg tablets | ACE inhibitors |  |
| 59141000033113 | Amias 16mg tablets | Angiotensin receptor blockers |  |
| 59241000033118 | Amias 2mg tablets | Angiotensin receptor blockers |  |
| 3227341000033118 | Amias 32mg tablets | Angiotensin receptor blockers |  |
| 59341000033111 | Amias 4mg tablets | Angiotensin receptor blockers |  |
| 59441000033117 | Amias 8mg tablets | Angiotensin receptor blockers |  |
| 75941000033114 | Aprovel 150mg tablets | Angiotensin receptor blockers |  |
| 76041000033116 | Aprovel 300mg tablets | Angiotensin receptor blockers |  |
| 76141000033117 | Aprovel 75mg tablets | Angiotensin receptor blockers |  |
| 7754841000033119 | Azilsartan medoxomil 20mg tablets | Angiotensin receptor blockers |  |
| 7754941000033110 | Azilsartan medoxomil 40mg tablets | Angiotensin receptor blockers |  |
| 7755041000033110 | Azilsartan medoxomil 80mg tablets | Angiotensin receptor blockers |  |
| 211541000033119 | Candesartan 16mg tablets | Angiotensin receptor blockers |  |
| 211641000033118 | Candesartan 2mg tablets | Angiotensin receptor blockers |  |
| 3227241000033111 | Candesartan 32mg tablets | Angiotensin receptor blockers |  |
| 211741000033110 | Candesartan 4mg tablets | Angiotensin receptor blockers |  |
| 211841000033117 | Candesartan 8mg tablets | Angiotensin receptor blockers |  |
| 2720241000033110 | Cozaar 100mg tablets | Angiotensin receptor blockers |  |
| 4957641000033110 | Cozaar 12.5mg tablets | Angiotensin receptor blockers |  |
| 5150041000033110 | Cozaar 2.5mg/ml oral suspension | Angiotensin receptor blockers |  |
| 370441000033112 | Cozaar 25mg tablets | Angiotensin receptor blockers |  |
| 370541000033113 | Cozaar 50mg tablets | Angiotensin receptor blockers |  |
| 437641000033118 | Diovan 160mg capsules | Angiotensin receptor blockers |  |
| 4425041000033113 | Diovan 320mg tablets | Angiotensin receptor blockers |  |
| 6528541000033114 | Diovan 3mg/1ml oral solution | Angiotensin receptor blockers |  |
| 437741000033110 | Diovan 40mg capsules | Angiotensin receptor blockers |  |
| 3201441000033114 | Diovan 40mg tablets | Angiotensin receptor blockers |  |
| 437841000033117 | Diovan 80mg capsules | Angiotensin receptor blockers |  |
| 7755141000033114 | Edarbi 20mg tablets | Angiotensin receptor blockers |  |
| 7755241000033119 | Edarbi 40mg tablets | Angiotensin receptor blockers |  |
| 7755341000033112 | Edarbi 80mg tablets | Angiotensin receptor blockers |  |
| 10943941000033112 | Entresto 24mg/26mg tablets | Angiotensin receptor blockers |  |
| 10944041000033114 | Entresto 49mg/51mg tablets | Angiotensin receptor blockers |  |
| 10944141000033112 | Entresto 97mg/103mg tablets | Angiotensin receptor blockers |  |
| 10944141000033113 | Entresto 97mg/103mg tablets | Angiotensin receptor blockers |  |
| 2036341000033115 | Eprosartan 300mg tablets | Angiotensin receptor blockers |  |
| 2036441000033114 | Eprosartan 400mg tablets | Angiotensin receptor blockers |  |
| 2036541000033110 | Eprosartan 600mg tablets | Angiotensin receptor blockers |  |
| 8554241000033111 | Ifirmasta 150mg tablets | Angiotensin receptor blockers |  |
| 8554341000033118 | Ifirmasta 300mg tablets | Angiotensin receptor blockers |  |
| 8554141000033116 | Ifirmasta 75mg tablets | Angiotensin receptor blockers |  |
| 775441000033117 | Irbesartan 150mg tablets | Angiotensin receptor blockers |  |
| 5970641000033117 | Irbesartan 150mg/5ml oral suspension | Angiotensin receptor blockers |  |
| 6167841000033112 | Irbesartan 20mg oral powder sachets | Angiotensin receptor blockers |  |
| 775541000033116 | Irbesartan 300mg tablets | Angiotensin receptor blockers |  |
| 4954341000033111 | Irbesartan 300mg/5ml oral suspension | Angiotensin receptor blockers |  |
| 6423941000033116 | Irbesartan 30mg oral powder sachets | Angiotensin receptor blockers |  |
| 6435741000033117 | Irbesartan 37.5mg oral powder sachets | Angiotensin receptor blockers |  |
| 775641000033115 | Irbesartan 75mg tablets | Angiotensin receptor blockers |  |
| 2720141000033115 | Losartan 100mg tablets | Angiotensin receptor blockers |  |
| 5971741000033116 | Losartan 100mg/5ml oral solution | Angiotensin receptor blockers |  |
| 5971841000033114 | Losartan 100mg/5ml oral suspension | Angiotensin receptor blockers |  |
| 4957541000033114 | Losartan 12.5mg tablets | Angiotensin receptor blockers |  |
| 5149941000033118 | Losartan 2.5mg/ml oral suspension sugar free | Angiotensin receptor blockers |  |
| 851841000033117 | Losartan 25mg tablets | Angiotensin receptor blockers |  |
| 851941000033113 | Losartan 50mg tablets | Angiotensin receptor blockers |  |
| 5971941000033118 | Losartan 50mg/5ml oral solution | Angiotensin receptor blockers |  |
| 5972041000033112 | Losartan 50mg/5ml oral suspension | Angiotensin receptor blockers |  |
| 2272741000033111 | Micardis 20mg tablets | Angiotensin receptor blockers |  |
| 1849741000033114 | Micardis 40mg tablets | Angiotensin receptor blockers |  |
| 1849841000033116 | Micardis 80mg tablets | Angiotensin receptor blockers |  |
| 2944541000033111 | Olmesartan medoxomil 10mg tablets | Angiotensin receptor blockers |  |
| 4273241000033118 | Olmesartan medoxomil 10mg/5ml oral suspension | Angiotensin receptor blockers |  |
| 2944641000033112 | Olmesartan medoxomil 20mg tablets | Angiotensin receptor blockers |  |
| 2944741000033115 | Olmesartan medoxomil 40mg tablets | Angiotensin receptor blockers |  |
| 2944841000033113 | Olmetec 10mg tablets | Angiotensin receptor blockers |  |
| 2944941000033117 | Olmetec 20mg tablets | Angiotensin receptor blockers |  |
| 2945041000033117 | Olmetec 40mg tablets | Angiotensin receptor blockers |  |
| 8194041000033119 | Sabervel 150mg tablets | Angiotensin receptor blockers |  |
| 8194141000033115 | Sabervel 300mg tablets | Angiotensin receptor blockers |  |
| 8193941000033116 | Sabervel 75mg tablets | Angiotensin receptor blockers |  |
| 10943641000033117 | Sacubitril 24mg / Valsartan 26mg tablets | Angiotensin receptor blockers |  |
| 10943641000033116 | Sacubitril 24mg / Valsartan 26mg tablets | Angiotensin receptor blockers |  |
| 10943741000033114 | Sacubitril 49mg / Valsartan 51mg tablets | Angiotensin receptor blockers |  |
| 10943841000033116 | Sacubitril 97mg / Valsartan 103mg tablets | Angiotensin receptor blockers |  |
| 2272641000033119 | Telmisartan 20mg tablets | Angiotensin receptor blockers |  |
| 1849541000033118 | Telmisartan 40mg tablets | Angiotensin receptor blockers |  |
| 1849641000033117 | Telmisartan 80mg tablets | Angiotensin receptor blockers |  |
| 2040841000033113 | Teveten 300mg tablets | Angiotensin receptor blockers |  |
| 2040941000033117 | Teveten 400mg tablets | Angiotensin receptor blockers |  |
| 2041041000033110 | Teveten 600mg tablets | Angiotensin receptor blockers |  |
| 8030141000033111 | Teveten Plus Tablets 600 mg/12.5 mg | Angiotensin receptor blockers |  |
| 9209341000033116 | Tolura 20mg tablets | Angiotensin receptor blockers |  |
| 9209441000033110 | Tolura 40mg tablets | Angiotensin receptor blockers |  |
| 9209541000033112 | Tolura 80mg tablets | Angiotensin receptor blockers |  |
| 9209541000033111 | Tolura 80mg tablets | Angiotensin receptor blockers |  |
| 1498241000033111 | Valsartan 160mg capsules | Angiotensin receptor blockers |  |
| 6515541000033114 | Valsartan 160mg tablets | Angiotensin receptor blockers |  |
| 4424941000033113 | Valsartan 320mg tablets | Angiotensin receptor blockers |  |
| 6528441000033113 | Valsartan 3mg/ml oral solution | Angiotensin receptor blockers |  |
| 1498341000033118 | Valsartan 40mg capsules | Angiotensin receptor blockers |  |
| 3201341000033115 | Valsartan 40mg tablets | Angiotensin receptor blockers |  |
| 1498441000033112 | Valsartan 80mg capsules | Angiotensin receptor blockers |  |
| 6515441000033113 | Valsartan 80mg tablets | Angiotensin receptor blockers |  |
| 11241000033113 | Accuretic 10mg/12.5mg tablets | Combined | ACE inhibitor / Thiazide diuretic |
| 13141000033119 | Acezide 25mg/50mg tablets | Combined | ACE inhibitor / Thiazide diuretic |
| 9159341000033112 | Actelsar HCT 40mg/12.5mg tablets | Combined | Angiotensin receptor blocker / Thiazide diuretic |
| 9159441000033118 | Actelsar HCT 80mg/12.5mg tablets | Combined | Angiotensin receptor blocker / Thiazide diuretic |
| 9159541000033117 | Actelsar HCT 80mg/25mg tablets | Combined | Angiotensin receptor blocker/ Thiazide diuretic |
| 9159541000033116 | Actelsar HCT 80mg/25mg tablets | Combined | Angiotensin receptor blocker / Thiazide diuretic |
| 4021641000033111 | Amlodipine 10mg / Valsartan 160mg tablets | Combined | Angiotensin receptor blocker / Calcium channel blocker |
| 4021541000033110 | Amlodipine 5mg / Valsartan 160mg tablets | Combined | Angiotensin receptor blocker / Calcium channel blocker |
| 4021441000033114 | Amlodipine 5mg / Valsartan 80mg tablets | Combined | Angiotensin receptor blocker / Calcium channel blocker |
| 216441000033110 | Capozide 25mg/50mg tablets | Combined | ACE inhibitor / Thiazide diuretic |
| 209241000033119 | Capozide LS 12.5mg/25mg tablets | Combined | ACE inhibitor / Thiazide diuretic |
| 1893341000033116 | Capto-Co Tablets 12.5 mg/25 mg | Combined | ACE inhibitor / Thiazide diuretic |
| 1893441000033110 | Capto-Co Tablets 25 mg/50 mg | Combined | ACE inhibitor / Thiazide diuretic |
| 209841000033115 | Carace 10 Plus tablets | Combined | ACE inhibitor / Thiazide diuretic |
| 217041000033116 | Carace 20 Plus tablets | Combined | ACE inhibitor / Thiazide diuretic |
| 3136041000033117 | Caralpha 10mg/12.5mg tablets | Combined | ACE inhibitor / Thiazide diuretic |
| 3136141000033118 | Caralpha 20mg/12.5mg tablets | Combined | ACE inhibitor / Thiazide diuretic |
| 2216141000033115 | CoAprovel 150mg/12.5mg tablets | Combined | Angiotensin receptor blocker / Thiazide diuretic |
| 2216241000033110 | CoAprovel 300mg/12.5mg tablets | Combined | Angiotensin receptor blocker / Thiazide diuretic |
| 3995341000033119 | CoAprovel 300mg/25mg tablets | Combined | Angiotensin receptor blocker / Thiazide diuretic |
| 3152541000033112 | Co-Diovan 160mg/12.5mg tablets | Combined | Angiotensin receptor blocker / Thiazide diuretic |
| 3152641000033113 | Co-Diovan 160mg/25mg tablets | Combined | Angiotensin receptor blocker / Thiazide diuretic |
| 3190841000033119 | Co-Diovan 80mg/12.5mg tablets | Combined | Angiotensin receptor blocker / Thiazide diuretic |
| 6028541000033112 | Co-Tritace Tablets 10 mg + 12.5 mg | Combined | ACE inhibitor / Thiazide diuretic |
| 6028641000033113 | Co-Tritace Tablets 10 mg + 25 mg | Combined | ACE inhibitor / Thiazide diuretic |
| 4454641000033117 | Coversyl Arginine Plus 5mg/1.25mg tablets | Combined | ACE inhibitor / Thiazide diuretic |
| 2739541000033117 | Coversyl Plus tablets | Combined | ACE inhibitor / Thiazide diuretic |
| 4424841000033117 | Cozaar-Comp 100mg/12.5mg tablets | Combined | Angiotensin receptor blocker / Thiazide diuretic |
| 3346941000033113 | Cozaar-Comp 100mg/25mg tablets | Combined | Angiotensin receptor blocker / Thiazide diuretic |
| 370841000033110 | Cozaar-Comp 50mg/12.5mg tablets | Combined | Angiotensin receptor blocker / Thiazide diuretic |
| 1893141000033119 | Co-zidocapt 12.5mg/25mg tablets | Combined | ACE inhibitor / Thiazide diuretic |
| 1893241000033114 | Co-zidocapt 25mg/50mg tablets | Combined | ACE inhibitor / Thiazide diuretic |
| 3162241000033117 | Enalapril 20mg / Hydrochlorothiazide 12.5mg tablets | Combined | ACE inhibitor / Thiazide diuretic |
| 4021941000033116 | Exforge 10mg/160mg tablets | Combined | Angiotensin receptor blocker / Calcium channel blocker |
| 4021841000033112 | Exforge 5mg/160mg tablets | Combined | Angiotensin receptor blocker / Calcium channel blocker |
| 4021741000033119 | Exforge 5mg/80mg tablets | Combined | Angiotensin receptor blocker / Calcium channel blocker |
| 8029241000033113 | Exforge Hct Tablets 5 mg/160 mg/12.5 mg | Combined | Angiotensin receptor blocker / Calcium channel blocker |
| 8029341000033115 | Exforge Hct Tablets 5 mg/160 mg/25 mg | Combined | Angiotensin receptor blocker / Calcium channel blocker |
| 8027941000033110 | Exforge Tablets 10 mg/320 mg | Combined | Angiotensin receptor blocker / Calcium channel blocker |
| 8027841000033119 | Exforge Tablets 5 mg/320 mg | Combined | Angiotensin receptor blocker / Calcium channel blocker |
| 3163341000033119 | Felodipine 2.5mg modified-release / Ramipril 2.5mg tablets | Combined | ACE inhibitor / Calcium channel blocker |
| 3163241000033112 | Felodipine 5mg modified-release / Ramipril 5mg tablets | Combined | ACE inhibitor / Calcium channel blocker |
| 770141000033115 | Innozide 20mg/12.5mg tablets | Combined | ACE inhibitor / Thiazide diuretic |
| 3161141000033116 | Irbesartan 150mg / Hydrochlorothiazide 12.5mg tablets | Combined | Angiotensin receptor blocker / Thiazide diuretic |
| 3161241000033111 | Irbesartan 300mg / Hydrochlorothiazide 12.5mg tablets | Combined | Angiotensin receptor blocker / Thiazide diuretic |
| 3995241000033112 | Irbesartan 300mg / Hydrochlorothiazide 25mg tablets | Combined | Angiotensin receptor blocker / Thiazide diuretic |
| 3279541000033115 | Lisicostad 10mg/12.5mg tablets | Combined | ACE inhibitor / Thiazide diuretic |
| 3279641000033119 | Lisicostad 20mg/12.5mg tablets | Combined | ACE inhibitor / Thiazide diuretic |
| 3160841000033117 | Lisinopril 10mg / Hydrochlorothiazide 12.5mg tablets | Combined | ACE inhibitor / Thiazide diuretic |
| 3160941000033113 | Lisinopril 20mg / Hydrochlorothiazide 12.5mg tablets | Combined | ACE inhibitor / Thiazide diuretic |
| 4424741000033110 | Losartan 100mg / Hydrochlorothiazide 12.5mg tablets | Combined | Angiotensin receptor blocker / Thiazide diuretic |
| 3346841000033117 | Losartan 100mg / Hydrochlorothiazide 25mg tablets | Combined | Angiotensin receptor blocker / Thiazide diuretic |
| 3161741000033117 | Losartan 50mg / Hydrochlorothiazide 12.5mg tablets | Combined | Angiotensin receptor blocker / Thiazide diuretic |
| 2864241000033110 | MicardisPlus 40mg/12.5mg tablets | Combined | Angiotensin receptor blocker / Thiazide diuretic |
| 2864341000033117 | MicardisPlus 80mg/12.5mg tablets | Combined | Angiotensin receptor blocker / Thiazide diuretic |
| 4548841000033118 | MicardisPlus 80mg/25mg tablets | Combined | Angiotensin receptor blocker / Thiazide diuretic |
| 5005841000033114 | Olmesartan medoxomil 20mg / Amlodipine 5mg tablets | Combined | Angiotensin receptor blocker / Calcium channel blocker |
| 3908941000033112 | Olmesartan medoxomil 20mg / Hydrochlorothiazide 12.5mg tablets | Combined | Angiotensin receptor blocker / Thiazide diuretic |
| 3909041000033115 | Olmesartan medoxomil 20mg / Hydrochlorothiazide 25mg tablets | Combined | Angiotensin receptor blocker / Thiazide diuretic |
| 5006041000033111 | Olmesartan medoxomil 40mg / Amlodipine 10mg tablets | Combined | Angiotensin receptor blocker / Calcium channel blocker |
| 5005941000033118 | Olmesartan medoxomil 40mg / Amlodipine 5mg tablets | Combined | Angiotensin receptor blocker / Calcium channel blocker |
| 5566341000033111 | Olmesartan medoxomil 40mg / Hydrochlorothiazide 12.5mg tablets | Combined | Angiotensin receptor blocker / Thiazide diuretic |
| 3909141000033116 | Olmetec Plus 20mg/12.5mg tablets | Combined | Angiotensin receptor blocker / Thiazide diuretic |
| 3909241000033111 | Olmetec Plus 20mg/25mg tablets | Combined | Angiotensin receptor blocker / Thiazide diuretic |
| 5566441000033117 | Olmetec Plus 40mg/12.5mg tablets | Combined | Angiotensin receptor blocker / Thiazide diuretic |
| 4454541000033118 | Perindopril arginine 5mg / Indapamide 1.25mg tablets | Combined | ACE inhibitor / Thiazide diuretic |
| 9105341000033116 | Perindopril erbumine 4mg / Amlodipine 10mg tablets | Combined | ACE inhibitor / Calcium channel blocker |
| 9105241000033114 | Perindopril erbumine 4mg / Amlodipine 5mg tablets | Combined | ACE inhibitor / Calcium channel blocker |
| 3161641000033114 | Perindopril erbumine 4mg / Indapamide 1.25mg tablets | Combined | ACE inhibitor / Thiazide diuretic |
| 9105541000033112 | Perindopril erbumine 8mg / Amlodipine 10mg tablets | Combined | ACE inhibitor / Calcium channel blocker |
| 9105541000033111 | Perindopril erbumine 8mg / Amlodipine 10mg tablets | Combined | ACE inhibitor / Calcium channel blocker |
| 9105441000033110 | Perindopril erbumine 8mg / Amlodipine 5mg tablets | Combined | ACE inhibitor / Calcium channel blocker |
| 8263641000033112 | Perindopril tosilate 5mg / Indapamide 1.25mg tablets | Combined | ACE inhibitor / Thiazide diuretic |
| 3160641000033118 | Quinapril 10mg / Hydrochlorothiazide 12.5mg tablets | Combined | ACE inhibitor / Thiazide diuretic |
| 5006141000033110 | Sevikar 20mg/5mg tablets | Combined | Angiotensin receptor blocker / Calcium channel blocker |
| 5006341000033113 | Sevikar 40mg/10mg tablets | Combined | Angiotensin receptor blocker / Calcium channel blocker |
| 5006241000033115 | Sevikar 40mg/5mg tablets | Combined | Angiotensin receptor blocker / Calcium channel blocker |
| 6180641000033112 | Sevikar HCT 20mg/5mg/12.5mg tablets | Combined | Angiotensin receptor blocker / Calcium channel blocker / Thiazide diuretic |
| 6180841000033113 | Sevikar HCT 40mg/10mg/12.5mg tablets | Combined | Angiotensin receptor blocker / Calcium channel blocker / Thiazide diuretic |
| 6181041000033110 | Sevikar HCT 40mg/10mg/25mg tablets | Combined | Angiotensin receptor blocker / Calcium channel blocker / Thiazide diuretic |
| 6180741000033115 | Sevikar HCT 40mg/5mg/12.5mg tablets | Combined | Angiotensin receptor blocker / Calcium channel blocker / Thiazide diuretic |
| 6180941000033117 | Sevikar HCT 40mg/5mg/25mg tablets | Combined | Angiotensin receptor blocker / Calcium channel blocker / Thiazide diuretic |
| 1406341000033117 | Tarka modified-release capsules | Combined | ACE inhibitor / Calcium channel blocker |
| 3162641000033119 | Telmisartan 40mg / Hydrochlorothiazide 12.5mg tablets | Combined | Angiotensin receptor blocker / Thiazide diuretic |
| 3162741000033111 | Telmisartan 80mg / Hydrochlorothiazide 12.5mg tablets | Combined | Angiotensin receptor blocker / Thiazide diuretic |
| 4548741000033111 | Telmisartan 80mg / Hydrochlorothiazide 25mg tablets | Combined | Angiotensin receptor blocker / Thiazide diuretic |
| 9292641000033116 | Tolucombi 40mg/12.5mg tablets | Combined | Angiotensin receptor blocker / Thiazide diuretic |
| 9292741000033112 | Tolucombi 80mg/12.5mg tablets | Combined | Angiotensin receptor blocker / Thiazide diuretic |
| 9292741000033113 | Tolucombi 80mg/12.5mg tablets | Combined | Angiotensin receptor blocker/ Thiazide diuretic |
| 9292841000033115 | Tolucombi 80mg/25mg tablets | Combined | Angiotensin receptor blocker/ Thiazide diuretic |
| 9292841000033116 | Tolucombi 80mg/25mg tablets | Combined | Angiotensin receptor blocker / Thiazide diuretic |
| 8264941000033119 | Triapin 2.5mg/2.5mg modified-release tablets | Combined | ACE inhibitor / Calcium channel blocker |
| 1834341000033110 | Triapin 5mg/5mg modified-release tablets | Combined | ACE inhibitor / Calcium channel blocker |
| 1834241000033117 | Triapin Mite Tablets | Combined | ACE inhibitor / Calcium channel blocker |
| 3161441000033112 | Valsartan 160mg / Hydrochlorothiazide 12.5mg tablets | Combined | Angiotensin receptor blocker / Thiazide diuretic |
| 3161541000033113 | Valsartan 160mg / Hydrochlorothiazide 25mg tablets | Combined | Angiotensin receptor blocker / Thiazide diuretic |
| 3190741000033112 | Valsartan 80mg / Hydrochlorothiazide 12.5mg tablets | Combined | Angiotensin receptor blocker / Thiazide diuretic |
| 3163041000033116 | Verapamil 180mg modified-release / Trandolapril 2mg capsules | Combined | ACE inhibitor / Calcium channel blocker |
| 1551841000033118 | Zestoretic 10 tablets | Combined | ACE inhibitor / Thiazide diuretic |
| 1552341000033118 | Zestoretic 20 tablets | Combined | ACE inhibitor / Thiazide diuretic |

## **Product codes used to identify NSAID prescribing in CPRD Aurum**

| prodcodeid | termfromemis |
| --- | --- |
| 12041000033110 | Aceclofenac 100mg tablets |
| 2941000033113 | Acemetacin 60mg capsules |
| 2968941000033119 | Advil 200mg tablets (Wyeth Consumer Healthcare) |
| 22641000033110 | Alrheumat Capsules 50 mg |
| 2969041000033111 | Anadin Ultra Capsules 200 mg |
| 72941000033116 | Apsifen F Effervescent tablets 600 mg |
| 74741000033110 | Apsifen Tablets 200 mg |
| 74841000033117 | Apsifen Tablets 400 mg |
| 74941000033113 | Apsifen Tablets 600 mg |
| 2731241000033118 | Arcoxia 120mg tablets (Organon Pharma (UK) Ltd) |
| 4425241000033117 | Arcoxia 30mg tablets (Organon Pharma (UK) Ltd) |
| 2731041000033114 | Arcoxia 60mg tablets (Organon Pharma (UK) Ltd) |
| 2731141000033113 | Arcoxia 90mg tablets (Organon Pharma (UK) Ltd) |
| 81641000033113 | Arthrofen 200 tablets (Ashbourne Pharmaceuticals Ltd) |
| 81741000033116 | Arthrofen 400 tablets (Ashbourne Pharmaceuticals Ltd) |
| 81841000033114 | Arthrofen 600 tablets (Ashbourne Pharmaceuticals Ltd) |
| 80741000033114 | Arthrotec 50 gastro-resistant tablets (Pfizer Ltd) |
| 80941000033112 | Arthrotec 75 gastro-resistant tablets (Pfizer Ltd) |
| 2105241000033115 | Arthroxen Tablets 250 mg |
| 2105341000033113 | Arthroxen Tablets 500 mg |
| 77641000033113 | Artracin Capsules 25 mg |
| 78941000033119 | Artracin Sr M/R capsules 75 mg |
| 13862341000033115 | Aspirin 325mg / Caffeine 15mg tablets |
| 5324141000033111 | Axorid 100mg/20mg modified-release capsules (Meda Pharmaceuticals Ltd) |
| 5324041000033112 | Axorid 200mg/20mg modified-release capsules (Meda Pharmaceuticals Ltd) |
| 97041000033114 | Azapropazone 300mg capsules |
| 99041000033117 | Azapropazone 600mg tablets |
| 138141000033114 | Benoral 2g granules sachets (Sanofi-Synthelabo Ltd) |
| 139941000033118 | Benoral 2g/5ml oral suspension (Sanofi-Synthelabo Ltd) |
| 142341000033111 | Benoral 750mg tablets (Sanofi-Synthelabo Ltd) |
| 138241000033119 | Benorilate 2g granules sachets sugar free |
| 3083341000033110 | Benorilate 2g/5ml oral suspension sugar free |
| 3083441000033116 | Benorilate 750mg tablets |
| 2799441000033111 | Berlind 75 Retard capsules (Tillomed Laboratories Ltd) |
| 2916541000033113 | Bextra 10mg tablets (Pfizer Ltd) |
| 2916641000033114 | Bextra 20mg tablets (Pfizer Ltd) |
| 2916741000033117 | Bextra 40mg tablets (Pfizer Ltd) |
| 1579741000033114 | Brexidol 20mg tablets (Chiesi Ltd) |
| 161341000033113 | Brufen 100mg/5ml syrup (Mylan) |
| 162841000033113 | Brufen 200mg tablets (Abbott Laboratories Ltd) |
| 162941000033117 | Brufen 400mg tablets (Mylan) |
| 157841000033115 | Brufen 600mg effervescent granules sachets (Mylan) |
| 163041000033110 | Brufen 600mg tablets (Mylan) |
| 159441000033110 | Brufen Retard 800mg tablets (Mylan) |
| 173141000033119 | Butacote 100mg gastro-resistant tablets (Novartis Pharmaceuticals UK Ltd) |
| 173241000033114 | Butacote Tablets 200 mg |
| 173341000033116 | Butazolidin Tablets 100 mg |
| 173441000033110 | Butazolidin Tablets 200 mg |
| 2871341000033112 | Calprofen 100mg/5ml oral suspension (McNeil Products Ltd) |
| 2103241000033116 | Celebrex 100mg capsules (Upjohn UK Ltd) |
| 2103341000033114 | Celebrex 200mg capsules (Upjohn UK Ltd) |
| 2103041000033112 | Celecoxib 100mg capsules |
| 2103141000033111 | Celecoxib 200mg capsules |
| 5707441000033119 | Celecoxib 400mg capsules |
| 286341000033119 | Clinoril 100mg tablets (Organon Pharma (UK) Ltd) |
| 286441000033113 | Clinoril 200mg tablets (Organon Pharma (UK) Ltd) |
| 261441000033116 | Clotam Capsules 200 mg |
| 285541000033115 | Clotam Rapid 200mg tablets (Galen Ltd) |
| 336741000033117 | Codafen Continus tablets (Napp Pharmaceuticals Ltd) |
| 11781641000033113 | Combogesic 500mg/150mg tablets (Thornton & Ross Ltd) |
| 4432641000033114 | Cuprofen 200mg tablets (SSL International Plc) |
| 3077941000033110 | Cuprofen PLUS tablets (SSL International Plc) |
| 2780441000033116 | Defanac 25mg gastro-resistant tablets (Ranbaxy (UK) Ltd) |
| 2780541000033115 | Defanac 50mg gastro-resistant tablets (Ranbaxy (UK) Ltd) |
| 2295441000033118 | Defanac 75mg SR tablets (Ranbaxy (UK) Ltd) |
| 2716541000033115 | Defanac Retard 100mg tablets (Ranbaxy (UK) Ltd) |
| 2295541000033117 | Defanac Sr M/R tablets 100 mg |
| 3334141000033116 | Dexibuprofen 300mg tablets |
| 3334241000033111 | Dexibuprofen 400mg tablets |
| 1795841000033111 | Dexketoprofen 25mg tablets |
| 13469741000033119 | Diclo-SR 75mg tablets (Strides Pharma UK Ltd) |
| 442241000033119 | Diclofenac + Misoprostol E/c tablets |
| 5809541000033110 | Diclofenac 10mg dispersible tablets |
| 441941000033116 | Diclofenac 50mg dispersible tablets sugar free |
| 10494141000033115 | Diclofenac 50mg/5ml oral solution |
| 12361741000033115 | Diclofenac 50mg/5ml oral suspension |
| 461841000033117 | Diclofenac Retard Tablets 100 mg |
| 467141000033117 | Diclofenac Sodium Tablets 25 mg |
| 467241000033112 | Diclofenac Sodium Tablets 50 mg |
| 4657241000033111 | Diclofenac potassium 12.5mg tablets |
| 1848741000033117 | Diclofenac potassium 25mg tablets |
| 1848841000033110 | Diclofenac potassium 50mg tablets |
| 453341000033119 | Diclofenac sodium 100mg modified-release capsules |
| 467041000033116 | Diclofenac sodium 100mg modified-release tablets |
| 1922741000033112 | Diclofenac sodium 25mg gastro-resistant tablets |
| 1845841000033118 | Diclofenac sodium 50mg gastro-resistant / Misoprostol 200microgram tablets |
| 1922841000033119 | Diclofenac sodium 50mg gastro-resistant tablets |
| 1845941000033114 | Diclofenac sodium 75mg gastro-resistant / Misoprostol 200microgram tablets |
| 442141000033114 | Diclofenac sodium 75mg gastro-resistant modified-release capsules |
| 453541000033114 | Diclofenac sodium 75mg modified-release capsules |
| 451541000033113 | Diclofenac sodium 75mg modified-release tablets |
| 463141000033110 | Dicloflex 25mg gastro-resistant tablets (Dexcel-Pharma Ltd) |
| 463241000033115 | Dicloflex 50mg gastro-resistant tablets (Dexcel-Pharma Ltd) |
| 1727941000033118 | Dicloflex 75mg SR tablets (Dexcel-Pharma Ltd) |
| 453641000033110 | Dicloflex M/R tablets 100 mg |
| 9995841000033113 | Dicloflex Retard 100mg tablets (Dexcel-Pharma Ltd) |
| 452241000033117 | Diclomax Retard 100mg capsules (Galen Ltd) |
| 453441000033113 | Diclomax SR 75mg capsules (Galen Ltd) |
| 2104841000033115 | Diclovol 25mg gastro-resistant tablets (Arun Pharmaceuticals Ltd) |
| 2104941000033111 | Diclovol 50mg gastro-resistant tablets (Arun Pharmaceuticals Ltd) |
| 1905241000033117 | Diclovol 75mg SR tablets (Arun Pharmaceuticals Ltd) |
| 1905341000033110 | Diclovol Retard 100mg tablets (Arun Pharmaceuticals Ltd) |
| 462341000033117 | Diclozip 25mg gastro-resistant tablets (Ashbourne Pharmaceuticals Ltd) |
| 462441000033111 | Diclozip 50mg gastro-resistant tablets (Ashbourne Pharmaceuticals Ltd) |
| 467841000033111 | Diflunisal 250mg tablets |
| 467941000033115 | Diflunisal 500mg tablets |
| 2104141000033114 | Digenac Xl 100 M/R tablets 100 mg |
| 438741000033114 | Disalcid Capsules 500 mg |
| 481041000033114 | Dolobid 250mg tablets (Organon Pharma (UK) Ltd) |
| 481141000033113 | Dolobid 500mg tablets (Organon Pharma (UK) Ltd) |
| 491841000033115 | Dysman 250 capsules (Ashbourne Pharmaceuticals Ltd) |
| 491941000033111 | Dysman 500 tablets (Ashbourne Pharmaceuticals Ltd) |
| 2295841000033115 | Ebretin 200mg capsules (Ranbaxy (UK) Ltd) |
| 2295941000033111 | Ebretin 300mg capsules (Ranbaxy (UK) Ltd) |
| 493341000033111 | Ebufac Tablets 400 mg |
| 2946341000033111 | Eccoxolac 300mg capsules (Meda Pharmaceuticals Ltd) |
| 3078341000033110 | Econac SR 75mg tablets (Advanz Pharma) |
| 3078441000033116 | Econac XL 100mg tablets (Advanz Pharma) |
| 509941000033118 | Emflex 60mg capsules (Merck Serono Ltd) |
| 8113541000033114 | Enstar SR 75 tablets (Ennogen Pharma Ltd) |
| 8113641000033110 | Enstar XL 100 tablets (Ennogen Pharma Ltd) |
| 547241000033111 | Etodolac 200mg capsules |
| 546941000033116 | Etodolac 300mg capsules |
| 548541000033114 | Etodolac 600mg modified-release tablets |
| 550341000033119 | Etodolac Tablets 200 mg |
| 11781141000033115 | Etolyn 600mg modified-release tablets (Mylan) |
| 4010241000033113 | Etopan XL 600mg tablets (Sun Pharmaceutical Industries Europe B.V.) |
| 2731541000033116 | Etoricoxib 120mg tablets |
| 4425141000033112 | Etoricoxib 30mg tablets |
| 2731341000033111 | Etoricoxib 60mg tablets |
| 2731441000033117 | Etoricoxib 90mg tablets |
| 563841000033112 | Feldene 10mg capsules (Pfizer Ltd) |
| 565941000033117 | Feldene 10mg dispersible tablets (Pfizer Ltd) |
| 563941000033116 | Feldene 20 capsules (Pfizer Ltd) |
| 566041000033110 | Feldene 20mg dispersible tablets (Pfizer Ltd) |
| 571741000033113 | Feldene Melt 20mg tablets (Pfizer Ltd) |
| 2787541000033119 | Fenactol 25mg gastro-resistant tablets (Discovery Pharmaceuticals) |
| 2787641000033118 | Fenactol 50mg gastro-resistant tablets (Dexcel-Pharma Ltd) |
| 2787841000033117 | Fenactol Retard 100mg tablets (Dexcel-Pharma Ltd) |
| 2787741000033110 | Fenactol SR 75mg tablets (Discovery Pharmaceuticals) |
| 564041000033119 | Fenbid 300mg Spansules (Mercury Pharma Group Ltd) |
| 564141000033115 | Fenbufen 300mg capsules |
| 570341000033116 | Fenbufen 300mg tablets |
| 574141000033111 | Fenbufen 450mg tablets |
| 566341000033112 | Fenbufen Effervescent tablets 450 mg |
| 578541000033113 | Fenbuzip 300 Capsules 300 mg |
| 594141000033117 | Fenbuzip 300 Tablets 300 mg |
| 594241000033112 | Fenbuzip 450 Tablets 450 mg |
| 567941000033113 | Fenoket 200mg modified-release capsules (Opus Pharmaceuticals Ltd) |
| 570541000033111 | Fenoprofen 300mg tablets |
| 570641000033112 | Fenoprofen 600mg tablets |
| 570441000033110 | Fenoprofen Tablets 200 mg |
| 570741000033115 | Fenopron 300 tablets (Typharm Ltd) |
| 570841000033113 | Fenopron 600 tablets (Typharm Ltd) |
| 2765841000033110 | Feverfen 100mg/5ml oral suspension (Wise Pharmaceuticals Ltd) |
| 3839241000033110 | Flamatak MR 100mg tablets (Actavis UK Ltd) |
| 3836241000033118 | Flamatak MR 75mg tablets (Actavis UK Ltd) |
| 578841000033110 | Flamatrol Capsules 10 mg |
| 578941000033119 | Flamatrol Capsules 20 mg |
| 1730241000033112 | Flamrase 75mg SR tablets (Teva UK Ltd) |
| 588641000033115 | Flamrase SR 100mg tablets (Teva UK Ltd) |
| 594841000033111 | Flamrase Tablets 25 mg |
| 594941000033115 | Flamrase Tablets 50 mg |
| 13057641000033116 | Flarin 200mg capsules (infirst Ltd) |
| 596341000033114 | Flexin-25 Continus tablets (Napp Pharmaceuticals Ltd) |
| 596241000033116 | Flexin-50 Continus tablets (Napp Pharmaceuticals Ltd) |
| 595941000033119 | Flexin-75 Continus tablets (Napp Pharmaceuticals Ltd) |
| 1610441000033114 | Flexotard MR 100mg tablets (Pfizer Ltd) |
| 593941000033118 | Flurbiprofen 100mg tablets |
| 577741000033116 | Flurbiprofen 200mg modified-release capsules |
| 594041000033116 | Flurbiprofen 50mg tablets |
| 1889841000033112 | Flurbiprofen 8.75mg lozenges |
| 614441000033119 | Froben 100mg tablets (Abbott Laboratories Ltd) |
| 614541000033118 | Froben 50mg tablets (Abbott Laboratories Ltd) |
| 609441000033115 | Froben SR 200mg capsules (Abbott Laboratories Ltd) |
| 2273241000033112 | Galprofen 100mg/5ml oral suspension (Galpharm International Ltd) |
| 2968541000033113 | Hedex Ibuprofen 200mg tablets (Omega Pharma Ltd) |
| 747641000033110 | Ibular Tablets 200 mg |
| 747841000033111 | Ibular Tablets 400 mg |
| 747741000033118 | Ibumetin Tablets 200 mg |
| 747941000033115 | Ibumetin Tablets 400 mg |
| 748041000033117 | Ibumetin Tablets 600 mg |
| 13806041000033117 | Ibuprofen 100mg chewable capsules |
| 2765941000033119 | Ibuprofen 100mg/5ml oral suspension |
| 4088341000033115 | Ibuprofen 100mg/5ml oral suspension 5ml sachets sugar free |
| 1739441000033117 | Ibuprofen 100mg/5ml oral suspension sugar free |
| 12708841000033116 | Ibuprofen 200mg / Codeine 12.8mg tablets |
| 12496141000033117 | Ibuprofen 200mg / Phenylephrine 6.1mg tablets |
| 4123241000033119 | Ibuprofen 200mg / Pseudoephedrine hydrochloride 30mg tablets |
| 4451841000033119 | Ibuprofen 200mg capsules |
| 10643541000033117 | Ibuprofen 200mg modified-release capsules |
| 2839441000033116 | Ibuprofen 200mg orodispersible tablets sugar free |
| 747341000033119 | Ibuprofen 200mg tablets |
| 13177941000033116 | Ibuprofen 200mg/5ml oral suspension sugar free |
| 3229141000033111 | Ibuprofen 300mg modified-release / Codeine 20mg tablets |
| 4158541000033113 | Ibuprofen 300mg modified-release capsules |
| 11790641000033116 | Ibuprofen 400mg capsules |
| 747441000033113 | Ibuprofen 400mg tablets |
| 746841000033113 | Ibuprofen 600mg effervescent granules sachets |
| 747541000033114 | Ibuprofen 600mg tablets |
| 746941000033117 | Ibuprofen 800mg modified-release tablets |
| 746541000033111 | Ibuprofen 800mg tablets |
| 746341000033116 | Ibuprofen Capsules 300 mg |
| 747241000033112 | Ibuprofen Syrup 100 mg/5 ml |
| 4661241000033110 | Ibuprofen lysine 200mg tablets |
| 5376641000033111 | Ibuprofen lysine 400mg tablets |
| 751841000033119 | Imbrilon Capsules 25 mg |
| 751941000033110 | Imbrilon Capsules 50 mg |
| 756841000033110 | Indocid 25mg capsules (Organon Pharma (UK) Ltd) |
| 756941000033119 | Indocid 50mg capsules (Organon Pharma (UK) Ltd) |
| 767941000033114 | Indocid 5mg/ml oral suspension (Organon Pharma (UK) Ltd) |
| 757041000033118 | Indocid R 75mg capsules (Organon Pharma (UK) Ltd) |
| 757141000033119 | Indoflex Capsules 25 mg |
| 757241000033114 | Indolar SR 75mg capsules (Sandoz Ltd) |
| 767741000033111 | Indomax 75 SR capsules (Ashbourne Pharmaceuticals Ltd) |
| 13122441000033111 | Indometacin 10mg/5ml oral suspension |
| 3093041000033114 | Indometacin 25mg capsules |
| 3093641000033115 | Indometacin 25mg modified-release capsules |
| 3093341000033111 | Indometacin 25mg modified-release tablets |
| 5996241000033113 | Indometacin 25mg/5ml oral solution |
| 4452141000033117 | Indometacin 25mg/5ml oral suspension |
| 4447641000033110 | Indometacin 25mg/5ml oral suspension sugar free |
| 3093141000033113 | Indometacin 50mg capsules |
| 3093441000033117 | Indometacin 50mg modified-release tablets |
| 3093741000033112 | Indometacin 75mg modified-release capsules |
| 3093541000033116 | Indometacin 75mg modified-release tablets |
| 757341000033116 | Indomod 25mg modified-release capsules (Pfizer Ltd) |
| 757441000033110 | Indomod 75mg modified-release capsules (Pfizer Ltd) |
| 2128241000033118 | Jomethid XL 200mg capsules (Actavis UK Ltd) |
| 792341000033114 | Junifen Suspension 100 mg/5 ml |
| 1795941000033115 | Keral 25mg tablets (A. Menarini Farmaceutica Internazionale SRL) |
| 801841000033114 | Ketocid 200 modified-release capsules (Chiesi Ltd) |
| 5324341000033114 | Ketoprofen 100mg / Omeprazole 20mg modified-release capsules |
| 797241000033119 | Ketoprofen 100mg capsules |
| 797841000033115 | Ketoprofen 100mg modified-release capsules |
| 801541000033112 | Ketoprofen 150mg modified-release capsules |
| 5324241000033116 | Ketoprofen 200mg / Omeprazole 20mg modified-release capsules |
| 797941000033111 | Ketoprofen 200mg modified-release capsules |
| 797441000033118 | Ketoprofen 50mg capsules |
| 797341000033112 | Ketoprofen Capsules 200 mg |
| 801641000033113 | Ketovail 100mg modified-release capsules (Teva UK Ltd) |
| 801741000033116 | Ketovail 200mg modified-release capsules (Teva UK Ltd) |
| 801941000033118 | Ketozip 200 XL capsules (Ashbourne Pharmaceuticals Ltd) |
| 2104441000033118 | Larafen CR 200mg capsules (Ennogen Pharma Ltd) |
| 808641000033115 | Larapam (Piroxicam) Capsules 10 mg |
| 808741000033112 | Larapam (Piroxicam) Capsules 20 mg |
| 819641000033114 | Lederfen 300mg capsules (Mercury Pharma Group Ltd) |
| 825341000033113 | Lederfen 300mg tablets (Mercury Pharma Group Ltd) |
| 825041000033111 | Lederfen 450mg tablets (Mercury Pharma Group Ltd) |
| 820941000033115 | Lederfen Effervescent tablets 450 mg |
| 825441000033119 | Lederfen F Tablets 450 mg |
| 1627241000033117 | Librofem 200mg tablets (LPC Medical (UK) Ltd) |
| 838341000033119 | Lidifen Tablets 200 mg |
| 838441000033113 | Lidifen Tablets 400 mg |
| 838741000033118 | Lodine Capsules 200 mg |
| 841941000033111 | Lodine Capsules 300 mg |
| 845041000033113 | Lodine SR 600mg tablets (Almirall Ltd) |
| 849841000033116 | Lodine Tablets 200 mg |
| 852141000033115 | Lofensaid 25mg gastro-resistant tablets (Opus Pharmaceuticals Ltd) |
| 852241000033110 | Lofensaid 50mg gastro-resistant tablets (Opus Pharmaceuticals Ltd) |
| 845241000033117 | Lofensaid Retard 100 tablets (Opus Pharmaceuticals Ltd) |
| 845341000033110 | Lofensaid Retard 75 tablets (Opus Pharmaceuticals Ltd) |
| 2638441000033118 | Lornoxicam 4mg tablets |
| 2638541000033117 | Lornoxicam 8mg tablets |
| 3189841000033112 | Lumiracoxib 100mg tablets |
| 3190041000033114 | Lumiracoxib 400mg tablets |
| 3189941000033116 | Lumiracoxib Tablets 200 mg |
| 10332641000033116 | Masidemen 50mg/200microgram gastro-resistant tablets (Actavis UK Ltd) |
| 10332741000033113 | Masidemen 75mg/200microgram gastro-resistant tablets (Actavis UK Ltd) |
| 863541000033114 | Maximet Sr M/R capsules 75 mg |
| 874641000033117 | Mefenamic Acid Dispersible tablets 250 mg |
| 889141000033118 | Mefenamic Acid Paediatric suspension 50 mg/5 ml |
| 872641000033110 | Mefenamic acid 250mg capsules |
| 895741000033116 | Mefenamic acid 500mg tablets |
| 2661841000033116 | Mefenamic acid 50mg/5ml oral suspension |
| 873241000033117 | Meflam Capsules 250 mg |
| 898941000033113 | Meflam Tablets 500 mg |
| 8617841000033117 | Meloxicam 15mg orodispersible tablets sugar free |
| 899241000033112 | Meloxicam 15mg tablets |
| 8617741000033110 | Meloxicam 7.5mg orodispersible tablets sugar free |
| 899341000033119 | Meloxicam 7.5mg tablets |
| 1654041000033116 | Mendys Capsules 250 mg |
| 8494241000033114 | Misofen 50mg/200microgram gastro-resistant tablets (Morningside Healthcare Ltd) |
| 8494341000033116 | Misofen 75mg/200microgram gastro-resistant tablets (Morningside Healthcare Ltd) |
| 938341000033110 | Mobic 15mg tablets (Boehringer Ingelheim Ltd) |
| 938441000033116 | Mobic 7.5mg tablets (Boehringer Ingelheim Ltd) |
| 939041000033117 | Mobiflex 20mg tablets (Mylan) |
| 924741000033116 | Mobiflex Effervescent tablets 20 mg |
| 925141000033119 | Mobiflex Granules 20 mg/sachet |
| 923441000033114 | Mobilan Capsules 25 mg |
| 923541000033110 | Mobilan Capsules 50 mg |
| 930441000033118 | Motifene 75mg modified-release capsules (Daiichi Sankyo UK Ltd) |
| 939741000033119 | Motrin 400mg tablets (Pfizer Ltd) |
| 939841000033112 | Motrin 600mg tablets (Pfizer Ltd) |
| 939941000033116 | Motrin 800mg tablets (Pfizer Ltd) |
| 939641000033111 | Motrin Tablets 200 mg |
| 2850541000033114 | Nabumetone 500mg dispersible tablets sugar free |
| 956241000033115 | Nabumetone 500mg tablets |
| 13746841000033117 | Nabumetone 500mg tablets (Imported) |
| 954341000033118 | Nabumetone Suspension Sugar Free 500 mg/5 ml |
| 954141000033116 | Naprosyn 125mg/5ml oral suspension (Roche Products Ltd) |
| 954841000033110 | Naprosyn 250mg tablets (Atnahs Pharma UK Ltd) |
| 954941000033119 | Naprosyn 500mg tablets (Atnahs Pharma UK Ltd) |
| 949841000033113 | Naprosyn EC 250mg tablets (Atnahs Pharma UK Ltd) |
| 949941000033117 | Naprosyn EC 375mg tablets (Atnahs Pharma UK Ltd) |
| 950041000033114 | Naprosyn EC 500mg tablets (Atnahs Pharma UK Ltd) |
| 950241000033118 | Naprosyn Granules 500 mg |
| 953041000033118 | Naprosyn S/R 500mg tablets (Roche Products Ltd) |
| 956841000033116 | Naprosyn Tablets 375 mg |
| 954241000033111 | Naproxen 125mg/5ml oral suspension |
| 4452241000033112 | Naproxen 125mg/5ml oral suspension sugar free |
| 5973641000033114 | Naproxen 200mg/5ml oral suspension |
| 10976541000033110 | Naproxen 250mg effervescent tablets sugar free |
| 949641000033112 | Naproxen 250mg gastro-resistant tablets |
| 955041000033119 | Naproxen 250mg tablets |
| 11898941000033112 | Naproxen 250mg/5ml oral suspension |
| 950141000033113 | Naproxen 375mg gastro-resistant tablets |
| 6037241000033112 | Naproxen 500mg / Esomeprazole 20mg modified-release tablets |
| 949741000033115 | Naproxen 500mg gastro-resistant tablets |
| 952941000033111 | Naproxen 500mg modified-release tablets |
| 955141000033115 | Naproxen 500mg tablets |
| 3229541000033119 | Naproxen 500mg tablets and Misoprostol 200microgram tablets |
| 950341000033111 | Naproxen Granules 500 mg |
| 952841000033115 | Naproxen M/R tablets 375 mg |
| 956941000033112 | Naproxen Tablets 375 mg |
| 956441000033119 | Naproxen sodium 275mg tablets |
| 12903841000033119 | Nexocin EC 250mg gastro-resistant tablets (Noumed Life Sciences Ltd) |
| 12903941000033110 | Nexocin EC 375mg gastro-resistant tablets (Noumed Life Sciences Ltd) |
| 12904041000033112 | Nexocin EC 500mg gastro-resistant tablets (Noumed Life Sciences Ltd) |
| 2970741000033111 | Nurofen 200mg caplets (Reckitt Benckiser Healthcare (UK) Ltd) |
| 2971241000033112 | Nurofen 200mg liquid capsules (Reckitt Benckiser Healthcare (UK) Ltd) |
| 2616241000033112 | Nurofen Advance Tablets |
| 3248541000033111 | Nurofen Back Pain SR 300mg capsules (Reckitt Benckiser Healthcare (UK) Ltd) |
| 4123741000033113 | Nurofen Cold and Flu tablets (Reckitt Benckiser Healthcare (UK) Ltd) |
| 11790741000033113 | Nurofen Express 400mg liquid capsules (Reckitt Benckiser Healthcare (UK) Ltd) |
| 1746541000033111 | Nurofen For Children Sugar-free suspension 100 mg/5 ml |
| 2616341000033119 | Nurofen Long Lasting 300mg capsules (Crookes Healthcare Ltd) |
| 5376741000033119 | Nurofen Maximum Strength Migraine Pain 684mg caplets (Reckitt Benckiser Healthcare (UK) Ltd) |
| 2839541000033115 | Nurofen Meltlets 200mg tablets (Reckitt Benckiser Healthcare (UK) Ltd) |
| 2228341000033113 | Nurofen Plus tablets (Reckitt Benckiser Healthcare (UK) Ltd) |
| 4803341000033111 | Nurofen for Children 100mg/5ml oral suspension orange (Reckitt Benckiser Healthcare (UK) Ltd) |
| 4803141000033113 | Nurofen for Children 100mg/5ml oral suspension strawberry (Reckitt Benckiser Healthcare (UK) Ltd) |
| 13579141000033115 | Nurofen for Children 200mg/5ml oral suspension orange (Reckitt Benckiser Healthcare (UK) Ltd) |
| 13579241000033110 | Nurofen for Children 200mg/5ml oral suspension strawberry (Reckitt Benckiser Healthcare (UK) Ltd) |
| 4088541000033110 | Nurofen for Children Singles 100mg/5ml oral suspension 5ml sachets orange (Reckitt Benckiser Healthcare (UK) Ltd) |
| 4088441000033114 | Nurofen for Children Singles 100mg/5ml oral suspension 5ml sachets strawberry (Reckitt Benckiser Healthcare (UK) Ltd) |
| 6386841000033117 | Nuromol 200mg/500mg tablets (Reckitt Benckiser Healthcare (UK) Ltd) |
| 994741000033112 | Nycopren 250mg gastro-resistant tablets (Ardern Healthcare Ltd) |
| 994841000033119 | Nycopren 500mg gastro-resistant tablets (Ardern Healthcare Ltd) |
| 5707541000033118 | Onsenal 400mg capsules (Pfizer Ltd) |
| 1006441000033115 | Opustan Capsules 250 mg |
| 1009141000033118 | Opustan Tablets 500 mg |
| 2797741000033114 | Orbifen For Children 100mg/5ml oral suspension (Orbis Consumer Products Ltd) |
| 1010141000033111 | Orudis 100mg capsules (Sanofi) |
| 1010241000033116 | Orudis 50mg capsules (Sanofi) |
| 1010341000033114 | Oruvail 100 modified-release capsules (Sanofi) |
| 1013741000033118 | Oruvail 150 modified-release capsules (Sanofi) |
| 1013841000033111 | Oruvail 200 modified-release capsules (Sanofi) |
| 1010441000033115 | Oruvail S/r capsules 200 mg |
| 11781541000033112 | Paracetamol 500mg / Ibuprofen 150mg tablets |
| 6386741000033110 | Paracetamol 500mg / Ibuprofen 200mg tablets |
| 2128341000033111 | Pardelprin MR 75mg capsules (Actavis UK Ltd) |
| 1043441000033116 | Paxofen Tablets 200 mg |
| 1043541000033115 | Paxofen Tablets 400 mg |
| 1043641000033119 | Paxofen Tablets 600 mg |
| 1082041000033110 | Phenylbutazone 100mg tablets |
| 1082241000033119 | Phenylbutazone 200mg tablets |
| 1083641000033112 | Piroflam Capsules 10 mg |
| 1083741000033115 | Piroflam Capsules 20 mg |
| 1083441000033110 | Piroxicam 10mg capsules |
| 1084541000033113 | Piroxicam 10mg dispersible tablets |
| 1083541000033111 | Piroxicam 20mg capsules |
| 1084641000033114 | Piroxicam 20mg dispersible tablets |
| 1088641000033118 | Piroxicam 20mg orodispersible tablets sugar free |
| 1666941000033115 | Piroxicam betadex 20mg tablets |
| 1083241000033114 | Pirozip 10 capsules (Ashbourne Pharmaceuticals Ltd) |
| 1083341000033116 | Pirozip 20 capsules (Ashbourne Pharmaceuticals Ltd) |
| 1096241000033119 | Ponstan 250mg capsules (Chemidex Pharma Ltd) |
| 1098341000033113 | Ponstan Dispersible Dispersible tablets 250 mg |
| 1109541000033115 | Ponstan Forte 500mg tablets (Chemidex Pharma Ltd) |
| 1106341000033116 | Ponstan Paediatric suspension 50 mg/5 ml |
| 1124141000033117 | Pranoxen Continus M/R tablets 375 mg |
| 1124241000033112 | Pranoxen Continus M/R tablets 500 mg |
| 1136241000033118 | Preservex 100mg tablets (Almirall Ltd) |
| 3190141000033113 | Prexige 100mg tablets (Novartis Pharmaceuticals UK Ltd) |
| 3190341000033111 | Prexige 400mg tablets (Novartis Pharmaceuticals UK Ltd) |
| 3190241000033118 | Prexige Tablets 200 mg |
| 1153141000033116 | Ramodar Tablets 200 mg |
| 2850641000033110 | Relifex 500mg dispersible tablets (Meda Pharmaceuticals Ltd) |
| 1164641000033119 | Relifex 500mg tablets (Mylan) |
| 1164141000033112 | Relifex Suspension 500 mg/5 ml |
| 1168141000033117 | Rheumacin LA 75mg capsules (Hillcross Pharmaceuticals Ltd) |
| 2274341000033116 | Rheumatac Retard 75 tablets (Advanz Pharma) |
| 1168041000033116 | Rheumox 300mg capsules (Mercury Pharma Group Ltd) |
| 1168941000033115 | Rheumox 600mg tablets (Mercury Pharma Group Ltd) |
| 1673141000033118 | Rhumalgan CR 100 tablets (Sandoz Ltd) |
| 1673241000033113 | Rhumalgan CR 75 tablets (Sandoz Ltd) |
| 1168241000033112 | Rhumalgan M/R tablets 100 mg |
| 1168341000033119 | Rhumalgan M/R tablets 75 mg |
| 3217541000033117 | Rhumalgan SR 75mg capsules (Almus Pharmaceuticals Ltd) |
| 1169041000033112 | Rhumalgan Tablets 25 mg |
| 1169141000033111 | Rhumalgan Tablets 50 mg |
| 3217641000033116 | Rhumalgan XL 100mg capsules (Sandoz Ltd) |
| 1803941000033119 | Rofecoxib 12.5mg tablets |
| 1804141000033118 | Rofecoxib 12.5mg/5ml oral suspension sugar free |
| 1804041000033117 | Rofecoxib 25mg tablets |
| 1804241000033113 | Rofecoxib 25mg/5ml oral suspension sugar free |
| 2636741000033112 | Rofecoxib 50mg tablets |
| 1222241000033117 | Salsalate Capsules 500 mg |
| 3334341000033118 | Seractil 300mg tablets (Gebro Pharma GmbH) |
| 3334441000033112 | Seractil 400mg tablets (Thornton & Ross Ltd) |
| 12471641000033112 | Skudexa 75mg/25mg tablets (A. Menarini Farmaceutica Internazionale SRL) |
| 1346341000033117 | Slo-Indo 75mg capsules (Mylan) |
| 1346841000033114 | Slofenac 75mg SR tablets (Sterwin Medicines) |
| 1346741000033116 | Slofenac Sr M/R tablets 100 mg |
| 9292941000033111 | Solpadeine Max 12.8mg/500mg tablets (Omega Pharma Ltd) |
| 2968641000033114 | Solpaflex tablets (GlaxoSmithKline Consumer Healthcare) |
| 10976641000033111 | Stirlescent 250mg effervescent tablets (Stirling Anglian Pharmaceuticals Ltd) |
| 1889941000033116 | Strefen Honey and Lemon 8.75mg lozenges (Reckitt Benckiser Healthcare (UK) Ltd) |
| 2871841000033115 | Streflam Lozenges 8.75 mg |
| 1396041000033114 | Sulindac 100mg tablets |
| 1396141000033113 | Sulindac 200mg tablets |
| 1398841000033115 | Surgam 200mg tablets (Sanofi) |
| 1396641000033115 | Surgam 300mg tablets (Sanofi) |
| 1389341000033118 | Surgam Capsules 300 mg |
| 1389641000033114 | Surgam SA 300mg capsules (Sanofi) |
| 1394341000033116 | Surgam Sachets 300 mg |
| 1403341000033114 | Synflex 275mg tablets (Roche Products Ltd) |
| 1425241000033112 | Tenoxicam 20mg tablets |
| 1416641000033111 | Tenoxicam Effervescent tablets 20 mg |
| 1417941000033119 | Tenoxicam Granules 20 mg/sachet |
| 1443041000033118 | Tiaprofenic Acid Sachets 300 mg |
| 1444841000033110 | Tiaprofenic acid 200mg tablets |
| 1442941000033111 | Tiaprofenic acid 300mg modified-release capsules |
| 1443741000033115 | Tiaprofenic acid 300mg tablets |
| 3136641000033111 | Tiloket 50mg capsules (Tillomed Laboratories Ltd) |
| 2781041000033116 | Tiloket CR 100mg capsules (Tillomed Laboratories Ltd) |
| 2781141000033117 | Tiloket CR 200mg capsules (Tillomed Laboratories Ltd) |
| 1700241000033117 | Timpron 250 Ec E/c tablets 250 mg |
| 1700341000033110 | Timpron 500 Ec E/c tablets 500 mg |
| 1443441000033110 | Timpron Tablets 250 mg |
| 1443541000033111 | Timpron Tablets 500 mg |
| 1445341000033117 | Tolectin Capsules 200 mg |
| 1445041000033119 | Tolectin Capsules 400 mg |
| 1445141000033115 | Tolfenamic Acid Capsules 200 mg |
| 1451941000033119 | Tolfenamic acid 200mg tablets |
| 1445441000033111 | Tolmetin Capsules 200 mg |
| 1445241000033110 | Tolmetin Capsules 400 mg |
| 12471541000033111 | Tramadol 75mg / Dexketoprofen 25mg tablets |
| 2916241000033111 | Valdecoxib 10mg tablets |
| 2916341000033118 | Valdecoxib 20mg tablets |
| 2916441000033112 | Valdecoxib 40mg tablets |
| 2730341000033115 | Valdic 100 Retard tablets (Fannin UK Ltd) |
| 2730241000033113 | Valdic 75 Retard tablets (Fannin UK Ltd) |
| 1500341000033114 | Valenac Tablets 25 mg |
| 1500441000033115 | Valenac Tablets 50 mg |
| 6018341000033114 | Valket 200 Retard capsules (Tillomed Laboratories Ltd) |
| 6037341000033119 | Vimovo 500mg/20mg modified-release tablets (AstraZeneca UK Ltd) |
| 1804541000033110 | Vioxx 12.5mg tablets (Organon Pharma (UK) Ltd) |
| 1804341000033115 | Vioxx 12.5mg/5ml oral suspension (Organon Pharma (UK) Ltd) |
| 1804641000033111 | Vioxx 25mg tablets (Organon Pharma (UK) Ltd) |
| 1804441000033114 | Vioxx 25mg/5ml oral suspension (Organon Pharma (UK) Ltd) |
| 2636841000033119 | VioxxAcute 25mg tablets (Organon Pharma (UK) Ltd) |
| 2636941000033110 | VioxxAcute 50mg tablets (Organon Pharma (UK) Ltd) |
| 1527541000033111 | Volraman 25mg gastro-resistant tablets (LPC Medical (UK) Ltd) |
| 1527641000033112 | Volraman 50mg gastro-resistant tablets (LPC Medical (UK) Ltd) |
| 2779041000033113 | Volsaid Retard 100 tablets (Chiesi Ltd) |
| 2778941000033116 | Volsaid Retard 75 tablets (Chiesi Ltd) |
| 1526141000033116 | Volsaid Sr 100 M/R tablets 100 mg |
| 1526041000033115 | Volsaid Sr 75 M/R tablets 75 mg |
| 1527041000033118 | Voltarol 25mg gastro-resistant tablets (Novartis Pharmaceuticals UK Ltd) |
| 1525541000033119 | Voltarol 50mg dispersible tablets (Novartis Pharmaceuticals UK Ltd) |
| 1527141000033119 | Voltarol 50mg gastro-resistant tablets (Novartis Pharmaceuticals UK Ltd) |
| 1526341000033118 | Voltarol 75mg SR tablets (Novartis Pharmaceuticals UK Ltd) |
| 4657341000033118 | Voltarol Pain-eze 12.5mg tablets (Novartis Consumer Health UK Ltd) |
| 6430441000033118 | Voltarol Pain-eze Extra Strength 25mg tablets (Novartis Consumer Health UK Ltd) |
| 1848941000033119 | Voltarol Rapid 25mg tablets (Novartis Pharmaceuticals UK Ltd) |
| 1849041000033111 | Voltarol Rapid 50mg tablets (Novartis Pharmaceuticals UK Ltd) |
| 1527241000033114 | Voltarol Retard 100mg tablets (Novartis Pharmaceuticals UK Ltd) |
| 2638741000033113 | Xefo 4mg tablets (CeNeS Pharmaceuticals Plc) |
| 2638841000033115 | Xefo 8mg tablets (CeNeS Pharmaceuticals Plc) |
